# Effects of adaptation to crowded larval environment on the evolution of sperm competitive ability in males of *Drosophila melanogaster*

**DOI:** 10.1080/19336934.2024.2437204

**Published:** 2024-12-18

**Authors:** Rohit Kapila, Komal Maggu, Neetika Ahlawat, Nagaraj Guru Prasad

**Affiliations:** a Department of Biology, Florida International University, Miami, Florida, USA; b Department of Evolutionary Biology and Environmental Studies, University of Zurich, Winterthurerstrasse, Switzerland; c Department of Chemical Engineering, Indian Institute of Technology Bombay, Mumbai, India; d Department of Biological Sciences, Indian Institute of Science Education and Research (IISER), Mohali, India

**Keywords:** Experimental evolution, larval crowding, *Drosophila melanogaster*, sperm competition

## Abstract

Two of the most important environmental factors that affect the sperm competitive ability in males are the availability of resources and the socio-sexual environment. Numerous studies have investigated the individual effects of these factors, but their combined effect on the evolution of sperm competitive ability remains untested. A crowded larval environment is unique because it simultaneously affects the fitness of the organism through both resource availability and the socio-sexual environment. In this study, we used a set of four laboratory populations of *D. melanogaster*, evolved under a crowded larval environment for more than 165 generations and their respective controls to investigate how the sperm competitive ability of the males is affected by a single generation of larval crowding versus evolution under a crowded larval environment for more than 165 generations. Our results show that larval crowding negatively affects the sperm defence ability of males evolved in a crowded larval environment, while it has no effect on the sperm defence ability of control males. Additionally, larval crowding negatively impacts the sperm offence ability in both control and evolved populations. Males from populations adapted to a crowded larval environment exhibit lower sperm offence ability at an older age compared to control populations.

## Introduction

Sperm competition is a widely observed phenomenon in promiscuous species. It is quantified by two components: a) Sperm defence (P1) – paternity share of the first male mating with the female, b) Sperm offence (P2) – paternity share of the male mating with a non-virgin female [1–3]. Various studies have shown that socio-sexual environment [4,5] and the availability of resources [6–8] are the two major factors affecting sperm competitive ability of males. With respect to socio-sexual environment, theoretical predictions suggest that mating expenditure of males should increase with increasing risk of sperm competition but should decrease with increasing intensity of sperm competition [9–11]. There is evidence both supporting [12–16] and opposing [17] these predictions. Previous research has demonstrated that tactile cues, like larval densities, function as reliable indicators of future adult densities which triggers socially cued anticipatory plasticity, a mechanism that induces developmental shifts [18]. This phenomenon has been observed in various invertebrates [19,20] where individuals exhibit allocation shifts in response to changing tactile cues. For example, studies have shown that when male insects are raised in higher larval densities, they experience extended development times and develop larger testes compared to those raised in lower densities [21,22].

With respect to nutritional requirements, it is well known that sperm performance is affected by the diet of the organism [23–26] and change in diet affects the ejaculate quality and sperm competitive abilities of males [27–32].

Earlier experimental evolution studies investigating the reproductive traits of *D. melanogaster*, established a correlation between sperm length and seminal receptacle (SR) length [33]. Later research highlighted that SR length is more influenced by environmental factors like larval density and nutrition, while sperm length remains relatively stable [34]. More recent studies have demonstrated the increased sensitivity of male fly testes and accessory glands to cues during the larval stages linked with reproductive competition [21]. In the context of these findings, the crowded larval environment emerges as a distinctive setting where both socio-sexual cues and nutritional availability are directly impacted.

Crowded larval environments arise due to the poor locomotor ability of the larvae of some holometabolous insects, as the egg-laying site becomes the primary feeding ground for them, exposing the organisms to increased competition for resources and an environment full of toxic waste products like urea [35–38]. Previous studies have shown that adaptation to a crowded larval environment leads to the evolution of both larval [38] as well as adult traits [39,40] [41,42]. In a crowded larval environment, due to the increased risk of sperm competition, it is expected that males will increase investment in mating [9–11]. At the same time, due to a decrease in nutritional uptake in a crowded environment, the quality of male ejaculates is expected to go down [31]. However, it is important to note that the combined effect of these two factors on sperm competitive ability has not been well studied. Further, the effect of adaptation to development in a crowded larval environment on the evolution of male’s sperm competitive ability remains unexplored. Therefore, in this study, we addressed the following questions:

1. How does growing in a crowded larval environment for a single generation affect sperm competitive ability?
2. Does adaptation to crowded larval environment for more than 165 generations lead to correlated evolution of sperm competitive ability?

Previous studies have shown that the effect of larval crowding on adult reproductive traits differ between single generation of crowding versus long-term adaptation to larval crowding, for example, unlike single-generation studies, long-term adaptation to larval crowding leads to the evolution of increased adult pre-copulatory behaviour [43] —possibly as an overcompensation for smaller body size – without increased investment in reproductive tissues [40]. This suggests that, for corelated evolution of reproductive traits, the effects of a single generation of larval crowding may differ markedly from those resulting from long-term adaptation to crowded conditions. In line with these findings, we hypothesize that long-term adaptation to larval crowding may lead to a negative correlated response in sperm competitive ability. Specifically, the increased investment in pre-copulatory behaviour may come at a trade-off with post-copulatory traits [44], such as sperm defence and offence, as resources are diverted to behaviours that enhance mating opportunities rather than competitive sperm function. To test this hypothesis, we evolved four populations of *D. melanogaster* under crowded larval conditions, along with their respective low larval density control populations for more than 165 generations. In a full-factorial experimental design, we grew larvae from these populations in high and low larval densities. Adults emerging from these cultures were subjected to sperm defence and sperm offence assays against a common competitor male to study the effect of larval crowding on sperm competitive ability.

## Materials and methods

### Experimental system

We used replicate laboratory populations of *D. melanogaster* as our study system. Briefly, we used eight large populations of *D. melanogaster*: four populations selected for adaptation to larval crowding called ‘Melanogaster Crowded as larvae and Uncrowded’ as adult (MCU 1–4), and four ‘Melanogaster Baseline’ populations (MB 1–4), mentioned as crowding-adapted and control populations, respectively, hereafter. MCU (crowding-adapted) and MB (control) populations represented by the same subscript are related by ancestry (i.e. MCU-1 was derived from MB-1; MCU-2 was derived from MB-2, and so on) and are treated as a statistical block. These populations were initially maintained by Prof. Amitabh Joshi’s lab at JNCSAR for 75 generations before they were handed over to us in February 2011, and they have been maintained as per their maintenance regime ever since. Crowding-adapted flies were derived from control populations, which were in turn derived from JB populations. JB flies trace their ancestry to UU populations used by Muller [37], which trace their ancestry back to B populations used by Rose [45] that were maintained in laboratory conditions for several hundred generations at effective population size of 800–1200.

These populations were reared in 8-dram (25 mm diameter × 90 mm height) vials at a density of 800 larvae per 1.5 ml food for crowding-adapted populations and 70 larvae per 6–8 ml food for control populations. Every generation, 24 high larval density vials were cultured for crowding-adapted populations, and 40 low larval density vials were cultured for control populations. Due to very little food and too many larvae in the vials of crowding-adapted populations, the food runs out in those vials during the first 2 to 3 days of development, which causes massive mortality in the vials of crowding-adapted populations. Previous studies on the same and similar populations have shown that the ability to reach the critical minimum size is strongly under selection. There is evidence that this critical minimum size itself might have been reduced in these populations. Further, key adaptation to larval crowding is not rapid development but increased larval competitive ability [38]. Selection pressure has enhanced their food acquisition and survival traits, leading to physiological adaptations such as stress resistance and metabolic efficiency, rather than faster development [38]. As a result of intense larval crowding, only 100–120 adults eclose out of each vial in the crowding-adapted populations. Whereas, there is close to zero larval mortality in the control populations. Adults eclosing out of the vials that belong to the same replicate population were transferred into Plexiglas cages (24 × 19 × 14 cm) with *ad libitum* media and a cotton ball wet with distilled water. Each population has 2400–2800 flies that are maintained in Plexiglas cages. All the populations were maintained on a 21-day discrete generation cycle. At the time of the experiment, each replicate of crowding-adapted populations had undergone a selection for larval crowding for at least 165 generations.

## Standard maintenance protocol of control flies

Every generation, day-zero of the population maintenance cycle was the day when eggs were collected. On this day, 40 culture vials were prepared for each replicate block. In each culture vial 60–70 eggs were placed in 6–8 ml of food. Flies start to eclose as adults 9 days post egg collection, and eclosion peaks at day 10. By the 12th day post egg collection, when almost all the adults had eclosed, the adults were transferred into the Plexiglas cages (24 × 19 × 14 cm) containing a Petri plate of cornmeal-charcoal food and a piece of cotton ball soaked in distilled water (to ensure a humid environment). The adult population size per replicate population was maintained at ~2500. Throughout their maintenance cycle, populations were maintained at a constant temperature of 25°C and 90% RH. A fresh cornmeal-charcoal food plate (refer to Supplementary materials for detailed media recipe) and wet cotton ball was provided on alternate day in the cages. Eighteen days after egg collection, cornmeal-charcoal food plate was supplemented with yeast paste for 2 days. Twenty days after the egg collection, the cornmeal-charcoal yeast food plate was replaced with an egg-collection food plate for 18 h. The eggs laid by the females during those 18 h were collected for setting up the next generation. The simplified sketch of population maintenance scheme is provided in Supplementary Figure S1.

## Standard maintenance protocol of crowding-adapted flies

Just like control populations, crowding-adapted populations were also maintained on a 21-day generation cycle at 25°C and 90% RH. On day zero of the maintenance cycle, 800 eggs were collected and placed in 8-dram vial with 1.5 ml of cornmeal-charcoal food. For each replicate population, enough eggs to fill 24 vials were collected. Due to facing crowded larval environments for several generations, the eclosion pattern of these populations was more spread out across days compared to flies of control populations. Therefore, to avoid adult crowding, adults were transferred into the cages daily from the 8th day till day 18 post egg collection. Fresh cornmeal-charcoal food plate was provided into the cages with a moist cotton ball every alternate day. On day 18 post egg collection, similar to control populations, flies of crowding-adapted populations were also provided with a cornmeal-charcoal food plate supplemented with yeast paste for 2 days, which is followed by a 18-h period of oviposition plate. Eggs laid by the flies during that 18-h period were collected for setting up the next generation. It is noteworthy that this level of intense crowding negatively affected the adult body size of the flies [46]. Studies have shown that bigger flies get a mating advantage [47], however this is not a matter of concern in this study because at no point was a female given a choice between mating with a focal or competitor male. Further, we know from previous studies on the same populations that mating success does not differ between the crowding-adapted and control populations [42].

## Details of common competitor population

For the sperm competition assay, it is crucial to have a heritable phenotypic marker in males from crowding-adapted populations to distinguish them from control population males. Without this, we cannot determine the progeny’s paternity. Since there was no such marker available in these populations, we used a common competitor male in our experiments. Additionally, to eliminate any mating bias by females that might arise due using the females from different populations in the assays, we used females from the same populations as the common competitor males. PJB-W (referred as ‘common competitor’ hereafter) and control populations (MB) share a common ancestor laboratory population JB. Common competitor flies contain a recessive mutation on the X-chromosome which results in a white-eyed phenotype in homozygotes for this mutation. This mutation has no obvious fitness effect. Briefly, common competitor flies were maintained on a 14-day discrete generation cycle, at 25°C, and under constant light. Every generation eggs were collected at a density of 60–70 eggs per vial containing 8–10 ml of standard banana-jaggery food (refer to Supplementary materials for detailed media recipe). On day 12 post egg collection, flies are transferred to a plexiglass cage (24 × 19 × 14 cm) supplied with a petri plate of banana-jaggery food supplemented with ad libitum live yeast paste. On 14th day post egg collection, a plate is provided for 18 h on which females oviposit to produce eggs for the next generation. A total of 40 vials were collected each generation, and the adult census size of this population was approximately 2400 individuals.

## Generation of flies for sperm competition assays

For all the sperm defence and sperm offence assays, files were generated from crowding-adapted and control populations in the same way. Each replicate of crowding-adapted and control population was first subjected to standardization by rearing at low-densities for one generation to get rid of any non-genetic parental effects [45]. Standardized adults were housed in cages with a food plate supplemented with ad-libitum live yeast for 48 h. Subsequently, a fresh food plate was introduced for 6 h for egg laying. For all the sperm defence and sperm offence assays, each replicate of both crowding-adapted and control populations had two treatments:

1. The high-density (HD) treatment with 600 eggs per vial containing 2 ml of food.
2. The low-density treatment (LD) with 70 eggs per vial containing 6 ml of food.

Growing 800 larvae in 1.5 ml of food proved too stressful for males in the control populations, leading to population crashes whenever they were cultured at that density. Therefore, all assays were conducted at a density of 600 larvae in 2 ml of food. The focal males used in the assays were not virgins. Our study aimed to replicate the general maintenance regime of these populations, where males are housed with females in cages for over a week before egg collection. Thus, it is highly unlikely that males would remain virgins by the time of sampling. While we acknowledge the possibility that a male could have been sampled immediately after mating, this is improbable given our large sample size. Further, in a previous study by Shenoi et al. [42], it was reported that on average, one mating event occurred every 30 min in a population of 100 males and 100 females observed over 36 h. This means that only a small proportion of males would be actively mating at any given time. Since all focal males in our study were sampled within a 30-min period, it is unlikely that more than one male per treatment was sampled immediately after mating.

Larval crowding, as well as adaptation to larval crowding, leads to a decrease in the mean and an increase in the variation of development time in *D. melanogaster* populations [48]. Therefore, egg collection for different treatments was done on different days, ensuring the adults were of the same age on the day of their sperm competition assay. In the HD treatment, to avoid adult crowding, adult flies were transferred into mixed-sex cages on the day they eclosed where males and females were allowed to interact. In the low-density cultures, adults were transferred into cages on the 12th day post egg collection. Adults were maintained in cages until the day of their sperm competition assay. Flies generated using this protocol were then used for sperm defence and sperm offence assays.

Different sets of flies were used for each assay. As part of the regular maintenance cycle, on the 18th day post-egg collection, approximately when the flies reach 8–9 days of adulthood, yeast paste supplementation was provided in addition to their standard cornmeal-charcoal diet. This 48-h yeasting period is succeeded by an 18-h egg collection phase, during which only eggs laid by flies were collected for the subsequent generation. Notably, based on findings from prior studies on the same populations [42], this yeasting event on the 18th day post-egg collection signifies the peak in reproductive activities in these populations. Additionally, in most *Drosophila* populations (including the ones used in this study), the reproductive activities peaks 3–4 days post eclosion [49]. Hence, in the present study, we sampled males at day 4 post-eclosion, which represents their natural reproductive peak and at day 9 post-eclosion which corresponds to the egg collection window in their maintenance regime.

## Procedure for sperm defence (P1) assay

Four days after they eclosed, males from the crowding-adapted and control populations that had experienced one of the larval density treatments (focal males) were assayed for their sperm defence (P1) ability. The focal males were randomly sampled from the cages and transferred into separate food vials. A virgin female from the common competitor (PJB-W) population was then introduced into each vial, and mating was observed for an hour. A minimum of 50 mating pairs were set up for each treatment. Flies that did not mate within the first 60 min from the start of observation were excluded from the further assays. After the first mating, focal males were discarded, and a single male from the common competitor population was introduced to each vial. All the vials were then observed for mating over the next 12 h. Females that mated twice, once with the focal male and once with the common competitor male, were transferred into egg-laying vials for the next 24 h. Egg-laying vials were then maintained in the incubator for 12 days while progeny developed. Progeny were scored for paternity based on eye colour. Female progeny sired by focal males had red eyes, while female progeny sired by the common competitor males had white eyes, because their father carried a white eye recessive mutation on their X-chromosome. The proportion of female progeny sired by males of crowding-adapted and control populations against a common competitor male was used as a unit of analysis to measure the sperm defence ability. Final sample sizes across replicate blocks were: crowding-adapted/High density *N* = 147; crowding-adapted/Low density *N* = 215; control/High density *N* = 183; control/Low density *N* = 213. The simplified scheme of sperm defence and sperm offence assay is provided in Supplementary Figure S2.

## Procedure for sperm offence (P2) assay

Since age affects the reproductive fitness of males negatively [50] we conducted sperm offence (P2) assays for males at two different ages, 4 days old and 9 days old. For both ages, all flies from the selected and control populations were generated in the same way as for the P1 assays (see above). For the P2 assays, we followed a similar protocol to the P1 assay except that in the P2 assays, virgin females from the common competitor populations were first mated to the common competitor males, and then the control/crowding-adapted focal males. Since the remating latency of females was very high in the P2 assay, mating with focal males was not observed. Instead, females were kept with males for 24 h and were then transferred to the egg-laying vials. When counting the progeny, vials with no red-eyed female progeny were excluded from further analysis, assuming focal males failed to remate with the female in the 24-h mating window. For 4-day-old males, the final sample sizes combined for all replicate blocks were 400 and 230 for the high-density and low-density treatments, respectively, of the crowding-adapted population. For the control populations, the sample sizes were 364 and 221 for the high-density and low-density treatments, respectively.

For 9-day-old males, the mating success of males with non-virgin common competitor females was even lower than the mating success of 4-day-old males, therefore the final sample sizes combined for all replicate blocks were 215 and 153 for the high-density and low-density treatments, respectively, of the crowding-adapted population. For the control populations, the sample sizes were 246 and 116 for the high-density and low-density treatments, respectively.

For both crowding-adapted and control populations, we measured sperm offence ability for 4-day and 9-day-old males, whereas, sperm defence ability was measured for 4-day-old adults. In the regular maintenance of these populations, eggs are collected from 9- to 10-day-old flies to start the next generation. Therefore, only those eggs that are laid by these flies during an 18-h window, when they are 9–10-days-old as adults, contribute to the next generation. Flies eclosed by day 10 post egg collection were held in mixed sex cages for the next 9–10 days. We know from previous experiments that the flies mate multiple times during this 9–10-day period. In *Drosophila*, there is extremely strong last male precedence with most of the progenies sired by the last male to mate [51]. The proportion of the progeny sired by the first male declines rapidly with each successive mating. Therefore, among the eggs laid during the egg collection window, the fraction of eggs sired by the first male (P1) is likely to be negligibly small. Therefore, measuring the sperm defence ability of 9-day-old males is irrelevant for these flies.

## Statistical analysis

The proportion of female progeny sired by the focal male was arcsine square-root transformed to improve normality. Raw data tables are listed in supplementary materials (Supplementary Tables S1–3). The transformed data were then analysed using a linear mixed-effects model (LMM) in R (version 1.4.1717). The model was implemented using the lmer function from the lme4 package [52] version 1.1–27.1). To access the statistical significance of effects, we conducted Type III ANOVA using car package (Fox & Weisberg 2012, version 3.1.0) [53].

The model formula was:

lmer(P1 ~ SelectionHistory * DensityTreatment + (1 | Block))

lmer(P2 ~ SelectionHistory * DensityTreatment + (1 | Block))

Selection history (adapted vs. control) and density treatment were included as fixed factors, and block was included as random factor to account for the variability among blocks [54]. Further, to dwell deeper into the interaction of selection history and density treatment effects, we conducted a post-hoc Tukey’s Honest Significant Difference (HSD) (Tukey [55], test, using the emmeans (Lenth [56], separately for 9-day-old and 4-day-old males.

To test the robustness of our LMM results, in addition to our primary LMM analysis, we also conducted a Generalised Linear Mixed Model (GLMM) analysis as an additional step using the following formula:

glmer(cbind(Red, White) Selection * Treatment + (1 | Block), family = binomial)

This GLMM approach, implemented with a binomial family, used the proportion of red-eyed versus white-eyed progeny as the response variable.

We also analysed the total number of red-eyed female progeny in each assay using the following model.

lmer(Red~ Selection*Treatment+ (1|Block))

Raw data tables are listed in supplementary materials (Supplementary Tables 4–6)

In addition, we also did a logistic regression analysis for the sperm defence assay where the focal male failed to sire any progeny was coded as ‘success = 0’, while instances where the focal male sired at least one progeny were coded as ‘success = 1’, results of which are discussed in supplementary materials (Supplementary Table 7).

## Results

### Sperm defence (P1) assay

**Figure 1.**
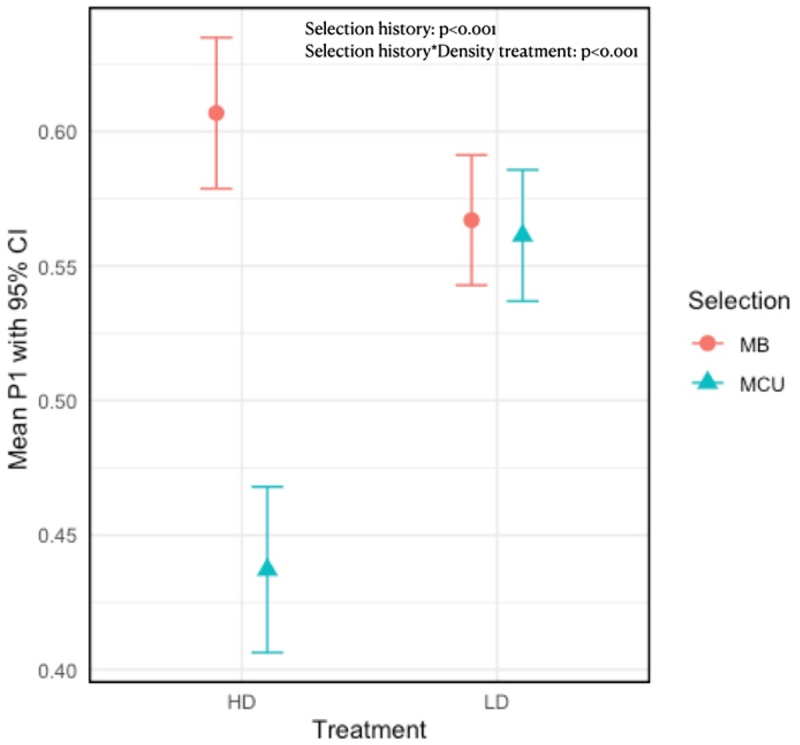
Effect of selection and treatment interaction on sperm defense ability of 4-day-old males. The error bars represent the 95% confidence interval. The presented data are arc-sine square-root transformed.

In examining the sperm defence ability, we found that in crowded larval conditions, males from crowding-adapted populations exhibited lower sperm defence ability than those from the control populations, across all the replicate blocks (Supplementary Figure 3). Conversely, in an uncrowded larval environment, males from both populations demonstrated comparable sperm defence abilities (Table 1, Figure 1). There was a significant impact of selection history, and the interaction between selection and density treatment had a significant effect on the sperm defence ability of 4-day-old males.

**Table 1.** Effects of selection regime and larval crowding treatment on sperm defense (P1) of males 4 days post-eclosion. The table presents F-statistic (F), degrees of freedom (df), residual degrees of freedom (Df.Res), and the probability value (Pr(>f)) for each term in the model. Significant effects are marked with an asterisk (*), indicating a p-value <0.05.

	F	Df	Df.res	Pr (>F)
(Intercept)	174.786	1	5.41	<0.001*
Selection	19.849	1	751.51	<0.001*
Treatment	0.625	1	752.45	0.430
Selection X Treatment	10.240	1	751.36	<0.001*

Similar to the results of our LMM, the results of the GLMM indicated that males from selected populations had poorer sperm defence ability, particularly under crowded larval conditions. There was a significant effect of selection and a significant interaction between selection and treatment. Additionally, there was a significant effect of density treatment in the GLMM model, with males from the high-density treatment performing better than those from the low-density treatment. This effect was primarily driven by control males from high larval density treatment being better in sperm defence ability than other treatments (Supplementary Table 8). The total number of red-eyed females progeny laid in this assay followed a similar trend as sperm defence assay with selection having a significant effect with control males laying more number of red-eyed female progeny (Supplementary Table 9) (Supplementary Figure 4).

### Sperm offence (P2) assay

**Figure 2.**
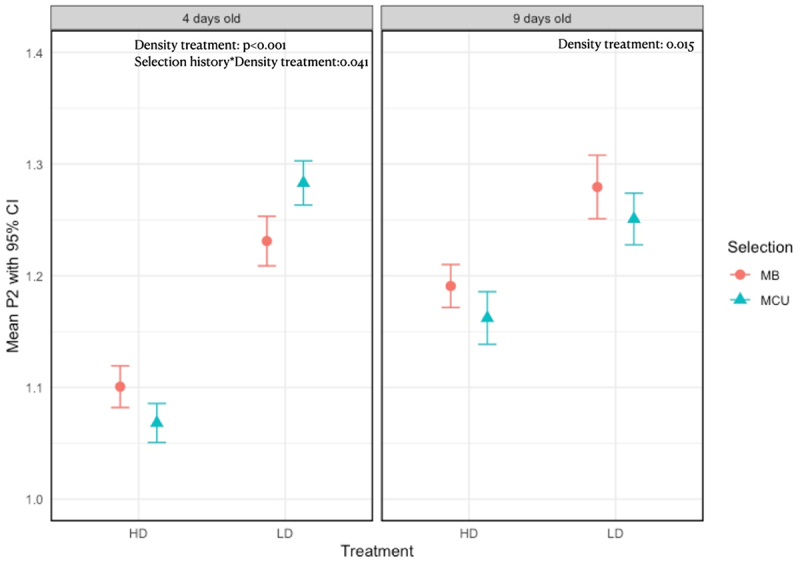
Effect of selection and treatment interaction on sperm offense ability of: A- 4-day-old males, B- 9-day-old males. The error bars represent the 95% confidence interval. The presented data are arc-sine square-root transformed.

Males of both crowding-adapted and control populations had better sperm offence ability when grown in an uncrowded environment as compared to when grown in a crowded environment. However, within the same density treatment, the sperm defence ability was comparable between males from both crowding-adapted and control populations. Further, 4-day-old males from crowding-adapted populations, when reared in low larval density conditions, exhibited higher mean sperm offence ability compared to males from both crowding-adapted and control populations grown in high larval density (Supplementary Table 10 and 11), but this difference was not statistically significant, leading to a significant interaction between selection history and density treatment regarding the sperm offence ability of 4-day-old males, while no such interaction was observed for 9-day-old males (Table 2, Figure 2).

**Table 2.** Effects of selection regime and larval crowding treatment on sperm offense (P2) of males: A – 4-day-old males, B – 9-day-old males. The table presents F-statistic (F), degrees of freedom (df), residual degrees of freedom (Df.Res), and the probability value (Pr(>f)) for each term in the model. Significant effects are marked with an asterisk (*), indicating a p-value <0.05.

4-day-old males	F	Df	Df.res	Pr (>F)
(Intercept)	604.384	1	3.76	<0.001*
Selection	1.687	1	1208.1	0.194
Treatment	22.946	1	1208.32	<0.001*
Selection X Treatment	4.190	1	1208.18	0.041*
**9-day-old males**				
(Intercept)	2184.647	1	8.29	<0.001*
Selection	0.949	1	725.9	0.330
Treatment	5.922	1	725.76	0.015*
Selection X Treatment	0.002	1	725.19	0.966

The results were same for GLMM and LMM for 4-day-old males, i.e. males grown in low larval density had better sperm offence ability than the males grown in high larval density. There was a significant effect of density treatment and selection cross treatment interaction as observed with LMM (Supplementary Table 12). For 9-day-old males we found from GLMM that, similar to LMM there was a significant effect of density treatment but we found an additional significant effect of selection treatment with males from crowding-adapted populations being worse than control males in sperm offence ability (Supplementary Table 13). Total female progeny of red-eye-colour produced by males when the assay was done on 4-day-old males showed a significant effect of density treatment (Supplementary Table 14) (Supplementary Figure 5). For 9-day-old males, the effects of treatment and selection were similar in trend to LMM and GLMM sperm defence (Supplementary Table 15) (Supplementary Figure 6), with males from control populations giving more red-eyed progenies than the males of crowding-adapted populations when grown in high larval density.

While the overall results were consistent across blocks, some block-specific differences were observed when each block was analysed separately. For instance, in the sperm offence assay for 4-day-old males, block 3 exhibited a higher mean for the crowding-adapted population compared to other treatments and blocks (Supplementary Figure 7). Additionally, for P2 in 9-day-old males, block 1 control males reared in low larval density had a higher mean than those in other treatments and blocks (Supplementary Figure 8). In our study, blocks serve as independent replicates of experiments and represents ancestry, demonstrating the consistency of the evolutionary response. Given that all blocks were maintained independently for over 165 generations by the time of this experiment, it is possible that different correlated traits evolved in different blocks, potentially influencing sperm competitive abilities. Minor differences between blocks are fairly common in long-term experimental evolution studies [39,57]). Despite these minor variations, our overall results, combined across all blocks, remain consistent, and the main conclusions of our study are unchanged.

## Discussion

We tested how a single generation of larval crowding versus long-term adaptation to a crowded environment over 165 generations affects the evolution of sperm competitive ability in *D. melanogaster*. Previous studies have shown that adaptation to crowded larval environments can lead to correlated evolution in adult reproductive traits, such as increased courtship activity. We hypothesized that resource limitations during development would create a trade-off between pre- and post-copulatory traits, resulting in lower sperm competitive ability in populations adapted to crowded larval conditions. Using experimentally evolved populations, we tested this hypothesis and found that, when reared in a crowded developmental environment, males from populations adapted to crowding exhibited decreased sperm defence ability.

In *Drosophila*, it is known that larval crowding and adaptation to a crowded larval environment directly influence growth rates and adult body size [58], and studies have demonstrated that the testis and accessory gland size of males are also affected by these factors [59]. It is possible that 4 days post eclosion, the testis and accessory glands of crowding-adapted males may not be fully matured, leading to the reduction in sperm defence ability. However, we note that there is no difference in the sperm offence ability of 4-day-old control and crowding-adapted males. Thus, it is unlikely that immature reproductive organs are the cause of poor sperm defence ability in 4-day-old crowding-adapted males exposed to crowded larval environments. Furthermore, the results from a previous study on the same populations indicate that the absolute size of both testes and accessory glands, 4 days post eclosion is similar in males from both selected and control populations when grown under crowded larval conditions [40]. Therefore, the possibility of immature reproductive tissues in 4-day-old crowding-adapted males can be reasonably discounted.

In *D. melanogaster*, paternity bias favours the last male that mates with the female [60]. Moreover, in these populations, the eggs laid by the females on day 20–21 of their regular maintenance cycle are selected for next generation. Considering the last male’s sperm precedence and late-life fecundity selection happening in these populations, increased investment of sperm and accessory gland proteins in a virgin female is unlikely to fetch a fitness advantage for the males. Moreover, since males of crowding-adapted populations every generation face a resource-limited larval crowding environment, they could suffer a severe negative fitness consequence via trade-offs if they invest too many resources in mating with virgin females. Therefore, it is possible that these males might have evolved condition-dependent sperm defence strategies to decrease the investment in their ejaculate when they mate with a virgin female. However, when grown in a low larval density environment where the resources are not limited, their sperm defence ability is as good as the males of the control populations. Further, consistent with the findings of McGraw et al., [61], we did not find any significant difference in sperm defence ability of males from high larval density and low larval density treatments in control populations. Although in their study the high larval density was not as extreme as ours, our result is still consistent with their finding that manipulation of the larval densities for one generation does not affect the sperm defence ability.

Another possible explanation for our results lies in the difference of perception between sperm competition intensity and sperm competition risk. Larval crowding could act as an anticipatory cue for increased adult densities and higher levels of sperm competition. Theoretical studies suggest that males facing a high risk of sperm competition (likelihood of encountering rivals) should increase investment in their ejaculate, while those facing a high intensity of sperm competition (competition with multiple males) may decrease ejaculate investment due to diminishing returns [9–11].

A single generation of larval crowding might signal an increased risk of sperm competition, prompting males to boost ejaculate investment, as observed in our results for P1 of control populations males grown under high larval density. In contrast, populations adapted to crowded environments over many generations may perceive high densities as an indicator of sperm competition intensity rather than just risk. This perception combined with last male sperm precedence phenomenon could lead to reduced sperm defence ability and a shift in resource allocation towards pre-copulatory traits, such as increased courtship behaviour [42], which has been observed in crowding-adapted populations in this study.

In our analysis of sperm offence in 9-day-old males, we observed a significant effect of selection history using the GLMM (Supplementary Table 12), whereas no such effect was detected with the LMM. This suggests that selection history may have subtle yet impactful influences on sperm offence ability in older males, potentially due to age-related changes that the LMM was less equipped to identify. While these males may be adapted for a 9-day reproductive peak, the constant exposure to resource limitations during larval development could have long-term consequences on their overall resource reserves and reproductive capabilities. By the time these males reach their late-age reproductive peak, they might experience a slight decline in reproductive success due to the cumulative strain of developing under resource-limited conditions. This may manifest as a reduced ability to sustain high-quality ejaculate investment or effective sperm competition traits when they reach 9 days old (Supplementary Table 13).

We found that sperm offence ability of the males is negatively affected by the larval density for both crowding-adapted and control populations. From a previous study using the same populations, we know that while absolute sizes of testis and accessory glands are affected similarly by larval crowding, when normalized with the body size only the relative size of accessory glands is affected but not testis [40]. Together, these two results suggest a possible correlation between sperm offence ability of males and the relative size of their accessory glands. The role of accessory gland proteins (ACPs) in mediating sperm displacement (and hence sperm offence) has been identified by previous studies [62–64]. It is possible that the observed negative effect of larval density on sperm offence ability of males in this study could be due to lower quality and/or quantity of ACPs of high-density males as compared to low density males.

The analysis of total red-eyed progeny provides additional context to the sperm defence assay, as it reflects the absolute reproductive success of focal males. While this measure aligns with the trends observed in the proportion-based metrics, it is important to note that total progeny counts may also be influenced by external factors, such as female fecundity or differences in mating success. As a result, proportion-based metrics remain the primary and more reliable measure of sperm defence ability. Nevertheless, the observed consistency between total progeny counts and P1 values reinforces the robustness of our findings and highlights the role of developmental and evolutionary history in shaping male reproductive performance.

Environmental conditions like increased density and decreased nutrition negatively influences the lengths of both the seminal receptacle (SR) and sperm [34]. Long-term experimental evolution research has further revealed a link where the length of a female’s SR can drive the evolution of longer sperm in males. However, this advantage appears to be weak or even non-existent in females with shorter SR [33,65]. In the context of our experiment, it is conceivable that adaptation to a crowded larval environment for more than 165 generations could have led to directional selection for shorter SR length in females. This, in turn, might have contributed to the evolution of shorter sperm length in males, resulting in a potential decrease in sperm competitive ability. Previous findings have indicated a trade-off between sperm length and sperm number. Moreover, the evolution of longer sperm has been associated with high life-history costs [66,67]. In the sperm offence assay, it is plausible that males from populations selected for adaptation to crowded larval environments may have compensated for their smaller sperm size by increasing the size of their ejaculate. Testing these hypotheses within this system would be of considerable interest.

In conclusion, the results of this study show that contrary to single generation of larval crowding, evolution under crowded larval environments could lead to the correlated evolution of lower sperm defence ability in males grown in crowded larval environment. In contrast, sperm offence ability is not influenced by evolutionary history in 4-day-old males but is slightly reduced in 9-day-old males adapted to crowded larval environments.

## Supplementary Material

Supplementary material.docx

## Data Availability

All the raw data is attached in the supplementary file submitted along with the manuscript labelled.
