# Supplementary material for: Effects of adaptation to crowded larval environment on the evolution of sperm competitive ability in males of Drosophila melanogaster
Source: Fly (Austin). 2024 Dec 18;19(1):2437204. doi: 10.1080/19336934.2024.2437204 (PMC11660399; doi:10.1080/19336934.2024.2437204)
Supplement: Supplementary material.docx [file KFLY_A_2437204_SM1699.docx]

Preparation of fly media (Charcoal-Cornmeal food):

For preparing 1L of cornmeal-charcoal food, the following ingredients are mixed in a pressure

cooker: 12g of agar, 40g of dry yeast, 40g of refined sugar, 100g of cornmeal, 0.5g of charcoal

are mixed with 1100ml of water. The mixture is then mixed with a stirrer to attain consistency.

After achieving a consistent mixture, the lid of the pressure cooker is closed for about 15

minutes until it whistles once. The vessel is then taken off the stove and allowed to cool down

to 60°C. Methylparaben and propionic acid (preservatives) are added to final concentrations

of 0.1% (w/v) and 1% (v/v) respectively when the food is at 60°C. Preservatives are adequately

mixed by continuous stirring for about 2 minutes. The food is then poured into the vials/plates

and allowed to cool down and solidify. Once dried, the food is ready to use.

Preparation of fly media (Banana-Jaggery food):

To prepare 1L of Banana-Jaggery food, the following ingredients are added in 1800 ml of food- Banana 205g, Barley flour 25g, Jaggery 35g, Yeast 36g , agar 12.4g and the mixture is boiled. After the boil is achieved, the mixture is allowed to cool until the media gets down to 60°C after which 2.4g of p-Hydroxymethyl Benzoate is added to 45ml of ethanol and this mixture is added to the media.


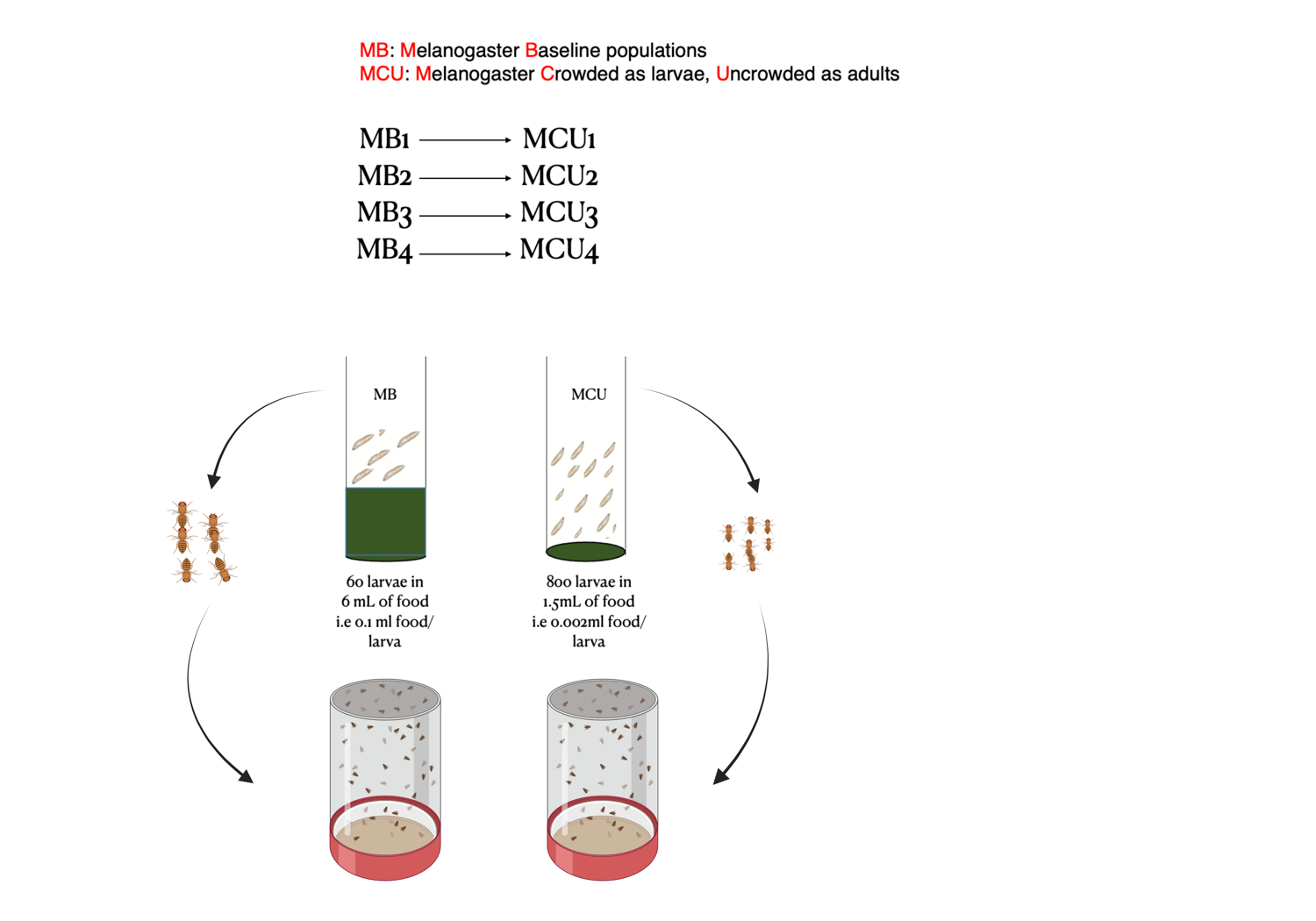


Eggs

Supplementary figure 1: Simplified sketch of population maintenance scheme that we followed for more than 165 generations of adaptation


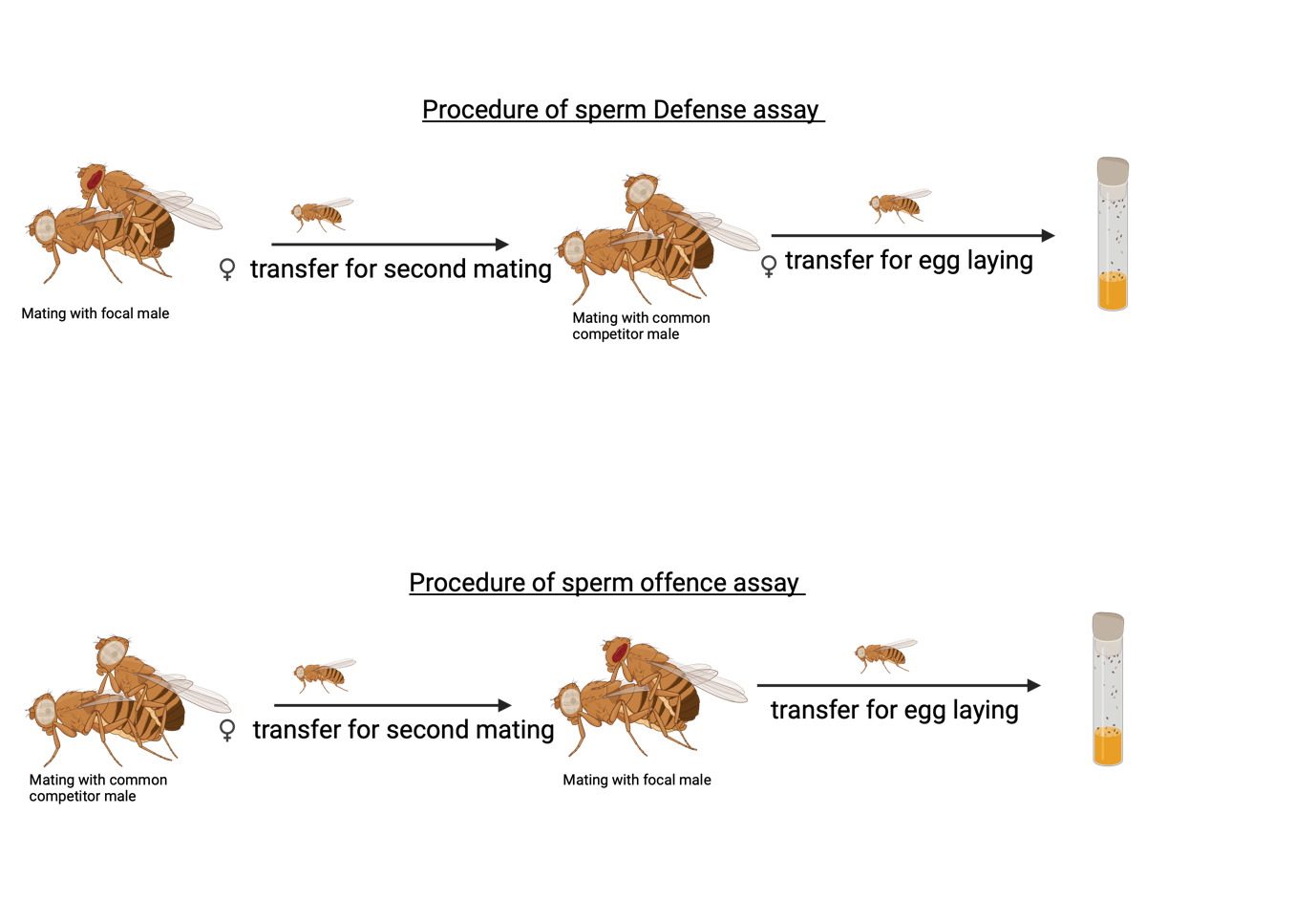


Supplementary figure 2: Simplified scheme of sperm Defense and sperm offense assay

Statistical analysis and results

In addition to the analysis mentioned in the main manuscript we also conducted a logistic regression for the sperm defense assay. In this analysis, each instance where the focal male failed to sire any progeny was coded as ‘success = 0’, while instances where the focal male sired at least one progeny were coded as ‘success = 1’. The model used was:

model <- glm(success ~ Selection * Treatment, data = a, family = binomial).

Results

Sperm defense (P1) assay

We analysed each block separately to determine if in any block males from the crowding-adapted population reared in a crowded environment exhibited better sperm defense ability than males from the control population. No such instances were observed, and results were generally consistent across all four blocks (Supplementary Figure 3). The variance components for the random effect (Block) and residuals were estimated and were as follows: Random effect (Block) variance: 0.0055; Residual variance 0.1288; Total variance: 0.1343; Proportion of variance explained by random effect (Block): 0.041 (or 4.1%) (Supplementary figure 3).

The results of logistic regression were similar to what we observed in LLM model sperm defense.. There was a significant effect of Selection history(p=0.001), with crowding adapted males having lower odds of success than control males. Additionally, similar to the sperm defense assay, there was a significant Selection × Treatment interaction (p=0.02), indicating that males from crowding-adapted populations had higher odds of success under the LD treatment (Supplementary Table 7).

Sperm offense (P2) assay

We analysed each block separately to determine block specific effects. The variance components for the random effect (Block) and residuals were estimated and were as follows for 4-day-old males: Random effect (Block) variance: 0.0067; Residual variance: 0.1104; Total variance: 0.1171; Proportion of variance explained by the random effect (Block): 5.75% and for 9-day-old males were as follows: Random effect (Block) variance: 0.001; Residual variance: 0.971; Total variance: 0.0981; Proportion of variance explained by the random effect (Block): 1.01%.


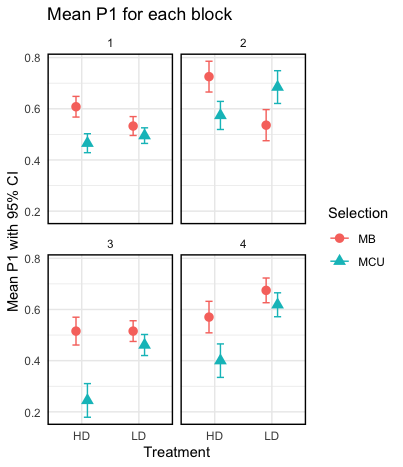


Supplementary figure 3: Effect of selection and treatment interaction on sperm Defense ability of 4 day old males for each block. The error bars represent the 95% confidence interval. The presented data are arc-sine square-root transformed.


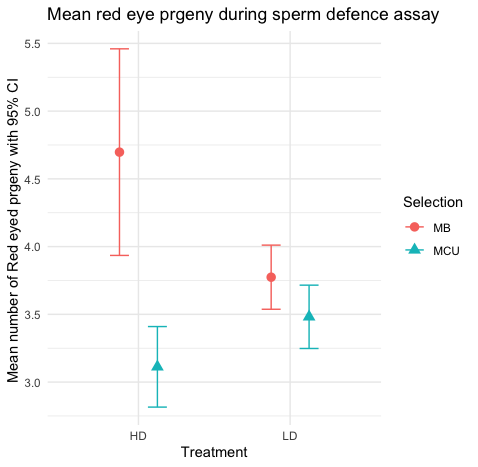


Supplementary Figure 4: Effect of selection and treatment interaction on red eye progeny production of 4 day old males sperm Defense assay. The error bars represent the 95% confidence interval.


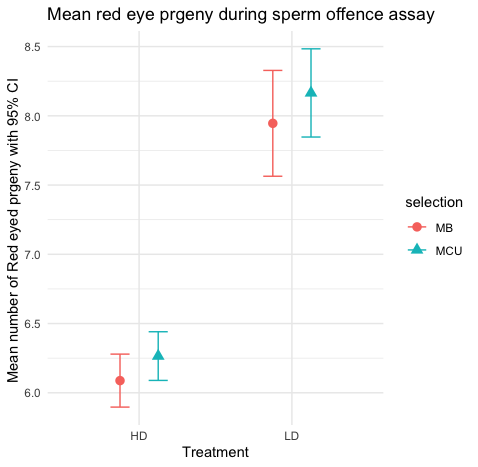


Supplementary Figure 5: Effect of selection and treatment interaction on red eye progeny production of 4 day old males during sperm offense assay. The error bars represent the 95% confidence interval.


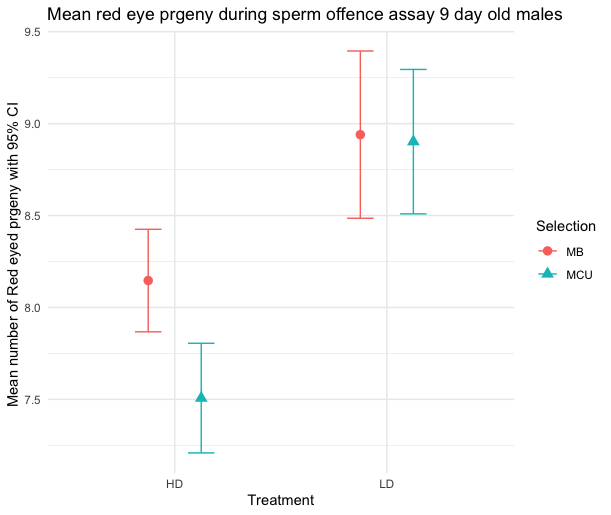


Supplementary Figure 6: Effect of selection and treatment interaction on red eye progeny production of 9 day old males during sperm offense assay. The error bars represent the 95% confidence interval.


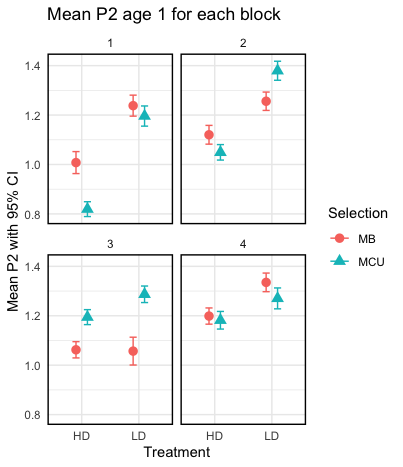


Supplementary figure 7: Effect of selection and treatment interaction on sperm offense ability of 4 day old males for each block. The error bars represent the 95% confidence interval. The presented data are arc-sine square-root transformed.


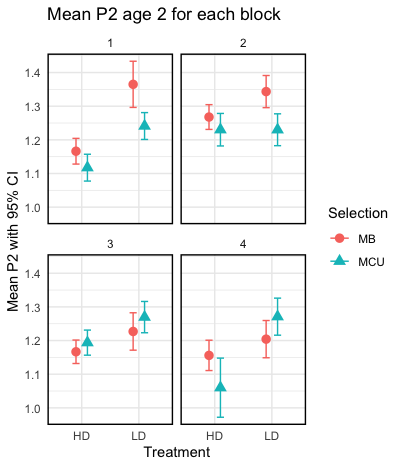


Supplementary figure 8: Effect of selection and treatment interaction on sperm offense ability of 9 day old males for each block . The error bars represent the 95% confidence interval. The presented data are arc-sine square-root transformed.

Supplementary table 1: 4 day old male P1= Untransformed raw data values of P1; Selection: MCU= Populations adapted to larval crowding, MB: Control populations; Treatment= HD= High larval density, LD= Low larval density; Block= Replicate populations of MCU and MB; P1 (arcsin sqrt) Arcsin square root transformed values P1 values

| P1 values | Selection | Treatment | Block | P1 (arcsin sqrt) |
| --- | --- | --- | --- | --- |
| 0.333333 | MCU | HD | 1 | 0.61548 |
| 0.2 | MCU | HD | 1 | 0.463648 |
| 0.0625 | MCU | HD | 1 | 0.25268 |
| 0.090909 | MCU | HD | 1 | 0.306277 |
| 0.35 | MCU | HD | 1 | 0.633052 |
| 0.428571 | MCU | HD | 1 | 0.713724 |
| 0.2 | MCU | HD | 1 | 0.463648 |
| 0.142857 | MCU | HD | 1 | 0.387597 |
| 0.076923 | MCU | HD | 1 | 0.281035 |
| 0.5625 | MCU | HD | 1 | 0.848062 |
| 0.153846 | MCU | HD | 1 | 0.403057 |
| 0.25 | MCU | HD | 1 | 0.523599 |
| 0.0625 | MCU | HD | 1 | 0.25268 |
| 0.291667 | MCU | HD | 1 | 0.57051 |
| 0.2 | MCU | HD | 1 | 0.463648 |
| 0.5 | MCU | HD | 1 | 0.785398 |
| 0.1 | MCU | HD | 1 | 0.321751 |
| 0.142857 | MCU | HD | 1 | 0.387597 |
| 0.714286 | MCU | HD | 1 | 1.006854 |
| 0.1 | MCU | HD | 1 | 0.321751 |
| 0.055556 | MCU | HD | 1 | 0.237941 |
| 0.066667 | MCU | HD | 1 | 0.261157 |
| 0.142857 | MCU | HD | 1 | 0.387597 |
| 0.454545 | MCU | HD | 1 | 0.739881 |
| 0.125 | MCU | HD | 1 | 0.361367 |
| 0.125 | MCU | HD | 1 | 0.361367 |
| 0.1 | MCU | HD | 1 | 0.321751 |
| 0.4 | MCU | HD | 1 | 0.684719 |
| 0.083333 | MCU | HD | 1 | 0.292843 |
| 0.095238 | MCU | HD | 1 | 0.313728 |
| 0.105263 | MB | HD | 1 | 0.330423 |
| 0.166667 | MB | HD | 1 | 0.420534 |
| 0.461538 | MB | HD | 1 | 0.746899 |
| 0.0625 | MB | HD | 1 | 0.25268 |
| 0.083333 | MB | HD | 1 | 0.292843 |
| 0.111111 | MB | HD | 1 | 0.339837 |
| 0.363636 | MB | HD | 1 | 0.647285 |
| 0.333333 | MB | HD | 1 | 0.61548 |
| 0.2 | MB | HD | 1 | 0.463648 |
| 0.764706 | MB | HD | 1 | 1.064352 |
| 0.076923 | MB | HD | 1 | 0.281035 |
| 0.32 | MB | HD | 1 | 0.601264 |
| 0.714286 | MB | HD | 1 | 1.006854 |
| 0.125 | MB | HD | 1 | 0.361367 |
| 0.5 | MB | HD | 1 | 0.785398 |
| 0.222222 | MB | HD | 1 | 0.490883 |
| 0.142857 | MB | HD | 1 | 0.387597 |
| 0.166667 | MB | HD | 1 | 0.420534 |
| 0.785714 | MB | HD | 1 | 1.089521 |
| 0.055556 | MB | HD | 1 | 0.237941 |
| 0.714286 | MB | HD | 1 | 1.006854 |
| 0.333333 | MB | HD | 1 | 0.61548 |
| 0.16 | MB | HD | 1 | 0.411517 |
| 0.586207 | MB | HD | 1 | 0.872038 |
| 0.090909 | MB | HD | 1 | 0.306277 |
| 0.384615 | MB | HD | 1 | 0.668964 |
| 0.346154 | MB | HD | 1 | 0.629015 |
| 0.647059 | MB | HD | 1 | 0.934664 |
| 0.5 | MB | HD | 1 | 0.785398 |
| 0.6 | MB | HD | 1 | 0.886077 |
| 0.142857 | MB | HD | 1 | 0.387597 |
| 0.045455 | MB | HD | 1 | 0.21485 |
| 0.666667 | MB | HD | 1 | 0.955317 |
| 0.2 | MB | HD | 1 | 0.463648 |
| 0.8 | MB | HD | 1 | 1.107149 |
| 0.235294 | MB | HD | 1 | 0.506445 |
| 0.230769 | MB | HD | 1 | 0.501093 |
| 0.235294 | MB | HD | 1 | 0.506445 |
| 0.142857 | MB | HD | 1 | 0.387597 |
| 0.875 | MB | HD | 1 | 1.209429 |
| 0.105263 | MB | HD | 1 | 0.330423 |
| 0.363636 | MB | HD | 1 | 0.647285 |
| 0.5 | MB | HD | 1 | 0.785398 |
| 0.625 | MB | HD | 1 | 0.911738 |
| 0.125 | MB | HD | 1 | 0.361367 |
| 0.454545 | MB | HD | 1 | 0.739881 |
| 0.125 | MB | LD | 1 | 0.361367 |
| 0.055556 | MB | LD | 1 | 0.237941 |
| 0.230769 | MB | LD | 1 | 0.501093 |
| 0.590909 | MB | LD | 1 | 0.876816 |
| 0.5 | MB | LD | 1 | 0.785398 |
| 0.333333 | MB | LD | 1 | 0.61548 |
| 0.4 | MB | LD | 1 | 0.684719 |
| 0.25 | MB | LD | 1 | 0.523599 |
| 0.222222 | MB | LD | 1 | 0.490883 |
| 0.153846 | MB | LD | 1 | 0.403057 |
| 0.214286 | MB | LD | 1 | 0.481275 |
| 0.1 | MB | LD | 1 | 0.321751 |
| 0.333333 | MB | LD | 1 | 0.61548 |
| 0.111111 | MB | LD | 1 | 0.339837 |
| 0.25 | MB | LD | 1 | 0.523599 |
| 0.235294 | MB | LD | 1 | 0.506445 |
| 0.25 | MB | LD | 1 | 0.523599 |
| 0.8 | MB | LD | 1 | 1.107149 |
| 0.030303 | MB | LD | 1 | 0.174969 |
| 0.25 | MB | LD | 1 | 0.523599 |
| 0.37037 | MB | LD | 1 | 0.654271 |
| 0.125 | MB | LD | 1 | 0.361367 |
| 0.363636 | MB | LD | 1 | 0.647285 |
| 0.111111 | MB | LD | 1 | 0.339837 |
| 0.096774 | MB | LD | 1 | 0.316335 |
| 0.388889 | MB | LD | 1 | 0.673352 |
| 0.2 | MB | LD | 1 | 0.463648 |
| 0.391304 | MB | LD | 1 | 0.675828 |
| 0.8 | MB | LD | 1 | 1.107149 |
| 0.071429 | MB | LD | 1 | 0.27055 |
| 0.304348 | MB | LD | 1 | 0.584374 |
| 0.047619 | MB | LD | 1 | 0.219988 |
| 0.076923 | MB | LD | 1 | 0.281035 |
| 0.217391 | MB | LD | 1 | 0.48505 |
| 0.153846 | MB | LD | 1 | 0.403057 |
| 0.2 | MB | LD | 1 | 0.463648 |
| 0.4 | MB | LD | 1 | 0.684719 |
| 0.714286 | MB | LD | 1 | 1.006854 |
| 0.1 | MCU | LD | 1 | 0.321751 |
| 0.277778 | MCU | LD | 1 | 0.555121 |
| 0.916667 | MCU | LD | 1 | 1.277954 |
| 0.166667 | MCU | LD | 1 | 0.420534 |
| 0.083333 | MCU | LD | 1 | 0.292843 |
| 0.318182 | MCU | LD | 1 | 0.599314 |
| 0.2 | MCU | LD | 1 | 0.463648 |
| 0.516129 | MCU | LD | 1 | 0.80153 |
| 0.368421 | MCU | LD | 1 | 0.652251 |
| 0.222222 | MCU | LD | 1 | 0.490883 |
| 0.5 | MCU | LD | 1 | 0.785398 |
| 0.294118 | MCU | LD | 1 | 0.573203 |
| 0.136364 | MCU | LD | 1 | 0.378228 |
| 0.181818 | MCU | LD | 1 | 0.440511 |
| 0.058824 | MCU | LD | 1 | 0.244979 |
| 0.388889 | MCU | LD | 1 | 0.673352 |
| 0.363636 | MCU | LD | 1 | 0.647285 |
| 0.272727 | MCU | LD | 1 | 0.549467 |
| 0.181818 | MCU | LD | 1 | 0.440511 |
| 0.066667 | MCU | LD | 1 | 0.261157 |
| 0.1875 | MCU | LD | 1 | 0.447832 |
| 0.636364 | MCU | LD | 1 | 0.923511 |
| 0.214286 | MCU | LD | 1 | 0.481275 |
| 0.285714 | MCU | LD | 1 | 0.563943 |
| 0.25 | MCU | LD | 1 | 0.523599 |
| 0.1875 | MCU | LD | 1 | 0.447832 |
| 0.153846 | MCU | LD | 1 | 0.403057 |
| 0.181818 | MCU | LD | 1 | 0.440511 |
| 0.166667 | MCU | LD | 1 | 0.420534 |
| 0.421053 | MCU | LD | 1 | 0.706119 |
| 0.142857 | MCU | LD | 1 | 0.387597 |
| 0.222222 | MCU | LD | 1 | 0.490883 |
| 0.058824 | MCU | LD | 1 | 0.244979 |
| 0.333333 | MCU | LD | 1 | 0.61548 |
| 0.066667 | MCU | LD | 1 | 0.261157 |
| 0.190476 | MCU | LD | 1 | 0.451633 |
| 0.125 | MCU | LD | 1 | 0.361367 |
| 0.222222 | MCU | LD | 1 | 0.490883 |
| 0.045455 | MCU | LD | 1 | 0.21485 |
| 0.166667 | MCU | LD | 1 | 0.420534 |
| 0.0625 | MCU | LD | 1 | 0.25268 |
| 0.166667 | MCU | LD | 1 | 0.420534 |
| 0.1875 | MCU | LD | 1 | 0.447832 |
| 0 | MCU | HD | 2 | 0 |
| 0 | MCU | HD | 2 | 0 |
| 0 | MCU | HD | 2 | 0 |
| 0 | MCU | HD | 2 | 0 |
| 0 | MCU | HD | 2 | 0 |
| 0 | MCU | HD | 2 | 0 |
| 0 | MCU | HD | 2 | 0 |
| 0 | MCU | HD | 2 | 0 |
| 0 | MCU | HD | 2 | 0 |
| 0 | MB | HD | 2 | 0 |
| 0 | MB | HD | 2 | 0 |
| 0 | MB | HD | 2 | 0 |
| 0 | MB | HD | 2 | 0 |
| 0 | MB | HD | 2 | 0 |
| 0 | MB | HD | 2 | 0 |
| 0 | MB | HD | 2 | 0 |
| 0 | MCU | LD | 2 | 0 |
| 0 | MCU | LD | 2 | 0 |
| 0 | MCU | LD | 2 | 0 |
| 0 | MCU | LD | 2 | 0 |
| 0 | MCU | LD | 2 | 0 |
| 0 | MCU | LD | 2 | 0 |
| 0 | MCU | LD | 2 | 0 |
| 0 | MB | LD | 2 | 0 |
| 0 | MB | LD | 2 | 0 |
| 0 | MB | LD | 2 | 0 |
| 0 | MB | LD | 2 | 0 |
| 0 | MB | LD | 2 | 0 |
| 0 | MB | LD | 2 | 0 |
| 0 | MB | LD | 2 | 0 |
| 0 | MB | LD | 2 | 0 |
| 0 | MB | LD | 2 | 0 |
| 0 | MB | LD | 2 | 0 |
| 0 | MB | LD | 2 | 0 |
| 0.1 | MCU | HD | 2 | 0.321751 |
| 0.1 | MCU | LD | 2 | 0.321751 |
| 0.111111 | MCU | LD | 2 | 0.339837 |
| 0.111111 | MB | LD | 2 | 0.339837 |
| 0.125 | MB | HD | 2 | 0.361367 |
| 0.125 | MB | LD | 2 | 0.361367 |
| 0.125 | MB | LD | 2 | 0.361367 |
| 0.142857 | MCU | LD | 2 | 0.387597 |
| 0.142857 | MCU | LD | 2 | 0.387597 |
| 0.142857 | MB | LD | 2 | 0.387597 |
| 0.166667 | MCU | HD | 2 | 0.420534 |
| 0.166667 | MB | HD | 2 | 0.420534 |
| 0.166667 | MCU | LD | 2 | 0.420534 |
| 0.166667 | MB | LD | 2 | 0.420534 |
| 0.1875 | MCU | HD | 2 | 0.447832 |
| 0.2 | MCU | HD | 2 | 0.463648 |
| 0.2 | MCU | HD | 2 | 0.463648 |
| 0.2 | MB | HD | 2 | 0.463648 |
| 0.2 | MB | HD | 2 | 0.463648 |
| 0.2 | MCU | LD | 2 | 0.463648 |
| 0.2 | MCU | LD | 2 | 0.463648 |
| 0.2 | MCU | LD | 2 | 0.463648 |
| 0.2 | MB | LD | 2 | 0.463648 |
| 0.214286 | MCU | HD | 2 | 0.481275 |
| 0.222222 | MB | HD | 2 | 0.490883 |
| 0.222222 | MB | LD | 2 | 0.490883 |
| 0.25 | MCU | HD | 2 | 0.523599 |
| 0.25 | MCU | HD | 2 | 0.523599 |
| 0.25 | MCU | HD | 2 | 0.523599 |
| 0.25 | MCU | HD | 2 | 0.523599 |
| 0.25 | MCU | HD | 2 | 0.523599 |
| 0.25 | MCU | HD | 2 | 0.523599 |
| 0.25 | MB | HD | 2 | 0.523599 |
| 0.25 | MCU | LD | 2 | 0.523599 |
| 0.25 | MCU | LD | 2 | 0.523599 |
| 0.25 | MCU | LD | 2 | 0.523599 |
| 0.25 | MCU | LD | 2 | 0.523599 |
| 0.25 | MB | LD | 2 | 0.523599 |
| 0.25 | MB | LD | 2 | 0.523599 |
| 0.25 | MB | LD | 2 | 0.523599 |
| 0.25 | MB | LD | 2 | 0.523599 |
| 0.25 | MB | LD | 2 | 0.523599 |
| 0.25 | MB | LD | 2 | 0.523599 |
| 0.25 | MB | LD | 2 | 0.523599 |
| 0.25 | MB | LD | 2 | 0.523599 |
| 0.266667 | MCU | HD | 2 | 0.542639 |
| 0.266667 | MB | LD | 2 | 0.542639 |
| 0.272727 | MCU | LD | 2 | 0.549467 |
| 0.285714 | MCU | HD | 2 | 0.563943 |
| 0.285714 | MCU | LD | 2 | 0.563943 |
| 0.3 | MCU | LD | 2 | 0.57964 |
| 0.333333 | MCU | HD | 2 | 0.61548 |
| 0.333333 | MCU | HD | 2 | 0.61548 |
| 0.333333 | MCU | HD | 2 | 0.61548 |
| 0.333333 | MCU | HD | 2 | 0.61548 |
| 0.333333 | MCU | HD | 2 | 0.61548 |
| 0.333333 | MB | HD | 2 | 0.61548 |
| 0.333333 | MCU | LD | 2 | 0.61548 |
| 0.333333 | MCU | LD | 2 | 0.61548 |
| 0.333333 | MCU | LD | 2 | 0.61548 |
| 0.333333 | MCU | LD | 2 | 0.61548 |
| 0.333333 | MB | LD | 2 | 0.61548 |
| 0.333333 | MB | LD | 2 | 0.61548 |
| 0.363636 | MCU | HD | 2 | 0.647285 |
| 0.375 | MB | LD | 2 | 0.659058 |
| 0.4 | MCU | HD | 2 | 0.684719 |
| 0.4 | MCU | HD | 2 | 0.684719 |
| 0.4 | MCU | HD | 2 | 0.684719 |
| 0.4 | MCU | HD | 2 | 0.684719 |
| 0.4 | MB | HD | 2 | 0.684719 |
| 0.4 | MB | HD | 2 | 0.684719 |
| 0.4 | MCU | LD | 2 | 0.684719 |
| 0.4 | MB | LD | 2 | 0.684719 |
| 0.4 | MB | LD | 2 | 0.684719 |
| 0.428571 | MB | HD | 2 | 0.713724 |
| 0.428571 | MCU | LD | 2 | 0.713724 |
| 0.428571 | MCU | LD | 2 | 0.713724 |
| 0.428571 | MB | LD | 2 | 0.713724 |
| 0.4375 | MCU | LD | 2 | 0.722734 |
| 0.444444 | MB | LD | 2 | 0.729728 |
| 0.444444 | MB | LD | 2 | 0.729728 |
| 0.454545 | MB | HD | 2 | 0.739881 |
| 0.461538 | MCU | HD | 2 | 0.746899 |
| 0.461538 | MB | HD | 2 | 0.746899 |
| 0.461538 | MCU | LD | 2 | 0.746899 |
| 0.5 | MCU | HD | 2 | 0.785398 |
| 0.5 | MCU | HD | 2 | 0.785398 |
| 0.5 | MB | HD | 2 | 0.785398 |
| 0.5 | MB | HD | 2 | 0.785398 |
| 0.5 | MB | HD | 2 | 0.785398 |
| 0.5 | MB | HD | 2 | 0.785398 |
| 0.5 | MB | HD | 2 | 0.785398 |
| 0.5 | MB | HD | 2 | 0.785398 |
| 0.5 | MB | HD | 2 | 0.785398 |
| 0.5 | MB | HD | 2 | 0.785398 |
| 0.5 | MB | HD | 2 | 0.785398 |
| 0.5 | MB | HD | 2 | 0.785398 |
| 0.5 | MCU | LD | 2 | 0.785398 |
| 0.5 | MCU | LD | 2 | 0.785398 |
| 0.5 | MCU | LD | 2 | 0.785398 |
| 0.5 | MB | LD | 2 | 0.785398 |
| 0.5 | MB | LD | 2 | 0.785398 |
| 0.5 | MB | LD | 2 | 0.785398 |
| 0.5 | MB | LD | 2 | 0.785398 |
| 0.555556 | MB | HD | 2 | 0.841069 |
| 0.571429 | MCU | HD | 2 | 0.857072 |
| 0.571429 | MCU | HD | 2 | 0.857072 |
| 0.571429 | MCU | LD | 2 | 0.857072 |
| 0.6 | MCU | LD | 2 | 0.886077 |
| 0.6 | MCU | LD | 2 | 0.886077 |
| 0.6 | MB | LD | 2 | 0.886077 |
| 0.6 | MB | LD | 2 | 0.886077 |
| 0.615385 | MB | HD | 2 | 0.901832 |
| 0.625 | MB | HD | 2 | 0.911738 |
| 0.625 | MB | HD | 2 | 0.911738 |
| 0.625 | MCU | LD | 2 | 0.911738 |
| 0.636364 | MB | HD | 2 | 0.923511 |
| 0.642857 | MCU | LD | 2 | 0.930274 |
| 0.666667 | MB | HD | 2 | 0.955317 |
| 0.666667 | MB | HD | 2 | 0.955317 |
| 0.666667 | MB | HD | 2 | 0.955317 |
| 0.666667 | MCU | LD | 2 | 0.955317 |
| 0.684211 | MCU | HD | 2 | 0.974053 |
| 0.7 | MCU | HD | 2 | 0.991157 |
| 0.7 | MB | HD | 2 | 0.991157 |
| 0.7 | MCU | LD | 2 | 0.991157 |
| 0.7 | MB | LD | 2 | 0.991157 |
| 0.714286 | MCU | HD | 2 | 1.006854 |
| 0.714286 | MB | HD | 2 | 1.006854 |
| 0.75 | MCU | HD | 2 | 1.047198 |
| 0.75 | MB | LD | 2 | 1.047198 |
| 0.769231 | MCU | HD | 2 | 1.069703 |
| 0.777778 | MB | HD | 2 | 1.079914 |
| 0.8 | MB | HD | 2 | 1.107149 |
| 0.8 | MCU | LD | 2 | 1.107149 |
| 0.818182 | MB | HD | 2 | 1.130286 |
| 0.833333 | MCU | HD | 2 | 1.150262 |
| 0.846154 | MCU | LD | 2 | 1.167739 |
| 0.888889 | MB | HD | 2 | 1.230959 |
| 0.9 | MCU | HD | 2 | 1.249046 |
| 1 | MCU | HD | 2 | 1.570796 |
| 1 | MB | HD | 2 | 1.570796 |
| 1 | MB | HD | 2 | 1.570796 |
| 1 | MB | HD | 2 | 1.570796 |
| 1 | MCU | LD | 2 | 1.570796 |
| 1 | MCU | LD | 2 | 1.570796 |
| 1 | MCU | LD | 2 | 1.570796 |
| 1 | MCU | LD | 2 | 1.570796 |
| 1 | MCU | LD | 2 | 1.570796 |
| 1 | MCU | LD | 2 | 1.570796 |
| 1 | MB | LD | 2 | 1.570796 |
| 1 | MB | LD | 2 | 1.570796 |
| 1 | MB | LD | 2 | 1.570796 |
| 0 | MCU | HD | 3 | 0 |
| 0 | MCU | HD | 3 | 0 |
| 0 | MCU | HD | 3 | 0 |
| 0 | MCU | HD | 3 | 0 |
| 0 | MCU | HD | 3 | 0 |
| 0 | MCU | HD | 3 | 0 |
| 0 | MCU | HD | 3 | 0 |
| 0 | MCU | HD | 3 | 0 |
| 0 | MCU | HD | 3 | 0 |
| 0 | MCU | HD | 3 | 0 |
| 0 | MCU | HD | 3 | 0 |
| 0 | MCU | HD | 3 | 0 |
| 0 | MCU | HD | 3 | 0 |
| 0 | MCU | HD | 3 | 0 |
| 0 | MCU | HD | 3 | 0 |
| 0 | MCU | HD | 3 | 0 |
| 0 | MCU | HD | 3 | 0 |
| 0 | MB | HD | 3 | 0 |
| 0 | MB | HD | 3 | 0 |
| 0 | MB | HD | 3 | 0 |
| 0 | MB | HD | 3 | 0 |
| 0 | MB | HD | 3 | 0 |
| 0 | MB | HD | 3 | 0 |
| 0 | MB | HD | 3 | 0 |
| 0 | MCU | LD | 3 | 0 |
| 0 | MCU | LD | 3 | 0 |
| 0 | MCU | LD | 3 | 0 |
| 0 | MCU | LD | 3 | 0 |
| 0 | MCU | LD | 3 | 0 |
| 0 | MCU | LD | 3 | 0 |
| 0 | MCU | LD | 3 | 0 |
| 0 | MCU | LD | 3 | 0 |
| 0 | MCU | LD | 3 | 0 |
| 0 | MCU | LD | 3 | 0 |
| 0 | MCU | LD | 3 | 0 |
| 0 | MCU | LD | 3 | 0 |
| 0 | MCU | LD | 3 | 0 |
| 0 | MB | LD | 3 | 0 |
| 0 | MB | LD | 3 | 0 |
| 0 | MB | LD | 3 | 0 |
| 0 | MB | LD | 3 | 0 |
| 0 | MB | LD | 3 | 0 |
| 0 | MB | LD | 3 | 0 |
| 0 | MB | LD | 3 | 0 |
| 0 | MB | LD | 3 | 0 |
| 0 | MB | LD | 3 | 0 |
| 0 | MB | LD | 3 | 0 |
| 0 | MB | LD | 3 | 0 |
| 0 | MB | LD | 3 | 0 |
| 0 | MB | LD | 3 | 0 |
| 0 | MB | LD | 3 | 0 |
| 0.047619 | MB | LD | 3 | 0.219988 |
| 0.05 | MB | LD | 3 | 0.225513 |
| 0.052632 | MB | HD | 3 | 0.231477 |
| 0.052632 | MCU | LD | 3 | 0.231477 |
| 0.052632 | MB | LD | 3 | 0.231477 |
| 0.055556 | MCU | HD | 3 | 0.237941 |
| 0.0625 | MCU | LD | 3 | 0.25268 |
| 0.066667 | MB | HD | 3 | 0.261157 |
| 0.066667 | MCU | LD | 3 | 0.261157 |
| 0.066667 | MCU | LD | 3 | 0.261157 |
| 0.076923 | MCU | HD | 3 | 0.281035 |
| 0.076923 | MB | LD | 3 | 0.281035 |
| 0.083333 | MCU | LD | 3 | 0.292843 |
| 0.083333 | MCU | LD | 3 | 0.292843 |
| 0.090909 | MCU | LD | 3 | 0.306277 |
| 0.1 | MCU | HD | 3 | 0.321751 |
| 0.1 | MB | HD | 3 | 0.321751 |
| 0.1 | MB | HD | 3 | 0.321751 |
| 0.1 | MCU | LD | 3 | 0.321751 |
| 0.1 | MCU | LD | 3 | 0.321751 |
| 0.1 | MCU | LD | 3 | 0.321751 |
| 0.105263 | MB | HD | 3 | 0.330423 |
| 0.105263 | MB | LD | 3 | 0.330423 |
| 0.111111 | MB | HD | 3 | 0.339837 |
| 0.111111 | MCU | LD | 3 | 0.339837 |
| 0.111111 | MCU | LD | 3 | 0.339837 |
| 0.111111 | MCU | LD | 3 | 0.339837 |
| 0.125 | MCU | HD | 3 | 0.361367 |
| 0.125 | MB | LD | 3 | 0.361367 |
| 0.125 | MB | LD | 3 | 0.361367 |
| 0.125 | MB | LD | 3 | 0.361367 |
| 0.130435 | MCU | LD | 3 | 0.369509 |
| 0.133333 | MB | HD | 3 | 0.373792 |
| 0.133333 | MCU | LD | 3 | 0.373792 |
| 0.133333 | MCU | LD | 3 | 0.373792 |
| 0.133333 | MCU | LD | 3 | 0.373792 |
| 0.142857 | MB | LD | 3 | 0.387597 |
| 0.142857 | MB | LD | 3 | 0.387597 |
| 0.153846 | MB | HD | 3 | 0.403057 |
| 0.157895 | MB | HD | 3 | 0.408638 |
| 0.157895 | MB | LD | 3 | 0.408638 |
| 0.166667 | MCU | LD | 3 | 0.420534 |
| 0.166667 | MCU | LD | 3 | 0.420534 |
| 0.166667 | MB | LD | 3 | 0.420534 |
| 0.176471 | MCU | HD | 3 | 0.433538 |
| 0.181818 | MB | HD | 3 | 0.440511 |
| 0.2 | MCU | LD | 3 | 0.463648 |
| 0.2 | MCU | LD | 3 | 0.463648 |
| 0.2 | MB | LD | 3 | 0.463648 |
| 0.2 | MB | LD | 3 | 0.463648 |
| 0.2 | MB | LD | 3 | 0.463648 |
| 0.2 | MB | LD | 3 | 0.463648 |
| 0.2 | MB | LD | 3 | 0.463648 |
| 0.214286 | MCU | LD | 3 | 0.481275 |
| 0.222222 | MCU | HD | 3 | 0.490883 |
| 0.222222 | MCU | LD | 3 | 0.490883 |
| 0.222222 | MB | LD | 3 | 0.490883 |
| 0.222222 | MB | LD | 3 | 0.490883 |
| 0.222222 | MB | LD | 3 | 0.490883 |
| 0.227273 | MCU | LD | 3 | 0.496932 |
| 0.230769 | MCU | LD | 3 | 0.501093 |
| 0.235294 | MB | LD | 3 | 0.506445 |
| 0.238095 | MCU | LD | 3 | 0.50974 |
| 0.25 | MB | HD | 3 | 0.523599 |
| 0.25 | MB | HD | 3 | 0.523599 |
| 0.25 | MB | HD | 3 | 0.523599 |
| 0.25 | MB | HD | 3 | 0.523599 |
| 0.25 | MCU | LD | 3 | 0.523599 |
| 0.25 | MCU | LD | 3 | 0.523599 |
| 0.25 | MB | LD | 3 | 0.523599 |
| 0.266667 | MB | HD | 3 | 0.542639 |
| 0.272727 | MCU | LD | 3 | 0.549467 |
| 0.272727 | MCU | LD | 3 | 0.549467 |
| 0.277778 | MB | LD | 3 | 0.555121 |
| 0.285714 | MB | HD | 3 | 0.563943 |
| 0.285714 | MB | HD | 3 | 0.563943 |
| 0.285714 | MCU | LD | 3 | 0.563943 |
| 0.285714 | MB | LD | 3 | 0.563943 |
| 0.285714 | MB | LD | 3 | 0.563943 |
| 0.3 | MB | HD | 3 | 0.57964 |
| 0.3 | MCU | LD | 3 | 0.57964 |
| 0.3 | MB | LD | 3 | 0.57964 |
| 0.307692 | MCU | LD | 3 | 0.588003 |
| 0.307692 | MB | LD | 3 | 0.588003 |
| 0.3125 | MB | HD | 3 | 0.5932 |
| 0.315789 | MCU | HD | 3 | 0.596743 |
| 0.333333 | MCU | HD | 3 | 0.61548 |
| 0.333333 | MCU | LD | 3 | 0.61548 |
| 0.333333 | MCU | LD | 3 | 0.61548 |
| 0.333333 | MCU | LD | 3 | 0.61548 |
| 0.333333 | MB | LD | 3 | 0.61548 |
| 0.333333 | MB | LD | 3 | 0.61548 |
| 0.357143 | MB | HD | 3 | 0.640522 |
| 0.357143 | MB | LD | 3 | 0.640522 |
| 0.375 | MB | HD | 3 | 0.659058 |
| 0.375 | MCU | LD | 3 | 0.659058 |
| 0.375 | MCU | LD | 3 | 0.659058 |
| 0.4 | MB | HD | 3 | 0.684719 |
| 0.4 | MB | LD | 3 | 0.684719 |
| 0.4 | MB | LD | 3 | 0.684719 |
| 0.409091 | MB | HD | 3 | 0.693981 |
| 0.409091 | MB | LD | 3 | 0.693981 |
| 0.416667 | MB | HD | 3 | 0.701674 |
| 0.4375 | MCU | LD | 3 | 0.722734 |
| 0.444444 | MCU | HD | 3 | 0.729728 |
| 0.444444 | MB | LD | 3 | 0.729728 |
| 0.444444 | MB | LD | 3 | 0.729728 |
| 0.454545 | MB | HD | 3 | 0.739881 |
| 0.454545 | MCU | LD | 3 | 0.739881 |
| 0.454545 | MB | LD | 3 | 0.739881 |
| 0.473684 | MB | LD | 3 | 0.75907 |
| 0.5 | MB | HD | 3 | 0.785398 |
| 0.5 | MCU | LD | 3 | 0.785398 |
| 0.5 | MB | LD | 3 | 0.785398 |
| 0.5 | MB | LD | 3 | 0.785398 |
| 0.5 | MB | LD | 3 | 0.785398 |
| 0.5 | MB | LD | 3 | 0.785398 |
| 0.529412 | MB | LD | 3 | 0.814827 |
| 0.545455 | MB | LD | 3 | 0.830916 |
| 0.571429 | MCU | HD | 3 | 0.857072 |
| 0.571429 | MB | HD | 3 | 0.857072 |
| 0.571429 | MB | LD | 3 | 0.857072 |
| 0.583333 | MCU | HD | 3 | 0.869122 |
| 0.583333 | MCU | LD | 3 | 0.869122 |
| 0.6 | MB | HD | 3 | 0.886077 |
| 0.611111 | MCU | LD | 3 | 0.897445 |
| 0.615385 | MB | LD | 3 | 0.901832 |
| 0.625 | MCU | LD | 3 | 0.911738 |
| 0.625 | MB | LD | 3 | 0.911738 |
| 0.625 | MB | LD | 3 | 0.911738 |
| 0.642857 | MB | LD | 3 | 0.930274 |
| 0.647059 | MCU | LD | 3 | 0.934664 |
| 0.666667 | MCU | LD | 3 | 0.955317 |
| 0.666667 | MCU | LD | 3 | 0.955317 |
| 0.666667 | MCU | LD | 3 | 0.955317 |
| 0.666667 | MB | LD | 3 | 0.955317 |
| 0.7 | MB | LD | 3 | 0.991157 |
| 0.705882 | MB | LD | 3 | 0.997593 |
| 0.714286 | MB | HD | 3 | 1.006854 |
| 0.736842 | MB | LD | 3 | 1.032133 |
| 0.75 | MB | LD | 3 | 1.047198 |
| 0.764706 | MB | HD | 3 | 1.064352 |
| 0.764706 | MCU | LD | 3 | 1.064352 |
| 0.769231 | MCU | LD | 3 | 1.069703 |
| 0.769231 | MB | LD | 3 | 1.069703 |
| 0.769231 | MB | LD | 3 | 1.069703 |
| 0.777778 | MB | LD | 3 | 1.079914 |
| 0.785714 | MB | HD | 3 | 1.089521 |
| 0.8 | MB | HD | 3 | 1.107149 |
| 0.875 | MCU | LD | 3 | 1.209429 |
| 0.894737 | MCU | LD | 3 | 1.240374 |
| 0.928571 | MCU | HD | 3 | 1.300247 |
| 0.944444 | MB | HD | 3 | 1.332855 |
| 0 | MCU | HD | 4 | 0 |
| 0 | MCU | HD | 4 | 0 |
| 0 | MCU | HD | 4 | 0 |
| 0 | MCU | HD | 4 | 0 |
| 0 | MCU | HD | 4 | 0 |
| 0 | MCU | HD | 4 | 0 |
| 0 | MCU | HD | 4 | 0 |
| 0 | MCU | HD | 4 | 0 |
| 0 | MCU | HD | 4 | 0 |
| 0 | MCU | HD | 4 | 0 |
| 0 | MCU | HD | 4 | 0 |
| 0 | MCU | HD | 4 | 0 |
| 0 | MCU | HD | 4 | 0 |
| 0 | MCU | HD | 4 | 0 |
| 0 | MCU | HD | 4 | 0 |
| 0 | MCU | HD | 4 | 0 |
| 0 | MCU | HD | 4 | 0 |
| 0 | MCU | HD | 4 | 0 |
| 0 | MB | HD | 4 | 0 |
| 0 | MB | HD | 4 | 0 |
| 0 | MB | HD | 4 | 0 |
| 0 | MB | HD | 4 | 0 |
| 0 | MB | HD | 4 | 0 |
| 0 | MB | HD | 4 | 0 |
| 0 | MB | HD | 4 | 0 |
| 0 | MB | HD | 4 | 0 |
| 0 | MB | HD | 4 | 0 |
| 0 | MB | HD | 4 | 0 |
| 0 | MB | HD | 4 | 0 |
| 0 | MB | HD | 4 | 0 |
| 0 | MCU | LD | 4 | 0 |
| 0 | MCU | LD | 4 | 0 |
| 0 | MCU | LD | 4 | 0 |
| 0 | MCU | LD | 4 | 0 |
| 0 | MCU | LD | 4 | 0 |
| 0 | MB | LD | 4 | 0 |
| 0 | MB | LD | 4 | 0 |
| 0 | MB | LD | 4 | 0 |
| 0 | MB | LD | 4 | 0 |
| 0 | MB | LD | 4 | 0 |
| 0 | MB | LD | 4 | 0 |
| 0 | MB | LD | 4 | 0 |
| 0.041667 | MCU | HD | 4 | 0.205569 |
| 0.047619 | MCU | LD | 4 | 0.219988 |
| 0.052632 | MB | HD | 4 | 0.231477 |
| 0.055556 | MCU | LD | 4 | 0.237941 |
| 0.071429 | MCU | LD | 4 | 0.27055 |
| 0.071429 | MCU | LD | 4 | 0.27055 |
| 0.090909 | MB | HD | 4 | 0.306277 |
| 0.1 | MB | HD | 4 | 0.321751 |
| 0.1 | MCU | LD | 4 | 0.321751 |
| 0.1 | MCU | LD | 4 | 0.321751 |
| 0.1 | MCU | LD | 4 | 0.321751 |
| 0.111111 | MCU | LD | 4 | 0.339837 |
| 0.111111 | MB | LD | 4 | 0.339837 |
| 0.117647 | MCU | HD | 4 | 0.350106 |
| 0.125 | MB | HD | 4 | 0.361367 |
| 0.125 | MCU | LD | 4 | 0.361367 |
| 0.142857 | MB | HD | 4 | 0.387597 |
| 0.142857 | MCU | LD | 4 | 0.387597 |
| 0.142857 | MB | LD | 4 | 0.387597 |
| 0.142857 | MB | LD | 4 | 0.387597 |
| 0.142857 | MB | LD | 4 | 0.387597 |
| 0.153846 | MCU | HD | 4 | 0.403057 |
| 0.153846 | MB | LD | 4 | 0.403057 |
| 0.166667 | MCU | HD | 4 | 0.420534 |
| 0.166667 | MCU | HD | 4 | 0.420534 |
| 0.166667 | MCU | HD | 4 | 0.420534 |
| 0.166667 | MCU | LD | 4 | 0.420534 |
| 0.166667 | MB | LD | 4 | 0.420534 |
| 0.166667 | MB | LD | 4 | 0.420534 |
| 0.166667 | MB | LD | 4 | 0.420534 |
| 0.181818 | MCU | LD | 4 | 0.440511 |
| 0.181818 | MB | LD | 4 | 0.440511 |
| 0.2 | MB | HD | 4 | 0.463648 |
| 0.2 | MB | HD | 4 | 0.463648 |
| 0.2 | MCU | LD | 4 | 0.463648 |
| 0.2 | MB | LD | 4 | 0.463648 |
| 0.2 | MB | LD | 4 | 0.463648 |
| 0.2 | MB | LD | 4 | 0.463648 |
| 0.214286 | MB | HD | 4 | 0.481275 |
| 0.214286 | MB | HD | 4 | 0.481275 |
| 0.214286 | MB | LD | 4 | 0.481275 |
| 0.222222 | MCU | LD | 4 | 0.490883 |
| 0.222222 | MCU | LD | 4 | 0.490883 |
| 0.230769 | MB | HD | 4 | 0.501093 |
| 0.25 | MCU | HD | 4 | 0.523599 |
| 0.25 | MB | HD | 4 | 0.523599 |
| 0.25 | MB | HD | 4 | 0.523599 |
| 0.25 | MB | HD | 4 | 0.523599 |
| 0.25 | MCU | LD | 4 | 0.523599 |
| 0.25 | MCU | LD | 4 | 0.523599 |
| 0.25 | MCU | LD | 4 | 0.523599 |
| 0.25 | MCU | LD | 4 | 0.523599 |
| 0.25 | MB | LD | 4 | 0.523599 |
| 0.266667 | MCU | HD | 4 | 0.542639 |
| 0.266667 | MCU | HD | 4 | 0.542639 |
| 0.272727 | MB | HD | 4 | 0.549467 |
| 0.272727 | MB | HD | 4 | 0.549467 |
| 0.272727 | MCU | LD | 4 | 0.549467 |
| 0.277778 | MCU | HD | 4 | 0.555121 |
| 0.285714 | MCU | HD | 4 | 0.563943 |
| 0.294118 | MB | HD | 4 | 0.573203 |
| 0.3 | MCU | LD | 4 | 0.57964 |
| 0.3 | MB | LD | 4 | 0.57964 |
| 0.304348 | MCU | HD | 4 | 0.584374 |
| 0.3125 | MB | HD | 4 | 0.5932 |
| 0.3125 | MCU | LD | 4 | 0.5932 |
| 0.3125 | MCU | LD | 4 | 0.5932 |
| 0.3125 | MCU | LD | 4 | 0.5932 |
| 0.3125 | MB | LD | 4 | 0.5932 |
| 0.315789 | MCU | LD | 4 | 0.596743 |
| 0.333333 | MB | HD | 4 | 0.61548 |
| 0.333333 | MCU | LD | 4 | 0.61548 |
| 0.333333 | MB | LD | 4 | 0.61548 |
| 0.333333 | MB | LD | 4 | 0.61548 |
| 0.333333 | MB | LD | 4 | 0.61548 |
| 0.35 | MCU | LD | 4 | 0.633052 |
| 0.35 | MB | LD | 4 | 0.633052 |
| 0.375 | MB | HD | 4 | 0.659058 |
| 0.375 | MCU | LD | 4 | 0.659058 |
| 0.375 | MB | LD | 4 | 0.659058 |
| 0.375 | MB | LD | 4 | 0.659058 |
| 0.380952 | MB | LD | 4 | 0.665196 |
| 0.384615 | MCU | LD | 4 | 0.668964 |
| 0.4 | MCU | HD | 4 | 0.684719 |
| 0.4 | MB | HD | 4 | 0.684719 |
| 0.4 | MB | HD | 4 | 0.684719 |
| 0.4 | MB | HD | 4 | 0.684719 |
| 0.4 | MB | HD | 4 | 0.684719 |
| 0.4 | MCU | LD | 4 | 0.684719 |
| 0.416667 | MCU | LD | 4 | 0.701674 |
| 0.416667 | MCU | LD | 4 | 0.701674 |
| 0.428571 | MB | HD | 4 | 0.713724 |
| 0.428571 | MB | HD | 4 | 0.713724 |
| 0.428571 | MCU | LD | 4 | 0.713724 |
| 0.428571 | MCU | LD | 4 | 0.713724 |
| 0.428571 | MB | LD | 4 | 0.713724 |
| 0.444444 | MB | HD | 4 | 0.729728 |
| 0.444444 | MCU | LD | 4 | 0.729728 |
| 0.444444 | MB | LD | 4 | 0.729728 |
| 0.45 | MCU | LD | 4 | 0.735314 |
| 0.454545 | MCU | HD | 4 | 0.739881 |
| 0.461538 | MCU | HD | 4 | 0.746899 |
| 0.461538 | MCU | LD | 4 | 0.746899 |
| 0.466667 | MCU | LD | 4 | 0.75204 |
| 0.470588 | MB | LD | 4 | 0.755969 |
| 0.5 | MCU | HD | 4 | 0.785398 |
| 0.5 | MCU | HD | 4 | 0.785398 |
| 0.5 | MCU | HD | 4 | 0.785398 |
| 0.5 | MCU | HD | 4 | 0.785398 |
| 0.5 | MB | HD | 4 | 0.785398 |
| 0.5 | MCU | LD | 4 | 0.785398 |
| 0.5 | MCU | LD | 4 | 0.785398 |
| 0.5 | MB | LD | 4 | 0.785398 |
| 0.5 | MB | LD | 4 | 0.785398 |
| 0.5 | MB | LD | 4 | 0.785398 |
| 0.5 | MB | LD | 4 | 0.785398 |
| 0.533333 | MB | LD | 4 | 0.818756 |
| 0.538462 | MB | LD | 4 | 0.823898 |
| 0.545455 | MB | LD | 4 | 0.830916 |
| 0.555556 | MCU | LD | 4 | 0.841069 |
| 0.5625 | MCU | HD | 4 | 0.848062 |
| 0.571429 | MCU | LD | 4 | 0.857072 |
| 0.571429 | MCU | LD | 4 | 0.857072 |
| 0.583333 | MCU | LD | 4 | 0.869122 |
| 0.583333 | MB | LD | 4 | 0.869122 |
| 0.6 | MB | LD | 4 | 0.886077 |
| 0.615385 | MB | LD | 4 | 0.901832 |
| 0.625 | MB | HD | 4 | 0.911738 |
| 0.625 | MB | LD | 4 | 0.911738 |
| 0.625 | MB | LD | 4 | 0.911738 |
| 0.636364 | MCU | LD | 4 | 0.923511 |
| 0.647059 | MB | HD | 4 | 0.934664 |
| 0.647059 | MB | LD | 4 | 0.934664 |
| 0.666667 | MB | HD | 4 | 0.955317 |
| 0.666667 | MCU | LD | 4 | 0.955317 |
| 0.666667 | MCU | LD | 4 | 0.955317 |
| 0.666667 | MB | LD | 4 | 0.955317 |
| 0.666667 | MB | LD | 4 | 0.955317 |
| 0.7 | MB | LD | 4 | 0.991157 |
| 0.722222 | MB | LD | 4 | 1.015675 |
| 0.727273 | MB | HD | 4 | 1.021329 |
| 0.75 | MB | HD | 4 | 1.047198 |
| 0.75 | MCU | LD | 4 | 1.047198 |
| 0.75 | MB | LD | 4 | 1.047198 |
| 0.75 | MB | LD | 4 | 1.047198 |
| 0.777778 | MCU | LD | 4 | 1.079914 |
| 0.8 | MCU | HD | 4 | 1.107149 |
| 0.8 | MB | HD | 4 | 1.107149 |
| 0.8 | MB | LD | 4 | 1.107149 |
| 0.8 | MB | LD | 4 | 1.107149 |
| 0.8 | MB | LD | 4 | 1.107149 |
| 0.833333 | MB | LD | 4 | 1.150262 |
| 0.857143 | MCU | LD | 4 | 1.1832 |
| 0.866667 | MCU | HD | 4 | 1.197004 |
| 0.888889 | MCU | HD | 4 | 1.230959 |
| 0.916667 | MB | HD | 4 | 1.277954 |
| 0.916667 | MB | HD | 4 | 1.277954 |
| 0.917241 | MB | HD | 4 | 1.278995 |
| 0.933333 | MB | LD | 4 | 1.309639 |
| 0.947368 | MB | HD | 4 | 1.339319 |
| 1 | MCU | HD | 4 | 1.570796 |
| 1 | MB | HD | 4 | 1.570796 |
| 1 | MB | HD | 4 | 1.570796 |
| 1 | MCU | LD | 4 | 1.570796 |
| 1 | MCU | LD | 4 | 1.570796 |
| 1 | MCU | LD | 4 | 1.570796 |
| 1 | MB | LD | 4 | 1.570796 |
| 1 | MB | LD | 4 | 1.570796 |

Supplementary Table 2: 4 day old male P2: Untransformed raw values of P2; Selection: MCU= Populations adapted to larval crowding, MB= Control populations; Treatment: HD= High larval density, LD= Low larval density; Block= Replicate populations of MCU and MB; P2 (arcsin sqrt): Arcsin square root transformed values P2 value

| P2 | Selection | Treatment | Block | P2 (arcsin sqrt) |
| --- | --- | --- | --- | --- |
| 0.2 | MCU | HD | 1 | 0.463648 |
| 0.5 | MCU | HD | 1 | 0.785398 |
| 0.5 | MCU | HD | 1 | 0.785398 |
| 0.555556 | MCU | HD | 1 | 0.841069 |
| 0.4 | MCU | HD | 1 | 0.684719 |
| 0.555556 | MCU | HD | 1 | 0.841069 |
| 0.2 | MCU | HD | 1 | 0.463648 |
| 0.909091 | MCU | HD | 1 | 1.264519 |
| 0.4 | MCU | HD | 1 | 0.684719 |
| 0.75 | MCU | HD | 1 | 1.047198 |
| 0.5 | MCU | HD | 1 | 0.785398 |
| 0.666667 | MCU | HD | 1 | 0.955317 |
| 0.666667 | MCU | HD | 1 | 0.955317 |
| 0.25 | MCU | HD | 1 | 0.523599 |
| 0.333333 | MCU | HD | 1 | 0.61548 |
| 0.142857 | MCU | HD | 1 | 0.387597 |
| 0.111111 | MCU | HD | 1 | 0.339837 |
| 0.714286 | MCU | HD | 1 | 1.006854 |
| 0.5 | MCU | HD | 1 | 0.785398 |
| 0.181818 | MCU | HD | 1 | 0.440511 |
| 0.75 | MCU | HD | 1 | 1.047198 |
| 0.5 | MCU | HD | 1 | 0.785398 |
| 0.2 | MCU | HD | 1 | 0.463648 |
| 0.142857 | MCU | HD | 1 | 0.387597 |
| 0.714286 | MCU | HD | 1 | 1.006854 |
| 0.75 | MCU | HD | 1 | 1.047198 |
| 0.5 | MCU | HD | 1 | 0.785398 |
| 0.25 | MCU | HD | 1 | 0.523599 |
| 0.5 | MCU | HD | 1 | 0.785398 |
| 0.25 | MCU | HD | 1 | 0.523599 |
| 0.142857 | MCU | HD | 1 | 0.387597 |
| 0.272727 | MCU | HD | 1 | 0.549467 |
| 0.8 | MCU | HD | 1 | 1.107149 |
| 0.75 | MCU | HD | 1 | 1.047198 |
| 0.846154 | MCU | HD | 1 | 1.167739 |
| 0.555556 | MCU | HD | 1 | 0.841069 |
| 0.166667 | MCU | HD | 1 | 0.420534 |
| 0.5 | MCU | HD | 1 | 0.785398 |
| 0.666667 | MCU | HD | 1 | 0.955317 |
| 0.166667 | MCU | HD | 1 | 0.420534 |
| 1 | MCU | HD | 1 | 1.570796 |
| 0.6 | MCU | HD | 1 | 0.886077 |
| 0.5 | MCU | HD | 1 | 0.785398 |
| 0.9 | MCU | HD | 1 | 1.249046 |
| 0.571429 | MCU | HD | 1 | 0.857072 |
| 0.142857 | MCU | HD | 1 | 0.387597 |
| 0.066667 | MCU | HD | 1 | 0.261157 |
| 0.5 | MCU | HD | 1 | 0.785398 |
| 0.625 | MCU | HD | 1 | 0.911738 |
| 0.75 | MCU | HD | 1 | 1.047198 |
| 0.9 | MCU | HD | 1 | 1.249046 |
| 0.9 | MCU | HD | 1 | 1.249046 |
| 0.75 | MCU | HD | 1 | 1.047198 |
| 0.769231 | MCU | HD | 1 | 1.069703 |
| 0.75 | MCU | HD | 1 | 1.047198 |
| 0.8 | MCU | HD | 1 | 1.107149 |
| 0.625 | MCU | HD | 1 | 0.911738 |
| 0.333333 | MCU | HD | 1 | 0.61548 |
| 0.555556 | MCU | HD | 1 | 0.841069 |
| 0.818182 | MCU | HD | 1 | 1.130286 |
| 0.6 | MCU | HD | 1 | 0.886077 |
| 0.5 | MCU | HD | 1 | 0.785398 |
| 0.777778 | MCU | HD | 1 | 1.079914 |
| 0.714286 | MCU | HD | 1 | 1.006854 |
| 0.833333 | MCU | HD | 1 | 1.150262 |
| 0.666667 | MCU | HD | 1 | 0.955317 |
| 0.714286 | MCU | HD | 1 | 1.006854 |
| 0.375 | MCU | HD | 1 | 0.659058 |
| 0.5 | MCU | HD | 1 | 0.785398 |
| 0.25 | MCU | HD | 1 | 0.523599 |
| 0.428571 | MCU | HD | 1 | 0.713724 |
| 0.4 | MCU | HD | 1 | 0.684719 |
| 0.857143 | MCU | HD | 1 | 1.1832 |
| 0.5 | MCU | HD | 1 | 0.785398 |
| 0.5 | MCU | HD | 1 | 0.785398 |
| 0.5 | MCU | HD | 1 | 0.785398 |
| 1 | MCU | HD | 1 | 1.570796 |
| 0.571429 | MCU | HD | 1 | 0.857072 |
| 0.666667 | MCU | HD | 1 | 0.955317 |
| 0.625 | MCU | HD | 1 | 0.911738 |
| 0.375 | MCU | HD | 1 | 0.659058 |
| 0.181818 | MCU | HD | 1 | 0.440511 |
| 0.333333 | MCU | HD | 1 | 0.61548 |
| 0.666667 | MCU | HD | 1 | 0.955317 |
| 0.067797 | MCU | HD | 1 | 0.263414 |
| 0.222222 | MCU | HD | 1 | 0.490883 |
| 0.777778 | MCU | HD | 1 | 1.079914 |
| 1 | MCU | LD | 2 | 1.570796 |
| 0.833333 | MCU | LD | 2 | 1.150262 |
| 1 | MCU | LD | 2 | 1.570796 |
| 1 | MCU | LD | 2 | 1.570796 |
| 0.888889 | MCU | LD | 2 | 1.230959 |
| 1 | MCU | LD | 2 | 1.570796 |
| 1 | MCU | LD | 2 | 1.570796 |
| 1 | MCU | LD | 2 | 1.570796 |
| 1 | MCU | LD | 2 | 1.570796 |
| 0.142857 | MCU | LD | 2 | 0.387597 |
| 1 | MCU | LD | 2 | 1.570796 |
| 1 | MCU | LD | 2 | 1.570796 |
| 0.5 | MCU | LD | 2 | 0.785398 |
| 0.571429 | MCU | LD | 2 | 0.857072 |
| 1 | MCU | LD | 2 | 1.570796 |
| 0.9 | MCU | LD | 2 | 1.249046 |
| 0.875 | MCU | LD | 2 | 1.209429 |
| 0.8 | MCU | LD | 2 | 1.107149 |
| 1 | MCU | LD | 2 | 1.570796 |
| 0.9375 | MCU | LD | 2 | 1.318116 |
| 1 | MCU | LD | 2 | 1.570796 |
| 0.909091 | MCU | LD | 2 | 1.264519 |
| 0.714286 | MCU | LD | 2 | 1.006854 |
| 1 | MCU | LD | 2 | 1.570796 |
| 0.8 | MCU | LD | 2 | 1.107149 |
| 1 | MCU | LD | 2 | 1.570796 |
| 0.928571 | MCU | LD | 2 | 1.300247 |
| 0.5 | MCU | LD | 2 | 0.785398 |
| 0.857143 | MCU | LD | 2 | 1.1832 |
| 1 | MCU | LD | 2 | 1.570796 |
| 1 | MCU | LD | 2 | 1.570796 |
| 1 | MCU | LD | 2 | 1.570796 |
| 1 | MCU | LD | 2 | 1.570796 |
| 1 | MCU | LD | 2 | 1.570796 |
| 0.846154 | MCU | LD | 2 | 1.167739 |
| 0.444444 | MCU | LD | 2 | 0.729728 |
| 1 | MCU | LD | 2 | 1.570796 |
| 1 | MCU | LD | 2 | 1.570796 |
| 1 | MCU | LD | 2 | 1.570796 |
| 1 | MCU | LD | 2 | 1.570796 |
| 1 | MCU | LD | 2 | 1.570796 |
| 0.875 | MCU | LD | 2 | 1.209429 |
| 0.833333 | MCU | LD | 2 | 1.150262 |
| 1 | MCU | LD | 2 | 1.570796 |
| 1 | MCU | LD | 2 | 1.570796 |
| 1 | MCU | LD | 2 | 1.570796 |
| 0.875 | MCU | LD | 2 | 1.209429 |
| 1 | MCU | LD | 2 | 1.570796 |
| 1 | MCU | LD | 2 | 1.570796 |
| 1 | MCU | LD | 2 | 1.570796 |
| 1 | MCU | LD | 2 | 1.570796 |
| 1 | MCU | LD | 2 | 1.570796 |
| 0.571429 | MCU | LD | 2 | 0.857072 |
| 1 | MCU | LD | 2 | 1.570796 |
| 1 | MCU | LD | 2 | 1.570796 |
| 1 | MCU | LD | 2 | 1.570796 |
| 0.777778 | MCU | HD | 2 | 1.079914 |
| 0.727273 | MCU | HD | 2 | 1.021329 |
| 0.1 | MCU | HD | 2 | 0.321751 |
| 0.8 | MCU | HD | 2 | 1.107149 |
| 1 | MCU | HD | 2 | 1.570796 |
| 0.8 | MCU | HD | 2 | 1.107149 |
| 1 | MCU | HD | 2 | 1.570796 |
| 0.777778 | MCU | HD | 2 | 1.079914 |
| 0.272727 | MCU | HD | 2 | 0.549467 |
| 0.75 | MCU | HD | 2 | 1.047198 |
| 0.857143 | MCU | HD | 2 | 1.1832 |
| 0.666667 | MCU | HD | 2 | 0.955317 |
| 0.8 | MCU | HD | 2 | 1.107149 |
| 0.833333 | MCU | HD | 2 | 1.150262 |
| 0.4 | MCU | HD | 2 | 0.684719 |
| 0.6 | MCU | HD | 2 | 0.886077 |
| 1 | MCU | HD | 2 | 1.570796 |
| 1 | MCU | HD | 2 | 1.570796 |
| 0.25 | MCU | HD | 2 | 0.523599 |
| 0.666667 | MCU | HD | 2 | 0.955317 |
| 0.5 | MCU | HD | 2 | 0.785398 |
| 0.714286 | MCU | HD | 2 | 1.006854 |
| 0.5 | MCU | HD | 2 | 0.785398 |
| 0.6 | MCU | HD | 2 | 0.886077 |
| 0.692308 | MCU | HD | 2 | 0.982794 |
| 0.4 | MCU | HD | 2 | 0.684719 |
| 0.7 | MCU | HD | 2 | 0.991157 |
| 0.6 | MCU | HD | 2 | 0.886077 |
| 0.75 | MCU | HD | 2 | 1.047198 |
| 0.5 | MCU | HD | 2 | 0.785398 |
| 0.5 | MCU | HD | 2 | 0.785398 |
| 1 | MCU | HD | 2 | 1.570796 |
| 0.5 | MCU | HD | 2 | 0.785398 |
| 0.5 | MCU | HD | 2 | 0.785398 |
| 0.888889 | MCU | HD | 2 | 1.230959 |
| 0.8 | MCU | HD | 2 | 1.107149 |
| 0.875 | MCU | HD | 2 | 1.209429 |
| 0.125 | MCU | HD | 2 | 0.361367 |
| 0.363636 | MCU | HD | 2 | 0.647285 |
| 1 | MCU | HD | 2 | 1.570796 |
| 0.714286 | MCU | HD | 2 | 1.006854 |
| 1 | MCU | HD | 2 | 1.570796 |
| 0.625 | MCU | HD | 2 | 0.911738 |
| 0.777778 | MCU | HD | 2 | 1.079914 |
| 1 | MCU | HD | 2 | 1.570796 |
| 0.4 | MCU | HD | 2 | 0.684719 |
| 0.714286 | MCU | HD | 2 | 1.006854 |
| 1 | MCU | HD | 2 | 1.570796 |
| 1 | MCU | HD | 2 | 1.570796 |
| 0.625 | MCU | HD | 2 | 0.911738 |
| 1 | MCU | HD | 2 | 1.570796 |
| 1 | MCU | HD | 2 | 1.570796 |
| 0.142857 | MCU | HD | 2 | 0.387597 |
| 0.916667 | MCU | HD | 2 | 1.277954 |
| 0.714286 | MCU | HD | 2 | 1.006854 |
| 0.875 | MCU | HD | 2 | 1.209429 |
| 0.5 | MCU | HD | 2 | 0.785398 |
| 1 | MCU | HD | 2 | 1.570796 |
| 0.533333 | MCU | HD | 2 | 0.818756 |
| 0.555556 | MCU | HD | 2 | 0.841069 |
| 0.714286 | MCU | HD | 2 | 1.006854 |
| 0.833333 | MCU | HD | 2 | 1.150262 |
| 0.75 | MCU | HD | 2 | 1.047198 |
| 0.466667 | MCU | HD | 2 | 0.75204 |
| 1 | MCU | HD | 2 | 1.570796 |
| 0.5 | MCU | HD | 2 | 0.785398 |
| 1 | MCU | HD | 2 | 1.570796 |
| 0.75 | MCU | HD | 2 | 1.047198 |
| 0.25 | MCU | HD | 2 | 0.523599 |
| 0.5 | MCU | HD | 2 | 0.785398 |
| 1 | MCU | HD | 2 | 1.570796 |
| 1 | MCU | HD | 2 | 1.570796 |
| 0.8 | MCU | HD | 2 | 1.107149 |
| 0.875 | MCU | HD | 2 | 1.209429 |
| 0.25 | MCU | HD | 2 | 0.523599 |
| 0.857143 | MCU | HD | 2 | 1.1832 |
| 0.818182 | MCU | HD | 2 | 1.130286 |
| 0.666667 | MCU | HD | 2 | 0.955317 |
| 1 | MCU | HD | 2 | 1.570796 |
| 1 | MCU | HD | 2 | 1.570796 |
| 0.285714 | MCU | HD | 2 | 0.563943 |
| 0.583333 | MCU | HD | 2 | 0.869122 |
| 0.769231 | MCU | HD | 2 | 1.069703 |
| 0.5 | MCU | HD | 2 | 0.785398 |
| 0.8 | MCU | HD | 2 | 1.107149 |
| 0.666667 | MCU | HD | 2 | 0.955317 |
| 0.571429 | MCU | HD | 2 | 0.857072 |
| 0.6 | MCU | HD | 2 | 0.886077 |
| 0.571429 | MCU | HD | 2 | 0.857072 |
| 1 | MCU | HD | 2 | 1.570796 |
| 0.916667 | MCU | HD | 2 | 1.277954 |
| 0.714286 | MCU | HD | 2 | 1.006854 |
| 1 | MCU | HD | 2 | 1.570796 |
| 0.75 | MCU | HD | 2 | 1.047198 |
| 0.333333 | MCU | HD | 2 | 0.61548 |
| 0.5 | MCU | HD | 2 | 0.785398 |
| 0.7 | MCU | HD | 2 | 0.991157 |
| 0.75 | MCU | HD | 2 | 1.047198 |
| 0.777778 | MCU | HD | 2 | 1.079914 |
| 0.818182 | MCU | HD | 2 | 1.130286 |
| 0.666667 | MCU | HD | 2 | 0.955317 |
| 0.2 | MCU | HD | 2 | 0.463648 |
| 1 | MCU | HD | 2 | 1.570796 |
| 0.222222 | MCU | HD | 2 | 0.490883 |
| 0.5 | MCU | HD | 2 | 0.785398 |
| 0.875 | MCU | HD | 2 | 1.209429 |
| 0.75 | MCU | HD | 2 | 1.047198 |
| 0.4 | MCU | HD | 2 | 0.684719 |
| 0.25 | MCU | HD | 2 | 0.523599 |
| 0.571429 | MCU | HD | 2 | 0.857072 |
| 1 | MCU | HD | 2 | 1.570796 |
| 0.909091 | MCU | HD | 2 | 1.264519 |
| 0.75 | MCU | HD | 2 | 1.047198 |
| 1 | MCU | HD | 2 | 1.570796 |
| 0.75 | MCU | HD | 2 | 1.047198 |
| 1 | MCU | HD | 3 | 1.570796 |
| 1 | MCU | HD | 3 | 1.570796 |
| 1 | MCU | HD | 3 | 1.570796 |
| 1 | MCU | HD | 3 | 1.570796 |
| 1 | MCU | HD | 3 | 1.570796 |
| 1 | MCU | HD | 3 | 1.570796 |
| 1 | MCU | HD | 3 | 1.570796 |
| 1 | MCU | HD | 3 | 1.570796 |
| 1 | MCU | HD | 3 | 1.570796 |
| 1 | MCU | HD | 3 | 1.570796 |
| 1 | MCU | HD | 3 | 1.570796 |
| 1 | MCU | HD | 3 | 1.570796 |
| 1 | MCU | HD | 3 | 1.570796 |
| 1 | MCU | HD | 3 | 1.570796 |
| 1 | MCU | HD | 3 | 1.570796 |
| 1 | MCU | HD | 3 | 1.570796 |
| 1 | MCU | HD | 3 | 1.570796 |
| 1 | MCU | HD | 3 | 1.570796 |
| 1 | MCU | HD | 3 | 1.570796 |
| 1 | MCU | HD | 3 | 1.570796 |
| 1 | MCU | HD | 3 | 1.570796 |
| 1 | MCU | HD | 3 | 1.570796 |
| 1 | MCU | HD | 3 | 1.570796 |
| 1 | MCU | HD | 3 | 1.570796 |
| 1 | MCU | HD | 3 | 1.570796 |
| 1 | MCU | HD | 3 | 1.570796 |
| 1 | MCU | HD | 3 | 1.570796 |
| 1 | MCU | HD | 3 | 1.570796 |
| 1 | MCU | HD | 3 | 1.570796 |
| 1 | MCU | HD | 3 | 1.570796 |
| 1 | MCU | HD | 3 | 1.570796 |
| 1 | MCU | HD | 3 | 1.570796 |
| 1 | MCU | LD | 3 | 1.570796 |
| 1 | MCU | LD | 3 | 1.570796 |
| 1 | MCU | LD | 3 | 1.570796 |
| 1 | MCU | LD | 3 | 1.570796 |
| 1 | MCU | LD | 3 | 1.570796 |
| 1 | MCU | LD | 3 | 1.570796 |
| 1 | MCU | LD | 3 | 1.570796 |
| 1 | MCU | LD | 3 | 1.570796 |
| 1 | MCU | LD | 3 | 1.570796 |
| 1 | MCU | LD | 3 | 1.570796 |
| 1 | MCU | LD | 3 | 1.570796 |
| 1 | MCU | LD | 3 | 1.570796 |
| 1 | MCU | LD | 3 | 1.570796 |
| 1 | MCU | LD | 3 | 1.570796 |
| 1 | MCU | LD | 3 | 1.570796 |
| 1 | MCU | LD | 3 | 1.570796 |
| 1 | MCU | LD | 3 | 1.570796 |
| 1 | MCU | LD | 3 | 1.570796 |
| 1 | MCU | LD | 3 | 1.570796 |
| 1 | MCU | LD | 3 | 1.570796 |
| 1 | MCU | LD | 3 | 1.570796 |
| 0.947368 | MCU | LD | 3 | 1.339319 |
| 0.928571 | MCU | HD | 3 | 1.300247 |
| 0.928571 | MCU | LD | 3 | 1.300247 |
| 0.928571 | MCU | LD | 3 | 1.300247 |
| 0.928571 | MCU | LD | 3 | 1.300247 |
| 0.923077 | MCU | HD | 3 | 1.289761 |
| 0.923077 | MCU | HD | 3 | 1.289761 |
| 0.923077 | MCU | HD | 3 | 1.289761 |
| 0.923077 | MCU | LD | 3 | 1.289761 |
| 0.916667 | MCU | HD | 3 | 1.277954 |
| 0.916667 | MCU | HD | 3 | 1.277954 |
| 0.916667 | MCU | LD | 3 | 1.277954 |
| 0.916667 | MCU | LD | 3 | 1.277954 |
| 0.909091 | MCU | HD | 3 | 1.264519 |
| 0.909091 | MCU | HD | 3 | 1.264519 |
| 0.909091 | MCU | HD | 3 | 1.264519 |
| 0.909091 | MCU | HD | 3 | 1.264519 |
| 0.909091 | MCU | LD | 3 | 1.264519 |
| 0.9 | MCU | HD | 3 | 1.249046 |
| 0.9 | MCU | HD | 3 | 1.249046 |
| 0.9 | MCU | HD | 3 | 1.249046 |
| 0.9 | MCU | HD | 3 | 1.249046 |
| 0.9 | MCU | LD | 3 | 1.249046 |
| 0.9 | MCU | LD | 3 | 1.249046 |
| 0.894737 | MCU | LD | 3 | 1.240374 |
| 0.888889 | MCU | HD | 3 | 1.230959 |
| 0.888889 | MCU | HD | 3 | 1.230959 |
| 0.888889 | MCU | HD | 3 | 1.230959 |
| 0.888889 | MCU | HD | 3 | 1.230959 |
| 0.888889 | MCU | HD | 3 | 1.230959 |
| 0.888889 | MCU | LD | 3 | 1.230959 |
| 0.888889 | MCU | LD | 3 | 1.230959 |
| 0.888889 | MCU | LD | 3 | 1.230959 |
| 0.878049 | MCU | LD | 3 | 1.214063 |
| 0.875 | MCU | HD | 3 | 1.209429 |
| 0.875 | MCU | HD | 3 | 1.209429 |
| 0.875 | MCU | HD | 3 | 1.209429 |
| 0.875 | MCU | LD | 3 | 1.209429 |
| 0.875 | MCU | LD | 3 | 1.209429 |
| 0.875 | MCU | LD | 3 | 1.209429 |
| 0.866667 | MCU | HD | 3 | 1.197004 |
| 0.866667 | MCU | LD | 3 | 1.197004 |
| 0.866667 | MCU | LD | 3 | 1.197004 |
| 0.866667 | MCU | LD | 3 | 1.197004 |
| 0.857143 | MCU | HD | 3 | 1.1832 |
| 0.857143 | MCU | HD | 3 | 1.1832 |
| 0.857143 | MCU | HD | 3 | 1.1832 |
| 0.857143 | MCU | LD | 3 | 1.1832 |
| 0.846154 | MCU | HD | 3 | 1.167739 |
| 0.846154 | MCU | HD | 3 | 1.167739 |
| 0.846154 | MCU | HD | 3 | 1.167739 |
| 0.842105 | MCU | LD | 3 | 1.162158 |
| 0.833333 | MCU | HD | 3 | 1.150262 |
| 0.833333 | MCU | LD | 3 | 1.150262 |
| 0.833333 | MCU | LD | 3 | 1.150262 |
| 0.823529 | MCU | HD | 3 | 1.137258 |
| 0.823529 | MCU | LD | 3 | 1.137258 |
| 0.818182 | MCU | HD | 3 | 1.130286 |
| 0.8 | MCU | HD | 3 | 1.107149 |
| 0.8 | MCU | HD | 3 | 1.107149 |
| 0.8 | MCU | HD | 3 | 1.107149 |
| 0.8 | MCU | HD | 3 | 1.107149 |
| 0.8 | MCU | LD | 3 | 1.107149 |
| 0.777778 | MCU | HD | 3 | 1.079914 |
| 0.769231 | MCU | LD | 3 | 1.069703 |
| 0.75 | MCU | HD | 3 | 1.047198 |
| 0.75 | MCU | HD | 3 | 1.047198 |
| 0.75 | MCU | HD | 3 | 1.047198 |
| 0.75 | MCU | LD | 3 | 1.047198 |
| 0.75 | MCU | LD | 3 | 1.047198 |
| 0.727273 | MCU | HD | 3 | 1.021329 |
| 0.714286 | MCU | HD | 3 | 1.006854 |
| 0.7 | MCU | HD | 3 | 0.991157 |
| 0.692308 | MCU | HD | 3 | 0.982794 |
| 0.692308 | MCU | HD | 3 | 0.982794 |
| 0.684211 | MCU | HD | 3 | 0.974053 |
| 0.684211 | MCU | HD | 3 | 0.974053 |
| 0.666667 | MCU | LD | 3 | 0.955317 |
| 0.666667 | MCU | LD | 3 | 0.955317 |
| 0.647059 | MCU | HD | 3 | 0.934664 |
| 0.642857 | MCU | HD | 3 | 0.930274 |
| 0.642857 | MCU | LD | 3 | 0.930274 |
| 0.636364 | MCU | HD | 3 | 0.923511 |
| 0.636364 | MCU | HD | 3 | 0.923511 |
| 0.636364 | MCU | HD | 3 | 0.923511 |
| 0.625 | MCU | HD | 3 | 0.911738 |
| 0.625 | MCU | HD | 3 | 0.911738 |
| 0.625 | MCU | HD | 3 | 0.911738 |
| 0.615385 | MCU | HD | 3 | 0.901832 |
| 0.615385 | MCU | LD | 3 | 0.901832 |
| 0.6 | MCU | HD | 3 | 0.886077 |
| 0.571429 | MCU | HD | 3 | 0.857072 |
| 0.571429 | MCU | LD | 3 | 0.857072 |
| 0.555556 | MCU | HD | 3 | 0.841069 |
| 0.545455 | MCU | HD | 3 | 0.830916 |
| 0.533333 | MCU | HD | 3 | 0.818756 |
| 0.533333 | MCU | LD | 3 | 0.818756 |
| 0.5 | MCU | HD | 3 | 0.785398 |
| 0.5 | MCU | HD | 3 | 0.785398 |
| 0.5 | MCU | HD | 3 | 0.785398 |
| 0.444444 | MCU | HD | 3 | 0.729728 |
| 0.444444 | MCU | LD | 3 | 0.729728 |
| 0.4 | MCU | HD | 3 | 0.684719 |
| 0.4 | MCU | HD | 3 | 0.684719 |
| 0.384615 | MCU | HD | 3 | 0.668964 |
| 0.368421 | MCU | HD | 3 | 0.652251 |
| 0.333333 | MCU | HD | 3 | 0.61548 |
| 0.291667 | MCU | HD | 3 | 0.57051 |
| 0.25 | MCU | HD | 3 | 0.523599 |
| 0.25 | MCU | LD | 3 | 0.523599 |
| 0.117647 | MCU | HD | 3 | 0.350106 |
| 1 | MCU | LD | 4 | 1.570796 |
| 1 | MCU | LD | 4 | 1.570796 |
| 1 | MCU | LD | 4 | 1.570796 |
| 1 | MCU | LD | 4 | 1.570796 |
| 1 | MCU | LD | 4 | 1.570796 |
| 1 | MCU | LD | 4 | 1.570796 |
| 1 | MCU | LD | 4 | 1.570796 |
| 1 | MCU | LD | 4 | 1.570796 |
| 1 | MCU | LD | 4 | 1.570796 |
| 1 | MCU | LD | 4 | 1.570796 |
| 1 | MCU | LD | 4 | 1.570796 |
| 1 | MCU | LD | 4 | 1.570796 |
| 1 | MCU | LD | 4 | 1.570796 |
| 1 | MCU | LD | 4 | 1.570796 |
| 1 | MCU | LD | 4 | 1.570796 |
| 1 | MCU | LD | 4 | 1.570796 |
| 1 | MCU | LD | 4 | 1.570796 |
| 1 | MCU | LD | 4 | 1.570796 |
| 1 | MCU | LD | 4 | 1.570796 |
| 1 | MCU | LD | 4 | 1.570796 |
| 1 | MCU | LD | 4 | 1.570796 |
| 1 | MCU | LD | 4 | 1.570796 |
| 1 | MCU | LD | 4 | 1.570796 |
| 1 | MCU | LD | 4 | 1.570796 |
| 1 | MCU | LD | 4 | 1.570796 |
| 1 | MCU | LD | 4 | 1.570796 |
| 1 | MCU | LD | 4 | 1.570796 |
| 1 | MCU | LD | 4 | 1.570796 |
| 1 | MCU | HD | 4 | 1.570796 |
| 1 | MCU | HD | 4 | 1.570796 |
| 1 | MCU | HD | 4 | 1.570796 |
| 1 | MCU | HD | 4 | 1.570796 |
| 1 | MCU | HD | 4 | 1.570796 |
| 1 | MCU | HD | 4 | 1.570796 |
| 1 | MCU | HD | 4 | 1.570796 |
| 1 | MCU | HD | 4 | 1.570796 |
| 1 | MCU | HD | 4 | 1.570796 |
| 1 | MCU | HD | 4 | 1.570796 |
| 1 | MCU | HD | 4 | 1.570796 |
| 1 | MCU | HD | 4 | 1.570796 |
| 1 | MCU | HD | 4 | 1.570796 |
| 1 | MCU | HD | 4 | 1.570796 |
| 1 | MCU | HD | 4 | 1.570796 |
| 1 | MCU | HD | 4 | 1.570796 |
| 1 | MCU | HD | 4 | 1.570796 |
| 1 | MCU | HD | 4 | 1.570796 |
| 1 | MCU | HD | 4 | 1.570796 |
| 1 | MCU | HD | 4 | 1.570796 |
| 1 | MCU | HD | 4 | 1.570796 |
| 1 | MCU | HD | 4 | 1.570796 |
| 1 | MCU | HD | 4 | 1.570796 |
| 1 | MCU | HD | 4 | 1.570796 |
| 1 | MCU | HD | 4 | 1.570796 |
| 1 | MCU | HD | 4 | 1.570796 |
| 1 | MCU | HD | 4 | 1.570796 |
| 1 | MCU | HD | 4 | 1.570796 |
| 1 | MCU | HD | 4 | 1.570796 |
| 1 | MCU | HD | 4 | 1.570796 |
| 1 | MCU | HD | 4 | 1.570796 |
| 1 | MCU | HD | 4 | 1.570796 |
| 1 | MCU | HD | 4 | 1.570796 |
| 0.971429 | MCU | LD | 4 | 1.40095 |
| 0.933333 | MCU | HD | 4 | 1.309639 |
| 0.916667 | MCU | HD | 4 | 1.277954 |
| 0.909091 | MCU | HD | 4 | 1.264519 |
| 0.9 | MCU | HD | 4 | 1.249046 |
| 0.9 | MCU | HD | 4 | 1.249046 |
| 0.888889 | MCU | LD | 4 | 1.230959 |
| 0.888889 | MCU | LD | 4 | 1.230959 |
| 0.888889 | MCU | HD | 4 | 1.230959 |
| 0.888889 | MCU | HD | 4 | 1.230959 |
| 0.875 | MCU | LD | 4 | 1.209429 |
| 0.875 | MCU | HD | 4 | 1.209429 |
| 0.866667 | MCU | HD | 4 | 1.197004 |
| 0.857143 | MCU | LD | 4 | 1.1832 |
| 0.857143 | MCU | HD | 4 | 1.1832 |
| 0.857143 | MCU | HD | 4 | 1.1832 |
| 0.857143 | MCU | HD | 4 | 1.1832 |
| 0.857143 | MCU | HD | 4 | 1.1832 |
| 0.846154 | MCU | LD | 4 | 1.167739 |
| 0.846154 | MCU | LD | 4 | 1.167739 |
| 0.846154 | MCU | HD | 4 | 1.167739 |
| 0.833333 | MCU | LD | 4 | 1.150262 |
| 0.833333 | MCU | LD | 4 | 1.150262 |
| 0.818182 | MCU | LD | 4 | 1.130286 |
| 0.818182 | MCU | HD | 4 | 1.130286 |
| 0.818182 | MCU | HD | 4 | 1.130286 |
| 0.818182 | MCU | HD | 4 | 1.130286 |
| 0.818182 | MCU | HD | 4 | 1.130286 |
| 0.8125 | MCU | LD | 4 | 1.122964 |
| 0.8 | MCU | LD | 4 | 1.107149 |
| 0.8 | MCU | LD | 4 | 1.107149 |
| 0.785714 | MCU | HD | 4 | 1.089521 |
| 0.777778 | MCU | HD | 4 | 1.079914 |
| 0.777778 | MCU | HD | 4 | 1.079914 |
| 0.777778 | MCU | HD | 4 | 1.079914 |
| 0.75 | MCU | LD | 4 | 1.047198 |
| 0.75 | MCU | LD | 4 | 1.047198 |
| 0.75 | MCU | LD | 4 | 1.047198 |
| 0.75 | MCU | LD | 4 | 1.047198 |
| 0.75 | MCU | HD | 4 | 1.047198 |
| 0.75 | MCU | HD | 4 | 1.047198 |
| 0.75 | MCU | HD | 4 | 1.047198 |
| 0.75 | MCU | HD | 4 | 1.047198 |
| 0.75 | MCU | HD | 4 | 1.047198 |
| 0.714286 | MCU | HD | 4 | 1.006854 |
| 0.714286 | MCU | HD | 4 | 1.006854 |
| 0.714286 | MCU | HD | 4 | 1.006854 |
| 0.7 | MCU | LD | 4 | 0.991157 |
| 0.7 | MCU | HD | 4 | 0.991157 |
| 0.7 | MCU | HD | 4 | 0.991157 |
| 0.666667 | MCU | LD | 4 | 0.955317 |
| 0.666667 | MCU | LD | 4 | 0.955317 |
| 0.666667 | MCU | HD | 4 | 0.955317 |
| 0.666667 | MCU | HD | 4 | 0.955317 |
| 0.666667 | MCU | HD | 4 | 0.955317 |
| 0.666667 | MCU | HD | 4 | 0.955317 |
| 0.666667 | MCU | HD | 4 | 0.955317 |
| 0.666667 | MCU | HD | 4 | 0.955317 |
| 0.636364 | MCU | LD | 4 | 0.923511 |
| 0.636364 | MCU | HD | 4 | 0.923511 |
| 0.636364 | MCU | HD | 4 | 0.923511 |
| 0.625 | MCU | LD | 4 | 0.911738 |
| 0.625 | MCU | HD | 4 | 0.911738 |
| 0.625 | MCU | HD | 4 | 0.911738 |
| 0.615385 | MCU | LD | 4 | 0.901832 |
| 0.615385 | MCU | HD | 4 | 0.901832 |
| 0.6 | MCU | LD | 4 | 0.886077 |
| 0.6 | MCU | HD | 4 | 0.886077 |
| 0.583333 | MCU | HD | 4 | 0.869122 |
| 0.571429 | MCU | HD | 4 | 0.857072 |
| 0.555556 | MCU | LD | 4 | 0.841069 |
| 0.555556 | MCU | HD | 4 | 0.841069 |
| 0.545455 | MCU | HD | 4 | 0.830916 |
| 0.545455 | MCU | HD | 4 | 0.830916 |
| 0.52 | MCU | HD | 4 | 0.805404 |
| 0.5 | MCU | LD | 4 | 0.785398 |
| 0.5 | MCU | LD | 4 | 0.785398 |
| 0.5 | MCU | HD | 4 | 0.785398 |
| 0.461538 | MCU | LD | 4 | 0.746899 |
| 0.454545 | MCU | HD | 4 | 0.739881 |
| 0.444444 | MCU | HD | 4 | 0.729728 |
| 0.428571 | MCU | LD | 4 | 0.713724 |
| 0.428571 | MCU | HD | 4 | 0.713724 |
| 0.32 | MCU | LD | 4 | 0.601264 |
| 0.3 | MCU | HD | 4 | 0.57964 |
| 0.25 | MCU | HD | 4 | 0.523599 |
| 0.214286 | MCU | HD | 4 | 0.481275 |
| 0.2 | MCU | HD | 4 | 0.463648 |
| 0.166667 | MCU | LD | 4 | 0.420534 |
| 0.1 | MCU | HD | 4 | 0.321751 |
| 0.090909 | MCU | HD | 4 | 0.306277 |
| 1 | MCU | LD | 1 | 1.570796 |
| 1 | MCU | LD | 1 | 1.570796 |
| 0.75 | MCU | LD | 1 | 1.047198 |
| 0.5 | MCU | LD | 1 | 0.785398 |
| 0.333333 | MCU | LD | 1 | 0.61548 |
| 1 | MCU | LD | 1 | 1.570796 |
| 0.857143 | MCU | LD | 1 | 1.1832 |
| 1 | MCU | LD | 1 | 1.570796 |
| 1 | MCU | LD | 1 | 1.570796 |
| 1 | MCU | LD | 1 | 1.570796 |
| 1 | MCU | LD | 1 | 1.570796 |
| 0.666667 | MCU | LD | 1 | 0.955317 |
| 0.818182 | MCU | LD | 1 | 1.130286 |
| 0.75 | MCU | LD | 1 | 1.047198 |
| 1 | MCU | LD | 1 | 1.570796 |
| 0.714286 | MCU | LD | 1 | 1.006854 |
| 0.857143 | MCU | LD | 1 | 1.1832 |
| 1 | MCU | LD | 1 | 1.570796 |
| 0.833333 | MCU | LD | 1 | 1.150262 |
| 1 | MCU | LD | 1 | 1.570796 |
| 0.857143 | MCU | LD | 1 | 1.1832 |
| 0.7 | MCU | LD | 1 | 0.991157 |
| 0.857143 | MCU | LD | 1 | 1.1832 |
| 0.714286 | MCU | LD | 1 | 1.006854 |
| 0.857143 | MCU | LD | 1 | 1.1832 |
| 0.75 | MCU | LD | 1 | 1.047198 |
| 1 | MCU | LD | 1 | 1.570796 |
| 1 | MCU | LD | 1 | 1.570796 |
| 0.5 | MCU | LD | 1 | 0.785398 |
| 1 | MCU | LD | 1 | 1.570796 |
| 0.75 | MCU | LD | 1 | 1.047198 |
| 1 | MCU | LD | 1 | 1.570796 |
| 0.666667 | MCU | LD | 1 | 0.955317 |
| 0.818182 | MCU | LD | 1 | 1.130286 |
| 0.7 | MCU | LD | 1 | 0.991157 |
| 0.4 | MCU | LD | 1 | 0.684719 |
| 1 | MCU | LD | 1 | 1.570796 |
| 1 | MCU | LD | 1 | 1.570796 |
| 0.75 | MCU | LD | 1 | 1.047198 |
| 1 | MCU | LD | 1 | 1.570796 |
| 0.75 | MCU | LD | 1 | 1.047198 |
| 0.7 | MCU | LD | 1 | 0.991157 |
| 0.888889 | MCU | LD | 1 | 1.230959 |
| 0.25 | MCU | LD | 1 | 0.523599 |
| 1 | MCU | LD | 1 | 1.570796 |
| 0.75 | MCU | LD | 1 | 1.047198 |
| 0.7 | MCU | LD | 1 | 0.991157 |
| 0.666667 | MCU | LD | 1 | 0.955317 |
| 0.75 | MCU | LD | 1 | 1.047198 |
| 0.75 | MCU | LD | 1 | 1.047198 |
| 0.857143 | MCU | LD | 1 | 1.1832 |
| 1 | MCU | LD | 1 | 1.570796 |
| 0.75 | MCU | LD | 1 | 1.047198 |
| 0.4 | MCU | LD | 1 | 0.684719 |
| 0.875 | MCU | LD | 1 | 1.209429 |
| 0.5 | MCU | LD | 1 | 0.785398 |
| 0.36 | MB | HD | 1 | 0.643501 |
| 1 | MB | HD | 1 | 1.570796 |
| 0.2 | MB | HD | 1 | 0.463648 |
| 0.857143 | MB | HD | 1 | 1.1832 |
| 0.166667 | MB | HD | 1 | 0.420534 |
| 0.833333 | MB | HD | 1 | 1.150262 |
| 0.777778 | MB | HD | 1 | 1.079914 |
| 1 | MB | HD | 1 | 1.570796 |
| 1 | MB | HD | 1 | 1.570796 |
| 1 | MB | HD | 1 | 1.570796 |
| 0.8 | MB | HD | 1 | 1.107149 |
| 1 | MB | HD | 1 | 1.570796 |
| 0.818182 | MB | HD | 1 | 1.130286 |
| 0.8 | MB | HD | 1 | 1.107149 |
| 0.846154 | MB | HD | 1 | 1.167739 |
| 0.75 | MB | HD | 1 | 1.047198 |
| 0.4 | MB | HD | 1 | 0.684719 |
| 0.166667 | MB | HD | 1 | 0.420534 |
| 0.6 | MB | HD | 1 | 0.886077 |
| 1 | MB | HD | 1 | 1.570796 |
| 0.272727 | MB | HD | 1 | 0.549467 |
| 0.083333 | MB | HD | 1 | 0.292843 |
| 0.75 | MB | HD | 1 | 1.047198 |
| 0.8 | MB | HD | 1 | 1.107149 |
| 0.777778 | MB | HD | 1 | 1.079914 |
| 1 | MB | HD | 1 | 1.570796 |
| 0.75 | MB | HD | 1 | 1.047198 |
| 1 | MB | HD | 1 | 1.570796 |
| 0.333333 | MB | HD | 1 | 0.61548 |
| 0.2 | MB | HD | 1 | 0.463648 |
| 0.3 | MB | HD | 1 | 0.57964 |
| 0.818182 | MB | HD | 1 | 1.130286 |
| 0.5 | MB | HD | 1 | 0.785398 |
| 0.833333 | MB | HD | 1 | 1.150262 |
| 1 | MB | HD | 1 | 1.570796 |
| 1 | MB | HD | 1 | 1.570796 |
| 0.5 | MB | HD | 1 | 0.785398 |
| 0.571429 | MB | HD | 1 | 0.857072 |
| 0.6 | MB | HD | 1 | 0.886077 |
| 1 | MB | HD | 1 | 1.570796 |
| 0.142857 | MB | HD | 1 | 0.387597 |
| 0.125 | MB | HD | 1 | 0.361367 |
| 0.625 | MB | HD | 1 | 0.911738 |
| 1 | MB | HD | 1 | 1.570796 |
| 0.5 | MB | HD | 1 | 0.785398 |
| 0.2 | MB | HD | 1 | 0.463648 |
| 0.875 | MB | HD | 1 | 1.209429 |
| 0.9 | MB | HD | 1 | 1.249046 |
| 0.333333 | MB | HD | 1 | 0.61548 |
| 0.333333 | MB | HD | 1 | 0.61548 |
| 0.6 | MB | HD | 1 | 0.886077 |
| 1 | MB | HD | 1 | 1.570796 |
| 0.5 | MB | HD | 1 | 0.785398 |
| 0.666667 | MB | HD | 1 | 0.955317 |
| 0.428571 | MB | HD | 1 | 0.713724 |
| 0.333333 | MB | HD | 1 | 0.61548 |
| 0.333333 | MB | HD | 1 | 0.61548 |
| 0.5 | MB | HD | 1 | 0.785398 |
| 1 | MB | HD | 1 | 1.570796 |
| 0.666667 | MB | HD | 1 | 0.955317 |
| 1 | MB | HD | 1 | 1.570796 |
| 0.285714 | MB | HD | 1 | 0.563943 |
| 0.8 | MB | HD | 1 | 1.107149 |
| 1 | MB | HD | 1 | 1.570796 |
| 0.25 | MB | HD | 1 | 0.523599 |
| 0.6 | MB | HD | 1 | 0.886077 |
| 0.714286 | MB | HD | 1 | 1.006854 |
| 1 | MB | HD | 1 | 1.570796 |
| 0.75 | MB | HD | 1 | 1.047198 |
| 0.2 | MB | HD | 1 | 0.463648 |
| 0.5 | MB | HD | 1 | 0.785398 |
| 0.6 | MB | HD | 1 | 0.886077 |
| 1 | MB | HD | 1 | 1.570796 |
| 1 | MB | HD | 1 | 1.570796 |
| 0.75 | MB | HD | 1 | 1.047198 |
| 0.588235 | MB | HD | 1 | 0.874098 |
| 0.285714 | MB | HD | 1 | 0.563943 |
| 0.333333 | MB | HD | 1 | 0.61548 |
| 1 | MB | HD | 1 | 1.570796 |
| 0.75 | MB | HD | 1 | 1.047198 |
| 0.555556 | MB | LD | 1 | 0.841069 |
| 1 | MB | LD | 1 | 1.570796 |
| 0.5 | MB | LD | 1 | 0.785398 |
| 0.666667 | MB | LD | 1 | 0.955317 |
| 0.75 | MB | LD | 1 | 1.047198 |
| 0.6 | MB | LD | 1 | 0.886077 |
| 0.714286 | MB | LD | 1 | 1.006854 |
| 1 | MB | LD | 1 | 1.570796 |
| 0.538462 | MB | LD | 1 | 0.823898 |
| 1 | MB | LD | 1 | 1.570796 |
| 1 | MB | LD | 1 | 1.570796 |
| 0.571429 | MB | LD | 1 | 0.857072 |
| 1 | MB | LD | 1 | 1.570796 |
| 1 | MB | LD | 1 | 1.570796 |
| 0.857143 | MB | LD | 1 | 1.1832 |
| 0.75 | MB | LD | 1 | 1.047198 |
| 0.75 | MB | LD | 1 | 1.047198 |
| 0.636364 | MB | LD | 1 | 0.923511 |
| 1 | MB | LD | 1 | 1.570796 |
| 0.888889 | MB | LD | 1 | 1.230959 |
| 1 | MB | LD | 1 | 1.570796 |
| 0.5 | MB | LD | 1 | 0.785398 |
| 0.857143 | MB | LD | 1 | 1.1832 |
| 1 | MB | LD | 1 | 1.570796 |
| 1 | MB | LD | 1 | 1.570796 |
| 0.875 | MB | LD | 1 | 1.209429 |
| 1 | MB | LD | 1 | 1.570796 |
| 1 | MB | LD | 1 | 1.570796 |
| 1 | MB | LD | 1 | 1.570796 |
| 0.2 | MB | LD | 1 | 0.463648 |
| 1 | MB | LD | 1 | 1.570796 |
| 0.75 | MB | LD | 1 | 1.047198 |
| 1 | MB | LD | 1 | 1.570796 |
| 1 | MB | LD | 1 | 1.570796 |
| 1 | MB | LD | 1 | 1.570796 |
| 0.777778 | MB | LD | 1 | 1.079914 |
| 0.875 | MB | LD | 1 | 1.209429 |
| 0.75 | MB | LD | 1 | 1.047198 |
| 1 | MB | LD | 1 | 1.570796 |
| 0.8 | MB | LD | 1 | 1.107149 |
| 0.666667 | MB | LD | 1 | 0.955317 |
| 0.4 | MB | LD | 1 | 0.684719 |
| 1 | MB | LD | 1 | 1.570796 |
| 1 | MB | LD | 1 | 1.570796 |
| 0.8 | MB | LD | 1 | 1.107149 |
| 0.909091 | MB | LD | 1 | 1.264519 |
| 1 | MB | LD | 1 | 1.570796 |
| 1 | MB | LD | 1 | 1.570796 |
| 1 | MB | LD | 1 | 1.570796 |
| 0.9 | MB | LD | 1 | 1.249046 |
| 0.875 | MB | LD | 1 | 1.209429 |
| 0.8 | MB | LD | 1 | 1.107149 |
| 0.2 | MB | LD | 1 | 0.463648 |
| 0.833333 | MB | LD | 1 | 1.150262 |
| 1 | MB | LD | 1 | 1.570796 |
| 1 | MB | LD | 1 | 1.570796 |
| 0.777778 | MB | LD | 1 | 1.079914 |
| 0.666667 | MB | LD | 1 | 0.955317 |
| 0.5 | MB | LD | 1 | 0.785398 |
| 0.6 | MB | LD | 2 | 0.886077 |
| 0.888889 | MB | LD | 2 | 1.230959 |
| 1 | MB | LD | 2 | 1.570796 |
| 0.571429 | MB | LD | 2 | 0.857072 |
| 0.5 | MB | LD | 2 | 0.785398 |
| 1 | MB | LD | 2 | 1.570796 |
| 0.833333 | MB | LD | 2 | 1.150262 |
| 0.714286 | MB | LD | 2 | 1.006854 |
| 1 | MB | LD | 2 | 1.570796 |
| 0.75 | MB | LD | 2 | 1.047198 |
| 0.75 | MB | LD | 2 | 1.047198 |
| 0.857143 | MB | LD | 2 | 1.1832 |
| 0.857143 | MB | LD | 2 | 1.1832 |
| 0.785714 | MB | LD | 2 | 1.089521 |
| 0.916667 | MB | LD | 2 | 1.277954 |
| 1 | MB | LD | 2 | 1.570796 |
| 1 | MB | LD | 2 | 1.570796 |
| 0.909091 | MB | LD | 2 | 1.264519 |
| 0.875 | MB | LD | 2 | 1.209429 |
| 0.818182 | MB | LD | 2 | 1.130286 |
| 0.833333 | MB | LD | 2 | 1.150262 |
| 1 | MB | LD | 2 | 1.570796 |
| 0.571429 | MB | LD | 2 | 0.857072 |
| 1 | MB | LD | 2 | 1.570796 |
| 0.777778 | MB | LD | 2 | 1.079914 |
| 0.833333 | MB | LD | 2 | 1.150262 |
| 1 | MB | LD | 2 | 1.570796 |
| 1 | MB | LD | 2 | 1.570796 |
| 1 | MB | LD | 2 | 1.570796 |
| 0.75 | MB | LD | 2 | 1.047198 |
| 0.75 | MB | LD | 2 | 1.047198 |
| 1 | MB | LD | 2 | 1.570796 |
| 1 | MB | LD | 2 | 1.570796 |
| 1 | MB | LD | 2 | 1.570796 |
| 0.666667 | MB | LD | 2 | 0.955317 |
| 1 | MB | LD | 2 | 1.570796 |
| 0.916667 | MB | LD | 2 | 1.277954 |
| 0.5 | MB | LD | 2 | 0.785398 |
| 1 | MB | LD | 2 | 1.570796 |
| 0.875 | MB | LD | 2 | 1.209429 |
| 0.875 | MB | LD | 2 | 1.209429 |
| 1 | MB | LD | 2 | 1.570796 |
| 0.631579 | MB | LD | 2 | 0.918545 |
| 1 | MB | LD | 2 | 1.570796 |
| 0.666667 | MB | LD | 2 | 0.955317 |
| 0.714286 | MB | LD | 2 | 1.006854 |
| 0.333333 | MB | LD | 2 | 0.61548 |
| 1 | MB | LD | 2 | 1.570796 |
| 0.6875 | MB | LD | 2 | 0.977597 |
| 1 | MB | LD | 2 | 1.570796 |
| 0.75 | MB | LD | 2 | 1.047198 |
| 1 | MB | LD | 2 | 1.570796 |
| 0.666667 | MB | LD | 2 | 0.955317 |
| 0.833333 | MB | LD | 2 | 1.150262 |
| 0.47619 | MB | LD | 2 | 0.76158 |
| 1 | MB | LD | 2 | 1.570796 |
| 1 | MB | LD | 2 | 1.570796 |
| 1 | MB | LD | 2 | 1.570796 |
| 1 | MB | LD | 2 | 1.570796 |
| 0.833333 | MB | LD | 2 | 1.150262 |
| 1 | MB | HD | 2 | 1.570796 |
| 1 | MB | HD | 2 | 1.570796 |
| 0.5 | MB | HD | 2 | 0.785398 |
| 1 | MB | HD | 2 | 1.570796 |
| 0.666667 | MB | HD | 2 | 0.955317 |
| 0.181818 | MB | HD | 2 | 0.440511 |
| 0.833333 | MB | HD | 2 | 1.150262 |
| 0.5 | MB | HD | 2 | 0.785398 |
| 0.75 | MB | HD | 2 | 1.047198 |
| 0.857143 | MB | HD | 2 | 1.1832 |
| 0.571429 | MB | HD | 2 | 0.857072 |
| 0.666667 | MB | HD | 2 | 0.955317 |
| 1 | MB | HD | 2 | 1.570796 |
| 1 | MB | HD | 2 | 1.570796 |
| 0.5 | MB | HD | 2 | 0.785398 |
| 0.777778 | MB | HD | 2 | 1.079914 |
| 0.666667 | MB | HD | 2 | 0.955317 |
| 0.75 | MB | HD | 2 | 1.047198 |
| 0.5 | MB | HD | 2 | 0.785398 |
| 0.571429 | MB | HD | 2 | 0.857072 |
| 0.3 | MB | HD | 2 | 0.57964 |
| 1 | MB | HD | 2 | 1.570796 |
| 0.5 | MB | HD | 2 | 0.785398 |
| 0.571429 | MB | HD | 2 | 0.857072 |
| 1 | MB | HD | 2 | 1.570796 |
| 1 | MB | HD | 2 | 1.570796 |
| 0.777778 | MB | HD | 2 | 1.079914 |
| 1 | MB | HD | 2 | 1.570796 |
| 0.166667 | MB | HD | 2 | 0.420534 |
| 0.875 | MB | HD | 2 | 1.209429 |
| 0.714286 | MB | HD | 2 | 1.006854 |
| 0.875 | MB | HD | 2 | 1.209429 |
| 0.125 | MB | HD | 2 | 0.361367 |
| 1 | MB | HD | 2 | 1.570796 |
| 0.833333 | MB | HD | 2 | 1.150262 |
| 0.625 | MB | HD | 2 | 0.911738 |
| 0.5 | MB | HD | 2 | 0.785398 |
| 0.5 | MB | HD | 2 | 0.785398 |
| 0.833333 | MB | HD | 2 | 1.150262 |
| 0.3 | MB | HD | 2 | 0.57964 |
| 0.6 | MB | HD | 2 | 0.886077 |
| 0.4 | MB | HD | 2 | 0.684719 |
| 0.571429 | MB | HD | 2 | 0.857072 |
| 0.666667 | MB | HD | 2 | 0.955317 |
| 1 | MB | HD | 2 | 1.570796 |
| 0.4 | MB | HD | 2 | 0.684719 |
| 1 | MB | HD | 2 | 1.570796 |
| 0.333333 | MB | HD | 2 | 0.61548 |
| 0.857143 | MB | HD | 2 | 1.1832 |
| 1 | MB | HD | 2 | 1.570796 |
| 0.285714 | MB | HD | 2 | 0.563943 |
| 1 | MB | HD | 2 | 1.570796 |
| 1 | MB | HD | 2 | 1.570796 |
| 1 | MB | HD | 2 | 1.570796 |
| 1 | MB | HD | 2 | 1.570796 |
| 1 | MB | HD | 2 | 1.570796 |
| 1 | MB | HD | 2 | 1.570796 |
| 1 | MB | HD | 2 | 1.570796 |
| 0.75 | MB | HD | 2 | 1.047198 |
| 0.75 | MB | HD | 2 | 1.047198 |
| 1 | MB | HD | 2 | 1.570796 |
| 1 | MB | HD | 2 | 1.570796 |
| 0.714286 | MB | HD | 2 | 1.006854 |
| 1 | MB | HD | 2 | 1.570796 |
| 0.444444 | MB | HD | 2 | 0.729728 |
| 1 | MB | HD | 2 | 1.570796 |
| 0.769231 | MB | HD | 2 | 1.069703 |
| 1 | MB | HD | 2 | 1.570796 |
| 0.5 | MB | HD | 2 | 0.785398 |
| 0.5 | MB | HD | 2 | 0.785398 |
| 0.75 | MB | HD | 2 | 1.047198 |
| 0.833333 | MB | HD | 2 | 1.150262 |
| 1 | MB | HD | 2 | 1.570796 |
| 0.666667 | MB | HD | 2 | 0.955317 |
| 1 | MB | HD | 2 | 1.570796 |
| 1 | MB | HD | 2 | 1.570796 |
| 0.714286 | MB | HD | 2 | 1.006854 |
| 0.857143 | MB | HD | 2 | 1.1832 |
| 1 | MB | HD | 2 | 1.570796 |
| 1 | MB | HD | 2 | 1.570796 |
| 0.846154 | MB | HD | 2 | 1.167739 |
| 0.166667 | MB | HD | 2 | 0.420534 |
| 0.166667 | MB | HD | 2 | 0.420534 |
| 0.888889 | MB | HD | 2 | 1.230959 |
| 0.5 | MB | HD | 2 | 0.785398 |
| 0.428571 | MB | HD | 2 | 0.713724 |
| 0.285714 | MB | HD | 2 | 0.563943 |
| 0.875 | MB | HD | 2 | 1.209429 |
| 0.888889 | MB | HD | 2 | 1.230959 |
| 0.571429 | MB | HD | 2 | 0.857072 |
| 0.9 | MB | HD | 2 | 1.249046 |
| 1 | MB | HD | 2 | 1.570796 |
| 0.666667 | MB | HD | 2 | 0.955317 |
| 0.857143 | MB | HD | 2 | 1.1832 |
| 0.666667 | MB | HD | 2 | 0.955317 |
| 1 | MB | HD | 3 | 1.570796 |
| 1 | MB | HD | 3 | 1.570796 |
| 1 | MB | HD | 3 | 1.570796 |
| 1 | MB | HD | 3 | 1.570796 |
| 1 | MB | HD | 3 | 1.570796 |
| 1 | MB | HD | 3 | 1.570796 |
| 1 | MB | HD | 3 | 1.570796 |
| 1 | MB | HD | 3 | 1.570796 |
| 1 | MB | HD | 3 | 1.570796 |
| 1 | MB | HD | 3 | 1.570796 |
| 1 | MB | HD | 3 | 1.570796 |
| 1 | MB | HD | 3 | 1.570796 |
| 1 | MB | HD | 3 | 1.570796 |
| 1 | MB | HD | 3 | 1.570796 |
| 1 | MB | HD | 3 | 1.570796 |
| 1 | MB | HD | 3 | 1.570796 |
| 1 | MB | HD | 3 | 1.570796 |
| 1 | MB | LD | 3 | 1.570796 |
| 1 | MB | LD | 3 | 1.570796 |
| 1 | MB | LD | 3 | 1.570796 |
| 1 | MB | LD | 3 | 1.570796 |
| 1 | MB | LD | 3 | 1.570796 |
| 1 | MB | LD | 3 | 1.570796 |
| 1 | MB | LD | 3 | 1.570796 |
| 1 | MB | LD | 3 | 1.570796 |
| 0.956522 | MB | LD | 3 | 1.360741 |
| 0.952381 | MB | HD | 3 | 1.350808 |
| 0.944444 | MB | LD | 3 | 1.332855 |
| 0.941176 | MB | LD | 3 | 1.325818 |
| 0.941176 | MB | LD | 3 | 1.325818 |
| 0.923077 | MB | HD | 3 | 1.289761 |
| 0.923077 | MB | LD | 3 | 1.289761 |
| 0.916667 | MB | LD | 3 | 1.277954 |
| 0.909091 | MB | HD | 3 | 1.264519 |
| 0.909091 | MB | LD | 3 | 1.264519 |
| 0.909091 | MB | LD | 3 | 1.264519 |
| 0.909091 | MB | LD | 3 | 1.264519 |
| 0.904762 | MB | HD | 3 | 1.257068 |
| 0.888889 | MB | HD | 3 | 1.230959 |
| 0.888889 | MB | HD | 3 | 1.230959 |
| 0.888889 | MB | HD | 3 | 1.230959 |
| 0.888889 | MB | LD | 3 | 1.230959 |
| 0.888889 | MB | LD | 3 | 1.230959 |
| 0.875 | MB | HD | 3 | 1.209429 |
| 0.875 | MB | HD | 3 | 1.209429 |
| 0.875 | MB | HD | 3 | 1.209429 |
| 0.875 | MB | HD | 3 | 1.209429 |
| 0.875 | MB | HD | 3 | 1.209429 |
| 0.866667 | MB | HD | 3 | 1.197004 |
| 0.866667 | MB | LD | 3 | 1.197004 |
| 0.857143 | MB | HD | 3 | 1.1832 |
| 0.857143 | MB | HD | 3 | 1.1832 |
| 0.857143 | MB | LD | 3 | 1.1832 |
| 0.85 | MB | LD | 3 | 1.173097 |
| 0.846154 | MB | HD | 3 | 1.167739 |
| 0.846154 | MB | HD | 3 | 1.167739 |
| 0.846154 | MB | HD | 3 | 1.167739 |
| 0.846154 | MB | HD | 3 | 1.167739 |
| 0.833333 | MB | HD | 3 | 1.150262 |
| 0.833333 | MB | HD | 3 | 1.150262 |
| 0.823529 | MB | HD | 3 | 1.137258 |
| 0.818182 | MB | HD | 3 | 1.130286 |
| 0.818182 | MB | HD | 3 | 1.130286 |
| 0.8 | MB | HD | 3 | 1.107149 |
| 0.785714 | MB | HD | 3 | 1.089521 |
| 0.769231 | MB | HD | 3 | 1.069703 |
| 0.769231 | MB | LD | 3 | 1.069703 |
| 0.764706 | MB | HD | 3 | 1.064352 |
| 0.75 | MB | HD | 3 | 1.047198 |
| 0.75 | MB | HD | 3 | 1.047198 |
| 0.75 | MB | HD | 3 | 1.047198 |
| 0.75 | MB | HD | 3 | 1.047198 |
| 0.75 | MB | HD | 3 | 1.047198 |
| 0.75 | MB | LD | 3 | 1.047198 |
| 0.733333 | MB | LD | 3 | 1.028157 |
| 0.727273 | MB | HD | 3 | 1.021329 |
| 0.727273 | MB | LD | 3 | 1.021329 |
| 0.7 | MB | HD | 3 | 0.991157 |
| 0.695652 | MB | HD | 3 | 0.986422 |
| 0.692308 | MB | HD | 3 | 0.982794 |
| 0.666667 | MB | HD | 3 | 0.955317 |
| 0.666667 | MB | HD | 3 | 0.955317 |
| 0.666667 | MB | HD | 3 | 0.955317 |
| 0.666667 | MB | HD | 3 | 0.955317 |
| 0.666667 | MB | HD | 3 | 0.955317 |
| 0.666667 | MB | HD | 3 | 0.955317 |
| 0.666667 | MB | HD | 3 | 0.955317 |
| 0.666667 | MB | HD | 3 | 0.955317 |
| 0.666667 | MB | HD | 3 | 0.955317 |
| 0.666667 | MB | HD | 3 | 0.955317 |
| 0.666667 | MB | HD | 3 | 0.955317 |
| 0.666667 | MB | LD | 3 | 0.955317 |
| 0.666667 | MB | LD | 3 | 0.955317 |
| 0.666667 | MB | LD | 3 | 0.955317 |
| 0.647059 | MB | HD | 3 | 0.934664 |
| 0.642857 | MB | HD | 3 | 0.930274 |
| 0.636364 | MB | LD | 3 | 0.923511 |
| 0.625 | MB | HD | 3 | 0.911738 |
| 0.615385 | MB | HD | 3 | 0.901832 |
| 0.6 | MB | HD | 3 | 0.886077 |
| 0.6 | MB | LD | 3 | 0.886077 |
| 0.588235 | MB | HD | 3 | 0.874098 |
| 0.583333 | MB | HD | 3 | 0.869122 |
| 0.571429 | MB | HD | 3 | 0.857072 |
| 0.555556 | MB | HD | 3 | 0.841069 |
| 0.545455 | MB | LD | 3 | 0.830916 |
| 0.538462 | MB | HD | 3 | 0.823898 |
| 0.533333 | MB | HD | 3 | 0.818756 |
| 0.533333 | MB | LD | 3 | 0.818756 |
| 0.53125 | MB | LD | 3 | 0.816669 |
| 0.526316 | MB | HD | 3 | 0.811726 |
| 0.5 | MB | HD | 3 | 0.785398 |
| 0.5 | MB | HD | 3 | 0.785398 |
| 0.5 | MB | HD | 3 | 0.785398 |
| 0.5 | MB | LD | 3 | 0.785398 |
| 0.5 | MB | LD | 3 | 0.785398 |
| 0.444444 | MB | HD | 3 | 0.729728 |
| 0.411765 | MB | HD | 3 | 0.696698 |
| 0.4 | MB | HD | 3 | 0.684719 |
| 0.4 | MB | HD | 3 | 0.684719 |
| 0.4 | MB | HD | 3 | 0.684719 |
| 0.4 | MB | LD | 3 | 0.684719 |
| 0.384615 | MB | LD | 3 | 0.668964 |
| 0.375 | MB | HD | 3 | 0.659058 |
| 0.352941 | MB | LD | 3 | 0.636132 |
| 0.333333 | MB | HD | 3 | 0.61548 |
| 0.333333 | MB | HD | 3 | 0.61548 |
| 0.285714 | MB | HD | 3 | 0.563943 |
| 0.266667 | MB | LD | 3 | 0.542639 |
| 0.25 | MB | HD | 3 | 0.523599 |
| 0.230769 | MB | LD | 3 | 0.501093 |
| 0.214286 | MB | HD | 3 | 0.481275 |
| 0.1875 | MB | HD | 3 | 0.447832 |
| 0.166667 | MB | HD | 3 | 0.420534 |
| 0.166667 | MB | HD | 3 | 0.420534 |
| 0.166667 | MB | LD | 3 | 0.420534 |
| 0.157895 | MB | LD | 3 | 0.408638 |
| 0.076923 | MB | HD | 3 | 0.281035 |
| 0.076923 | MB | LD | 3 | 0.281035 |
| 0.0625 | MB | LD | 3 | 0.25268 |
| 1 | MB | LD | 4 | 1.570796 |
| 1 | MB | LD | 4 | 1.570796 |
| 1 | MB | LD | 4 | 1.570796 |
| 1 | MB | LD | 4 | 1.570796 |
| 1 | MB | LD | 4 | 1.570796 |
| 1 | MB | LD | 4 | 1.570796 |
| 1 | MB | LD | 4 | 1.570796 |
| 1 | MB | LD | 4 | 1.570796 |
| 1 | MB | LD | 4 | 1.570796 |
| 1 | MB | LD | 4 | 1.570796 |
| 1 | MB | LD | 4 | 1.570796 |
| 1 | MB | LD | 4 | 1.570796 |
| 1 | MB | LD | 4 | 1.570796 |
| 1 | MB | LD | 4 | 1.570796 |
| 1 | MB | LD | 4 | 1.570796 |
| 1 | MB | LD | 4 | 1.570796 |
| 1 | MB | LD | 4 | 1.570796 |
| 1 | MB | LD | 4 | 1.570796 |
| 1 | MB | LD | 4 | 1.570796 |
| 1 | MB | LD | 4 | 1.570796 |
| 1 | MB | LD | 4 | 1.570796 |
| 1 | MB | LD | 4 | 1.570796 |
| 1 | MB | LD | 4 | 1.570796 |
| 1 | MB | LD | 4 | 1.570796 |
| 1 | MB | LD | 4 | 1.570796 |
| 1 | MB | LD | 4 | 1.570796 |
| 1 | MB | LD | 4 | 1.570796 |
| 1 | MB | LD | 4 | 1.570796 |
| 1 | MB | LD | 4 | 1.570796 |
| 1 | MB | HD | 4 | 1.570796 |
| 1 | MB | HD | 4 | 1.570796 |
| 1 | MB | HD | 4 | 1.570796 |
| 1 | MB | HD | 4 | 1.570796 |
| 1 | MB | HD | 4 | 1.570796 |
| 1 | MB | HD | 4 | 1.570796 |
| 1 | MB | HD | 4 | 1.570796 |
| 1 | MB | HD | 4 | 1.570796 |
| 1 | MB | HD | 4 | 1.570796 |
| 1 | MB | HD | 4 | 1.570796 |
| 1 | MB | HD | 4 | 1.570796 |
| 1 | MB | HD | 4 | 1.570796 |
| 1 | MB | HD | 4 | 1.570796 |
| 1 | MB | HD | 4 | 1.570796 |
| 1 | MB | HD | 4 | 1.570796 |
| 1 | MB | HD | 4 | 1.570796 |
| 1 | MB | HD | 4 | 1.570796 |
| 1 | MB | HD | 4 | 1.570796 |
| 1 | MB | HD | 4 | 1.570796 |
| 1 | MB | HD | 4 | 1.570796 |
| 1 | MB | HD | 4 | 1.570796 |
| 1 | MB | HD | 4 | 1.570796 |
| 1 | MB | HD | 4 | 1.570796 |
| 1 | MB | HD | 4 | 1.570796 |
| 1 | MB | HD | 4 | 1.570796 |
| 1 | MB | HD | 4 | 1.570796 |
| 1 | MB | HD | 4 | 1.570796 |
| 1 | MB | HD | 4 | 1.570796 |
| 1 | MB | HD | 4 | 1.570796 |
| 1 | MB | HD | 4 | 1.570796 |
| 0.931034 | MB | LD | 4 | 1.305067 |
| 0.928571 | MB | LD | 4 | 1.300247 |
| 0.928571 | MB | LD | 4 | 1.300247 |
| 0.923077 | MB | HD | 4 | 1.289761 |
| 0.916667 | MB | LD | 4 | 1.277954 |
| 0.909091 | MB | LD | 4 | 1.264519 |
| 0.909091 | MB | HD | 4 | 1.264519 |
| 0.9 | MB | LD | 4 | 1.249046 |
| 0.9 | MB | LD | 4 | 1.249046 |
| 0.9 | MB | HD | 4 | 1.249046 |
| 0.9 | MB | HD | 4 | 1.249046 |
| 0.9 | MB | HD | 4 | 1.249046 |
| 0.888889 | MB | LD | 4 | 1.230959 |
| 0.888889 | MB | HD | 4 | 1.230959 |
| 0.888889 | MB | HD | 4 | 1.230959 |
| 0.888889 | MB | HD | 4 | 1.230959 |
| 0.888889 | MB | HD | 4 | 1.230959 |
| 0.882353 | MB | HD | 4 | 1.220691 |
| 0.875 | MB | LD | 4 | 1.209429 |
| 0.875 | MB | LD | 4 | 1.209429 |
| 0.875 | MB | HD | 4 | 1.209429 |
| 0.875 | MB | HD | 4 | 1.209429 |
| 0.875 | MB | HD | 4 | 1.209429 |
| 0.862069 | MB | LD | 4 | 1.19029 |
| 0.857143 | MB | LD | 4 | 1.1832 |
| 0.857143 | MB | HD | 4 | 1.1832 |
| 0.857143 | MB | HD | 4 | 1.1832 |
| 0.857143 | MB | HD | 4 | 1.1832 |
| 0.857143 | MB | HD | 4 | 1.1832 |
| 0.857143 | MB | HD | 4 | 1.1832 |
| 0.857143 | MB | HD | 4 | 1.1832 |
| 0.857143 | MB | HD | 4 | 1.1832 |
| 0.851064 | MB | LD | 4 | 1.174589 |
| 0.846154 | MB | LD | 4 | 1.167739 |
| 0.846154 | MB | HD | 4 | 1.167739 |
| 0.833333 | MB | LD | 4 | 1.150262 |
| 0.833333 | MB | LD | 4 | 1.150262 |
| 0.833333 | MB | LD | 4 | 1.150262 |
| 0.833333 | MB | HD | 4 | 1.150262 |
| 0.833333 | MB | HD | 4 | 1.150262 |
| 0.833333 | MB | HD | 4 | 1.150262 |
| 0.833333 | MB | HD | 4 | 1.150262 |
| 0.833333 | MB | HD | 4 | 1.150262 |
| 0.833333 | MB | HD | 4 | 1.150262 |
| 0.833333 | MB | HD | 4 | 1.150262 |
| 0.833333 | MB | HD | 4 | 1.150262 |
| 0.818182 | MB | LD | 4 | 1.130286 |
| 0.818182 | MB | LD | 4 | 1.130286 |
| 0.818182 | MB | HD | 4 | 1.130286 |
| 0.8 | MB | LD | 4 | 1.107149 |
| 0.8 | MB | HD | 4 | 1.107149 |
| 0.8 | MB | HD | 4 | 1.107149 |
| 0.8 | MB | HD | 4 | 1.107149 |
| 0.8 | MB | HD | 4 | 1.107149 |
| 0.769231 | MB | HD | 4 | 1.069703 |
| 0.75 | MB | LD | 4 | 1.047198 |
| 0.75 | MB | LD | 4 | 1.047198 |
| 0.75 | MB | HD | 4 | 1.047198 |
| 0.75 | MB | HD | 4 | 1.047198 |
| 0.75 | MB | HD | 4 | 1.047198 |
| 0.75 | MB | HD | 4 | 1.047198 |
| 0.75 | MB | HD | 4 | 1.047198 |
| 0.75 | MB | HD | 4 | 1.047198 |
| 0.71875 | MB | HD | 4 | 1.011806 |
| 0.714286 | MB | HD | 4 | 1.006854 |
| 0.682927 | MB | LD | 4 | 0.972673 |
| 0.666667 | MB | HD | 4 | 0.955317 |
| 0.666667 | MB | HD | 4 | 0.955317 |
| 0.666667 | MB | HD | 4 | 0.955317 |
| 0.666667 | MB | HD | 4 | 0.955317 |
| 0.636364 | MB | HD | 4 | 0.923511 |
| 0.625 | MB | HD | 4 | 0.911738 |
| 0.625 | MB | HD | 4 | 0.911738 |
| 0.6 | MB | HD | 4 | 0.886077 |
| 0.6 | MB | HD | 4 | 0.886077 |
| 0.571429 | MB | HD | 4 | 0.857072 |
| 0.571429 | MB | HD | 4 | 0.857072 |
| 0.571429 | MB | HD | 4 | 0.857072 |
| 0.5 | MB | HD | 4 | 0.785398 |
| 0.454545 | MB | LD | 4 | 0.739881 |
| 0.428571 | MB | LD | 4 | 0.713724 |
| 0.428571 | MB | HD | 4 | 0.713724 |
| 0.4 | MB | HD | 4 | 0.684719 |
| 0.375 | MB | LD | 4 | 0.659058 |
| 0.375 | MB | LD | 4 | 0.659058 |
| 0.333333 | MB | HD | 4 | 0.61548 |
| 0.3 | MB | LD | 4 | 0.57964 |
| 0.285714 | MB | HD | 4 | 0.563943 |
| 0.222222 | MB | HD | 4 | 0.490883 |
| 0.2 | MB | HD | 4 | 0.463648 |
| 0.142857 | MB | HD | 4 | 0.387597 |
| 0.083333 | MB | HD | 4 | 0.292843 |

Supplementary table 3: 9 day old P2= Untransformed raw data values of P2; Selection: MCU= Populations adapted to larval crowding, MB: Control populations; Treatment= HD= High larval density, LD= Low larval density; Block= Replicate populations of MCU and MB; P2 (arcsin sqrt) Arcsin square root transformed values P2 values

| p2 | Treatment | Selection | Block | Arc_sqrt_P2 |
| --- | --- | --- | --- | --- |
| 0.153846 | HD | MB | 1 | 0.403057 |
| 0.190476 | HD | MB | 1 | 0.451633 |
| 0.230769 | HD | MB | 4 | 0.501093 |
| 0.25 | HD | MB | 3 | 0.523599 |
| 0.25 | HD | MB | 4 | 0.523599 |
| 0.266667 | HD | MB | 3 | 0.542639 |
| 0.285714 | HD | MB | 1 | 0.563943 |
| 0.285714 | HD | MB | 2 | 0.563943 |
| 0.333333 | HD | MB | 3 | 0.61548 |
| 0.357143 | HD | MB | 1 | 0.640522 |
| 0.4 | HD | MB | 2 | 0.684719 |
| 0.4 | HD | MB | 4 | 0.684719 |
| 0.454545 | HD | MB | 1 | 0.739881 |
| 0.454545 | HD | MB | 1 | 0.739881 |
| 0.454545 | HD | MB | 4 | 0.739881 |
| 0.5 | HD | MB | 1 | 0.785398 |
| 0.5 | HD | MB | 1 | 0.785398 |
| 0.5 | HD | MB | 1 | 0.785398 |
| 0.5 | HD | MB | 2 | 0.785398 |
| 0.5 | HD | MB | 2 | 0.785398 |
| 0.5 | HD | MB | 3 | 0.785398 |
| 0.5 | HD | MB | 3 | 0.785398 |
| 0.5 | HD | MB | 3 | 0.785398 |
| 0.5 | HD | MB | 4 | 0.785398 |
| 0.5 | HD | MB | 4 | 0.785398 |
| 0.538462 | HD | MB | 3 | 0.823898 |
| 0.555556 | HD | MB | 4 | 0.841069 |
| 0.565217 | HD | MB | 1 | 0.850802 |
| 0.571429 | HD | MB | 1 | 0.857072 |
| 0.571429 | HD | MB | 2 | 0.857072 |
| 0.571429 | HD | MB | 2 | 0.857072 |
| 0.571429 | HD | MB | 3 | 0.857072 |
| 0.571429 | HD | MB | 3 | 0.857072 |
| 0.571429 | HD | MB | 3 | 0.857072 |
| 0.571429 | HD | MB | 3 | 0.857072 |
| 0.571429 | HD | MB | 4 | 0.857072 |
| 0.571429 | HD | MB | 4 | 0.857072 |
| 0.583333 | HD | MB | 1 | 0.869122 |
| 0.583333 | HD | MB | 1 | 0.869122 |
| 0.6 | HD | MB | 2 | 0.886077 |
| 0.6 | HD | MB | 2 | 0.886077 |
| 0.6 | HD | MB | 4 | 0.886077 |
| 0.615385 | HD | MB | 1 | 0.901832 |
| 0.615385 | HD | MB | 2 | 0.901832 |
| 0.625 | HD | MB | 3 | 0.911738 |
| 0.625 | HD | MB | 3 | 0.911738 |
| 0.625 | HD | MB | 4 | 0.911738 |
| 0.625 | HD | MB | 4 | 0.911738 |
| 0.625 | HD | MB | 4 | 0.911738 |
| 0.642857 | HD | MB | 3 | 0.930274 |
| 0.642857 | HD | MB | 4 | 0.930274 |
| 0.647059 | HD | MB | 2 | 0.934664 |
| 0.647059 | HD | MB | 3 | 0.934664 |
| 0.666667 | HD | MB | 1 | 0.955317 |
| 0.666667 | HD | MB | 1 | 0.955317 |
| 0.666667 | HD | MB | 1 | 0.955317 |
| 0.666667 | HD | MB | 1 | 0.955317 |
| 0.666667 | HD | MB | 2 | 0.955317 |
| 0.666667 | HD | MB | 2 | 0.955317 |
| 0.666667 | HD | MB | 2 | 0.955317 |
| 0.666667 | HD | MB | 3 | 0.955317 |
| 0.666667 | HD | MB | 3 | 0.955317 |
| 0.666667 | HD | MB | 3 | 0.955317 |
| 0.666667 | HD | MB | 4 | 0.955317 |
| 0.666667 | HD | MB | 4 | 0.955317 |
| 0.666667 | HD | MB | 4 | 0.955317 |
| 0.6875 | HD | MB | 2 | 0.977597 |
| 0.6875 | HD | MB | 3 | 0.977597 |
| 0.7 | HD | MB | 1 | 0.991157 |
| 0.7 | HD | MB | 3 | 0.991157 |
| 0.714286 | HD | MB | 1 | 1.006854 |
| 0.714286 | HD | MB | 3 | 1.006854 |
| 0.714286 | HD | MB | 3 | 1.006854 |
| 0.727273 | HD | MB | 1 | 1.021329 |
| 0.733333 | HD | MB | 3 | 1.028157 |
| 0.75 | HD | MB | 1 | 1.047198 |
| 0.75 | HD | MB | 1 | 1.047198 |
| 0.75 | HD | MB | 1 | 1.047198 |
| 0.75 | HD | MB | 2 | 1.047198 |
| 0.75 | HD | MB | 2 | 1.047198 |
| 0.75 | HD | MB | 2 | 1.047198 |
| 0.75 | HD | MB | 2 | 1.047198 |
| 0.75 | HD | MB | 2 | 1.047198 |
| 0.75 | HD | MB | 3 | 1.047198 |
| 0.75 | HD | MB | 3 | 1.047198 |
| 0.75 | HD | MB | 3 | 1.047198 |
| 0.75 | HD | MB | 3 | 1.047198 |
| 0.75 | HD | MB | 3 | 1.047198 |
| 0.75 | HD | MB | 3 | 1.047198 |
| 0.764706 | HD | MB | 3 | 1.064352 |
| 0.769231 | HD | MB | 1 | 1.069703 |
| 0.769231 | HD | MB | 1 | 1.069703 |
| 0.777778 | HD | MB | 1 | 1.079914 |
| 0.777778 | HD | MB | 2 | 1.079914 |
| 0.777778 | HD | MB | 4 | 1.079914 |
| 0.785714 | HD | MB | 1 | 1.089521 |
| 0.785714 | HD | MB | 4 | 1.089521 |
| 0.785714 | HD | MB | 4 | 1.089521 |
| 0.8 | HD | MB | 1 | 1.107149 |
| 0.8 | HD | MB | 2 | 1.107149 |
| 0.8 | HD | MB | 2 | 1.107149 |
| 0.8 | HD | MB | 2 | 1.107149 |
| 0.8 | HD | MB | 3 | 1.107149 |
| 0.8 | HD | MB | 3 | 1.107149 |
| 0.8 | HD | MB | 3 | 1.107149 |
| 0.8 | HD | MB | 3 | 1.107149 |
| 0.8 | HD | MB | 3 | 1.107149 |
| 0.8 | HD | MB | 3 | 1.107149 |
| 0.8 | HD | MB | 4 | 1.107149 |
| 0.8 | HD | MB | 4 | 1.107149 |
| 0.809524 | HD | MB | 1 | 1.119163 |
| 0.8125 | HD | MB | 3 | 1.122964 |
| 0.818182 | HD | MB | 1 | 1.130286 |
| 0.818182 | HD | MB | 1 | 1.130286 |
| 0.818182 | HD | MB | 2 | 1.130286 |
| 0.818182 | HD | MB | 3 | 1.130286 |
| 0.833333 | HD | MB | 3 | 1.150262 |
| 0.833333 | HD | MB | 4 | 1.150262 |
| 0.846154 | HD | MB | 2 | 1.167739 |
| 0.846154 | HD | MB | 2 | 1.167739 |
| 0.857143 | HD | MB | 1 | 1.1832 |
| 0.857143 | HD | MB | 1 | 1.1832 |
| 0.857143 | HD | MB | 1 | 1.1832 |
| 0.857143 | HD | MB | 2 | 1.1832 |
| 0.857143 | HD | MB | 2 | 1.1832 |
| 0.857143 | HD | MB | 4 | 1.1832 |
| 0.857143 | HD | MB | 4 | 1.1832 |
| 0.869565 | HD | MB | 4 | 1.201287 |
| 0.875 | HD | MB | 1 | 1.209429 |
| 0.875 | HD | MB | 1 | 1.209429 |
| 0.875 | HD | MB | 1 | 1.209429 |
| 0.875 | HD | MB | 1 | 1.209429 |
| 0.875 | HD | MB | 2 | 1.209429 |
| 0.875 | HD | MB | 2 | 1.209429 |
| 0.875 | HD | MB | 2 | 1.209429 |
| 0.875 | HD | MB | 3 | 1.209429 |
| 0.875 | HD | MB | 3 | 1.209429 |
| 0.875 | HD | MB | 4 | 1.209429 |
| 0.875 | HD | MB | 4 | 1.209429 |
| 0.888889 | HD | MB | 1 | 1.230959 |
| 0.888889 | HD | MB | 1 | 1.230959 |
| 0.888889 | HD | MB | 2 | 1.230959 |
| 0.888889 | HD | MB | 3 | 1.230959 |
| 0.888889 | HD | MB | 4 | 1.230959 |
| 0.888889 | HD | MB | 4 | 1.230959 |
| 0.894737 | HD | MB | 1 | 1.240374 |
| 0.9 | HD | MB | 2 | 1.249046 |
| 0.9 | HD | MB | 3 | 1.249046 |
| 0.9 | HD | MB | 3 | 1.249046 |
| 0.9 | HD | MB | 3 | 1.249046 |
| 0.9 | HD | MB | 3 | 1.249046 |
| 0.9 | HD | MB | 3 | 1.249046 |
| 0.9 | HD | MB | 4 | 1.249046 |
| 0.909091 | HD | MB | 2 | 1.264519 |
| 0.909091 | HD | MB | 2 | 1.264519 |
| 0.909091 | HD | MB | 3 | 1.264519 |
| 0.909091 | HD | MB | 3 | 1.264519 |
| 0.909091 | HD | MB | 4 | 1.264519 |
| 0.909091 | HD | MB | 4 | 1.264519 |
| 0.913043 | HD | MB | 3 | 1.271462 |
| 0.916667 | HD | MB | 2 | 1.277954 |
| 0.916667 | HD | MB | 3 | 1.277954 |
| 0.923077 | HD | MB | 1 | 1.289761 |
| 0.923077 | HD | MB | 1 | 1.289761 |
| 0.928571 | HD | MB | 1 | 1.300247 |
| 0.928571 | HD | MB | 1 | 1.300247 |
| 0.928571 | HD | MB | 1 | 1.300247 |
| 0.933333 | HD | MB | 1 | 1.309639 |
| 0.9375 | HD | MB | 4 | 1.318116 |
| 0.9375 | HD | MB | 4 | 1.318116 |
| 0.954545 | HD | MB | 2 | 1.355946 |
| 0.958333 | HD | MB | 1 | 1.365227 |
| 1 | HD | MB | 1 | 1.570796 |
| 1 | HD | MB | 1 | 1.570796 |
| 1 | HD | MB | 1 | 1.570796 |
| 1 | HD | MB | 1 | 1.570796 |
| 1 | HD | MB | 1 | 1.570796 |
| 1 | HD | MB | 1 | 1.570796 |
| 1 | HD | MB | 1 | 1.570796 |
| 1 | HD | MB | 1 | 1.570796 |
| 1 | HD | MB | 1 | 1.570796 |
| 1 | HD | MB | 1 | 1.570796 |
| 1 | HD | MB | 1 | 1.570796 |
| 1 | HD | MB | 1 | 1.570796 |
| 1 | HD | MB | 1 | 1.570796 |
| 1 | HD | MB | 1 | 1.570796 |
| 1 | HD | MB | 1 | 1.570796 |
| 1 | HD | MB | 1 | 1.570796 |
| 1 | HD | MB | 1 | 1.570796 |
| 1 | HD | MB | 2 | 1.570796 |
| 1 | HD | MB | 2 | 1.570796 |
| 1 | HD | MB | 2 | 1.570796 |
| 1 | HD | MB | 2 | 1.570796 |
| 1 | HD | MB | 2 | 1.570796 |
| 1 | HD | MB | 2 | 1.570796 |
| 1 | HD | MB | 2 | 1.570796 |
| 1 | HD | MB | 2 | 1.570796 |
| 1 | HD | MB | 2 | 1.570796 |
| 1 | HD | MB | 2 | 1.570796 |
| 1 | HD | MB | 2 | 1.570796 |
| 1 | HD | MB | 2 | 1.570796 |
| 1 | HD | MB | 2 | 1.570796 |
| 1 | HD | MB | 2 | 1.570796 |
| 1 | HD | MB | 2 | 1.570796 |
| 1 | HD | MB | 2 | 1.570796 |
| 1 | HD | MB | 2 | 1.570796 |
| 1 | HD | MB | 2 | 1.570796 |
| 1 | HD | MB | 2 | 1.570796 |
| 1 | HD | MB | 2 | 1.570796 |
| 1 | HD | MB | 2 | 1.570796 |
| 1 | HD | MB | 2 | 1.570796 |
| 1 | HD | MB | 2 | 1.570796 |
| 1 | HD | MB | 2 | 1.570796 |
| 1 | HD | MB | 2 | 1.570796 |
| 1 | HD | MB | 2 | 1.570796 |
| 1 | HD | MB | 2 | 1.570796 |
| 1 | HD | MB | 3 | 1.570796 |
| 1 | HD | MB | 3 | 1.570796 |
| 1 | HD | MB | 3 | 1.570796 |
| 1 | HD | MB | 3 | 1.570796 |
| 1 | HD | MB | 3 | 1.570796 |
| 1 | HD | MB | 3 | 1.570796 |
| 1 | HD | MB | 3 | 1.570796 |
| 1 | HD | MB | 3 | 1.570796 |
| 1 | HD | MB | 3 | 1.570796 |
| 1 | HD | MB | 3 | 1.570796 |
| 1 | HD | MB | 3 | 1.570796 |
| 1 | HD | MB | 3 | 1.570796 |
| 1 | HD | MB | 3 | 1.570796 |
| 1 | HD | MB | 3 | 1.570796 |
| 1 | HD | MB | 3 | 1.570796 |
| 1 | HD | MB | 3 | 1.570796 |
| 1 | HD | MB | 3 | 1.570796 |
| 1 | HD | MB | 3 | 1.570796 |
| 1 | HD | MB | 4 | 1.570796 |
| 1 | HD | MB | 4 | 1.570796 |
| 1 | HD | MB | 4 | 1.570796 |
| 1 | HD | MB | 4 | 1.570796 |
| 1 | HD | MB | 4 | 1.570796 |
| 1 | HD | MB | 4 | 1.570796 |
| 1 | HD | MB | 4 | 1.570796 |
| 1 | HD | MB | 4 | 1.570796 |
| 1 | HD | MB | 4 | 1.570796 |
| 1 | HD | MB | 4 | 1.570796 |
| 1 | HD | MB | 4 | 1.570796 |
| 1 | HD | MB | 4 | 1.570796 |
| 0.1 | LD | MB | 3 | 0.321751 |
| 0.181818 | LD | MB | 2 | 0.440511 |
| 0.352941 | LD | MB | 3 | 0.636132 |
| 0.428571 | LD | MB | 3 | 0.713724 |
| 0.444444 | LD | MB | 4 | 0.729728 |
| 0.454545 | LD | MB | 1 | 0.739881 |
| 0.5 | LD | MB | 2 | 0.785398 |
| 0.5 | LD | MB | 3 | 0.785398 |
| 0.52 | LD | MB | 4 | 0.805404 |
| 0.533333 | LD | MB | 3 | 0.818756 |
| 0.571429 | LD | MB | 3 | 0.857072 |
| 0.571429 | LD | MB | 4 | 0.857072 |
| 0.6 | LD | MB | 2 | 0.886077 |
| 0.6 | LD | MB | 4 | 0.886077 |
| 0.625 | LD | MB | 3 | 0.911738 |
| 0.625 | LD | MB | 4 | 0.911738 |
| 0.642857 | LD | MB | 2 | 0.930274 |
| 0.666667 | LD | MB | 2 | 0.955317 |
| 0.666667 | LD | MB | 2 | 0.955317 |
| 0.666667 | LD | MB | 3 | 0.955317 |
| 0.666667 | LD | MB | 3 | 0.955317 |
| 0.6875 | LD | MB | 1 | 0.977597 |
| 0.692308 | LD | MB | 1 | 0.982794 |
| 0.692308 | LD | MB | 3 | 0.982794 |
| 0.7 | LD | MB | 4 | 0.991157 |
| 0.714286 | LD | MB | 3 | 1.006854 |
| 0.714286 | LD | MB | 4 | 1.006854 |
| 0.714286 | LD | MB | 4 | 1.006854 |
| 0.75 | LD | MB | 2 | 1.047198 |
| 0.75 | LD | MB | 4 | 1.047198 |
| 0.75 | LD | MB | 4 | 1.047198 |
| 0.764706 | LD | MB | 1 | 1.064352 |
| 0.777778 | LD | MB | 2 | 1.079914 |
| 0.777778 | LD | MB | 3 | 1.079914 |
| 0.785714 | LD | MB | 1 | 1.089521 |
| 0.8 | LD | MB | 2 | 1.107149 |
| 0.8 | LD | MB | 4 | 1.107149 |
| 0.818182 | LD | MB | 3 | 1.130286 |
| 0.818182 | LD | MB | 3 | 1.130286 |
| 0.823529 | LD | MB | 3 | 1.137258 |
| 0.842105 | LD | MB | 4 | 1.162158 |
| 0.85 | LD | MB | 3 | 1.173097 |
| 0.857143 | LD | MB | 3 | 1.1832 |
| 0.866667 | LD | MB | 3 | 1.197004 |
| 0.875 | LD | MB | 3 | 1.209429 |
| 0.875 | LD | MB | 3 | 1.209429 |
| 0.875 | LD | MB | 4 | 1.209429 |
| 0.882353 | LD | MB | 3 | 1.220691 |
| 0.882353 | LD | MB | 3 | 1.220691 |
| 0.888889 | LD | MB | 2 | 1.230959 |
| 0.888889 | LD | MB | 2 | 1.230959 |
| 0.888889 | LD | MB | 4 | 1.230959 |
| 0.909091 | LD | MB | 2 | 1.264519 |
| 0.909091 | LD | MB | 4 | 1.264519 |
| 0.916667 | LD | MB | 4 | 1.277954 |
| 0.916667 | LD | MB | 4 | 1.277954 |
| 0.923077 | LD | MB | 2 | 1.289761 |
| 0.923077 | LD | MB | 4 | 1.289761 |
| 0.933333 | LD | MB | 1 | 1.309639 |
| 0.933333 | LD | MB | 2 | 1.309639 |
| 0.933333 | LD | MB | 2 | 1.309639 |
| 0.944444 | LD | MB | 1 | 1.332855 |
| 0.944444 | LD | MB | 2 | 1.332855 |
| 0.95 | LD | MB | 2 | 1.345283 |
| 0.956522 | LD | MB | 2 | 1.360741 |
| 1 | LD | MB | 1 | 1.570796 |
| 1 | LD | MB | 1 | 1.570796 |
| 1 | LD | MB | 1 | 1.570796 |
| 1 | LD | MB | 1 | 1.570796 |
| 1 | LD | MB | 1 | 1.570796 |
| 1 | LD | MB | 1 | 1.570796 |
| 1 | LD | MB | 1 | 1.570796 |
| 1 | LD | MB | 1 | 1.570796 |
| 1 | LD | MB | 1 | 1.570796 |
| 1 | LD | MB | 1 | 1.570796 |
| 1 | LD | MB | 2 | 1.570796 |
| 1 | LD | MB | 2 | 1.570796 |
| 1 | LD | MB | 2 | 1.570796 |
| 1 | LD | MB | 2 | 1.570796 |
| 1 | LD | MB | 2 | 1.570796 |
| 1 | LD | MB | 2 | 1.570796 |
| 1 | LD | MB | 2 | 1.570796 |
| 1 | LD | MB | 2 | 1.570796 |
| 1 | LD | MB | 2 | 1.570796 |
| 1 | LD | MB | 2 | 1.570796 |
| 1 | LD | MB | 2 | 1.570796 |
| 1 | LD | MB | 2 | 1.570796 |
| 1 | LD | MB | 2 | 1.570796 |
| 1 | LD | MB | 2 | 1.570796 |
| 1 | LD | MB | 2 | 1.570796 |
| 1 | LD | MB | 2 | 1.570796 |
| 1 | LD | MB | 2 | 1.570796 |
| 1 | LD | MB | 2 | 1.570796 |
| 1 | LD | MB | 2 | 1.570796 |
| 1 | LD | MB | 3 | 1.570796 |
| 1 | LD | MB | 3 | 1.570796 |
| 1 | LD | MB | 3 | 1.570796 |
| 1 | LD | MB | 3 | 1.570796 |
| 1 | LD | MB | 3 | 1.570796 |
| 1 | LD | MB | 3 | 1.570796 |
| 1 | LD | MB | 3 | 1.570796 |
| 1 | LD | MB | 3 | 1.570796 |
| 1 | LD | MB | 3 | 1.570796 |
| 1 | LD | MB | 3 | 1.570796 |
| 1 | LD | MB | 3 | 1.570796 |
| 1 | LD | MB | 3 | 1.570796 |
| 1 | LD | MB | 3 | 1.570796 |
| 1 | LD | MB | 3 | 1.570796 |
| 1 | LD | MB | 3 | 1.570796 |
| 1 | LD | MB | 4 | 1.570796 |
| 1 | LD | MB | 4 | 1.570796 |
| 1 | LD | MB | 4 | 1.570796 |
| 1 | LD | MB | 4 | 1.570796 |
| 1 | LD | MB | 4 | 1.570796 |
| 1 | LD | MB | 4 | 1.570796 |
| 1 | LD | MB | 4 | 1.570796 |
| 0.043478 | HD | MCU | 4 | 0.210056 |
| 0.090909 | HD | MCU | 4 | 0.306277 |
| 0.1 | HD | MCU | 1 | 0.321751 |
| 0.111111 | HD | MCU | 4 | 0.339837 |
| 0.142857 | HD | MCU | 3 | 0.387597 |
| 0.142857 | HD | MCU | 3 | 0.387597 |
| 0.166667 | HD | MCU | 1 | 0.420534 |
| 0.2 | HD | MCU | 3 | 0.463648 |
| 0.2 | HD | MCU | 4 | 0.463648 |
| 0.2 | HD | MCU | 4 | 0.463648 |
| 0.230769 | HD | MCU | 4 | 0.501093 |
| 0.25 | HD | MCU | 3 | 0.523599 |
| 0.285714 | HD | MCU | 2 | 0.563943 |
| 0.3 | HD | MCU | 1 | 0.57964 |
| 0.333333 | HD | MCU | 1 | 0.61548 |
| 0.333333 | HD | MCU | 1 | 0.61548 |
| 0.333333 | HD | MCU | 1 | 0.61548 |
| 0.333333 | HD | MCU | 1 | 0.61548 |
| 0.333333 | HD | MCU | 2 | 0.61548 |
| 0.384615 | HD | MCU | 2 | 0.668964 |
| 0.428571 | HD | MCU | 2 | 0.713724 |
| 0.444444 | HD | MCU | 1 | 0.729728 |
| 0.461538 | HD | MCU | 1 | 0.746899 |
| 0.466667 | HD | MCU | 4 | 0.75204 |
| 0.47619 | HD | MCU | 3 | 0.76158 |
| 0.5 | HD | MCU | 1 | 0.785398 |
| 0.5 | HD | MCU | 1 | 0.785398 |
| 0.5 | HD | MCU | 2 | 0.785398 |
| 0.5 | HD | MCU | 2 | 0.785398 |
| 0.5 | HD | MCU | 2 | 0.785398 |
| 0.5 | HD | MCU | 3 | 0.785398 |
| 0.5 | HD | MCU | 3 | 0.785398 |
| 0.5 | HD | MCU | 3 | 0.785398 |
| 0.5 | HD | MCU | 3 | 0.785398 |
| 0.533333 | HD | MCU | 3 | 0.818756 |
| 0.545455 | HD | MCU | 2 | 0.830916 |
| 0.555556 | HD | MCU | 1 | 0.841069 |
| 0.555556 | HD | MCU | 1 | 0.841069 |
| 0.571429 | HD | MCU | 2 | 0.857072 |
| 0.571429 | HD | MCU | 3 | 0.857072 |
| 0.583333 | HD | MCU | 1 | 0.869122 |
| 0.583333 | HD | MCU | 3 | 0.869122 |
| 0.588235 | HD | MCU | 4 | 0.874098 |
| 0.6 | HD | MCU | 2 | 0.886077 |
| 0.6 | HD | MCU | 3 | 0.886077 |
| 0.6 | HD | MCU | 3 | 0.886077 |
| 0.625 | HD | MCU | 1 | 0.911738 |
| 0.625 | HD | MCU | 1 | 0.911738 |
| 0.636364 | HD | MCU | 1 | 0.923511 |
| 0.636364 | HD | MCU | 3 | 0.923511 |
| 0.642857 | HD | MCU | 1 | 0.930274 |
| 0.642857 | HD | MCU | 3 | 0.930274 |
| 0.65 | HD | MCU | 3 | 0.937744 |
| 0.666667 | HD | MCU | 1 | 0.955317 |
| 0.666667 | HD | MCU | 1 | 0.955317 |
| 0.666667 | HD | MCU | 1 | 0.955317 |
| 0.666667 | HD | MCU | 1 | 0.955317 |
| 0.666667 | HD | MCU | 1 | 0.955317 |
| 0.666667 | HD | MCU | 2 | 0.955317 |
| 0.666667 | HD | MCU | 2 | 0.955317 |
| 0.666667 | HD | MCU | 3 | 0.955317 |
| 0.666667 | HD | MCU | 3 | 0.955317 |
| 0.666667 | HD | MCU | 3 | 0.955317 |
| 0.666667 | HD | MCU | 3 | 0.955317 |
| 0.666667 | HD | MCU | 4 | 0.955317 |
| 0.7 | HD | MCU | 1 | 0.991157 |
| 0.7 | HD | MCU | 1 | 0.991157 |
| 0.7 | HD | MCU | 3 | 0.991157 |
| 0.7 | HD | MCU | 4 | 0.991157 |
| 0.7 | HD | MCU | 2 | 0.991157 |
| 0.708333 | HD | MCU | 1 | 1.000286 |
| 0.727273 | HD | MCU | 1 | 1.021329 |
| 0.727273 | HD | MCU | 3 | 1.021329 |
| 0.736842 | HD | MCU | 1 | 1.032133 |
| 0.75 | HD | MCU | 1 | 1.047198 |
| 0.75 | HD | MCU | 1 | 1.047198 |
| 0.75 | HD | MCU | 2 | 1.047198 |
| 0.75 | HD | MCU | 2 | 1.047198 |
| 0.75 | HD | MCU | 2 | 1.047198 |
| 0.75 | HD | MCU | 3 | 1.047198 |
| 0.75 | HD | MCU | 3 | 1.047198 |
| 0.75 | HD | MCU | 4 | 1.047198 |
| 0.764706 | HD | MCU | 2 | 1.064352 |
| 0.777778 | HD | MCU | 2 | 1.079914 |
| 0.777778 | HD | MCU | 2 | 1.079914 |
| 0.777778 | HD | MCU | 3 | 1.079914 |
| 0.777778 | HD | MCU | 3 | 1.079914 |
| 0.785714 | HD | MCU | 1 | 1.089521 |
| 0.785714 | HD | MCU | 1 | 1.089521 |
| 0.785714 | HD | MCU | 3 | 1.089521 |
| 0.791667 | HD | MCU | 3 | 1.096811 |
| 0.8 | HD | MCU | 1 | 1.107149 |
| 0.8 | HD | MCU | 3 | 1.107149 |
| 0.8 | HD | MCU | 3 | 1.107149 |
| 0.8 | HD | MCU | 3 | 1.107149 |
| 0.8 | HD | MCU | 4 | 1.107149 |
| 0.8 | HD | MCU | 4 | 1.107149 |
| 0.818182 | HD | MCU | 1 | 1.130286 |
| 0.818182 | HD | MCU | 1 | 1.130286 |
| 0.818182 | HD | MCU | 3 | 1.130286 |
| 0.818182 | HD | MCU | 3 | 1.130286 |
| 0.833333 | HD | MCU | 1 | 1.150262 |
| 0.833333 | HD | MCU | 3 | 1.150262 |
| 0.833333 | HD | MCU | 4 | 1.150262 |
| 0.833333 | HD | MCU | 2 | 1.150262 |
| 0.842105 | HD | MCU | 3 | 1.162158 |
| 0.846154 | HD | MCU | 1 | 1.167739 |
| 0.846154 | HD | MCU | 1 | 1.167739 |
| 0.857143 | HD | MCU | 2 | 1.1832 |
| 0.857143 | HD | MCU | 3 | 1.1832 |
| 0.857143 | HD | MCU | 3 | 1.1832 |
| 0.857143 | HD | MCU | 3 | 1.1832 |
| 0.857143 | HD | MCU | 3 | 1.1832 |
| 0.857143 | HD | MCU | 3 | 1.1832 |
| 0.866667 | HD | MCU | 1 | 1.197004 |
| 0.866667 | HD | MCU | 2 | 1.197004 |
| 0.875 | HD | MCU | 1 | 1.209429 |
| 0.875 | HD | MCU | 1 | 1.209429 |
| 0.875 | HD | MCU | 1 | 1.209429 |
| 0.875 | HD | MCU | 2 | 1.209429 |
| 0.875 | HD | MCU | 3 | 1.209429 |
| 0.875 | HD | MCU | 3 | 1.209429 |
| 0.875 | HD | MCU | 3 | 1.209429 |
| 0.875 | HD | MCU | 2 | 1.209429 |
| 0.884615 | HD | MCU | 1 | 1.224216 |
| 0.888889 | HD | MCU | 4 | 1.230959 |
| 0.888889 | HD | MCU | 4 | 1.230959 |
| 0.9 | HD | MCU | 1 | 1.249046 |
| 0.9 | HD | MCU | 1 | 1.249046 |
| 0.9 | HD | MCU | 1 | 1.249046 |
| 0.9 | HD | MCU | 1 | 1.249046 |
| 0.9 | HD | MCU | 2 | 1.249046 |
| 0.9 | HD | MCU | 3 | 1.249046 |
| 0.9 | HD | MCU | 3 | 1.249046 |
| 0.909091 | HD | MCU | 1 | 1.264519 |
| 0.909091 | HD | MCU | 3 | 1.264519 |
| 0.909091 | HD | MCU | 4 | 1.264519 |
| 0.916667 | HD | MCU | 1 | 1.277954 |
| 0.916667 | HD | MCU | 1 | 1.277954 |
| 0.916667 | HD | MCU | 3 | 1.277954 |
| 0.916667 | HD | MCU | 4 | 1.277954 |
| 0.923077 | HD | MCU | 3 | 1.289761 |
| 0.923077 | HD | MCU | 4 | 1.289761 |
| 0.933333 | HD | MCU | 3 | 1.309639 |
| 0.9375 | HD | MCU | 1 | 1.318116 |
| 0.941176 | HD | MCU | 2 | 1.325818 |
| 0.944444 | HD | MCU | 2 | 1.332855 |
| 0.944444 | HD | MCU | 3 | 1.332855 |
| 0.954545 | HD | MCU | 2 | 1.355946 |
| 1 | HD | MCU | 1 | 1.570796 |
| 1 | HD | MCU | 1 | 1.570796 |
| 1 | HD | MCU | 1 | 1.570796 |
| 1 | HD | MCU | 1 | 1.570796 |
| 1 | HD | MCU | 1 | 1.570796 |
| 1 | HD | MCU | 1 | 1.570796 |
| 1 | HD | MCU | 1 | 1.570796 |
| 1 | HD | MCU | 1 | 1.570796 |
| 1 | HD | MCU | 1 | 1.570796 |
| 1 | HD | MCU | 1 | 1.570796 |
| 1 | HD | MCU | 1 | 1.570796 |
| 1 | HD | MCU | 1 | 1.570796 |
| 1 | HD | MCU | 1 | 1.570796 |
| 1 | HD | MCU | 1 | 1.570796 |
| 1 | HD | MCU | 1 | 1.570796 |
| 1 | HD | MCU | 2 | 1.570796 |
| 1 | HD | MCU | 2 | 1.570796 |
| 1 | HD | MCU | 2 | 1.570796 |
| 1 | HD | MCU | 2 | 1.570796 |
| 1 | HD | MCU | 2 | 1.570796 |
| 1 | HD | MCU | 2 | 1.570796 |
| 1 | HD | MCU | 2 | 1.570796 |
| 1 | HD | MCU | 2 | 1.570796 |
| 1 | HD | MCU | 2 | 1.570796 |
| 1 | HD | MCU | 2 | 1.570796 |
| 1 | HD | MCU | 2 | 1.570796 |
| 1 | HD | MCU | 2 | 1.570796 |
| 1 | HD | MCU | 2 | 1.570796 |
| 1 | HD | MCU | 2 | 1.570796 |
| 1 | HD | MCU | 2 | 1.570796 |
| 1 | HD | MCU | 2 | 1.570796 |
| 1 | HD | MCU | 2 | 1.570796 |
| 1 | HD | MCU | 2 | 1.570796 |
| 1 | HD | MCU | 2 | 1.570796 |
| 1 | HD | MCU | 3 | 1.570796 |
| 1 | HD | MCU | 3 | 1.570796 |
| 1 | HD | MCU | 3 | 1.570796 |
| 1 | HD | MCU | 3 | 1.570796 |
| 1 | HD | MCU | 3 | 1.570796 |
| 1 | HD | MCU | 3 | 1.570796 |
| 1 | HD | MCU | 3 | 1.570796 |
| 1 | HD | MCU | 3 | 1.570796 |
| 1 | HD | MCU | 3 | 1.570796 |
| 1 | HD | MCU | 3 | 1.570796 |
| 1 | HD | MCU | 3 | 1.570796 |
| 1 | HD | MCU | 3 | 1.570796 |
| 1 | HD | MCU | 3 | 1.570796 |
| 1 | HD | MCU | 3 | 1.570796 |
| 1 | HD | MCU | 3 | 1.570796 |
| 1 | HD | MCU | 3 | 1.570796 |
| 1 | HD | MCU | 3 | 1.570796 |
| 1 | HD | MCU | 3 | 1.570796 |
| 1 | HD | MCU | 3 | 1.570796 |
| 1 | HD | MCU | 3 | 1.570796 |
| 1 | HD | MCU | 3 | 1.570796 |
| 1 | HD | MCU | 3 | 1.570796 |
| 1 | HD | MCU | 3 | 1.570796 |
| 1 | HD | MCU | 3 | 1.570796 |
| 1 | HD | MCU | 3 | 1.570796 |
| 1 | HD | MCU | 4 | 1.570796 |
| 1 | HD | MCU | 4 | 1.570796 |
| 1 | HD | MCU | 4 | 1.570796 |
| 1 | HD | MCU | 4 | 1.570796 |
| 1 | HD | MCU | 4 | 1.570796 |
| 1 | HD | MCU | 4 | 1.570796 |
| 1 | HD | MCU | 4 | 1.570796 |
| 0.277778 | LD | MCU | 3 | 0.555121 |
| 0.3 | LD | MCU | 2 | 0.57964 |
| 0.333333 | LD | MCU | 1 | 0.61548 |
| 0.333333 | LD | MCU | 2 | 0.61548 |
| 0.333333 | LD | MCU | 3 | 0.61548 |
| 0.333333 | LD | MCU | 4 | 0.61548 |
| 0.384615 | LD | MCU | 2 | 0.668964 |
| 0.473684 | LD | MCU | 1 | 0.75907 |
| 0.5 | LD | MCU | 2 | 0.785398 |
| 0.5 | LD | MCU | 2 | 0.785398 |
| 0.5 | LD | MCU | 3 | 0.785398 |
| 0.555556 | LD | MCU | 1 | 0.841069 |
| 0.571429 | LD | MCU | 2 | 0.857072 |
| 0.571429 | LD | MCU | 4 | 0.857072 |
| 0.583333 | LD | MCU | 3 | 0.869122 |
| 0.6 | LD | MCU | 3 | 0.886077 |
| 0.625 | LD | MCU | 4 | 0.911738 |
| 0.666667 | LD | MCU | 1 | 0.955317 |
| 0.666667 | LD | MCU | 1 | 0.955317 |
| 0.666667 | LD | MCU | 1 | 0.955317 |
| 0.666667 | LD | MCU | 1 | 0.955317 |
| 0.666667 | LD | MCU | 2 | 0.955317 |
| 0.666667 | LD | MCU | 2 | 0.955317 |
| 0.666667 | LD | MCU | 4 | 0.955317 |
| 0.684211 | LD | MCU | 3 | 0.974053 |
| 0.7 | LD | MCU | 1 | 0.991157 |
| 0.7 | LD | MCU | 4 | 0.991157 |
| 0.714286 | LD | MCU | 1 | 1.006854 |
| 0.714286 | LD | MCU | 2 | 1.006854 |
| 0.714286 | LD | MCU | 2 | 1.006854 |
| 0.714286 | LD | MCU | 3 | 1.006854 |
| 0.727273 | LD | MCU | 1 | 1.021329 |
| 0.727273 | LD | MCU | 1 | 1.021329 |
| 0.727273 | LD | MCU | 4 | 1.021329 |
| 0.733333 | LD | MCU | 1 | 1.028157 |
| 0.733333 | LD | MCU | 3 | 1.028157 |
| 0.75 | LD | MCU | 2 | 1.047198 |
| 0.75 | LD | MCU | 2 | 1.047198 |
| 0.75 | LD | MCU | 2 | 1.047198 |
| 0.75 | LD | MCU | 2 | 1.047198 |
| 0.75 | LD | MCU | 3 | 1.047198 |
| 0.75 | LD | MCU | 4 | 1.047198 |
| 0.761905 | LD | MCU | 1 | 1.061057 |
| 0.764706 | LD | MCU | 2 | 1.064352 |
| 0.764706 | LD | MCU | 3 | 1.064352 |
| 0.769231 | LD | MCU | 2 | 1.069703 |
| 0.769231 | LD | MCU | 4 | 1.069703 |
| 0.785714 | LD | MCU | 1 | 1.089521 |
| 0.785714 | LD | MCU | 3 | 1.089521 |
| 0.8 | LD | MCU | 1 | 1.107149 |
| 0.8 | LD | MCU | 4 | 1.107149 |
| 0.809524 | LD | MCU | 4 | 1.119163 |
| 0.8125 | LD | MCU | 3 | 1.122964 |
| 0.833333 | LD | MCU | 1 | 1.150262 |
| 0.833333 | LD | MCU | 1 | 1.150262 |
| 0.833333 | LD | MCU | 3 | 1.150262 |
| 0.833333 | LD | MCU | 3 | 1.150262 |
| 0.833333 | LD | MCU | 3 | 1.150262 |
| 0.842105 | LD | MCU | 4 | 1.162158 |
| 0.846154 | LD | MCU | 1 | 1.167739 |
| 0.846154 | LD | MCU | 1 | 1.167739 |
| 0.85 | LD | MCU | 1 | 1.173097 |
| 0.857143 | LD | MCU | 1 | 1.1832 |
| 0.857143 | LD | MCU | 2 | 1.1832 |
| 0.857143 | LD | MCU | 2 | 1.1832 |
| 0.857143 | LD | MCU | 2 | 1.1832 |
| 0.857143 | LD | MCU | 2 | 1.1832 |
| 0.857143 | LD | MCU | 3 | 1.1832 |
| 0.857143 | LD | MCU | 3 | 1.1832 |
| 0.857143 | LD | MCU | 4 | 1.1832 |
| 0.857143 | LD | MCU | 4 | 1.1832 |
| 0.866667 | LD | MCU | 3 | 1.197004 |
| 0.875 | LD | MCU | 1 | 1.209429 |
| 0.875 | LD | MCU | 1 | 1.209429 |
| 0.875 | LD | MCU | 1 | 1.209429 |
| 0.875 | LD | MCU | 2 | 1.209429 |
| 0.875 | LD | MCU | 3 | 1.209429 |
| 0.875 | LD | MCU | 3 | 1.209429 |
| 0.888889 | LD | MCU | 3 | 1.230959 |
| 0.894737 | LD | MCU | 2 | 1.240374 |
| 0.9 | LD | MCU | 1 | 1.249046 |
| 0.9 | LD | MCU | 1 | 1.249046 |
| 0.9 | LD | MCU | 2 | 1.249046 |
| 0.9 | LD | MCU | 2 | 1.249046 |
| 0.9 | LD | MCU | 2 | 1.249046 |
| 0.9 | LD | MCU | 4 | 1.249046 |
| 0.909091 | LD | MCU | 2 | 1.264519 |
| 0.909091 | LD | MCU | 3 | 1.264519 |
| 0.909091 | LD | MCU | 4 | 1.264519 |
| 0.916667 | LD | MCU | 1 | 1.277954 |
| 0.916667 | LD | MCU | 1 | 1.277954 |
| 0.916667 | LD | MCU | 1 | 1.277954 |
| 0.928571 | LD | MCU | 1 | 1.300247 |
| 0.928571 | LD | MCU | 4 | 1.300247 |
| 0.941176 | LD | MCU | 3 | 1.325818 |
| 0.954545 | LD | MCU | 3 | 1.355946 |
| 0.958333 | LD | MCU | 2 | 1.365227 |
| 1 | LD | MCU | 1 | 1.570796 |
| 1 | LD | MCU | 1 | 1.570796 |
| 1 | LD | MCU | 1 | 1.570796 |
| 1 | LD | MCU | 1 | 1.570796 |
| 1 | LD | MCU | 1 | 1.570796 |
| 1 | LD | MCU | 1 | 1.570796 |
| 1 | LD | MCU | 1 | 1.570796 |
| 1 | LD | MCU | 1 | 1.570796 |
| 1 | LD | MCU | 1 | 1.570796 |
| 1 | LD | MCU | 1 | 1.570796 |
| 1 | LD | MCU | 1 | 1.570796 |
| 1 | LD | MCU | 1 | 1.570796 |
| 1 | LD | MCU | 1 | 1.570796 |
| 1 | LD | MCU | 1 | 1.570796 |
| 1 | LD | MCU | 2 | 1.570796 |
| 1 | LD | MCU | 2 | 1.570796 |
| 1 | LD | MCU | 2 | 1.570796 |
| 1 | LD | MCU | 2 | 1.570796 |
| 1 | LD | MCU | 2 | 1.570796 |
| 1 | LD | MCU | 2 | 1.570796 |
| 1 | LD | MCU | 2 | 1.570796 |
| 1 | LD | MCU | 2 | 1.570796 |
| 1 | LD | MCU | 2 | 1.570796 |
| 1 | LD | MCU | 2 | 1.570796 |
| 1 | LD | MCU | 2 | 1.570796 |
| 1 | LD | MCU | 2 | 1.570796 |
| 1 | LD | MCU | 2 | 1.570796 |
| 1 | LD | MCU | 2 | 1.570796 |
| 1 | LD | MCU | 2 | 1.570796 |
| 1 | LD | MCU | 3 | 1.570796 |
| 1 | LD | MCU | 3 | 1.570796 |
| 1 | LD | MCU | 3 | 1.570796 |
| 1 | LD | MCU | 3 | 1.570796 |
| 1 | LD | MCU | 3 | 1.570796 |
| 1 | LD | MCU | 3 | 1.570796 |
| 1 | LD | MCU | 3 | 1.570796 |
| 1 | LD | MCU | 3 | 1.570796 |
| 1 | LD | MCU | 3 | 1.570796 |
| 1 | LD | MCU | 3 | 1.570796 |
| 1 | LD | MCU | 3 | 1.570796 |
| 1 | LD | MCU | 3 | 1.570796 |
| 1 | LD | MCU | 3 | 1.570796 |
| 1 | LD | MCU | 3 | 1.570796 |
| 1 | LD | MCU | 3 | 1.570796 |
| 1 | LD | MCU | 3 | 1.570796 |
| 1 | LD | MCU | 4 | 1.570796 |
| 1 | LD | MCU | 4 | 1.570796 |
| 1 | LD | MCU | 4 | 1.570796 |
| 1 | LD | MCU | 4 | 1.570796 |
| 1 | LD | MCU | 4 | 1.570796 |
| 1 | LD | MCU | 4 | 1.570796 |
| 1 | LD | MCU | 4 | 1.570796 |
| 1 | LD | MCU | 4 | 1.570796 |
| 1 | LD | MCU | 4 | 1.570796 |
| 1 | LD | MCU | 4 | 1.570796 |
| 1 | LD | MCU | 4 | 1.570796 |

Supplementary Table 4: Sperm defence assay, 4 day old males, non-transformed data with red and white eye values

| Selection | Treatment | Vial ID | Red | White | P1 | Block |
| --- | --- | --- | --- | --- | --- | --- |
| MCU | HD | 3 | 6 | 12 | 0.333333333 | 1 |
| MCU | HD | 4 | 2 | 8 | 0.2 | 1 |
| MCU | HD | 5 | 1 | 15 | 0.0625 | 1 |
| MCU | HD | 6 | 2 | 20 | 0.090909091 | 1 |
| MCU | HD | 7 | 6 | 0 | 1 | 1 |
| MCU | HD | 11 | 7 | 13 | 0.35 | 1 |
| MCU | HD | 13 | 6 | 8 | 0.428571429 | 1 |
| MCU | HD | 14 | 2 | 8 | 0.2 | 1 |
| MCU | HD | 15 | 2 | 12 | 0.142857143 | 1 |
| MCU | HD | 18 | 2 | 24 | 0.076923077 | 1 |
| MCU | HD | 20 | 9 | 7 | 0.5625 | 1 |
| MCU | HD | 22 | 4 | 22 | 0.153846154 | 1 |
| MCU | HD | 26 | 4 | 12 | 0.25 | 1 |
| MCU | HD | 29 | 1 | 15 | 0.0625 | 1 |
| MCU | HD | 30 | 7 | 17 | 0.291666667 | 1 |
| MCU | HD | 35 | 1 | 4 | 0.2 | 1 |
| MCU | HD | 36 | 10 | 0 | 1 | 1 |
| MCU | HD | 39 | 8 | 8 | 0.5 | 1 |
| MCU | HD | 41 | 1 | 9 | 0.1 | 1 |
| MCU | HD | 48 | 2 | 12 | 0.142857143 | 1 |
| MCU | HD | 54 | 17 | 0 | 1 | 1 |
| MCU | HD | 64 | 10 | 4 | 0.714285714 | 1 |
| MCU | HD | 67 | 20 | 0 | 1 | 1 |
| MCU | HD | 69 | 1 | 9 | 0.1 | 1 |
| MCU | HD | 70 | 1 | 17 | 0.055555556 | 1 |
| MCU | HD | 71 | 1 | 14 | 0.066666667 | 1 |
| MCU | HD | 73 | 4 | 24 | 0.142857143 | 1 |
| MCU | HD | 74 | 10 | 12 | 0.454545455 | 1 |
| MCU | HD | 77 | 3 | 21 | 0.125 | 1 |
| MCU | HD | 79 | 2 | 14 | 0.125 | 1 |
| MCU | HD | 80 | 2 | 18 | 0.1 | 1 |
| MCU | HD | 94 | 8 | 12 | 0.4 | 1 |
| MCU | HD | 97 | 2 | 22 | 0.083333333 | 1 |
| MCU | HD | 100 | 2 | 19 | 0.095238095 | 1 |
| MCU | LD | 301 | 1 | 9 | 0.1 | 1 |
| MCU | LD | 302 | 5 | 13 | 0.277777778 | 1 |
| MCU | LD | 304 | 11 | 1 | 0.916666667 | 1 |
| MCU | LD | 306 | 3 | 15 | 0.166666667 | 1 |
| MCU | LD | 309 | 1 | 11 | 0.083333333 | 1 |
| MCU | LD | 312 | 7 | 15 | 0.318181818 | 1 |
| MCU | LD | 313 | 4 | 16 | 0.2 | 1 |
| MCU | LD | 314 | 16 | 15 | 0.516129032 | 1 |
| MCU | LD | 315 | 7 | 12 | 0.368421053 | 1 |
| MCU | LD | 316 | 4 | 14 | 0.222222222 | 1 |
| MCU | LD | 322 | 4 | 4 | 0.5 | 1 |
| MCU | LD | 325 | 5 | 12 | 0.294117647 | 1 |
| MCU | LD | 327 | 3 | 19 | 0.136363636 | 1 |
| MCU | LD | 329 | 2 | 9 | 0.181818182 | 1 |
| MCU | LD | 330 | 1 | 16 | 0.058823529 | 1 |
| MCU | LD | 331 | 7 | 11 | 0.388888889 | 1 |
| MCU | LD | 332 | 4 | 7 | 0.363636364 | 1 |
| MCU | LD | 333 | 3 | 8 | 0.272727273 | 1 |
| MCU | LD | 334 | 2 | 9 | 0.181818182 | 1 |
| MCU | LD | 344 | 1 | 14 | 0.066666667 | 1 |
| MCU | LD | 345 | 3 | 13 | 0.1875 | 1 |
| MCU | LD | 347 | 7 | 4 | 0.636363636 | 1 |
| MCU | LD | 349 | 3 | 11 | 0.214285714 | 1 |
| MCU | LD | 351 | 2 | 5 | 0.285714286 | 1 |
| MCU | LD | 352 | 4 | 12 | 0.25 | 1 |
| MCU | LD | 353 | 3 | 13 | 0.1875 | 1 |
| MCU | LD | 354 | 3 | 0 | 1 | 1 |
| MCU | LD | 357 | 2 | 11 | 0.153846154 | 1 |
| MCU | LD | 358 | 2 | 9 | 0.181818182 | 1 |
| MCU | LD | 359 | 2 | 10 | 0.166666667 | 1 |
| MCU | LD | 362 | 8 | 11 | 0.421052632 | 1 |
| MCU | LD | 363 | 2 | 12 | 0.142857143 | 1 |
| MCU | LD | 364 | 9 | 0 | 1 | 1 |
| MCU | LD | 365 | 2 | 7 | 0.222222222 | 1 |
| MCU | LD | 368 | 1 | 16 | 0.058823529 | 1 |
| MCU | LD | 371 | 5 | 10 | 0.333333333 | 1 |
| MCU | LD | 374 | 1 | 14 | 0.066666667 | 1 |
| MCU | LD | 375 | 4 | 17 | 0.190476191 | 1 |
| MCU | LD | 378 | 2 | 14 | 0.125 | 1 |
| MCU | LD | 380 | 20 | 0 | 1 | 1 |
| MCU | LD | 381 | 2 | 7 | 0.222222222 | 1 |
| MCU | LD | 382 | 1 | 21 | 0.045454545 | 1 |
| MCU | LD | 383 | 1 | 5 | 0.166666667 | 1 |
| MCU | LD | 384 | 1 | 15 | 0.0625 | 1 |
| MCU | LD | 392 | 3 | 15 | 0.166666667 | 1 |
| MCU | LD | 399 | 3 | 13 | 0.1875 | 1 |
| MB | LD | 204 | 1 | 7 | 0.125 | 1 |
| MB | LD | 205 | 1 | 17 | 0.055555556 | 1 |
| MB | LD | 206 | 3 | 10 | 0.230769231 | 1 |
| MB | LD | 211 | 13 | 9 | 0.590909091 | 1 |
| MB | LD | 212 | 7 | 0 | 1 | 1 |
| MB | LD | 213 | 5 | 5 | 0.5 | 1 |
| MB | LD | 215 | 3 | 6 | 0.333333333 | 1 |
| MB | LD | 218 | 4 | 6 | 0.4 | 1 |
| MB | LD | 221 | 4 | 12 | 0.25 | 1 |
| MB | LD | 226 | 2 | 7 | 0.222222222 | 1 |
| MB | LD | 227 | 2 | 11 | 0.153846154 | 1 |
| MB | LD | 229 | 3 | 11 | 0.214285714 | 1 |
| MB | LD | 230 | 2 | 18 | 0.1 | 1 |
| MB | LD | 241 | 5 | 10 | 0.333333333 | 1 |
| MB | LD | 242 | 19 | 0 | 1 | 1 |
| MB | LD | 246 | 1 | 8 | 0.111111111 | 1 |
| MB | LD | 247 | 7 | 0 | 1 | 1 |
| MB | LD | 248 | 1 | 3 | 0.25 | 1 |
| MB | LD | 249 | 4 | 13 | 0.235294118 | 1 |
| MB | LD | 250 | 11 | 0 | 1 | 1 |
| MB | LD | 254 | 4 | 12 | 0.25 | 1 |
| MB | LD | 255 | 8 | 2 | 0.8 | 1 |
| MB | LD | 258 | 1 | 32 | 0.03030303 | 1 |
| MB | LD | 259 | 4 | 12 | 0.25 | 1 |
| MB | LD | 261 | 10 | 17 | 0.37037037 | 1 |
| MB | LD | 264 | 2 | 14 | 0.125 | 1 |
| MB | LD | 268 | 4 | 7 | 0.363636364 | 1 |
| MB | LD | 270 | 2 | 16 | 0.111111111 | 1 |
| MB | LD | 276 | 3 | 28 | 0.096774194 | 1 |
| MB | LD | 278 | 7 | 11 | 0.388888889 | 1 |
| MB | LD | 280 | 2 | 8 | 0.2 | 1 |
| MB | LD | 282 | 9 | 14 | 0.391304348 | 1 |
| MB | LD | 284 | 4 | 1 | 0.8 | 1 |
| MB | LD | 285 | 1 | 13 | 0.071428571 | 1 |
| MB | LD | 287 | 7 | 16 | 0.304347826 | 1 |
| MB | LD | 291 | 1 | 20 | 0.047619048 | 1 |
| MB | LD | 291 | 1 | 12 | 0.076923077 | 1 |
| MB | LD | 295 | 5 | 18 | 0.217391304 | 1 |
| MB | LD | 296 | 2 | 11 | 0.153846154 | 1 |
| MB | LD | 297 | 1 | 4 | 0.2 | 1 |
| MB | LD | 299 | 2 | 3 | 0.4 | 1 |
| MB | LD | 300 | 10 | 4 | 0.714285714 | 1 |
| MB | HD | 111 | 2 | 17 | 0.105263158 | 1 |
| MB | HD | 112 | 1 | 5 | 0.166666667 | 1 |
| MB | HD | 113 | 6 | 7 | 0.461538462 | 1 |
| MB | HD | 114 | 1 | 15 | 0.0625 | 1 |
| MB | HD | 116 | 1 | 11 | 0.083333333 | 1 |
| MB | HD | 119 | 1 | 8 | 0.111111111 | 1 |
| MB | HD | 121 | 4 | 7 | 0.363636364 | 1 |
| MB | HD | 125 | 3 | 6 | 0.333333333 | 1 |
| MB | HD | 128 | 1 | 4 | 0.2 | 1 |
| MB | HD | 129 | 13 | 4 | 0.764705882 | 1 |
| MB | HD | 130 | 1 | 12 | 0.076923077 | 1 |
| MB | HD | 133 | 8 | 17 | 0.32 | 1 |
| MB | HD | 134 | 10 | 4 | 0.714285714 | 1 |
| MB | HD | 135 | 1 | 7 | 0.125 | 1 |
| MB | HD | 137 | 4 | 4 | 0.5 | 1 |
| MB | HD | 139 | 6 | 21 | 0.222222222 | 1 |
| MB | HD | 140 | 3 | 18 | 0.142857143 | 1 |
| MB | HD | 141 | 20 | 0 | 1 | 1 |
| MB | HD | 142 | 3 | 15 | 0.166666667 | 1 |
| MB | HD | 143 | 11 | 3 | 0.785714286 | 1 |
| MB | HD | 149 | 1 | 17 | 0.055555556 | 1 |
| MB | HD | 162 | 25 | 10 | 0.714285714 | 1 |
| MB | HD | 164 | 2 | 4 | 0.333333333 | 1 |
| MB | HD | 165 | 4 | 21 | 0.16 | 1 |
| MB | HD | 166 | 17 | 12 | 0.586206897 | 1 |
| MB | HD | 167 | 3 | 30 | 0.090909091 | 1 |
| MB | HD | 169 | 15 | 24 | 0.384615385 | 1 |
| MB | HD | 170 | 9 | 17 | 0.346153846 | 1 |
| MB | HD | 172 | 11 | 6 | 0.647058824 | 1 |
| MB | HD | 174 | 4 | 4 | 0.5 | 1 |
| MB | HD | 175 | 6 | 4 | 0.6 | 1 |
| MB | HD | 176 | 3 | 18 | 0.142857143 | 1 |
| MB | HD | 179 | 1 | 21 | 0.045454545 | 1 |
| MB | HD | 182 | 6 | 3 | 0.666666667 | 1 |
| MB | HD | 183 | 3 | 12 | 0.2 | 1 |
| MB | HD | 184 | 12 | 3 | 0.8 | 1 |
| MB | HD | 185 | 4 | 13 | 0.235294118 | 1 |
| MB | HD | 187 | 3 | 10 | 0.230769231 | 1 |
| MB | HD | 188 | 4 | 13 | 0.235294118 | 1 |
| MB | HD | 189 | 2 | 12 | 0.142857143 | 1 |
| MB | HD | 190 | 7 | 1 | 0.875 | 1 |
| MB | HD | 192 | 2 | 17 | 0.105263158 | 1 |
| MB | HD | 194 | 4 | 7 | 0.363636364 | 1 |
| MB | HD | 195 | 9 | 9 | 0.5 | 1 |
| MB | HD | 196 | 6 | 0 | 1 | 1 |
| MB | HD | 197 | 10 | 6 | 0.625 | 1 |
| MB | HD | 198 | 2 | 14 | 0.125 | 1 |
| MB | HD | 200 | 5 | 6 | 0.454545455 | 1 |
| MCU | LD | 211 | 0 | 2 | 0 | 2 |
| MCU | LD | 215 | 0 | 2 | 0 | 2 |
| MCU | LD | 223 | 0 | 13 | 0 | 2 |
| MCU | LD | 277 | 0 | 9 | 0 | 2 |
| MCU | LD | 289 | 0 | 5 | 0 | 2 |
| MCU | LD | 294 | 0 | 8 | 0 | 2 |
| MCU | LD | 298 | 0 | 11 | 0 | 2 |
| MCU | LD | 255 | 1 | 9 | 0.1 | 2 |
| MCU | LD | 210 | 1 | 8 | 0.111111111 | 2 |
| MCU | LD | 201 | 1 | 6 | 0.142857143 | 2 |
| MCU | LD | 293 | 1 | 6 | 0.142857143 | 2 |
| MCU | LD | 247 | 1 | 5 | 0.166666667 | 2 |
| MCU | LD | 283 | 1 | 4 | 0.2 | 2 |
| MCU | LD | 292 | 1 | 4 | 0.2 | 2 |
| MCU | LD | 299 | 1 | 4 | 0.2 | 2 |
| MCU | LD | 225 | 1 | 3 | 0.25 | 2 |
| MCU | LD | 244 | 2 | 6 | 0.25 | 2 |
| MCU | LD | 281 | 2 | 6 | 0.25 | 2 |
| MCU | LD | 286 | 2 | 6 | 0.25 | 2 |
| MCU | LD | 205 | 3 | 8 | 0.272727273 | 2 |
| MCU | LD | 242 | 2 | 5 | 0.285714286 | 2 |
| MCU | LD | 221 | 3 | 7 | 0.3 | 2 |
| MCU | LD | 248 | 2 | 4 | 0.333333333 | 2 |
| MCU | LD | 265 | 2 | 4 | 0.333333333 | 2 |
| MCU | LD | 276 | 1 | 2 | 0.333333333 | 2 |
| MCU | LD | 278 | 4 | 8 | 0.333333333 | 2 |
| MCU | LD | 232 | 2 | 3 | 0.4 | 2 |
| MCU | LD | 252 | 9 | 12 | 0.428571429 | 2 |
| MCU | LD | 266 | 3 | 4 | 0.428571429 | 2 |
| MCU | LD | 222 | 7 | 9 | 0.4375 | 2 |
| MCU | LD | 250 | 6 | 7 | 0.461538462 | 2 |
| MCU | LD | 202 | 5 | 5 | 0.5 | 2 |
| MCU | LD | 260 | 2 | 2 | 0.5 | 2 |
| MCU | LD | 269 | 2 | 2 | 0.5 | 2 |
| MCU | LD | 207 | 4 | 3 | 0.571428571 | 2 |
| MCU | LD | 229 | 3 | 2 | 0.6 | 2 |
| MCU | LD | 243 | 3 | 2 | 0.6 | 2 |
| MCU | LD | 273 | 5 | 3 | 0.625 | 2 |
| MCU | LD | 251 | 9 | 5 | 0.642857143 | 2 |
| MCU | LD | 279 | 2 | 1 | 0.666666667 | 2 |
| MCU | LD | 246 | 7 | 3 | 0.7 | 2 |
| MCU | LD | 220 | 4 | 1 | 0.8 | 2 |
| MCU | LD | 238 | 11 | 2 | 0.846153846 | 2 |
| MCU | LD | 203 | 1 | 0 | 1 | 2 |
| MCU | LD | 216 | 7 | 0 | 1 | 2 |
| MCU | LD | 233 | 5 | 0 | 1 | 2 |
| MCU | LD | 245 | 1 | 0 | 1 | 2 |
| MCU | LD | 253 | 4 | 0 | 1 | 2 |
| MCU | LD | 291 | 1 | 0 | 1 | 2 |
| MCU | HD | 16 | 0 | 7 | 0 | 2 |
| MCU | HD | 26 | 0 | 1 | 0 | 2 |
| MCU | HD | 30 | 0 | 3 | 0 | 2 |
| MCU | HD | 49 | 0 | 5 | 0 | 2 |
| MCU | HD | 50 | 0 | 11 | 0 | 2 |
| MCU | HD | 72 | 0 | 9 | 0 | 2 |
| MCU | HD | 83 | 0 | 10 | 0 | 2 |
| MCU | HD | 94 | 0 | 4 | 0 | 2 |
| MCU | HD | 98 | 0 | 2 | 0 | 2 |
| MCU | HD | 18 | 1 | 9 | 0.1 | 2 |
| MCU | HD | 44 | 1 | 5 | 0.166666667 | 2 |
| MCU | HD | 59 | 3 | 13 | 0.1875 | 2 |
| MCU | HD | 69 | 3 | 12 | 0.2 | 2 |
| MCU | HD | 79 | 2 | 8 | 0.2 | 2 |
| MCU | HD | 27 | 3 | 11 | 0.214285714 | 2 |
| MCU | HD | 46 | 1 | 3 | 0.25 | 2 |
| MCU | HD | 60 | 1 | 3 | 0.25 | 2 |
| MCU | HD | 65 | 1 | 3 | 0.25 | 2 |
| MCU | HD | 82 | 2 | 6 | 0.25 | 2 |
| MCU | HD | 95 | 1 | 3 | 0.25 | 2 |
| MCU | HD | 99 | 1 | 3 | 0.25 | 2 |
| MCU | HD | 85 | 4 | 11 | 0.266666667 | 2 |
| MCU | HD | 2 | 2 | 5 | 0.285714286 | 2 |
| MCU | HD | 5 | 6 | 12 | 0.333333333 | 2 |
| MCU | HD | 8 | 2 | 4 | 0.333333333 | 2 |
| MCU | HD | 41 | 2 | 4 | 0.333333333 | 2 |
| MCU | HD | 77 | 1 | 2 | 0.333333333 | 2 |
| MCU | HD | 90 | 2 | 4 | 0.333333333 | 2 |
| MCU | HD | 28 | 4 | 7 | 0.363636364 | 2 |
| MCU | HD | 4 | 2 | 3 | 0.4 | 2 |
| MCU | HD | 15 | 4 | 6 | 0.4 | 2 |
| MCU | HD | 66 | 2 | 3 | 0.4 | 2 |
| MCU | HD | 89 | 2 | 3 | 0.4 | 2 |
| MCU | HD | 13 | 6 | 7 | 0.461538462 | 2 |
| MCU | HD | 12 | 3 | 3 | 0.5 | 2 |
| MCU | HD | 33 | 4 | 4 | 0.5 | 2 |
| MCU | HD | 17 | 4 | 3 | 0.571428571 | 2 |
| MCU | HD | 21 | 4 | 3 | 0.571428571 | 2 |
| MCU | HD | 23 | 13 | 6 | 0.684210526 | 2 |
| MCU | HD | 53 | 7 | 3 | 0.7 | 2 |
| MCU | HD | 71 | 5 | 2 | 0.714285714 | 2 |
| MCU | HD | 22 | 6 | 2 | 0.75 | 2 |
| MCU | HD | 80 | 10 | 3 | 0.769230769 | 2 |
| MCU | HD | 45 | 10 | 2 | 0.833333333 | 2 |
| MCU | HD | 86 | 9 | 1 | 0.9 | 2 |
| MCU | HD | 68 | 6 | 0 | 1 | 2 |
| MB | HD | 101 | 0 | 7 | 0 | 2 |
| MB | HD | 118 | 0 | 10 | 0 | 2 |
| MB | HD | 128 | 0 | 8 | 0 | 2 |
| MB | HD | 147 | 0 | 4 | 0 | 2 |
| MB | HD | 165 | 0 | 3 | 0 | 2 |
| MB | HD | 190 | 0 | 2 | 0 | 2 |
| MB | HD | 195 | 0 | 2 | 0 | 2 |
| MB | HD | 108 | 1 | 7 | 0.125 | 2 |
| MB | HD | 133 | 1 | 5 | 0.166666667 | 2 |
| MB | HD | 153 | 2 | 8 | 0.2 | 2 |
| MB | HD | 178 | 1 | 4 | 0.2 | 2 |
| MB | HD | 176 | 2 | 7 | 0.222222222 | 2 |
| MB | HD | 140 | 1 | 3 | 0.25 | 2 |
| MB | HD | 175 | 1 | 2 | 0.333333333 | 2 |
| MB | HD | 130 | 2 | 3 | 0.4 | 2 |
| MB | HD | 170 | 2 | 3 | 0.4 | 2 |
| MB | HD | 100 | 3 | 4 | 0.428571429 | 2 |
| MB | HD | 184 | 5 | 6 | 0.454545455 | 2 |
| MB | HD | 181 | 6 | 7 | 0.461538462 | 2 |
| MB | HD | 109 | 2 | 2 | 0.5 | 2 |
| MB | HD | 111 | 5 | 5 | 0.5 | 2 |
| MB | HD | 114 | 3 | 3 | 0.5 | 2 |
| MB | HD | 116 | 1 | 1 | 0.5 | 2 |
| MB | HD | 121 | 2 | 2 | 0.5 | 2 |
| MB | HD | 126 | 1 | 1 | 0.5 | 2 |
| MB | HD | 138 | 2 | 2 | 0.5 | 2 |
| MB | HD | 142 | 2 | 2 | 0.5 | 2 |
| MB | HD | 166 | 1 | 1 | 0.5 | 2 |
| MB | HD | 185 | 1 | 1 | 0.5 | 2 |
| MB | HD | 122 | 5 | 4 | 0.555555556 | 2 |
| MB | HD | 161 | 8 | 5 | 0.615384615 | 2 |
| MB | HD | 177 | 5 | 3 | 0.625 | 2 |
| MB | HD | 179 | 5 | 3 | 0.625 | 2 |
| MB | HD | 148 | 7 | 4 | 0.636363636 | 2 |
| MB | HD | 124 | 4 | 2 | 0.666666667 | 2 |
| MB | HD | 129 | 4 | 2 | 0.666666667 | 2 |
| MB | HD | 154 | 2 | 1 | 0.666666667 | 2 |
| MB | HD | 135 | 7 | 3 | 0.7 | 2 |
| MB | HD | 164 | 5 | 2 | 0.714285714 | 2 |
| MB | HD | 117 | 14 | 4 | 0.777777778 | 2 |
| MB | HD | 112 | 4 | 1 | 0.8 | 2 |
| MB | HD | 151 | 9 | 2 | 0.818181818 | 2 |
| MB | HD | 110 | 8 | 1 | 0.888888889 | 2 |
| MB | HD | 103 | 7 | 0 | 1 | 2 |
| MB | HD | 146 | 1 | 0 | 1 | 2 |
| MB | HD | 180 | 1 | 0 | 1 | 2 |
| MB | LD | 301 | 0 | 3 | 0 | 2 |
| MB | LD | 306 | 0 | 7 | 0 | 2 |
| MB | LD | 308 | 0 | 9 | 0 | 2 |
| MB | LD | 334 | 0 | 7 | 0 | 2 |
| MB | LD | 356 | 0 | 4 | 0 | 2 |
| MB | LD | 367 | 0 | 10 | 0 | 2 |
| MB | LD | 376 | 0 | 8 | 0 | 2 |
| MB | LD | 380 | 0 | 9 | 0 | 2 |
| MB | LD | 389 | 0 | 13 | 0 | 2 |
| MB | LD | 391 | 0 | 2 | 0 | 2 |
| MB | LD | 400 | 0 | 3 | 0 | 2 |
| MB | LD | 359 | 2 | 16 | 0.111111111 | 2 |
| MB | LD | 352 | 1 | 7 | 0.125 | 2 |
| MB | LD | 375 | 1 | 7 | 0.125 | 2 |
| MB | LD | 333 | 1 | 6 | 0.142857143 | 2 |
| MB | LD | 303 | 2 | 10 | 0.166666667 | 2 |
| MB | LD | 397 | 1 | 4 | 0.2 | 2 |
| MB | LD | 398 | 2 | 7 | 0.222222222 | 2 |
| MB | LD | 310 | 1 | 3 | 0.25 | 2 |
| MB | LD | 317 | 1 | 3 | 0.25 | 2 |
| MB | LD | 320 | 2 | 6 | 0.25 | 2 |
| MB | LD | 321 | 2 | 6 | 0.25 | 2 |
| MB | LD | 351 | 3 | 9 | 0.25 | 2 |
| MB | LD | 372 | 1 | 3 | 0.25 | 2 |
| MB | LD | 390 | 2 | 6 | 0.25 | 2 |
| MB | LD | 396 | 2 | 6 | 0.25 | 2 |
| MB | LD | 358 | 4 | 11 | 0.266666667 | 2 |
| MB | LD | 325 | 1 | 2 | 0.333333333 | 2 |
| MB | LD | 327 | 1 | 2 | 0.333333333 | 2 |
| MB | LD | 316 | 3 | 5 | 0.375 | 2 |
| MB | LD | 302 | 2 | 3 | 0.4 | 2 |
| MB | LD | 330 | 4 | 6 | 0.4 | 2 |
| MB | LD | 346 | 3 | 4 | 0.428571429 | 2 |
| MB | LD | 311 | 4 | 5 | 0.444444444 | 2 |
| MB | LD | 377 | 4 | 5 | 0.444444444 | 2 |
| MB | LD | 322 | 4 | 4 | 0.5 | 2 |
| MB | LD | 329 | 3 | 3 | 0.5 | 2 |
| MB | LD | 341 | 4 | 4 | 0.5 | 2 |
| MB | LD | 361 | 4 | 4 | 0.5 | 2 |
| MB | LD | 314 | 3 | 2 | 0.6 | 2 |
| MB | LD | 369 | 6 | 4 | 0.6 | 2 |
| MB | LD | 344 | 7 | 3 | 0.7 | 2 |
| MB | LD | 319 | 3 | 1 | 0.75 | 2 |
| MB | LD | 315 | 6 | 0 | 1 | 2 |
| MB | LD | 357 | 10 | 0 | 1 | 2 |
| MB | LD | 363 | 8 | 0 | 1 | 2 |
| MCU | HD | 2 | 0 | 3 | 0 | 3 |
| MCU | HD | 8 | 0 | 11 | 0 | 3 |
| MCU | HD | 9 | 0 | 5 | 0 | 3 |
| MCU | HD | 10 | 0 | 15 | 0 | 3 |
| MCU | HD | 13 | 0 | 5 | 0 | 3 |
| MCU | HD | 22 | 0 | 14 | 0 | 3 |
| MCU | HD | 29 | 0 | 11 | 0 | 3 |
| MCU | HD | 30 | 0 | 10 | 0 | 3 |
| MCU | HD | 31 | 0 | 21 | 0 | 3 |
| MCU | HD | 36 | 0 | 12 | 0 | 3 |
| MCU | HD | 44 | 0 | 27 | 0 | 3 |
| MCU | HD | 45 | 0 | 17 | 0 | 3 |
| MCU | HD | 55 | 0 | 20 | 0 | 3 |
| MCU | HD | 57 | 0 | 9 | 0 | 3 |
| MCU | HD | 63 | 0 | 21 | 0 | 3 |
| MCU | HD | 64 | 0 | 6 | 0 | 3 |
| MCU | HD | 68 | 0 | 12 | 0 | 3 |
| MCU | HD | 25 | 1 | 17 | 0.055555556 | 3 |
| MCU | HD | 78 | 1 | 12 | 0.076923077 | 3 |
| MCU | HD | 76 | 1 | 9 | 0.1 | 3 |
| MCU | HD | 28 | 1 | 7 | 0.125 | 3 |
| MCU | HD | 70 | 3 | 14 | 0.176470588 | 3 |
| MCU | HD | 53 | 2 | 7 | 0.222222222 | 3 |
| MCU | HD | 49 | 6 | 13 | 0.315789474 | 3 |
| MCU | HD | 3 | 3 | 6 | 0.333333333 | 3 |
| MCU | HD | 7 | 4 | 5 | 0.444444444 | 3 |
| MCU | HD | 39 | 8 | 6 | 0.571428571 | 3 |
| MCU | HD | 50 | 7 | 5 | 0.583333333 | 3 |
| MCU | HD | 6 | 13 | 1 | 0.928571429 | 3 |
| MCU | LD | 200 | 0 | 8 | 0 | 3 |
| MCU | LD | 204 | 0 | 3 | 0 | 3 |
| MCU | LD | 207 | 0 | 10 | 0 | 3 |
| MCU | LD | 223 | 0 | 4 | 0 | 3 |
| MCU | LD | 230 | 0 | 5 | 0 | 3 |
| MCU | LD | 234 | 0 | 13 | 0 | 3 |
| MCU | LD | 237 | 0 | 10 | 0 | 3 |
| MCU | LD | 240 | 0 | 1 | 0 | 3 |
| MCU | LD | 245 | 0 | 8 | 0 | 3 |
| MCU | LD | 254 | 0 | 25 | 0 | 3 |
| MCU | LD | 258 | 0 | 7 | 0 | 3 |
| MCU | LD | 286 | 0 | 10 | 0 | 3 |
| MCU | LD | 298 | 0 | 17 | 0 | 3 |
| MCU | LD | 205 | 1 | 18 | 0.052631579 | 3 |
| MCU | LD | 259 | 1 | 15 | 0.0625 | 3 |
| MCU | LD | 220 | 1 | 14 | 0.066666667 | 3 |
| MCU | LD | 261 | 1 | 14 | 0.066666667 | 3 |
| MCU | LD | 280 | 1 | 11 | 0.083333333 | 3 |
| MCU | LD | 296 | 1 | 11 | 0.083333333 | 3 |
| MCU | LD | 239 | 1 | 10 | 0.090909091 | 3 |
| MCU | LD | 252 | 2 | 18 | 0.1 | 3 |
| MCU | LD | 281 | 1 | 9 | 0.1 | 3 |
| MCU | LD | 294 | 1 | 9 | 0.1 | 3 |
| MCU | LD | 226 | 1 | 8 | 0.111111111 | 3 |
| MCU | LD | 238 | 1 | 8 | 0.111111111 | 3 |
| MCU | LD | 299 | 1 | 8 | 0.111111111 | 3 |
| MCU | LD | 232 | 3 | 20 | 0.130434783 | 3 |
| MCU | LD | 206 | 2 | 13 | 0.133333333 | 3 |
| MCU | LD | 269 | 2 | 13 | 0.133333333 | 3 |
| MCU | LD | 271 | 2 | 13 | 0.133333333 | 3 |
| MCU | LD | 222 | 2 | 10 | 0.166666667 | 3 |
| MCU | LD | 233 | 2 | 10 | 0.166666667 | 3 |
| MCU | LD | 272 | 2 | 8 | 0.2 | 3 |
| MCU | LD | 273 | 1 | 4 | 0.2 | 3 |
| MCU | LD | 257 | 3 | 11 | 0.214285714 | 3 |
| MCU | LD | 295 | 2 | 7 | 0.222222222 | 3 |
| MCU | LD | 216 | 5 | 17 | 0.227272727 | 3 |
| MCU | LD | 213 | 3 | 10 | 0.230769231 | 3 |
| MCU | LD | 253 | 5 | 16 | 0.238095238 | 3 |
| MCU | LD | 210 | 1 | 3 | 0.25 | 3 |
| MCU | LD | 244 | 2 | 6 | 0.25 | 3 |
| MCU | LD | 260 | 3 | 8 | 0.272727273 | 3 |
| MCU | LD | 292 | 3 | 8 | 0.272727273 | 3 |
| MCU | LD | 209 | 2 | 5 | 0.285714286 | 3 |
| MCU | LD | 225 | 3 | 7 | 0.3 | 3 |
| MCU | LD | 249 | 4 | 9 | 0.307692308 | 3 |
| MCU | LD | 202 | 4 | 8 | 0.333333333 | 3 |
| MCU | LD | 231 | 3 | 6 | 0.333333333 | 3 |
| MCU | LD | 282 | 3 | 6 | 0.333333333 | 3 |
| MCU | LD | 242 | 9 | 15 | 0.375 | 3 |
| MCU | LD | 247 | 3 | 5 | 0.375 | 3 |
| MCU | LD | 289 | 7 | 9 | 0.4375 | 3 |
| MCU | LD | 277 | 5 | 6 | 0.454545455 | 3 |
| MCU | LD | 217 | 2 | 2 | 0.5 | 3 |
| MCU | LD | 241 | 7 | 5 | 0.583333333 | 3 |
| MCU | LD | 243 | 11 | 7 | 0.611111111 | 3 |
| MCU | LD | 235 | 5 | 3 | 0.625 | 3 |
| MCU | LD | 208 | 11 | 6 | 0.647058824 | 3 |
| MCU | LD | 211 | 6 | 3 | 0.666666667 | 3 |
| MCU | LD | 246 | 4 | 2 | 0.666666667 | 3 |
| MCU | LD | 278 | 8 | 4 | 0.666666667 | 3 |
| MCU | LD | 250 | 13 | 4 | 0.764705882 | 3 |
| MCU | LD | 279 | 10 | 3 | 0.769230769 | 3 |
| MCU | LD | 221 | 7 | 1 | 0.875 | 3 |
| MCU | LD | 283 | 17 | 2 | 0.894736842 | 3 |
| MB | HD | 106 | 0 | 11 | 0 | 3 |
| MB | HD | 124 | 0 | 14 | 0 | 3 |
| MB | HD | 138 | 0 | 11 | 0 | 3 |
| MB | HD | 142 | 0 | 9 | 0 | 3 |
| MB | HD | 159 | 0 | 6 | 0 | 3 |
| MB | HD | 181 | 0 | 7 | 0 | 3 |
| MB | HD | 196 | 0 | 1 | 0 | 3 |
| MB | HD | 176 | 1 | 18 | 0.052631579 | 3 |
| MB | HD | 164 | 1 | 14 | 0.066666667 | 3 |
| MB | HD | 125 | 1 | 9 | 0.1 | 3 |
| MB | HD | 153 | 2 | 18 | 0.1 | 3 |
| MB | HD | 165 | 2 | 17 | 0.105263158 | 3 |
| MB | HD | 190 | 1 | 8 | 0.111111111 | 3 |
| MB | HD | 184 | 2 | 13 | 0.133333333 | 3 |
| MB | HD | 197 | 2 | 11 | 0.153846154 | 3 |
| MB | HD | 120 | 3 | 16 | 0.157894737 | 3 |
| MB | HD | 154 | 2 | 9 | 0.181818182 | 3 |
| MB | HD | 117 | 1 | 3 | 0.25 | 3 |
| MB | HD | 136 | 3 | 9 | 0.25 | 3 |
| MB | HD | 161 | 2 | 6 | 0.25 | 3 |
| MB | HD | 170 | 1 | 3 | 0.25 | 3 |
| MB | HD | 150 | 4 | 11 | 0.266666667 | 3 |
| MB | HD | 123 | 2 | 5 | 0.285714286 | 3 |
| MB | HD | 194 | 2 | 5 | 0.285714286 | 3 |
| MB | HD | 178 | 3 | 7 | 0.3 | 3 |
| MB | HD | 111 | 5 | 11 | 0.3125 | 3 |
| MB | HD | 168 | 5 | 9 | 0.357142857 | 3 |
| MB | HD | 110 | 3 | 5 | 0.375 | 3 |
| MB | HD | 135 | 4 | 6 | 0.4 | 3 |
| MB | HD | 132 | 9 | 13 | 0.409090909 | 3 |
| MB | HD | 148 | 5 | 7 | 0.416666667 | 3 |
| MB | HD | 185 | 5 | 6 | 0.454545455 | 3 |
| MB | HD | 155 | 9 | 9 | 0.5 | 3 |
| MB | HD | 107 | 8 | 6 | 0.571428571 | 3 |
| MB | HD | 156 | 6 | 4 | 0.6 | 3 |
| MB | HD | 172 | 10 | 4 | 0.714285714 | 3 |
| MB | HD | 122 | 13 | 4 | 0.764705882 | 3 |
| MB | HD | 105 | 11 | 3 | 0.785714286 | 3 |
| MB | HD | 188 | 4 | 1 | 0.8 | 3 |
| MB | HD | 180 | 17 | 1 | 0.944444444 | 3 |
| MB | LD | 303 | 0 | 2 | 0 | 3 |
| MB | LD | 307 | 0 | 7 | 0 | 3 |
| MB | LD | 318 | 0 | 13 | 0 | 3 |
| MB | LD | 320 | 0 | 3 | 0 | 3 |
| MB | LD | 326 | 0 | 5 | 0 | 3 |
| MB | LD | 356 | 0 | 5 | 0 | 3 |
| MB | LD | 358 | 0 | 17 | 0 | 3 |
| MB | LD | 366 | 0 | 4 | 0 | 3 |
| MB | LD | 370 | 0 | 11 | 0 | 3 |
| MB | LD | 372 | 0 | 10 | 0 | 3 |
| MB | LD | 375 | 0 | 7 | 0 | 3 |
| MB | LD | 378 | 0 | 24 | 0 | 3 |
| MB | LD | 381 | 0 | 2 | 0 | 3 |
| MB | LD | 390 | 0 | 11 | 0 | 3 |
| MB | LD | 397 | 1 | 20 | 0.047619048 | 3 |
| MB | LD | 389 | 1 | 19 | 0.05 | 3 |
| MB | LD | 392 | 1 | 18 | 0.052631579 | 3 |
| MB | LD | 387 | 1 | 12 | 0.076923077 | 3 |
| MB | LD | 349 | 2 | 17 | 0.105263158 | 3 |
| MB | LD | 300 | 1 | 7 | 0.125 | 3 |
| MB | LD | 357 | 1 | 7 | 0.125 | 3 |
| MB | LD | 368 | 2 | 14 | 0.125 | 3 |
| MB | LD | 317 | 2 | 12 | 0.142857143 | 3 |
| MB | LD | 327 | 2 | 12 | 0.142857143 | 3 |
| MB | LD | 386 | 3 | 16 | 0.157894737 | 3 |
| MB | LD | 394 | 2 | 10 | 0.166666667 | 3 |
| MB | LD | 308 | 1 | 4 | 0.2 | 3 |
| MB | LD | 311 | 2 | 8 | 0.2 | 3 |
| MB | LD | 323 | 2 | 8 | 0.2 | 3 |
| MB | LD | 355 | 2 | 8 | 0.2 | 3 |
| MB | LD | 388 | 3 | 12 | 0.2 | 3 |
| MB | LD | 306 | 2 | 7 | 0.222222222 | 3 |
| MB | LD | 315 | 4 | 14 | 0.222222222 | 3 |
| MB | LD | 362 | 2 | 7 | 0.222222222 | 3 |
| MB | LD | 316 | 4 | 13 | 0.235294118 | 3 |
| MB | LD | 379 | 2 | 6 | 0.25 | 3 |
| MB | LD | 348 | 5 | 13 | 0.277777778 | 3 |
| MB | LD | 313 | 4 | 10 | 0.285714286 | 3 |
| MB | LD | 324 | 4 | 10 | 0.285714286 | 3 |
| MB | LD | 305 | 3 | 7 | 0.3 | 3 |
| MB | LD | 301 | 4 | 9 | 0.307692308 | 3 |
| MB | LD | 333 | 2 | 4 | 0.333333333 | 3 |
| MB | LD | 334 | 3 | 6 | 0.333333333 | 3 |
| MB | LD | 398 | 5 | 9 | 0.357142857 | 3 |
| MB | LD | 337 | 6 | 9 | 0.4 | 3 |
| MB | LD | 345 | 4 | 6 | 0.4 | 3 |
| MB | LD | 374 | 9 | 13 | 0.409090909 | 3 |
| MB | LD | 353 | 4 | 5 | 0.444444444 | 3 |
| MB | LD | 365 | 4 | 5 | 0.444444444 | 3 |
| MB | LD | 364 | 5 | 6 | 0.454545455 | 3 |
| MB | LD | 339 | 9 | 10 | 0.473684211 | 3 |
| MB | LD | 302 | 6 | 6 | 0.5 | 3 |
| MB | LD | 304 | 4 | 4 | 0.5 | 3 |
| MB | LD | 384 | 5 | 5 | 0.5 | 3 |
| MB | LD | 396 | 8 | 8 | 0.5 | 3 |
| MB | LD | 371 | 9 | 8 | 0.529411765 | 3 |
| MB | LD | 332 | 12 | 10 | 0.545454545 | 3 |
| MB | LD | 354 | 4 | 3 | 0.571428571 | 3 |
| MB | LD | 361 | 8 | 5 | 0.615384615 | 3 |
| MB | LD | 314 | 5 | 3 | 0.625 | 3 |
| MB | LD | 338 | 5 | 3 | 0.625 | 3 |
| MB | LD | 328 | 9 | 5 | 0.642857143 | 3 |
| MB | LD | 400 | 2 | 1 | 0.666666667 | 3 |
| MB | LD | 393 | 7 | 3 | 0.7 | 3 |
| MB | LD | 399 | 12 | 5 | 0.705882353 | 3 |
| MB | LD | 319 | 14 | 5 | 0.736842105 | 3 |
| MB | LD | 344 | 3 | 1 | 0.75 | 3 |
| MB | LD | 325 | 10 | 3 | 0.769230769 | 3 |
| MB | LD | 382 | 10 | 3 | 0.769230769 | 3 |
| MB | LD | 312 | 14 | 4 | 0.777777778 | 3 |
| MCU | HD | 2 | 0 | 13 | 0 | 4 |
| MCU | HD | 3 | 0 | 1 | 0 | 4 |
| MCU | HD | 4 | 0 | 12 | 0 | 4 |
| MCU | HD | 5 | 0 | 8 | 0 | 4 |
| MCU | HD | 6 | 0 | 4 | 0 | 4 |
| MCU | HD | 7 | 0 | 9 | 0 | 4 |
| MCU | HD | 8 | 0 | 13 | 0 | 4 |
| MCU | HD | 9 | 0 | 1 | 0 | 4 |
| MCU | HD | 10 | 0 | 16 | 0 | 4 |
| MCU | HD | 11 | 0 | 25 | 0 | 4 |
| MCU | HD | 12 | 0 | 12 | 0 | 4 |
| MCU | HD | 13 | 0 | 16 | 0 | 4 |
| MCU | HD | 14 | 0 | 14 | 0 | 4 |
| MCU | HD | 15 | 0 | 15 | 0 | 4 |
| MCU | HD | 16 | 0 | 17 | 0 | 4 |
| MCU | HD | 17 | 0 | 11 | 0 | 4 |
| MCU | HD | 18 | 0 | 16 | 0 | 4 |
| MCU | HD | 19 | 0 | 23 | 0 | 4 |
| MB | HD | 20 | 0 | 11 | 0 | 4 |
| MB | HD | 21 | 0 | 15 | 0 | 4 |
| MB | HD | 22 | 0 | 18 | 0 | 4 |
| MB | HD | 23 | 0 | 9 | 0 | 4 |
| MB | HD | 24 | 0 | 16 | 0 | 4 |
| MB | HD | 25 | 0 | 16 | 0 | 4 |
| MB | HD | 26 | 0 | 14 | 0 | 4 |
| MB | HD | 27 | 0 | 8 | 0 | 4 |
| MB | HD | 28 | 0 | 9 | 0 | 4 |
| MB | HD | 29 | 0 | 10 | 0 | 4 |
| MB | HD | 30 | 0 | 10 | 0 | 4 |
| MB | HD | 31 | 0 | 13 | 0 | 4 |
| MCU | LD | 32 | 0 | 8 | 0 | 4 |
| MCU | LD | 33 | 0 | 6 | 0 | 4 |
| MCU | LD | 34 | 0 | 8 | 0 | 4 |
| MCU | LD | 35 | 0 | 11 | 0 | 4 |
| MCU | LD | 36 | 0 | 7 | 0 | 4 |
| MB | LD | 37 | 0 | 5 | 0 | 4 |
| MB | LD | 38 | 0 | 4 | 0 | 4 |
| MB | LD | 39 | 0 | 6 | 0 | 4 |
| MB | LD | 40 | 0 | 16 | 0 | 4 |
| MB | LD | 41 | 0 | 13 | 0 | 4 |
| MB | LD | 42 | 0 | 11 | 0 | 4 |
| MB | LD | 43 | 0 | 12 | 0 | 4 |
| MCU | HD | 44 | 1 | 23 | 0.04166667 | 4 |
| MCU | LD | 45 | 1 | 20 | 0.04761905 | 4 |
| MB | HD | 46 | 1 | 18 | 0.05263158 | 4 |
| MCU | LD | 47 | 1 | 17 | 0.05555556 | 4 |
| MCU | LD | 48 | 1 | 13 | 0.07142857 | 4 |
| MCU | LD | 49 | 1 | 13 | 0.07142857 | 4 |
| MB | HD | 50 | 1 | 10 | 0.09090909 | 4 |
| MB | HD | 51 | 1 | 9 | 0.1 | 4 |
| MCU | LD | 52 | 1 | 9 | 0.1 | 4 |
| MCU | LD | 53 | 1 | 9 | 0.1 | 4 |
| MCU | LD | 54 | 1 | 9 | 0.1 | 4 |
| MCU | LD | 55 | 1 | 8 | 0.11111111 | 4 |
| MB | LD | 56 | 1 | 8 | 0.11111111 | 4 |
| MCU | HD | 57 | 2 | 15 | 0.11764706 | 4 |
| MB | HD | 58 | 1 | 7 | 0.125 | 4 |
| MCU | LD | 59 | 1 | 7 | 0.125 | 4 |
| MB | HD | 60 | 1 | 6 | 0.14285714 | 4 |
| MCU | LD | 61 | 1 | 6 | 0.14285714 | 4 |
| MB | LD | 62 | 1 | 6 | 0.14285714 | 4 |
| MB | LD | 63 | 1 | 6 | 0.14285714 | 4 |
| MB | LD | 64 | 1 | 6 | 0.14285714 | 4 |
| MCU | HD | 65 | 2 | 11 | 0.15384615 | 4 |
| MB | LD | 66 | 2 | 11 | 0.15384615 | 4 |
| MCU | HD | 67 | 3 | 15 | 0.16666667 | 4 |
| MCU | HD | 68 | 2 | 10 | 0.16666667 | 4 |
| MCU | HD | 69 | 2 | 10 | 0.16666667 | 4 |
| MCU | LD | 70 | 2 | 10 | 0.16666667 | 4 |
| MB | LD | 71 | 2 | 10 | 0.16666667 | 4 |
| MB | LD | 72 | 1 | 5 | 0.16666667 | 4 |
| MB | LD | 73 | 2 | 10 | 0.16666667 | 4 |
| MCU | LD | 74 | 2 | 9 | 0.18181818 | 4 |
| MB | LD | 75 | 2 | 9 | 0.18181818 | 4 |
| MB | HD | 76 | 2 | 8 | 0.2 | 4 |
| MB | HD | 77 | 2 | 8 | 0.2 | 4 |
| MCU | LD | 78 | 2 | 8 | 0.2 | 4 |
| MB | LD | 79 | 2 | 8 | 0.2 | 4 |
| MB | LD | 80 | 1 | 4 | 0.2 | 4 |
| MB | LD | 81 | 2 | 8 | 0.2 | 4 |
| MB | HD | 82 | 3 | 11 | 0.21428571 | 4 |
| MB | HD | 83 | 3 | 11 | 0.21428571 | 4 |
| MB | LD | 84 | 3 | 11 | 0.21428571 | 4 |
| MCU | LD | 85 | 2 | 7 | 0.22222222 | 4 |
| MCU | LD | 86 | 4 | 14 | 0.22222222 | 4 |
| MB | HD | 87 | 3 | 10 | 0.23076923 | 4 |
| MCU | HD | 88 | 4 | 12 | 0.25 | 4 |
| MB | HD | 89 | 2 | 6 | 0.25 | 4 |
| MB | HD | 90 | 2 | 6 | 0.25 | 4 |
| MB | HD | 91 | 3 | 9 | 0.25 | 4 |
| MCU | LD | 92 | 2 | 6 | 0.25 | 4 |
| MCU | LD | 93 | 2 | 6 | 0.25 | 4 |
| MCU | LD | 94 | 3 | 9 | 0.25 | 4 |
| MCU | LD | 95 | 1 | 3 | 0.25 | 4 |
| MB | LD | 96 | 2 | 6 | 0.25 | 4 |
| MCU | HD | 97 | 4 | 11 | 0.26666667 | 4 |
| MCU | HD | 98 | 4 | 11 | 0.26666667 | 4 |
| MB | HD | 99 | 3 | 8 | 0.27272727 | 4 |
| MB | HD | 100 | 3 | 8 | 0.27272727 | 4 |
| MCU | LD | 101 | 3 | 8 | 0.27272727 | 4 |
| MCU | HD | 102 | 5 | 13 | 0.27777778 | 4 |
| MCU | HD | 103 | 2 | 5 | 0.28571429 | 4 |
| MB | HD | 104 | 5 | 12 | 0.29411765 | 4 |
| MCU | LD | 105 | 3 | 7 | 0.3 | 4 |
| MB | LD | 106 | 3 | 7 | 0.3 | 4 |
| MCU | HD | 107 | 7 | 16 | 0.30434783 | 4 |
| MB | HD | 108 | 5 | 11 | 0.3125 | 4 |
| MCU | LD | 109 | 5 | 11 | 0.3125 | 4 |
| MCU | LD | 110 | 5 | 11 | 0.3125 | 4 |
| MCU | LD | 111 | 5 | 11 | 0.3125 | 4 |
| MB | LD | 112 | 5 | 11 | 0.3125 | 4 |
| MCU | LD | 113 | 6 | 13 | 0.31578947 | 4 |
| MB | HD | 114 | 1 | 2 | 0.33333333 | 4 |
| MCU | LD | 115 | 2 | 4 | 0.33333333 | 4 |
| MB | LD | 116 | 4 | 8 | 0.33333333 | 4 |
| MB | LD | 117 | 4 | 8 | 0.33333333 | 4 |
| MB | LD | 118 | 4 | 8 | 0.33333333 | 4 |
| MCU | LD | 119 | 7 | 13 | 0.35 | 4 |
| MB | LD | 120 | 7 | 13 | 0.35 | 4 |
| MB | HD | 121 | 3 | 5 | 0.375 | 4 |
| MCU | LD | 122 | 3 | 5 | 0.375 | 4 |
| MB | LD | 123 | 3 | 5 | 0.375 | 4 |
| MB | LD | 124 | 3 | 5 | 0.375 | 4 |
| MB | LD | 125 | 8 | 13 | 0.38095238 | 4 |
| MCU | LD | 126 | 5 | 8 | 0.38461538 | 4 |
| MCU | HD | 127 | 8 | 12 | 0.4 | 4 |
| MB | HD | 128 | 2 | 3 | 0.4 | 4 |
| MB | HD | 129 | 4 | 6 | 0.4 | 4 |
| MB | HD | 130 | 6 | 9 | 0.4 | 4 |
| MB | HD | 131 | 2 | 3 | 0.4 | 4 |
| MCU | LD | 132 | 2 | 3 | 0.4 | 4 |
| MCU | LD | 133 | 5 | 7 | 0.41666667 | 4 |
| MCU | LD | 134 | 5 | 7 | 0.41666667 | 4 |
| MB | HD | 135 | 6 | 8 | 0.42857143 | 4 |
| MB | HD | 136 | 3 | 4 | 0.42857143 | 4 |
| MCU | LD | 137 | 6 | 8 | 0.42857143 | 4 |
| MCU | LD | 138 | 6 | 8 | 0.42857143 | 4 |
| MB | LD | 139 | 3 | 4 | 0.42857143 | 4 |
| MB | HD | 140 | 8 | 10 | 0.44444444 | 4 |
| MCU | LD | 141 | 4 | 5 | 0.44444444 | 4 |
| MB | LD | 142 | 12 | 15 | 0.44444444 | 4 |
| MCU | LD | 143 | 9 | 11 | 0.45 | 4 |
| MCU | HD | 144 | 5 | 6 | 0.45454545 | 4 |
| MCU | HD | 145 | 6 | 7 | 0.46153846 | 4 |
| MCU | LD | 146 | 6 | 7 | 0.46153846 | 4 |
| MCU | LD | 147 | 7 | 8 | 0.46666667 | 4 |
| MB | LD | 148 | 8 | 9 | 0.47058824 | 4 |
| MCU | HD | 149 | 2 | 2 | 0.5 | 4 |
| MCU | HD | 150 | 8 | 8 | 0.5 | 4 |
| MCU | HD | 151 | 2 | 2 | 0.5 | 4 |
| MCU | HD | 152 | 3 | 3 | 0.5 | 4 |
| MB | HD | 153 | 5 | 5 | 0.5 | 4 |
| MCU | LD | 154 | 5 | 5 | 0.5 | 4 |
| MCU | LD | 155 | 8 | 8 | 0.5 | 4 |
| MB | LD | 156 | 3 | 3 | 0.5 | 4 |
| MB | LD | 157 | 5 | 5 | 0.5 | 4 |
| MB | LD | 158 | 5 | 5 | 0.5 | 4 |
| MB | LD | 159 | 2 | 2 | 0.5 | 4 |
| MB | LD | 160 | 8 | 7 | 0.53333333 | 4 |
| MB | LD | 161 | 7 | 6 | 0.53846154 | 4 |
| MB | LD | 162 | 6 | 5 | 0.54545455 | 4 |
| MCU | LD | 163 | 5 | 4 | 0.55555556 | 4 |
| MCU | HD | 164 | 9 | 7 | 0.5625 | 4 |
| MCU | LD | 165 | 4 | 3 | 0.57142857 | 4 |
| MCU | LD | 166 | 8 | 6 | 0.57142857 | 4 |
| MCU | LD | 167 | 7 | 5 | 0.58333333 | 4 |
| MB | LD | 168 | 7 | 5 | 0.58333333 | 4 |
| MB | LD | 169 | 12 | 8 | 0.6 | 4 |
| MB | LD | 170 | 8 | 5 | 0.61538462 | 4 |
| MB | HD | 171 | 5 | 3 | 0.625 | 4 |
| MB | LD | 172 | 5 | 3 | 0.625 | 4 |
| MB | LD | 173 | 5 | 3 | 0.625 | 4 |
| MCU | LD | 174 | 14 | 8 | 0.63636364 | 4 |
| MB | HD | 175 | 11 | 6 | 0.64705882 | 4 |
| MB | LD | 176 | 11 | 6 | 0.64705882 | 4 |
| MB | HD | 177 | 2 | 1 | 0.66666667 | 4 |
| MCU | LD | 178 | 6 | 3 | 0.66666667 | 4 |
| MCU | LD | 179 | 2 | 1 | 0.66666667 | 4 |
| MB | LD | 180 | 6 | 3 | 0.66666667 | 4 |
| MB | LD | 181 | 2 | 1 | 0.66666667 | 4 |
| MB | LD | 182 | 7 | 3 | 0.7 | 4 |
| MB | LD | 183 | 13 | 5 | 0.72222222 | 4 |
| MB | HD | 184 | 8 | 3 | 0.72727273 | 4 |
| MB | HD | 185 | 6 | 2 | 0.75 | 4 |
| MCU | LD | 186 | 9 | 3 | 0.75 | 4 |
| MB | LD | 301 | 6 | 2 | 0.75 | 4 |
| MB | LD | 302 | 9 | 3 | 0.75 | 4 |
| MCU | LD | 303 | 7 | 2 | 0.77777778 | 4 |
| MCU | HD | 304 | 4 | 1 | 0.8 | 4 |
| MB | HD | 305 | 4 | 1 | 0.8 | 4 |
| MB | LD | 306 | 4 | 1 | 0.8 | 4 |
| MB | LD | 307 | 8 | 2 | 0.8 | 4 |
| MB | LD | 308 | 4 | 1 | 0.8 | 4 |
| MB | LD | 309 | 5 | 1 | 0.83333333 | 4 |
| MCU | LD | 310 | 6 | 1 | 0.85714286 | 4 |
| MCU | HD | 311 | 13 | 2 | 0.86666667 | 4 |
| MCU | HD | 312 | 8 | 1 | 0.88888889 | 4 |
| MB | HD | 313 | 11 | 1 | 0.91666667 | 4 |
| MB | HD | 314 | 11 | 1 | 0.91666667 | 4 |
| MB | HD | 315 | 133 | 12 | 0.91724138 | 4 |
| MB | LD | 316 | 14 | 1 | 0.93333333 | 4 |
| MB | HD | 317 | 18 | 1 | 0.94736842 | 4 |
| MCU | HD | 318 | 8 | 0 | 1 | 4 |
| MB | HD | 319 | 2 | 0 | 1 | 4 |
| MB | HD | 320 | 5 | 0 | 1 | 4 |
| MCU | LD | 321 | 5 |  | 1 | 4 |
| MCU | LD | 322 | 6 | 0 | 1 | 4 |
| MCU | LD | 323 | 20 | 0 | 1 | 4 |
| MB | LD | 324 | 9 | 0 | 1 | 4 |
| MB | LD | 325 | 4 | 0 | 1 | 4 |

Supplementary Table 5: Sperm offence assay, 4 day old males, non-transformed data with red and white eye values

| **Red** | **White** |  | **Selection** | **Treatment** | **P2** | **Block** |
| --- | --- | --- | --- | --- | --- | --- |
| 1 | 4 | 0.2 | MCU | HD | 0.463647609 | 1 |
| 1 | 1 | 0.5 | MCU | HD | 0.785398163 | 1 |
| 3 | 3 | 0.5 | MCU | HD | 0.785398163 | 1 |
| 5 | 4 | 0.555555556 | MCU | HD | 0.841068671 | 1 |
| 2 | 3 | 0.4 | MCU | HD | 0.684719203 | 1 |
| 5 | 4 | 0.555555556 | MCU | HD | 0.841068671 | 1 |
| 1 | 4 | 0.2 | MCU | HD | 0.463647609 | 1 |
| 10 | 1 | 0.909090909 | MCU | HD | 1.264518958 | 1 |
| 2 | 3 | 0.4 | MCU | HD | 0.684719203 | 1 |
| 6 | 2 | 0.75 | MCU | HD | 1.047197551 | 1 |
| 4 | 4 | 0.5 | MCU | HD | 0.785398163 | 1 |
| 4 | 2 | 0.666666667 | MCU | HD | 0.955316618 | 1 |
| 6 | 3 | 0.666666667 | MCU | HD | 0.955316618 | 1 |
| 1 | 3 | 0.25 | MCU | HD | 0.523598776 | 1 |
| 4 | 8 | 0.333333333 | MCU | HD | 0.615479709 | 1 |
| 1 | 6 | 0.142857143 | MCU | HD | 0.387596687 | 1 |
| 1 | 8 | 0.111111111 | MCU | HD | 0.339836909 | 1 |
| 5 | 2 | 0.714285714 | MCU | HD | 1.006853685 | 1 |
| 3 | 3 | 0.5 | MCU | HD | 0.785398163 | 1 |
| 2 | 9 | 0.181818182 | MCU | HD | 0.440510663 | 1 |
| 3 | 1 | 0.75 | MCU | HD | 1.047197551 | 1 |
| 3 | 3 | 0.5 | MCU | HD | 0.785398163 | 1 |
| 1 | 4 | 0.2 | MCU | HD | 0.463647609 | 1 |
| 1 | 6 | 0.142857143 | MCU | HD | 0.387596687 | 1 |
| 5 | 2 | 0.714285714 | MCU | HD | 1.006853685 | 1 |
| 3 | 1 | 0.75 | MCU | HD | 1.047197551 | 1 |
| 1 | 1 | 0.5 | MCU | HD | 0.785398163 | 1 |
| 2 | 6 | 0.25 | MCU | HD | 0.523598776 | 1 |
| 4 | 4 | 0.5 | MCU | HD | 0.785398163 | 1 |
| 1 | 3 | 0.25 | MCU | HD | 0.523598776 | 1 |
| 1 | 6 | 0.142857143 | MCU | HD | 0.387596687 | 1 |
| 3 | 8 | 0.272727273 | MCU | HD | 0.549467245 | 1 |
| 4 | 1 | 0.8 | MCU | HD | 1.107148718 | 1 |
| 6 | 2 | 0.75 | MCU | HD | 1.047197551 | 1 |
| 11 | 2 | 0.846153846 | MCU | HD | 1.167739252 | 1 |
| 5 | 4 | 0.555555556 | MCU | HD | 0.841068671 | 1 |
| 1 | 5 | 0.166666667 | MCU | HD | 0.420534335 | 1 |
| 2 | 2 | 0.5 | MCU | HD | 0.785398163 | 1 |
| 4 | 2 | 0.666666667 | MCU | HD | 0.955316618 | 1 |
| 1 | 5 | 0.166666667 | MCU | HD | 0.420534335 | 1 |
| 5 | 0 | 1 | MCU | HD | 1.570796327 | 1 |
| 3 | 2 | 0.6 | MCU | HD | 0.886077124 | 1 |
| 4 | 4 | 0.5 | MCU | HD | 0.785398163 | 1 |
| 9 | 1 | 0.9 | MCU | HD | 1.249045772 | 1 |
| 4 | 3 | 0.571428571 | MCU | HD | 0.857071948 | 1 |
| 1 | 6 | 0.142857143 | MCU | HD | 0.387596687 | 1 |
| 1 | 14 | 0.066666667 | MCU | HD | 0.261157411 | 1 |
| 1 | 1 | 0.5 | MCU | HD | 0.785398163 | 1 |
| 5 | 3 | 0.625 | MCU | HD | 0.911738291 | 1 |
| 3 | 1 | 0.75 | MCU | HD | 1.047197551 | 1 |
| 9 | 1 | 0.9 | MCU | HD | 1.249045772 | 1 |
| 9 | 1 | 0.9 | MCU | HD | 1.249045772 | 1 |
| 6 | 2 | 0.75 | MCU | HD | 1.047197551 | 1 |
| 10 | 3 | 0.769230769 | MCU | HD | 1.069703314 | 1 |
| 3 | 1 | 0.75 | MCU | HD | 1.047197551 | 1 |
| 4 | 1 | 0.8 | MCU | HD | 1.107148718 | 1 |
| 5 | 3 | 0.625 | MCU | HD | 0.911738291 | 1 |
| 1 | 2 | 0.333333333 | MCU | HD | 0.615479709 | 1 |
| 5 | 4 | 0.555555556 | MCU | HD | 0.841068671 | 1 |
| 9 | 2 | 0.818181818 | MCU | HD | 1.130285664 | 1 |
| 3 | 2 | 0.6 | MCU | HD | 0.886077124 | 1 |
| 2 | 2 | 0.5 | MCU | HD | 0.785398163 | 1 |
| 7 | 2 | 0.777777778 | MCU | HD | 1.079913649 | 1 |
| 5 | 2 | 0.714285714 | MCU | HD | 1.006853685 | 1 |
| 5 | 1 | 0.833333333 | MCU | HD | 1.150261992 | 1 |
| 6 | 3 | 0.666666667 | MCU | HD | 0.955316618 | 1 |
| 5 | 2 | 0.714285714 | MCU | HD | 1.006853685 | 1 |
| 3 | 5 | 0.375 | MCU | HD | 0.659058036 | 1 |
| 2 | 2 | 0.5 | MCU | HD | 0.785398163 | 1 |
| 3 | 9 | 0.25 | MCU | HD | 0.523598776 | 1 |
| 3 | 4 | 0.428571429 | MCU | HD | 0.713724379 | 1 |
| 4 | 6 | 0.4 | MCU | HD | 0.684719203 | 1 |
| 6 | 1 | 0.857142857 | MCU | HD | 1.18319964 | 1 |
| 4 | 4 | 0.5 | MCU | HD | 0.785398163 | 1 |
| 3 | 3 | 0.5 | MCU | HD | 0.785398163 | 1 |
| 3 | 3 | 0.5 | MCU | HD | 0.785398163 | 1 |
| 3 | 0 | 1 | MCU | HD | 1.570796327 | 1 |
| 4 | 3 | 0.571428571 | MCU | HD | 0.857071948 | 1 |
| 4 | 2 | 0.666666667 | MCU | HD | 0.955316618 | 1 |
| 5 | 3 | 0.625 | MCU | HD | 0.911738291 | 1 |
| 3 | 5 | 0.375 | MCU | HD | 0.659058036 | 1 |
| 2 | 9 | 0.181818182 | MCU | HD | 0.440510663 | 1 |
| 2 | 4 | 0.333333333 | MCU | HD | 0.615479709 | 1 |
| 6 | 3 | 0.666666667 | MCU | HD | 0.955316618 | 1 |
| 4 | 55 | 0.06779661 | MCU | HD | 0.263413502 | 1 |
| 2 | 7 | 0.222222222 | MCU | HD | 0.490882678 | 1 |
| 7 | 2 | 0.777777778 | MCU | HD | 1.079913649 | 1 |
| 1 | 9 | 0.1 | MCU | HD | 0.32175055 | 2 |
| 1 | 7 | 0.125 | MCU | HD | 0.36136712 | 2 |
| 1 | 6 | 0.14285714 | MCU | HD | 0.38759669 | 2 |
| 1 | 4 | 0.2 | MCU | HD | 0.46364761 | 2 |
| 2 | 7 | 0.22222222 | MCU | HD | 0.49088268 | 2 |
| 2 | 6 | 0.25 | MCU | HD | 0.52359878 | 2 |
| 2 | 6 | 0.25 | MCU | HD | 0.52359878 | 2 |
| 2 | 6 | 0.25 | MCU | HD | 0.52359878 | 2 |
| 4 | 12 | 0.25 | MCU | HD | 0.52359878 | 2 |
| 3 | 8 | 0.27272727 | MCU | HD | 0.54946724 | 2 |
| 2 | 5 | 0.28571429 | MCU | HD | 0.56394264 | 2 |
| 1 | 2 | 0.33333333 | MCU | HD | 0.61547971 | 2 |
| 4 | 7 | 0.36363636 | MCU | HD | 0.64728485 | 2 |
| 2 | 3 | 0.4 | MCU | HD | 0.6847192 | 2 |
| 2 | 3 | 0.4 | MCU | HD | 0.6847192 | 2 |
| 4 | 6 | 0.4 | MCU | HD | 0.6847192 | 2 |
| 4 | 6 | 0.4 | MCU | HD | 0.6847192 | 2 |
| 7 | 8 | 0.46666667 | MCU | HD | 0.75204009 | 2 |
| 1 | 1 | 0.5 | MCU | HD | 0.78539816 | 2 |
| 1 | 1 | 0.5 | MCU | HD | 0.78539816 | 2 |
| 1 | 1 | 0.5 | MCU | HD | 0.78539816 | 2 |
| 2 | 2 | 0.5 | MCU | HD | 0.78539816 | 2 |
| 2 | 2 | 0.5 | MCU | HD | 0.78539816 | 2 |
| 2 | 2 | 0.5 | MCU | HD | 0.78539816 | 2 |
| 3 | 3 | 0.5 | MCU | HD | 0.78539816 | 2 |
| 3 | 3 | 0.5 | MCU | HD | 0.78539816 | 2 |
| 3 | 3 | 0.5 | MCU | HD | 0.78539816 | 2 |
| 4 | 4 | 0.5 | MCU | HD | 0.78539816 | 2 |
| 4 | 4 | 0.5 | MCU | HD | 0.78539816 | 2 |
| 5 | 5 | 0.5 | MCU | HD | 0.78539816 | 2 |
| 8 | 7 | 0.53333333 | MCU | HD | 0.81875624 | 2 |
| 5 | 4 | 0.55555556 | MCU | HD | 0.84106867 | 2 |
| 4 | 3 | 0.57142857 | MCU | HD | 0.85707195 | 2 |
| 4 | 3 | 0.57142857 | MCU | HD | 0.85707195 | 2 |
| 4 | 3 | 0.57142857 | MCU | HD | 0.85707195 | 2 |
| 7 | 5 | 0.58333333 | MCU | HD | 0.8691222 | 2 |
| 3 | 2 | 0.6 | MCU | HD | 0.88607712 | 2 |
| 3 | 2 | 0.6 | MCU | HD | 0.88607712 | 2 |
| 3 | 2 | 0.6 | MCU | HD | 0.88607712 | 2 |
| 3 | 2 | 0.6 | MCU | HD | 0.88607712 | 2 |
| 5 | 3 | 0.625 | MCU | HD | 0.91173829 | 2 |
| 5 | 3 | 0.625 | MCU | HD | 0.91173829 | 2 |
| 2 | 1 | 0.66666667 | MCU | HD | 0.95531662 | 2 |
| 4 | 2 | 0.66666667 | MCU | HD | 0.95531662 | 2 |
| 4 | 2 | 0.66666667 | MCU | HD | 0.95531662 | 2 |
| 6 | 3 | 0.66666667 | MCU | HD | 0.95531662 | 2 |
| 8 | 4 | 0.66666667 | MCU | HD | 0.95531662 | 2 |
| 9 | 4 | 0.69230769 | MCU | HD | 0.98279372 | 2 |
| 7 | 3 | 0.7 | MCU | HD | 0.99115659 | 2 |
| 7 | 3 | 0.7 | MCU | HD | 0.99115659 | 2 |
| 5 | 2 | 0.71428571 | MCU | HD | 1.00685369 | 2 |
| 5 | 2 | 0.71428571 | MCU | HD | 1.00685369 | 2 |
| 5 | 2 | 0.71428571 | MCU | HD | 1.00685369 | 2 |
| 5 | 2 | 0.71428571 | MCU | HD | 1.00685369 | 2 |
| 5 | 2 | 0.71428571 | MCU | HD | 1.00685369 | 2 |
| 5 | 2 | 0.71428571 | MCU | HD | 1.00685369 | 2 |
| 8 | 3 | 0.72727273 | MCU | HD | 1.02132908 | 2 |
| 3 | 1 | 0.75 | MCU | HD | 1.04719755 | 2 |
| 3 | 1 | 0.75 | MCU | HD | 1.04719755 | 2 |
| 3 | 1 | 0.75 | MCU | HD | 1.04719755 | 2 |
| 3 | 1 | 0.75 | MCU | HD | 1.04719755 | 2 |
| 6 | 2 | 0.75 | MCU | HD | 1.04719755 | 2 |
| 6 | 2 | 0.75 | MCU | HD | 1.04719755 | 2 |
| 6 | 2 | 0.75 | MCU | HD | 1.04719755 | 2 |
| 6 | 2 | 0.75 | MCU | HD | 1.04719755 | 2 |
| 6 | 2 | 0.75 | MCU | HD | 1.04719755 | 2 |
| 10 | 3 | 0.76923077 | MCU | HD | 1.06970331 | 2 |
| 7 | 2 | 0.77777778 | MCU | HD | 1.07991365 | 2 |
| 7 | 2 | 0.77777778 | MCU | HD | 1.07991365 | 2 |
| 7 | 2 | 0.77777778 | MCU | HD | 1.07991365 | 2 |
| 7 | 2 | 0.77777778 | MCU | HD | 1.07991365 | 2 |
| 4 | 1 | 0.8 | MCU | HD | 1.10714872 | 2 |
| 4 | 1 | 0.8 | MCU | HD | 1.10714872 | 2 |
| 4 | 1 | 0.8 | MCU | HD | 1.10714872 | 2 |
| 4 | 1 | 0.8 | MCU | HD | 1.10714872 | 2 |
| 4 | 1 | 0.8 | MCU | HD | 1.10714872 | 2 |
| 4 | 1 | 0.8 | MCU | HD | 1.10714872 | 2 |
| 9 | 2 | 0.81818182 | MCU | HD | 1.13028566 | 2 |
| 9 | 2 | 0.81818182 | MCU | HD | 1.13028566 | 2 |
| 5 | 1 | 0.83333333 | MCU | HD | 1.15026199 | 2 |
| 10 | 2 | 0.83333333 | MCU | HD | 1.15026199 | 2 |
| 6 | 1 | 0.85714286 | MCU | HD | 1.18319964 | 2 |
| 6 | 1 | 0.85714286 | MCU | HD | 1.18319964 | 2 |
| 7 | 1 | 0.875 | MCU | HD | 1.2094292 | 2 |
| 7 | 1 | 0.875 | MCU | HD | 1.2094292 | 2 |
| 7 | 1 | 0.875 | MCU | HD | 1.2094292 | 2 |
| 7 | 1 | 0.875 | MCU | HD | 1.2094292 | 2 |
| 8 | 1 | 0.88888889 | MCU | HD | 1.23095942 | 2 |
| 10 | 1 | 0.90909091 | MCU | HD | 1.26451896 | 2 |
| 11 | 1 | 0.91666667 | MCU | HD | 1.27795356 | 2 |
| 11 | 1 | 0.91666667 | MCU | HD | 1.27795356 | 2 |
| 1 | 0 | 1 | MCU | HD | 1.57079633 | 2 |
| 2 | 0 | 1 | MCU | HD | 1.57079633 | 2 |
| 4 | 0 | 1 | MCU | HD | 1.57079633 | 2 |
| 5 | 0 | 1 | MCU | HD | 1.57079633 | 2 |
| 5 | 0 | 1 | MCU | HD | 1.57079633 | 2 |
| 5 | 0 | 1 | MCU | HD | 1.57079633 | 2 |
| 5 | 0 | 1 | MCU | HD | 1.57079633 | 2 |
| 6 | 0 | 1 | MCU | HD | 1.57079633 | 2 |
| 6 | 0 | 1 | MCU | HD | 1.57079633 | 2 |
| 7 | 0 | 1 | MCU | HD | 1.57079633 | 2 |
| 7 | 0 | 1 | MCU | HD | 1.57079633 | 2 |
| 7 | 0 | 1 | MCU | HD | 1.57079633 | 2 |
| 7 | 0 | 1 | MCU | HD | 1.57079633 | 2 |
| 8 | 0 | 1 | MCU | HD | 1.57079633 | 2 |
| 8 | 0 | 1 | MCU | HD | 1.57079633 | 2 |
| 9 | 0 | 1 | MCU | HD | 1.57079633 | 2 |
| 9 | 0 | 1 | MCU | HD | 1.57079633 | 2 |
| 9 | 0 | 1 | MCU | HD | 1.57079633 | 2 |
| 10 | 0 | 1 | MCU | HD | 1.57079633 | 2 |
| 10 | 0 | 1 | MCU | HD | 1.57079633 | 2 |
| 11 | 0 | 1 | MCU | HD | 1.57079633 | 2 |
| 11 | 0 | 1 | MCU | HD | 1.57079633 | 2 |
| 11 | 0 | 1 | MCU | HD | 1.57079633 | 2 |
| 13 | 0 | 1 | MCU | HD | 1.57079633 | 2 |
| 2 | 15 | 0.117647059 | MCU | HD | 0.350105778 | 3 |
| 2 | 6 | 0.25 | MCU | HD | 0.523598776 | 3 |
| 7 | 17 | 0.291666667 | MCU | HD | 0.570510448 | 3 |
| 3 | 6 | 0.333333333 | MCU | HD | 0.615479709 | 3 |
| 7 | 12 | 0.368421053 | MCU | HD | 0.652251155 | 3 |
| 5 | 8 | 0.384615385 | MCU | HD | 0.668964074 | 3 |
| 2 | 3 | 0.4 | MCU | HD | 0.684719203 | 3 |
| 4 | 6 | 0.4 | MCU | HD | 0.684719203 | 3 |
| 4 | 5 | 0.444444444 | MCU | HD | 0.729727656 | 3 |
| 8 | 8 | 0.5 | MCU | HD | 0.785398163 | 3 |
| 3 | 3 | 0.5 | MCU | HD | 0.785398163 | 3 |
| 5 | 5 | 0.5 | MCU | HD | 0.785398163 | 3 |
| 8 | 7 | 0.533333333 | MCU | HD | 0.818756238 | 3 |
| 6 | 5 | 0.545454545 | MCU | HD | 0.830915552 | 3 |
| 10 | 8 | 0.555555556 | MCU | HD | 0.841068671 | 3 |
| 4 | 3 | 0.571428571 | MCU | HD | 0.857071948 | 3 |
| 6 | 4 | 0.6 | MCU | HD | 0.886077124 | 3 |
| 8 | 5 | 0.615384615 | MCU | HD | 0.901832253 | 3 |
| 5 | 3 | 0.625 | MCU | HD | 0.911738291 | 3 |
| 5 | 3 | 0.625 | MCU | HD | 0.911738291 | 3 |
| 5 | 3 | 0.625 | MCU | HD | 0.911738291 | 3 |
| 7 | 4 | 0.636363636 | MCU | HD | 0.923511479 | 3 |
| 7 | 4 | 0.636363636 | MCU | HD | 0.923511479 | 3 |
| 7 | 4 | 0.636363636 | MCU | HD | 0.923511479 | 3 |
| 9 | 5 | 0.642857143 | MCU | HD | 0.930274014 | 3 |
| 11 | 6 | 0.647058824 | MCU | HD | 0.934664264 | 3 |
| 13 | 6 | 0.684210526 | MCU | HD | 0.97405318 | 3 |
| 13 | 6 | 0.684210526 | MCU | HD | 0.97405318 | 3 |
| 9 | 4 | 0.692307692 | MCU | HD | 0.982793723 | 3 |
| 9 | 4 | 0.692307692 | MCU | HD | 0.982793723 | 3 |
| 7 | 3 | 0.7 | MCU | HD | 0.991156586 | 3 |
| 5 | 2 | 0.714285714 | MCU | HD | 1.006853685 | 3 |
| 8 | 3 | 0.727272727 | MCU | HD | 1.021329082 | 3 |
| 3 | 1 | 0.75 | MCU | HD | 1.047197551 | 3 |
| 6 | 2 | 0.75 | MCU | HD | 1.047197551 | 3 |
| 3 | 1 | 0.75 | MCU | HD | 1.047197551 | 3 |
| 7 | 2 | 0.777777778 | MCU | HD | 1.079913649 | 3 |
| 8 | 2 | 0.8 | MCU | HD | 1.107148718 | 3 |
| 8 | 2 | 0.8 | MCU | HD | 1.107148718 | 3 |
| 4 | 1 | 0.8 | MCU | HD | 1.107148718 | 3 |
| 8 | 2 | 0.8 | MCU | HD | 1.107148718 | 3 |
| 9 | 2 | 0.818181818 | MCU | HD | 1.130285664 | 3 |
| 14 | 3 | 0.823529412 | MCU | HD | 1.137258417 | 3 |
| 15 | 3 | 0.833333333 | MCU | HD | 1.150261992 | 3 |
| 11 | 2 | 0.846153846 | MCU | HD | 1.167739252 | 3 |
| 11 | 2 | 0.846153846 | MCU | HD | 1.167739252 | 3 |
| 11 | 2 | 0.846153846 | MCU | HD | 1.167739252 | 3 |
| 12 | 2 | 0.857142857 | MCU | HD | 1.18319964 | 3 |
| 12 | 2 | 0.857142857 | MCU | HD | 1.18319964 | 3 |
| 24 | 4 | 0.857142857 | MCU | HD | 1.18319964 | 3 |
| 13 | 2 | 0.866666667 | MCU | HD | 1.197004152 | 3 |
| 14 | 2 | 0.875 | MCU | HD | 1.209429203 | 3 |
| 7 | 1 | 0.875 | MCU | HD | 1.209429203 | 3 |
| 7 | 1 | 0.875 | MCU | HD | 1.209429203 | 3 |
| 8 | 1 | 0.888888889 | MCU | HD | 1.230959417 | 3 |
| 8 | 1 | 0.888888889 | MCU | HD | 1.230959417 | 3 |
| 8 | 1 | 0.888888889 | MCU | HD | 1.230959417 | 3 |
| 8 | 1 | 0.888888889 | MCU | HD | 1.230959417 | 3 |
| 8 | 1 | 0.888888889 | MCU | HD | 1.230959417 | 3 |
| 9 | 1 | 0.9 | MCU | HD | 1.249045772 | 3 |
| 9 | 1 | 0.9 | MCU | HD | 1.249045772 | 3 |
| 9 | 1 | 0.9 | MCU | HD | 1.249045772 | 3 |
| 9 | 1 | 0.9 | MCU | HD | 1.249045772 | 3 |
| 10 | 1 | 0.909090909 | MCU | HD | 1.264518958 | 3 |
| 10 | 1 | 0.909090909 | MCU | HD | 1.264518958 | 3 |
| 10 | 1 | 0.909090909 | MCU | HD | 1.264518958 | 3 |
| 10 | 1 | 0.909090909 | MCU | HD | 1.264518958 | 3 |
| 11 | 1 | 0.916666667 | MCU | HD | 1.277953555 | 3 |
| 11 | 1 | 0.916666667 | MCU | HD | 1.277953555 | 3 |
| 12 | 1 | 0.923076923 | MCU | HD | 1.289761425 | 3 |
| 12 | 1 | 0.923076923 | MCU | HD | 1.289761425 | 3 |
| 12 | 1 | 0.923076923 | MCU | HD | 1.289761425 | 3 |
| 13 | 1 | 0.928571429 | MCU | HD | 1.300246564 | 3 |
| 7 | 0 | 1 | MCU | HD | 1.570796327 | 3 |
| 12 | 0 | 1 | MCU | HD | 1.570796327 | 3 |
| 12 | 0 | 1 | MCU | HD | 1.570796327 | 3 |
| 13 | 0 | 1 | MCU | HD | 1.570796327 | 3 |
| 15 | 0 | 1 | MCU | HD | 1.570796327 | 3 |
| 10 | 0 | 1 | MCU | HD | 1.570796327 | 3 |
| 3 | 0 | 1 | MCU | HD | 1.570796327 | 3 |
| 11 | 0 | 1 | MCU | HD | 1.570796327 | 3 |
| 13 | 0 | 1 | MCU | HD | 1.570796327 | 3 |
| 10 | 0 | 1 | MCU | HD | 1.570796327 | 3 |
| 9 | 0 | 1 | MCU | HD | 1.570796327 | 3 |
| 9 | 0 | 1 | MCU | HD | 1.570796327 | 3 |
| 10 | 0 | 1 | MCU | HD | 1.570796327 | 3 |
| 10 | 0 | 1 | MCU | HD | 1.570796327 | 3 |
| 8 | 0 | 1 | MCU | HD | 1.570796327 | 3 |
| 6 | 0 | 1 | MCU | HD | 1.570796327 | 3 |
| 14 | 0 | 1 | MCU | HD | 1.570796327 | 3 |
| 12 | 0 | 1 | MCU | HD | 1.570796327 | 3 |
| 12 | 0 | 1 | MCU | HD | 1.570796327 | 3 |
| 4 | 0 | 1 | MCU | HD | 1.570796327 | 3 |
| 13 | 0 | 1 | MCU | HD | 1.570796327 | 3 |
| 13 | 0 | 1 | MCU | HD | 1.570796327 | 3 |
| 13 | 0 | 1 | MCU | HD | 1.570796327 | 3 |
| 8 | 0 | 1 | MCU | HD | 1.570796327 | 3 |
| 7 | 0 | 1 | MCU | HD | 1.570796327 | 3 |
| 7 | 0 | 1 | MCU | HD | 1.570796327 | 3 |
| 11 | 0 | 1 | MCU | HD | 1.570796327 | 3 |
| 13 | 0 | 1 | MCU | HD | 1.570796327 | 3 |
| 11 | 0 | 1 | MCU | HD | 1.570796327 | 3 |
| 9 | 0 | 1 | MCU | HD | 1.570796327 | 3 |
| 12 | 0 | 1 | MCU | HD | 1.570796327 | 3 |
| 10 | 0 | 1 | MCU | HD | 1.570796327 | 3 |
| 8 | 0 | 1 | MCU | HD | 1.570796327 | 4 |
| 5 | 0 | 1 | MCU | HD | 1.570796327 | 4 |
| 12 | 0 | 1 | MCU | HD | 1.570796327 | 4 |
| 8 | 0 | 1 | MCU | HD | 1.570796327 | 4 |
| 10 | 0 | 1 | MCU | HD | 1.570796327 | 4 |
| 9 | 0 | 1 | MCU | HD | 1.570796327 | 4 |
| 6 | 0 | 1 | MCU | HD | 1.570796327 | 4 |
| 15 | 0 | 1 | MCU | HD | 1.570796327 | 4 |
| 9 | 0 | 1 | MCU | HD | 1.570796327 | 4 |
| 6 | 0 | 1 | MCU | HD | 1.570796327 | 4 |
| 9 | 0 | 1 | MCU | HD | 1.570796327 | 4 |
| 1 | 0 | 1 | MCU | HD | 1.570796327 | 4 |
| 4 | 0 | 1 | MCU | HD | 1.570796327 | 4 |
| 5 | 0 | 1 | MCU | HD | 1.570796327 | 4 |
| 7 | 0 | 1 | MCU | HD | 1.570796327 | 4 |
| 6 | 0 | 1 | MCU | HD | 1.570796327 | 4 |
| 8 | 0 | 1 | MCU | HD | 1.570796327 | 4 |
| 10 | 0 | 1 | MCU | HD | 1.570796327 | 4 |
| 5 | 0 | 1 | MCU | HD | 1.570796327 | 4 |
| 6 | 0 | 1 | MCU | HD | 1.570796327 | 4 |
| 9 | 0 | 1 | MCU | HD | 1.570796327 | 4 |
| 6 | 0 | 1 | MCU | HD | 1.570796327 | 4 |
| 6 | 0 | 1 | MCU | HD | 1.570796327 | 4 |
| 4 | 0 | 1 | MCU | HD | 1.570796327 | 4 |
| 9 | 0 | 1 | MCU | HD | 1.570796327 | 4 |
| 9 | 0 | 1 | MCU | HD | 1.570796327 | 4 |
| 1 | 0 | 1 | MCU | HD | 1.570796327 | 4 |
| 5 | 0 | 1 | MCU | HD | 1.570796327 | 4 |
| 8 | 0 | 1 | MCU | HD | 1.570796327 | 4 |
| 8 | 0 | 1 | MCU | HD | 1.570796327 | 4 |
| 8 | 0 | 1 | MCU | HD | 1.570796327 | 4 |
| 6 | 0 | 1 | MCU | HD | 1.570796327 | 4 |
| 13 | 0 | 1 | MCU | HD | 1.570796327 | 4 |
| 14 | 1 | 0.933333333 | MCU | HD | 1.309638916 | 4 |
| 11 | 1 | 0.916666667 | MCU | HD | 1.277953555 | 4 |
| 10 | 1 | 0.909090909 | MCU | HD | 1.264518958 | 4 |
| 9 | 1 | 0.9 | MCU | HD | 1.249045772 | 4 |
| 9 | 1 | 0.9 | MCU | HD | 1.249045772 | 4 |
| 8 | 1 | 0.888888889 | MCU | HD | 1.230959417 | 4 |
| 8 | 1 | 0.888888889 | MCU | HD | 1.230959417 | 4 |
| 7 | 1 | 0.875 | MCU | HD | 1.209429203 | 4 |
| 13 | 2 | 0.866666667 | MCU | HD | 1.197004152 | 4 |
| 6 | 1 | 0.857142857 | MCU | HD | 1.18319964 | 4 |
| 12 | 2 | 0.857142857 | MCU | HD | 1.18319964 | 4 |
| 6 | 1 | 0.857142857 | MCU | HD | 1.18319964 | 4 |
| 6 | 1 | 0.857142857 | MCU | HD | 1.18319964 | 4 |
| 11 | 2 | 0.846153846 | MCU | HD | 1.167739252 | 4 |
| 9 | 2 | 0.818181818 | MCU | HD | 1.130285664 | 4 |
| 9 | 2 | 0.818181818 | MCU | HD | 1.130285664 | 4 |
| 9 | 2 | 0.818181818 | MCU | HD | 1.130285664 | 4 |
| 9 | 2 | 0.818181818 | MCU | HD | 1.130285664 | 4 |
| 11 | 3 | 0.785714286 | MCU | HD | 1.089520953 | 4 |
| 7 | 2 | 0.777777778 | MCU | HD | 1.079913649 | 4 |
| 7 | 2 | 0.777777778 | MCU | HD | 1.079913649 | 4 |
| 7 | 2 | 0.777777778 | MCU | HD | 1.079913649 | 4 |
| 3 | 1 | 0.75 | MCU | HD | 1.047197551 | 4 |
| 3 | 1 | 0.75 | MCU | HD | 1.047197551 | 4 |
| 3 | 1 | 0.75 | MCU | HD | 1.047197551 | 4 |
| 6 | 2 | 0.75 | MCU | HD | 1.047197551 | 4 |
| 6 | 2 | 0.75 | MCU | HD | 1.047197551 | 4 |
| 5 | 2 | 0.714285714 | MCU | HD | 1.006853685 | 4 |
| 5 | 2 | 0.714285714 | MCU | HD | 1.006853685 | 4 |
| 5 | 2 | 0.714285714 | MCU | HD | 1.006853685 | 4 |
| 7 | 3 | 0.7 | MCU | HD | 0.991156586 | 4 |
| 7 | 3 | 0.7 | MCU | HD | 0.991156586 | 4 |
| 8 | 4 | 0.666666667 | MCU | HD | 0.955316618 | 4 |
| 6 | 3 | 0.666666667 | MCU | HD | 0.955316618 | 4 |
| 4 | 2 | 0.666666667 | MCU | HD | 0.955316618 | 4 |
| 6 | 3 | 0.666666667 | MCU | HD | 0.955316618 | 4 |
| 2 | 1 | 0.666666667 | MCU | HD | 0.955316618 | 4 |
| 6 | 3 | 0.666666667 | MCU | HD | 0.955316618 | 4 |
| 7 | 4 | 0.636363636 | MCU | HD | 0.923511479 | 4 |
| 7 | 4 | 0.636363636 | MCU | HD | 0.923511479 | 4 |
| 5 | 3 | 0.625 | MCU | HD | 0.911738291 | 4 |
| 5 | 3 | 0.625 | MCU | HD | 0.911738291 | 4 |
| 8 | 5 | 0.615384615 | MCU | HD | 0.901832253 | 4 |
| 9 | 6 | 0.6 | MCU | HD | 0.886077124 | 4 |
| 7 | 5 | 0.583333333 | MCU | HD | 0.869122203 | 4 |
| 4 | 3 | 0.571428571 | MCU | HD | 0.857071948 | 4 |
| 5 | 4 | 0.555555556 | MCU | HD | 0.841068671 | 4 |
| 6 | 5 | 0.545454545 | MCU | HD | 0.830915552 | 4 |
| 6 | 5 | 0.545454545 | MCU | HD | 0.830915552 | 4 |
| 13 | 12 | 0.52 | MCU | HD | 0.805403501 | 4 |
| 5 | 5 | 0.5 | MCU | HD | 0.785398163 | 4 |
| 5 | 6 | 0.454545455 | MCU | HD | 0.739880774 | 4 |
| 4 | 5 | 0.444444444 | MCU | HD | 0.729727656 | 4 |
| 3 | 4 | 0.428571429 | MCU | HD | 0.713724379 | 4 |
| 3 | 7 | 0.3 | MCU | HD | 0.57963974 | 4 |
| 2 | 6 | 0.25 | MCU | HD | 0.523598776 | 4 |
| 3 | 11 | 0.214285714 | MCU | HD | 0.481275374 | 4 |
| 1 | 4 | 0.2 | MCU | HD | 0.463647609 | 4 |
| 1 | 9 | 0.1 | MCU | HD | 0.321750554 | 4 |
| 1 | 10 | 0.090909091 | MCU | HD | 0.306277369 | 4 |
| 1 | 6 | 0.14285714 | MCU | LD | 0.38759669 | 2 |
| 4 | 5 | 0.44444444 | MCU | LD | 0.72972766 | 2 |
| 3 | 3 | 0.5 | MCU | LD | 0.78539816 | 2 |
| 5 | 5 | 0.5 | MCU | LD | 0.78539816 | 2 |
| 4 | 3 | 0.57142857 | MCU | LD | 0.85707195 | 2 |
| 4 | 3 | 0.57142857 | MCU | LD | 0.85707195 | 2 |
| 10 | 4 | 0.71428571 | MCU | LD | 1.00685369 | 2 |
| 4 | 1 | 0.8 | MCU | LD | 1.10714872 | 2 |
| 8 | 2 | 0.8 | MCU | LD | 1.10714872 | 2 |
| 5 | 1 | 0.83333333 | MCU | LD | 1.15026199 | 2 |
| 5 | 1 | 0.83333333 | MCU | LD | 1.15026199 | 2 |
| 11 | 2 | 0.84615385 | MCU | LD | 1.16773925 | 2 |
| 6 | 1 | 0.85714286 | MCU | LD | 1.18319964 | 2 |
| 7 | 1 | 0.875 | MCU | LD | 1.2094292 | 2 |
| 7 | 1 | 0.875 | MCU | LD | 1.2094292 | 2 |
| 7 | 1 | 0.875 | MCU | LD | 1.2094292 | 2 |
| 8 | 1 | 0.88888889 | MCU | LD | 1.23095942 | 2 |
| 9 | 1 | 0.9 | MCU | LD | 1.24904577 | 2 |
| 10 | 1 | 0.90909091 | MCU | LD | 1.26451896 | 2 |
| 13 | 1 | 0.92857143 | MCU | LD | 1.30024656 | 2 |
| 30 | 2 | 0.9375 | MCU | LD | 1.31811607 | 2 |
| 3 | 0 | 1 | MCU | LD | 1.57079633 | 2 |
| 4 | 0 | 1 | MCU | LD | 1.57079633 | 2 |
| 4 | 0 | 1 | MCU | LD | 1.57079633 | 2 |
| 5 | 0 | 1 | MCU | LD | 1.57079633 | 2 |
| 5 | 0 | 1 | MCU | LD | 1.57079633 | 2 |
| 5 | 0 | 1 | MCU | LD | 1.57079633 | 2 |
| 5 | 0 | 1 | MCU | LD | 1.57079633 | 2 |
| 5 | 0 | 1 | MCU | LD | 1.57079633 | 2 |
| 5 | 0 | 1 | MCU | LD | 1.57079633 | 2 |
| 6 | 0 | 1 | MCU | LD | 1.57079633 | 2 |
| 6 | 0 | 1 | MCU | LD | 1.57079633 | 2 |
| 6 | 0 | 1 | MCU | LD | 1.57079633 | 2 |
| 6 | 0 | 1 | MCU | LD | 1.57079633 | 2 |
| 7 | 0 | 1 | MCU | LD | 1.57079633 | 2 |
| 7 | 0 | 1 | MCU | LD | 1.57079633 | 2 |
| 7 | 0 | 1 | MCU | LD | 1.57079633 | 2 |
| 8 | 0 | 1 | MCU | LD | 1.57079633 | 2 |
| 8 | 0 | 1 | MCU | LD | 1.57079633 | 2 |
| 8 | 0 | 1 | MCU | LD | 1.57079633 | 2 |
| 8 | 0 | 1 | MCU | LD | 1.57079633 | 2 |
| 8 | 0 | 1 | MCU | LD | 1.57079633 | 2 |
| 8 | 0 | 1 | MCU | LD | 1.57079633 | 2 |
| 8 | 0 | 1 | MCU | LD | 1.57079633 | 2 |
| 9 | 0 | 1 | MCU | LD | 1.57079633 | 2 |
| 9 | 0 | 1 | MCU | LD | 1.57079633 | 2 |
| 10 | 0 | 1 | MCU | LD | 1.57079633 | 2 |
| 10 | 0 | 1 | MCU | LD | 1.57079633 | 2 |
| 10 | 0 | 1 | MCU | LD | 1.57079633 | 2 |
| 10 | 0 | 1 | MCU | LD | 1.57079633 | 2 |
| 11 | 0 | 1 | MCU | LD | 1.57079633 | 2 |
| 11 | 0 | 1 | MCU | LD | 1.57079633 | 2 |
| 15 | 0 | 1 | MCU | LD | 1.57079633 | 2 |
| 15 | 0 | 1 | MCU | LD | 1.57079633 | 2 |
| 17 | 0 | 1 | MCU | LD | 1.57079633 | 2 |
| 17 | 0 | 1 | MCU | LD | 1.57079633 | 2 |
| 3 | 9 | 0.25 | MCU | LD | 0.523598776 | 3 |
| 4 | 5 | 0.444444444 | MCU | LD | 0.729727656 | 3 |
| 8 | 7 | 0.533333333 | MCU | LD | 0.818756238 | 3 |
| 8 | 6 | 0.571428571 | MCU | LD | 0.857071948 | 3 |
| 8 | 5 | 0.615384615 | MCU | LD | 0.901832253 | 3 |
| 9 | 5 | 0.642857143 | MCU | LD | 0.930274014 | 3 |
| 8 | 4 | 0.666666667 | MCU | LD | 0.955316618 | 3 |
| 6 | 3 | 0.666666667 | MCU | LD | 0.955316618 | 3 |
| 9 | 3 | 0.75 | MCU | LD | 1.047197551 | 3 |
| 6 | 2 | 0.75 | MCU | LD | 1.047197551 | 3 |
| 10 | 3 | 0.769230769 | MCU | LD | 1.069703314 | 3 |
| 12 | 3 | 0.8 | MCU | LD | 1.107148718 | 3 |
| 14 | 3 | 0.823529412 | MCU | LD | 1.137258417 | 3 |
| 10 | 2 | 0.833333333 | MCU | LD | 1.150261992 | 3 |
| 5 | 1 | 0.833333333 | MCU | LD | 1.150261992 | 3 |
| 16 | 3 | 0.842105263 | MCU | LD | 1.162158472 | 3 |
| 12 | 2 | 0.857142857 | MCU | LD | 1.18319964 | 3 |
| 13 | 2 | 0.866666667 | MCU | LD | 1.197004152 | 3 |
| 13 | 2 | 0.866666667 | MCU | LD | 1.197004152 | 3 |
| 13 | 2 | 0.866666667 | MCU | LD | 1.197004152 | 3 |
| 7 | 1 | 0.875 | MCU | LD | 1.209429203 | 3 |
| 7 | 1 | 0.875 | MCU | LD | 1.209429203 | 3 |
| 7 | 1 | 0.875 | MCU | LD | 1.209429203 | 3 |
| 36 | 5 | 0.87804878 | MCU | LD | 1.214062938 | 3 |
| 16 | 2 | 0.888888889 | MCU | LD | 1.230959417 | 3 |
| 8 | 1 | 0.888888889 | MCU | LD | 1.230959417 | 3 |
| 8 | 1 | 0.888888889 | MCU | LD | 1.230959417 | 3 |
| 17 | 2 | 0.894736842 | MCU | LD | 1.240373679 | 3 |
| 9 | 1 | 0.9 | MCU | LD | 1.249045772 | 3 |
| 9 | 1 | 0.9 | MCU | LD | 1.249045772 | 3 |
| 10 | 1 | 0.909090909 | MCU | LD | 1.264518958 | 3 |
| 11 | 1 | 0.916666667 | MCU | LD | 1.277953555 | 3 |
| 11 | 1 | 0.916666667 | MCU | LD | 1.277953555 | 3 |
| 12 | 1 | 0.923076923 | MCU | LD | 1.289761425 | 3 |
| 13 | 1 | 0.928571429 | MCU | LD | 1.300246564 | 3 |
| 13 | 1 | 0.928571429 | MCU | LD | 1.300246564 | 3 |
| 13 | 1 | 0.928571429 | MCU | LD | 1.300246564 | 3 |
| 18 | 1 | 0.947368421 | MCU | LD | 1.339318963 | 3 |
| 11 | 0 | 1 | MCU | LD | 1.570796327 | 3 |
| 21 | 0 | 1 | MCU | LD | 1.570796327 | 3 |
| 10 | 0 | 1 | MCU | LD | 1.570796327 | 3 |
| 14 | 0 | 1 | MCU | LD | 1.570796327 | 3 |
| 8 | 0 | 1 | MCU | LD | 1.570796327 | 3 |
| 7 | 0 | 1 | MCU | LD | 1.570796327 | 3 |
| 15 | 0 | 1 | MCU | LD | 1.570796327 | 3 |
| 4 | 0 | 1 | MCU | LD | 1.570796327 | 3 |
| 8 | 0 | 1 | MCU | LD | 1.570796327 | 3 |
| 13 | 0 | 1 | MCU | LD | 1.570796327 | 3 |
| 10 | 0 | 1 | MCU | LD | 1.570796327 | 3 |
| 16 | 0 | 1 | MCU | LD | 1.570796327 | 3 |
| 9 | 0 | 1 | MCU | LD | 1.570796327 | 3 |
| 18 | 0 | 1 | MCU | LD | 1.570796327 | 3 |
| 16 | 0 | 1 | MCU | LD | 1.570796327 | 3 |
| 10 | 0 | 1 | MCU | LD | 1.570796327 | 3 |
| 17 | 0 | 1 | MCU | LD | 1.570796327 | 3 |
| 20 | 0 | 1 | MCU | LD | 1.570796327 | 3 |
| 16 | 0 | 1 | MCU | LD | 1.570796327 | 3 |
| 2 | 0 | 1 | MCU | LD | 1.570796327 | 3 |
| 10 | 0 | 1 | MCU | LD | 1.570796327 | 3 |
| 7 | 0 | 1 | MCU | LD | 1.570796327 | 4 |
| 10 | 0 | 1 | MCU | LD | 1.570796327 | 4 |
| 10 | 0 | 1 | MCU | LD | 1.570796327 | 4 |
| 3 | 0 | 1 | MCU | LD | 1.570796327 | 4 |
| 9 | 0 | 1 | MCU | LD | 1.570796327 | 4 |
| 4 | 0 | 1 | MCU | LD | 1.570796327 | 4 |
| 6 | 0 | 1 | MCU | LD | 1.570796327 | 4 |
| 7 | 0 | 1 | MCU | LD | 1.570796327 | 4 |
| 7 | 0 | 1 | MCU | LD | 1.570796327 | 4 |
| 5 | 0 | 1 | MCU | LD | 1.570796327 | 4 |
| 16 | 0 | 1 | MCU | LD | 1.570796327 | 4 |
| 4 | 0 | 1 | MCU | LD | 1.570796327 | 4 |
| 11 | 0 | 1 | MCU | LD | 1.570796327 | 4 |
| 11 | 0 | 1 | MCU | LD | 1.570796327 | 4 |
| 10 | 0 | 1 | MCU | LD | 1.570796327 | 4 |
| 6 | 0 | 1 | MCU | LD | 1.570796327 | 4 |
| 7 | 0 | 1 | MCU | LD | 1.570796327 | 4 |
| 2 | 0 | 1 | MCU | LD | 1.570796327 | 4 |
| 7 | 0 | 1 | MCU | LD | 1.570796327 | 4 |
| 5 | 0 | 1 | MCU | LD | 1.570796327 | 4 |
| 8 | 0 | 1 | MCU | LD | 1.570796327 | 4 |
| 7 | 0 | 1 | MCU | LD | 1.570796327 | 4 |
| 13 | 0 | 1 | MCU | LD | 1.570796327 | 4 |
| 4 | 0 | 1 | MCU | LD | 1.570796327 | 4 |
| 10 | 0 | 1 | MCU | LD | 1.570796327 | 4 |
| 10 | 0 | 1 | MCU | LD | 1.570796327 | 4 |
| 6 | 0 | 1 | MCU | LD | 1.570796327 | 4 |
| 12 | 0 | 1 | MCU | LD | 1.570796327 | 4 |
| 34 | 1 | 0.971428571 | MCU | LD | 1.400950039 | 4 |
| 8 | 1 | 0.888888889 | MCU | LD | 1.230959417 | 4 |
| 8 | 1 | 0.888888889 | MCU | LD | 1.230959417 | 4 |
| 7 | 1 | 0.875 | MCU | LD | 1.209429203 | 4 |
| 6 | 1 | 0.857142857 | MCU | LD | 1.18319964 | 4 |
| 11 | 2 | 0.846153846 | MCU | LD | 1.167739252 | 4 |
| 11 | 2 | 0.846153846 | MCU | LD | 1.167739252 | 4 |
| 5 | 1 | 0.833333333 | MCU | LD | 1.150261992 | 4 |
| 5 | 1 | 0.833333333 | MCU | LD | 1.150261992 | 4 |
| 9 | 2 | 0.818181818 | MCU | LD | 1.130285664 | 4 |
| 13 | 3 | 0.8125 | MCU | LD | 1.12296393 | 4 |
| 8 | 2 | 0.8 | MCU | LD | 1.107148718 | 4 |
| 8 | 2 | 0.8 | MCU | LD | 1.107148718 | 4 |
| 9 | 3 | 0.75 | MCU | LD | 1.047197551 | 4 |
| 3 | 1 | 0.75 | MCU | LD | 1.047197551 | 4 |
| 9 | 3 | 0.75 | MCU | LD | 1.047197551 | 4 |
| 9 | 3 | 0.75 | MCU | LD | 1.047197551 | 4 |
| 7 | 3 | 0.7 | MCU | LD | 0.991156586 | 4 |
| 6 | 3 | 0.666666667 | MCU | LD | 0.955316618 | 4 |
| 4 | 2 | 0.666666667 | MCU | LD | 0.955316618 | 4 |
| 7 | 4 | 0.636363636 | MCU | LD | 0.923511479 | 4 |
| 5 | 3 | 0.625 | MCU | LD | 0.911738291 | 4 |
| 8 | 5 | 0.615384615 | MCU | LD | 0.901832253 | 4 |
| 3 | 2 | 0.6 | MCU | LD | 0.886077124 | 4 |
| 5 | 4 | 0.555555556 | MCU | LD | 0.841068671 | 4 |
| 3 | 3 | 0.5 | MCU | LD | 0.785398163 | 4 |
| 3 | 3 | 0.5 | MCU | LD | 0.785398163 | 4 |
| 6 | 7 | 0.461538462 | MCU | LD | 0.746898593 | 4 |
| 6 | 8 | 0.428571429 | MCU | LD | 0.713724379 | 4 |
| 8 | 17 | 0.32 | MCU | LD | 0.601264217 | 4 |
| 1 | 5 | 0.166666667 | MCU | LD | 0.420534335 | 4 |
| 10 | 0 | 1 | MCU | LD | 1.570796327 | 1 |
| 8 | 0 | 1 | MCU | LD | 1.570796327 | 1 |
| 9 | 3 | 0.75 | MCU | LD | 1.047197551 | 1 |
| 2 | 2 | 0.5 | MCU | LD | 0.785398163 | 1 |
| 2 | 4 | 0.333333333 | MCU | LD | 0.615479709 | 1 |
| 7 | 0 | 1 | MCU | LD | 1.570796327 | 1 |
| 6 | 1 | 0.857142857 | MCU | LD | 1.18319964 | 1 |
| 12 | 0 | 1 | MCU | LD | 1.570796327 | 1 |
| 7 | 0 | 1 | MCU | LD | 1.570796327 | 1 |
| 2 | 0 | 1 | MCU | LD | 1.570796327 | 1 |
| 7 | 0 | 1 | MCU | LD | 1.570796327 | 1 |
| 2 | 1 | 0.666666667 | MCU | LD | 0.955316618 | 1 |
| 9 | 2 | 0.818181818 | MCU | LD | 1.130285664 | 1 |
| 3 | 1 | 0.75 | MCU | LD | 1.047197551 | 1 |
| 7 | 0 | 1 | MCU | LD | 1.570796327 | 1 |
| 5 | 2 | 0.714285714 | MCU | LD | 1.006853685 | 1 |
| 6 | 1 | 0.857142857 | MCU | LD | 1.18319964 | 1 |
| 8 | 0 | 1 | MCU | LD | 1.570796327 | 1 |
| 5 | 1 | 0.833333333 | MCU | LD | 1.150261992 | 1 |
| 5 | 0 | 1 | MCU | LD | 1.570796327 | 1 |
| 6 | 1 | 0.857142857 | MCU | LD | 1.18319964 | 1 |
| 7 | 3 | 0.7 | MCU | LD | 0.991156586 | 1 |
| 6 | 1 | 0.857142857 | MCU | LD | 1.18319964 | 1 |
| 5 | 2 | 0.714285714 | MCU | LD | 1.006853685 | 1 |
| 6 | 1 | 0.857142857 | MCU | LD | 1.18319964 | 1 |
| 3 | 1 | 0.75 | MCU | LD | 1.047197551 | 1 |
| 9 | 0 | 1 | MCU | LD | 1.570796327 | 1 |
| 1 | 0 | 1 | MCU | LD | 1.570796327 | 1 |
| 4 | 4 | 0.5 | MCU | LD | 0.785398163 | 1 |
| 5 | 0 | 1 | MCU | LD | 1.570796327 | 1 |
| 3 | 1 | 0.75 | MCU | LD | 1.047197551 | 1 |
| 5 | 0 | 1 | MCU | LD | 1.570796327 | 1 |
| 2 | 1 | 0.666666667 | MCU | LD | 0.955316618 | 1 |
| 9 | 2 | 0.818181818 | MCU | LD | 1.130285664 | 1 |
| 7 | 3 | 0.7 | MCU | LD | 0.991156586 | 1 |
| 2 | 3 | 0.4 | MCU | LD | 0.684719203 | 1 |
| 4 | 0 | 1 | MCU | LD | 1.570796327 | 1 |
| 5 | 0 | 1 | MCU | LD | 1.570796327 | 1 |
| 3 | 1 | 0.75 | MCU | LD | 1.047197551 | 1 |
| 11 | 0 | 1 | MCU | LD | 1.570796327 | 1 |
| 6 | 2 | 0.75 | MCU | LD | 1.047197551 | 1 |
| 7 | 3 | 0.7 | MCU | LD | 0.991156586 | 1 |
| 8 | 1 | 0.888888889 | MCU | LD | 1.230959417 | 1 |
| 1 | 3 | 0.25 | MCU | LD | 0.523598776 | 1 |
| 7 | 0 | 1 | MCU | LD | 1.570796327 | 1 |
| 3 | 1 | 0.75 | MCU | LD | 1.047197551 | 1 |
| 7 | 3 | 0.7 | MCU | LD | 0.991156586 | 1 |
| 6 | 3 | 0.666666667 | MCU | LD | 0.955316618 | 1 |
| 6 | 2 | 0.75 | MCU | LD | 1.047197551 | 1 |
| 3 | 1 | 0.75 | MCU | LD | 1.047197551 | 1 |
| 6 | 1 | 0.857142857 | MCU | LD | 1.18319964 | 1 |
| 8 | 0 | 1 | MCU | LD | 1.570796327 | 1 |
| 6 | 2 | 0.75 | MCU | LD | 1.047197551 | 1 |
| 2 | 3 | 0.4 | MCU | LD | 0.684719203 | 1 |
| 7 | 1 | 0.875 | MCU | LD | 1.209429203 | 1 |
| 4 | 4 | 0.5 | MCU | LD | 0.785398163 | 1 |
| 9 | 16 | 0.36 | MB | HD | 0.643501109 | 1 |
| 2 | 0 | 1 | MB | HD | 1.570796327 | 1 |
| 1 | 4 | 0.2 | MB | HD | 0.463647609 | 1 |
| 6 | 1 | 0.857142857 | MB | HD | 1.18319964 | 1 |
| 1 | 5 | 0.166666667 | MB | HD | 0.420534335 | 1 |
| 5 | 1 | 0.833333333 | MB | HD | 1.150261992 | 1 |
| 7 | 2 | 0.777777778 | MB | HD | 1.079913649 | 1 |
| 5 | 0 | 1 | MB | HD | 1.570796327 | 1 |
| 7 | 0 | 1 | MB | HD | 1.570796327 | 1 |
| 6 | 0 | 1 | MB | HD | 1.570796327 | 1 |
| 4 | 1 | 0.8 | MB | HD | 1.107148718 | 1 |
| 4 | 0 | 1 | MB | HD | 1.570796327 | 1 |
| 9 | 2 | 0.818181818 | MB | HD | 1.130285664 | 1 |
| 4 | 1 | 0.8 | MB | HD | 1.107148718 | 1 |
| 11 | 2 | 0.846153846 | MB | HD | 1.167739252 | 1 |
| 3 | 1 | 0.75 | MB | HD | 1.047197551 | 1 |
| 2 | 3 | 0.4 | MB | HD | 0.684719203 | 1 |
| 1 | 5 | 0.166666667 | MB | HD | 0.420534335 | 1 |
| 3 | 2 | 0.6 | MB | HD | 0.886077124 | 1 |
| 1 | 0 | 1 | MB | HD | 1.570796327 | 1 |
| 3 | 8 | 0.272727273 | MB | HD | 0.549467245 | 1 |
| 1 | 11 | 0.083333333 | MB | HD | 0.292842772 | 1 |
| 3 | 1 | 0.75 | MB | HD | 1.047197551 | 1 |
| 4 | 1 | 0.8 | MB | HD | 1.107148718 | 1 |
| 7 | 2 | 0.777777778 | MB | HD | 1.079913649 | 1 |
| 5 | 0 | 1 | MB | HD | 1.570796327 | 1 |
| 6 | 2 | 0.75 | MB | HD | 1.047197551 | 1 |
| 8 | 0 | 1 | MB | HD | 1.570796327 | 1 |
| 2 | 4 | 0.333333333 | MB | HD | 0.615479709 | 1 |
| 1 | 4 | 0.2 | MB | HD | 0.463647609 | 1 |
| 3 | 7 | 0.3 | MB | HD | 0.57963974 | 1 |
| 9 | 2 | 0.818181818 | MB | HD | 1.130285664 | 1 |
| 2 | 2 | 0.5 | MB | HD | 0.785398163 | 1 |
| 5 | 1 | 0.833333333 | MB | HD | 1.150261992 | 1 |
| 6 | 0 | 1 | MB | HD | 1.570796327 | 1 |
| 5 | 0 | 1 | MB | HD | 1.570796327 | 1 |
| 1 | 1 | 0.5 | MB | HD | 0.785398163 | 1 |
| 4 | 3 | 0.571428571 | MB | HD | 0.857071948 | 1 |
| 3 | 2 | 0.6 | MB | HD | 0.886077124 | 1 |
| 4 | 0 | 1 | MB | HD | 1.570796327 | 1 |
| 1 | 6 | 0.142857143 | MB | HD | 0.387596687 | 1 |
| 1 | 7 | 0.125 | MB | HD | 0.361367124 | 1 |
| 5 | 3 | 0.625 | MB | HD | 0.911738291 | 1 |
| 8 | 0 | 1 | MB | HD | 1.570796327 | 1 |
| 2 | 2 | 0.5 | MB | HD | 0.785398163 | 1 |
| 1 | 4 | 0.2 | MB | HD | 0.463647609 | 1 |
| 7 | 1 | 0.875 | MB | HD | 1.209429203 | 1 |
| 9 | 1 | 0.9 | MB | HD | 1.249045772 | 1 |
| 2 | 4 | 0.333333333 | MB | HD | 0.615479709 | 1 |
| 1 | 2 | 0.333333333 | MB | HD | 0.615479709 | 1 |
| 3 | 2 | 0.6 | MB | HD | 0.886077124 | 1 |
| 3 | 0 | 1 | MB | HD | 1.570796327 | 1 |
| 1 | 1 | 0.5 | MB | HD | 0.785398163 | 1 |
| 4 | 2 | 0.666666667 | MB | HD | 0.955316618 | 1 |
| 3 | 4 | 0.428571429 | MB | HD | 0.713724379 | 1 |
| 3 | 6 | 0.333333333 | MB | HD | 0.615479709 | 1 |
| 3 | 6 | 0.333333333 | MB | HD | 0.615479709 | 1 |
| 2 | 2 | 0.5 | MB | HD | 0.785398163 | 1 |
| 4 | 0 | 1 | MB | HD | 1.570796327 | 1 |
| 4 | 2 | 0.666666667 | MB | HD | 0.955316618 | 1 |
| 7 | 0 | 1 | MB | HD | 1.570796327 | 1 |
| 2 | 5 | 0.285714286 | MB | HD | 0.563942641 | 1 |
| 4 | 1 | 0.8 | MB | HD | 1.107148718 | 1 |
| 1 | 0 | 1 | MB | HD | 1.570796327 | 1 |
| 1 | 3 | 0.25 | MB | HD | 0.523598776 | 1 |
| 3 | 2 | 0.6 | MB | HD | 0.886077124 | 1 |
| 5 | 2 | 0.714285714 | MB | HD | 1.006853685 | 1 |
| 13 | 0 | 1 | MB | HD | 1.570796327 | 1 |
| 6 | 2 | 0.75 | MB | HD | 1.047197551 | 1 |
| 1 | 4 | 0.2 | MB | HD | 0.463647609 | 1 |
| 3 | 3 | 0.5 | MB | HD | 0.785398163 | 1 |
| 3 | 2 | 0.6 | MB | HD | 0.886077124 | 1 |
| 5 | 0 | 1 | MB | HD | 1.570796327 | 1 |
| 6 | 0 | 1 | MB | HD | 1.570796327 | 1 |
| 3 | 1 | 0.75 | MB | HD | 1.047197551 | 1 |
| 10 | 7 | 0.588235294 | MB | HD | 0.874097966 | 1 |
| 2 | 5 | 0.285714286 | MB | HD | 0.563942641 | 1 |
| 1 | 2 | 0.333333333 | MB | HD | 0.615479709 | 1 |
| 3 | 0 | 1 | MB | HD | 1.570796327 | 1 |
| 3 | 1 | 0.75 | MB | HD | 1.047197551 | 1 |
| 5 | 4 | 0.555555556 | MB | LD | 0.841068671 | 1 |
| 4 | 0 | 1 | MB | LD | 1.570796327 | 1 |
| 2 | 2 | 0.5 | MB | LD | 0.785398163 | 1 |
| 2 | 1 | 0.666666667 | MB | LD | 0.955316618 | 1 |
| 9 | 3 | 0.75 | MB | LD | 1.047197551 | 1 |
| 6 | 4 | 0.6 | MB | LD | 0.886077124 | 1 |
| 5 | 2 | 0.714285714 | MB | LD | 1.006853685 | 1 |
| 6 | 0 | 1 | MB | LD | 1.570796327 | 1 |
| 7 | 6 | 0.538461538 | MB | LD | 0.823897734 | 1 |
| 6 | 0 | 1 | MB | LD | 1.570796327 | 1 |
| 8 | 0 | 1 | MB | LD | 1.570796327 | 1 |
| 4 | 3 | 0.571428571 | MB | LD | 0.857071948 | 1 |
| 8 | 0 | 1 | MB | LD | 1.570796327 | 1 |
| 6 | 0 | 1 | MB | LD | 1.570796327 | 1 |
| 6 | 1 | 0.857142857 | MB | LD | 1.18319964 | 1 |
| 3 | 1 | 0.75 | MB | LD | 1.047197551 | 1 |
| 3 | 1 | 0.75 | MB | LD | 1.047197551 | 1 |
| 7 | 4 | 0.636363636 | MB | LD | 0.923511479 | 1 |
| 4 | 0 | 1 | MB | LD | 1.570796327 | 1 |
| 8 | 1 | 0.888888889 | MB | LD | 1.230959417 | 1 |
| 6 | 0 | 1 | MB | LD | 1.570796327 | 1 |
| 2 | 2 | 0.5 | MB | LD | 0.785398163 | 1 |
| 6 | 1 | 0.857142857 | MB | LD | 1.18319964 | 1 |
| 2 | 0 | 1 | MB | LD | 1.570796327 | 1 |
| 6 | 0 | 1 | MB | LD | 1.570796327 | 1 |
| 7 | 1 | 0.875 | MB | LD | 1.209429203 | 1 |
| 3 | 0 | 1 | MB | LD | 1.570796327 | 1 |
| 3 | 0 | 1 | MB | LD | 1.570796327 | 1 |
| 6 | 0 | 1 | MB | LD | 1.570796327 | 1 |
| 1 | 4 | 0.2 | MB | LD | 0.463647609 | 1 |
| 7 | 0 | 1 | MB | LD | 1.570796327 | 1 |
| 3 | 1 | 0.75 | MB | LD | 1.047197551 | 1 |
| 5 | 0 | 1 | MB | LD | 1.570796327 | 1 |
| 10 | 0 | 1 | MB | LD | 1.570796327 | 1 |
| 6 | 0 | 1 | MB | LD | 1.570796327 | 1 |
| 7 | 2 | 0.777777778 | MB | LD | 1.079913649 | 1 |
| 7 | 1 | 0.875 | MB | LD | 1.209429203 | 1 |
| 3 | 1 | 0.75 | MB | LD | 1.047197551 | 1 |
| 2 | 0 | 1 | MB | LD | 1.570796327 | 1 |
| 4 | 1 | 0.8 | MB | LD | 1.107148718 | 1 |
| 4 | 2 | 0.666666667 | MB | LD | 0.955316618 | 1 |
| 2 | 3 | 0.4 | MB | LD | 0.684719203 | 1 |
| 6 | 0 | 1 | MB | LD | 1.570796327 | 1 |
| 3 | 0 | 1 | MB | LD | 1.570796327 | 1 |
| 8 | 2 | 0.8 | MB | LD | 1.107148718 | 1 |
| 10 | 1 | 0.909090909 | MB | LD | 1.264518958 | 1 |
| 9 | 0 | 1 | MB | LD | 1.570796327 | 1 |
| 5 | 0 | 1 | MB | LD | 1.570796327 | 1 |
| 3 | 0 | 1 | MB | LD | 1.570796327 | 1 |
| 9 | 1 | 0.9 | MB | LD | 1.249045772 | 1 |
| 7 | 1 | 0.875 | MB | LD | 1.209429203 | 1 |
| 4 | 1 | 0.8 | MB | LD | 1.107148718 | 1 |
| 1 | 4 | 0.2 | MB | LD | 0.463647609 | 1 |
| 5 | 1 | 0.833333333 | MB | LD | 1.150261992 | 1 |
| 5 | 0 | 1 | MB | LD | 1.570796327 | 1 |
| 5 | 0 | 1 | MB | LD | 1.570796327 | 1 |
| 7 | 2 | 0.777777778 | MB | LD | 1.079913649 | 1 |
| 4 | 2 | 0.666666667 | MB | LD | 0.955316618 | 1 |
| 4 | 4 | 0.5 | MB | LD | 0.785398163 | 1 |
| 1 | 7 | 0.125 | MB | HD | 0.36136712 | 2 |
| 1 | 5 | 0.16666667 | MB | HD | 0.42053434 | 2 |
| 1 | 5 | 0.16666667 | MB | HD | 0.42053434 | 2 |
| 1 | 5 | 0.16666667 | MB | HD | 0.42053434 | 2 |
| 2 | 9 | 0.18181818 | MB | HD | 0.44051066 | 2 |
| 2 | 5 | 0.28571429 | MB | HD | 0.56394264 | 2 |
| 2 | 5 | 0.28571429 | MB | HD | 0.56394264 | 2 |
| 3 | 7 | 0.3 | MB | HD | 0.57963974 | 2 |
| 3 | 7 | 0.3 | MB | HD | 0.57963974 | 2 |
| 1 | 2 | 0.33333333 | MB | LD | 0.61547971 | 2 |
| 2 | 4 | 0.33333333 | MB | HD | 0.61547971 | 2 |
| 2 | 3 | 0.4 | MB | HD | 0.6847192 | 2 |
| 2 | 3 | 0.4 | MB | HD | 0.6847192 | 2 |
| 3 | 4 | 0.42857143 | MB | HD | 0.71372438 | 2 |
| 4 | 5 | 0.44444444 | MB | HD | 0.72972766 | 2 |
| 10 | 11 | 0.47619048 | MB | LD | 0.76157963 | 2 |
| 1 | 1 | 0.5 | MB | HD | 0.78539816 | 2 |
| 2 | 2 | 0.5 | MB | HD | 0.78539816 | 2 |
| 2 | 2 | 0.5 | MB | HD | 0.78539816 | 2 |
| 2 | 2 | 0.5 | MB | HD | 0.78539816 | 2 |
| 2 | 2 | 0.5 | MB | HD | 0.78539816 | 2 |
| 2 | 2 | 0.5 | MB | HD | 0.78539816 | 2 |
| 3 | 3 | 0.5 | MB | LD | 0.78539816 | 2 |
| 3 | 3 | 0.5 | MB | LD | 0.78539816 | 2 |
| 3 | 3 | 0.5 | MB | HD | 0.78539816 | 2 |
| 3 | 3 | 0.5 | MB | HD | 0.78539816 | 2 |
| 5 | 5 | 0.5 | MB | HD | 0.78539816 | 2 |
| 5 | 5 | 0.5 | MB | HD | 0.78539816 | 2 |
| 4 | 3 | 0.57142857 | MB | LD | 0.85707195 | 2 |
| 4 | 3 | 0.57142857 | MB | LD | 0.85707195 | 2 |
| 4 | 3 | 0.57142857 | MB | HD | 0.85707195 | 2 |
| 4 | 3 | 0.57142857 | MB | HD | 0.85707195 | 2 |
| 4 | 3 | 0.57142857 | MB | HD | 0.85707195 | 2 |
| 4 | 3 | 0.57142857 | MB | HD | 0.85707195 | 2 |
| 4 | 3 | 0.57142857 | MB | HD | 0.85707195 | 2 |
| 3 | 2 | 0.6 | MB | LD | 0.88607712 | 2 |
| 3 | 2 | 0.6 | MB | HD | 0.88607712 | 2 |
| 5 | 3 | 0.625 | MB | HD | 0.91173829 | 2 |
| 12 | 7 | 0.63157895 | MB | LD | 0.91854517 | 2 |
| 2 | 1 | 0.66666667 | MB | HD | 0.95531662 | 2 |
| 4 | 2 | 0.66666667 | MB | LD | 0.95531662 | 2 |
| 4 | 2 | 0.66666667 | MB | HD | 0.95531662 | 2 |
| 4 | 2 | 0.66666667 | MB | HD | 0.95531662 | 2 |
| 4 | 2 | 0.66666667 | MB | HD | 0.95531662 | 2 |
| 4 | 2 | 0.66666667 | MB | HD | 0.95531662 | 2 |
| 4 | 2 | 0.66666667 | MB | HD | 0.95531662 | 2 |
| 6 | 3 | 0.66666667 | MB | LD | 0.95531662 | 2 |
| 6 | 3 | 0.66666667 | MB | LD | 0.95531662 | 2 |
| 6 | 3 | 0.66666667 | MB | HD | 0.95531662 | 2 |
| 11 | 5 | 0.6875 | MB | LD | 0.97759655 | 2 |
| 5 | 2 | 0.71428571 | MB | LD | 1.00685369 | 2 |
| 5 | 2 | 0.71428571 | MB | HD | 1.00685369 | 2 |
| 5 | 2 | 0.71428571 | MB | HD | 1.00685369 | 2 |
| 5 | 2 | 0.71428571 | MB | HD | 1.00685369 | 2 |
| 10 | 4 | 0.71428571 | MB | LD | 1.00685369 | 2 |
| 3 | 1 | 0.75 | MB | LD | 1.04719755 | 2 |
| 3 | 1 | 0.75 | MB | LD | 1.04719755 | 2 |
| 3 | 1 | 0.75 | MB | HD | 1.04719755 | 2 |
| 3 | 1 | 0.75 | MB | HD | 1.04719755 | 2 |
| 3 | 1 | 0.75 | MB | HD | 1.04719755 | 2 |
| 6 | 2 | 0.75 | MB | LD | 1.04719755 | 2 |
| 6 | 2 | 0.75 | MB | HD | 1.04719755 | 2 |
| 6 | 2 | 0.75 | MB | HD | 1.04719755 | 2 |
| 9 | 3 | 0.75 | MB | LD | 1.04719755 | 2 |
| 9 | 3 | 0.75 | MB | LD | 1.04719755 | 2 |
| 10 | 3 | 0.76923077 | MB | HD | 1.06970331 | 2 |
| 7 | 2 | 0.77777778 | MB | LD | 1.07991365 | 2 |
| 7 | 2 | 0.77777778 | MB | HD | 1.07991365 | 2 |
| 7 | 2 | 0.77777778 | MB | HD | 1.07991365 | 2 |
| 11 | 3 | 0.78571429 | MB | LD | 1.08952095 | 2 |
| 9 | 2 | 0.81818182 | MB | LD | 1.13028566 | 2 |
| 5 | 1 | 0.83333333 | MB | LD | 1.15026199 | 2 |
| 5 | 1 | 0.83333333 | MB | LD | 1.15026199 | 2 |
| 5 | 1 | 0.83333333 | MB | LD | 1.15026199 | 2 |
| 5 | 1 | 0.83333333 | MB | HD | 1.15026199 | 2 |
| 5 | 1 | 0.83333333 | MB | HD | 1.15026199 | 2 |
| 5 | 1 | 0.83333333 | MB | HD | 1.15026199 | 2 |
| 5 | 1 | 0.83333333 | MB | HD | 1.15026199 | 2 |
| 10 | 2 | 0.83333333 | MB | LD | 1.15026199 | 2 |
| 10 | 2 | 0.83333333 | MB | LD | 1.15026199 | 2 |
| 11 | 2 | 0.84615385 | MB | HD | 1.16773925 | 2 |
| 6 | 1 | 0.85714286 | MB | LD | 1.18319964 | 2 |
| 6 | 1 | 0.85714286 | MB | LD | 1.18319964 | 2 |
| 6 | 1 | 0.85714286 | MB | HD | 1.18319964 | 2 |
| 6 | 1 | 0.85714286 | MB | HD | 1.18319964 | 2 |
| 6 | 1 | 0.85714286 | MB | HD | 1.18319964 | 2 |
| 6 | 1 | 0.85714286 | MB | HD | 1.18319964 | 2 |
| 7 | 1 | 0.875 | MB | LD | 1.2094292 | 2 |
| 7 | 1 | 0.875 | MB | LD | 1.2094292 | 2 |
| 7 | 1 | 0.875 | MB | LD | 1.2094292 | 2 |
| 7 | 1 | 0.875 | MB | HD | 1.2094292 | 2 |
| 7 | 1 | 0.875 | MB | HD | 1.2094292 | 2 |
| 7 | 1 | 0.875 | MB | HD | 1.2094292 | 2 |
| 8 | 1 | 0.88888889 | MB | LD | 1.23095942 | 2 |
| 8 | 1 | 0.88888889 | MB | HD | 1.23095942 | 2 |
| 8 | 1 | 0.88888889 | MB | HD | 1.23095942 | 2 |
| 9 | 1 | 0.9 | MB | HD | 1.24904577 | 2 |
| 10 | 1 | 0.90909091 | MB | LD | 1.26451896 | 2 |
| 11 | 1 | 0.91666667 | MB | LD | 1.27795356 | 2 |
| 11 | 1 | 0.91666667 | MB | LD | 1.27795356 | 2 |
| 2 | 0 | 1 | MB | HD | 1.57079633 | 2 |
| 2 | 0 | 1 | MB | HD | 1.57079633 | 2 |
| 2 | 0 | 1 | MB | HD | 1.57079633 | 2 |
| 3 | 0 | 1 | MB | LD | 1.57079633 | 2 |
| 3 | 0 | 1 | MB | LD | 1.57079633 | 2 |
| 3 | 0 | 1 | MB | HD | 1.57079633 | 2 |
| 3 | 0 | 1 | MB | HD | 1.57079633 | 2 |
| 3 | 0 | 1 | MB | HD | 1.57079633 | 2 |
| 4 | 0 | 1 | MB | LD | 1.57079633 | 2 |
| 4 | 0 | 1 | MB | LD | 1.57079633 | 2 |
| 4 | 0 | 1 | MB | HD | 1.57079633 | 2 |
| 4 | 0 | 1 | MB | HD | 1.57079633 | 2 |
| 4 | 0 | 1 | MB | HD | 1.57079633 | 2 |
| 4 | 0 | 1 | MB | HD | 1.57079633 | 2 |
| 5 | 0 | 1 | MB | LD | 1.57079633 | 2 |
| 5 | 0 | 1 | MB | LD | 1.57079633 | 2 |
| 5 | 0 | 1 | MB | LD | 1.57079633 | 2 |
| 5 | 0 | 1 | MB | HD | 1.57079633 | 2 |
| 5 | 0 | 1 | MB | HD | 1.57079633 | 2 |
| 5 | 0 | 1 | MB | HD | 1.57079633 | 2 |
| 5 | 0 | 1 | MB | HD | 1.57079633 | 2 |
| 6 | 0 | 1 | MB | LD | 1.57079633 | 2 |
| 6 | 0 | 1 | MB | LD | 1.57079633 | 2 |
| 6 | 0 | 1 | MB | LD | 1.57079633 | 2 |
| 6 | 0 | 1 | MB | LD | 1.57079633 | 2 |
| 6 | 0 | 1 | MB | LD | 1.57079633 | 2 |
| 6 | 0 | 1 | MB | HD | 1.57079633 | 2 |
| 6 | 0 | 1 | MB | HD | 1.57079633 | 2 |
| 6 | 0 | 1 | MB | HD | 1.57079633 | 2 |
| 6 | 0 | 1 | MB | HD | 1.57079633 | 2 |
| 6 | 0 | 1 | MB | HD | 1.57079633 | 2 |
| 7 | 0 | 1 | MB | LD | 1.57079633 | 2 |
| 7 | 0 | 1 | MB | LD | 1.57079633 | 2 |
| 7 | 0 | 1 | MB | LD | 1.57079633 | 2 |
| 7 | 0 | 1 | MB | LD | 1.57079633 | 2 |
| 7 | 0 | 1 | MB | HD | 1.57079633 | 2 |
| 7 | 0 | 1 | MB | HD | 1.57079633 | 2 |
| 8 | 0 | 1 | MB | LD | 1.57079633 | 2 |
| 8 | 0 | 1 | MB | HD | 1.57079633 | 2 |
| 8 | 0 | 1 | MB | HD | 1.57079633 | 2 |
| 8 | 0 | 1 | MB | HD | 1.57079633 | 2 |
| 8 | 0 | 1 | MB | HD | 1.57079633 | 2 |
| 9 | 0 | 1 | MB | HD | 1.57079633 | 2 |
| 9 | 0 | 1 | MB | HD | 1.57079633 | 2 |
| 9 | 0 | 1 | MB | HD | 1.57079633 | 2 |
| 9 | 0 | 1 | MB | HD | 1.57079633 | 2 |
| 10 | 0 | 1 | MB | LD | 1.57079633 | 2 |
| 10 | 0 | 1 | MB | HD | 1.57079633 | 2 |
| 11 | 0 | 1 | MB | HD | 1.57079633 | 2 |
| 12 | 0 | 1 | MB | LD | 1.57079633 | 2 |
| 14 | 0 | 1 | MB | LD | 1.57079633 | 2 |
| 14 | 0 | 1 | MB | LD | 1.57079633 | 2 |
| 15 | 0 | 1 | MB | LD | 1.57079633 | 2 |
| 24 | 0 | 1 | MB | LD | 1.57079633 | 2 |
| 24 | 0 | 1 | MB | LD | 1.57079633 | 2 |
| 1 | 15 | 0.0625 | MB | LD | 0.252680255 | 3 |
| 1 | 12 | 0.076923077 | MB | HD | 0.281034902 | 3 |
| 1 | 12 | 0.076923077 | MB | LD | 0.281034902 | 3 |
| 3 | 16 | 0.157894737 | MB | LD | 0.408637855 | 3 |
| 2 | 10 | 0.166666667 | MB | HD | 0.420534335 | 3 |
| 3 | 15 | 0.166666667 | MB | HD | 0.420534335 | 3 |
| 4 | 20 | 0.166666667 | MB | LD | 0.420534335 | 3 |
| 3 | 13 | 0.1875 | MB | HD | 0.447832397 | 3 |
| 3 | 11 | 0.214285714 | MB | HD | 0.481275374 | 3 |
| 3 | 10 | 0.230769231 | MB | LD | 0.501093013 | 3 |
| 2 | 6 | 0.25 | MB | HD | 0.523598776 | 3 |
| 4 | 11 | 0.266666667 | MB | LD | 0.542639102 | 3 |
| 4 | 10 | 0.285714286 | MB | HD | 0.563942641 | 3 |
| 4 | 8 | 0.333333333 | MB | HD | 0.615479709 | 3 |
| 4 | 8 | 0.333333333 | MB | HD | 0.615479709 | 3 |
| 6 | 11 | 0.352941176 | MB | LD | 0.636132063 | 3 |
| 6 | 10 | 0.375 | MB | HD | 0.659058036 | 3 |
| 10 | 16 | 0.384615385 | MB | LD | 0.668964074 | 3 |
| 2 | 3 | 0.4 | MB | HD | 0.684719203 | 3 |
| 4 | 6 | 0.4 | MB | HD | 0.684719203 | 3 |
| 2 | 3 | 0.4 | MB | HD | 0.684719203 | 3 |
| 6 | 9 | 0.4 | MB | LD | 0.684719203 | 3 |
| 7 | 10 | 0.411764706 | MB | HD | 0.696698361 | 3 |
| 4 | 5 | 0.444444444 | MB | HD | 0.729727656 | 3 |
| 4 | 4 | 0.5 | MB | HD | 0.785398163 | 3 |
| 1 | 1 | 0.5 | MB | HD | 0.785398163 | 3 |
| 4 | 4 | 0.5 | MB | HD | 0.785398163 | 3 |
| 6 | 6 | 0.5 | MB | LD | 0.785398163 | 3 |
| 3 | 3 | 0.5 | MB | LD | 0.785398163 | 3 |
| 10 | 9 | 0.526315789 | MB | HD | 0.811726118 | 3 |
| 17 | 15 | 0.53125 | MB | LD | 0.816668544 | 3 |
| 8 | 7 | 0.533333333 | MB | HD | 0.818756238 | 3 |
| 8 | 7 | 0.533333333 | MB | LD | 0.818756238 | 3 |
| 7 | 6 | 0.538461538 | MB | HD | 0.823897734 | 3 |
| 6 | 5 | 0.545454545 | MB | LD | 0.830915552 | 3 |
| 5 | 4 | 0.555555556 | MB | HD | 0.841068671 | 3 |
| 8 | 6 | 0.571428571 | MB | HD | 0.857071948 | 3 |
| 7 | 5 | 0.583333333 | MB | HD | 0.869122203 | 3 |
| 10 | 7 | 0.588235294 | MB | HD | 0.874097966 | 3 |
| 6 | 4 | 0.6 | MB | HD | 0.886077124 | 3 |
| 9 | 6 | 0.6 | MB | LD | 0.886077124 | 3 |
| 8 | 5 | 0.615384615 | MB | HD | 0.901832253 | 3 |
| 10 | 6 | 0.625 | MB | HD | 0.911738291 | 3 |
| 7 | 4 | 0.636363636 | MB | LD | 0.923511479 | 3 |
| 9 | 5 | 0.642857143 | MB | HD | 0.930274014 | 3 |
| 11 | 6 | 0.647058824 | MB | HD | 0.934664264 | 3 |
| 8 | 4 | 0.666666667 | MB | HD | 0.955316618 | 3 |
| 6 | 3 | 0.666666667 | MB | HD | 0.955316618 | 3 |
| 8 | 4 | 0.666666667 | MB | HD | 0.955316618 | 3 |
| 10 | 5 | 0.666666667 | MB | HD | 0.955316618 | 3 |
| 6 | 3 | 0.666666667 | MB | HD | 0.955316618 | 3 |
| 8 | 4 | 0.666666667 | MB | HD | 0.955316618 | 3 |
| 10 | 5 | 0.666666667 | MB | HD | 0.955316618 | 3 |
| 12 | 6 | 0.666666667 | MB | HD | 0.955316618 | 3 |
| 8 | 4 | 0.666666667 | MB | HD | 0.955316618 | 3 |
| 6 | 3 | 0.666666667 | MB | HD | 0.955316618 | 3 |
| 6 | 3 | 0.666666667 | MB | HD | 0.955316618 | 3 |
| 6 | 3 | 0.666666667 | MB | LD | 0.955316618 | 3 |
| 10 | 5 | 0.666666667 | MB | LD | 0.955316618 | 3 |
| 6 | 3 | 0.666666667 | MB | LD | 0.955316618 | 3 |
| 9 | 4 | 0.692307692 | MB | HD | 0.982793723 | 3 |
| 16 | 7 | 0.695652174 | MB | HD | 0.98642243 | 3 |
| 7 | 3 | 0.7 | MB | HD | 0.991156586 | 3 |
| 8 | 3 | 0.727272727 | MB | HD | 1.021329082 | 3 |
| 8 | 3 | 0.727272727 | MB | LD | 1.021329082 | 3 |
| 11 | 4 | 0.733333333 | MB | LD | 1.028157225 | 3 |
| 9 | 3 | 0.75 | MB | HD | 1.047197551 | 3 |
| 12 | 4 | 0.75 | MB | HD | 1.047197551 | 3 |
| 9 | 3 | 0.75 | MB | HD | 1.047197551 | 3 |
| 9 | 3 | 0.75 | MB | HD | 1.047197551 | 3 |
| 12 | 4 | 0.75 | MB | HD | 1.047197551 | 3 |
| 9 | 3 | 0.75 | MB | LD | 1.047197551 | 3 |
| 13 | 4 | 0.764705882 | MB | HD | 1.064351683 | 3 |
| 10 | 3 | 0.769230769 | MB | HD | 1.069703314 | 3 |
| 10 | 3 | 0.769230769 | MB | LD | 1.069703314 | 3 |
| 11 | 3 | 0.785714286 | MB | HD | 1.089520953 | 3 |
| 8 | 2 | 0.8 | MB | HD | 1.107148718 | 3 |
| 9 | 2 | 0.818181818 | MB | HD | 1.130285664 | 3 |
| 9 | 2 | 0.818181818 | MB | HD | 1.130285664 | 3 |
| 14 | 3 | 0.823529412 | MB | HD | 1.137258417 | 3 |
| 10 | 2 | 0.833333333 | MB | HD | 1.150261992 | 3 |
| 5 | 1 | 0.833333333 | MB | HD | 1.150261992 | 3 |
| 11 | 2 | 0.846153846 | MB | HD | 1.167739252 | 3 |
| 11 | 2 | 0.846153846 | MB | HD | 1.167739252 | 3 |
| 11 | 2 | 0.846153846 | MB | HD | 1.167739252 | 3 |
| 11 | 2 | 0.846153846 | MB | HD | 1.167739252 | 3 |
| 17 | 3 | 0.85 | MB | LD | 1.173096912 | 3 |
| 6 | 1 | 0.857142857 | MB | HD | 1.18319964 | 3 |
| 12 | 2 | 0.857142857 | MB | HD | 1.18319964 | 3 |
| 6 | 1 | 0.857142857 | MB | LD | 1.18319964 | 3 |
| 13 | 2 | 0.866666667 | MB | HD | 1.197004152 | 3 |
| 13 | 2 | 0.866666667 | MB | LD | 1.197004152 | 3 |
| 7 | 1 | 0.875 | MB | HD | 1.209429203 | 3 |
| 7 | 1 | 0.875 | MB | HD | 1.209429203 | 3 |
| 7 | 1 | 0.875 | MB | HD | 1.209429203 | 3 |
| 7 | 1 | 0.875 | MB | HD | 1.209429203 | 3 |
| 7 | 1 | 0.875 | MB | HD | 1.209429203 | 3 |
| 8 | 1 | 0.888888889 | MB | HD | 1.230959417 | 3 |
| 8 | 1 | 0.888888889 | MB | HD | 1.230959417 | 3 |
| 8 | 1 | 0.888888889 | MB | HD | 1.230959417 | 3 |
| 8 | 1 | 0.888888889 | MB | LD | 1.230959417 | 3 |
| 8 | 1 | 0.888888889 | MB | LD | 1.230959417 | 3 |
| 19 | 2 | 0.904761905 | MB | HD | 1.25706844 | 3 |
| 10 | 1 | 0.909090909 | MB | HD | 1.264518958 | 3 |
| 10 | 1 | 0.909090909 | MB | LD | 1.264518958 | 3 |
| 10 | 1 | 0.909090909 | MB | LD | 1.264518958 | 3 |
| 10 | 1 | 0.909090909 | MB | LD | 1.264518958 | 3 |
| 11 | 1 | 0.916666667 | MB | LD | 1.277953555 | 3 |
| 12 | 1 | 0.923076923 | MB | HD | 1.289761425 | 3 |
| 12 | 1 | 0.923076923 | MB | LD | 1.289761425 | 3 |
| 16 | 1 | 0.941176471 | MB | LD | 1.325817664 | 3 |
| 16 | 1 | 0.941176471 | MB | LD | 1.325817664 | 3 |
| 17 | 1 | 0.944444444 | MB | LD | 1.332855202 | 3 |
| 20 | 1 | 0.952380952 | MB | HD | 1.350808349 | 3 |
| 22 | 1 | 0.956521739 | MB | LD | 1.360740588 | 3 |
| 10 | 0 | 1 | MB | HD | 1.570796327 | 3 |
| 14 | 0 | 1 | MB | HD | 1.570796327 | 3 |
| 10 | 0 | 1 | MB | HD | 1.570796327 | 3 |
| 16 | 0 | 1 | MB | HD | 1.570796327 | 3 |
| 11 | 0 | 1 | MB | HD | 1.570796327 | 3 |
| 10 | 0 | 1 | MB | HD | 1.570796327 | 3 |
| 8 | 0 | 1 | MB | HD | 1.570796327 | 3 |
| 7 | 0 | 1 | MB | HD | 1.570796327 | 3 |
| 10 | 0 | 1 | MB | HD | 1.570796327 | 3 |
| 10 | 0 | 1 | MB | HD | 1.570796327 | 3 |
| 18 | 0 | 1 | MB | HD | 1.570796327 | 3 |
| 12 | 0 | 1 | MB | HD | 1.570796327 | 3 |
| 13 | 0 | 1 | MB | HD | 1.570796327 | 3 |
| 10 | 0 | 1 | MB | HD | 1.570796327 | 3 |
| 9 | 0 | 1 | MB | HD | 1.570796327 | 3 |
| 4 | 0 | 1 | MB | HD | 1.570796327 | 3 |
| 3 | 0 | 1 | MB | HD | 1.570796327 | 3 |
| 4 | 0 | 1 | MB | LD | 1.570796327 | 3 |
| 10 | 0 | 1 | MB | LD | 1.570796327 | 3 |
| 10 | 0 | 1 | MB | LD | 1.570796327 | 3 |
| 14 | 0 | 1 | MB | LD | 1.570796327 | 3 |
| 11 | 0 | 1 | MB | LD | 1.570796327 | 3 |
| 14 | 0 | 1 | MB | LD | 1.570796327 | 3 |
| 10 | 0 | 1 | MB | LD | 1.570796327 | 3 |
| 5 | 0 | 1 | MB | LD | 1.570796327 | 3 |
| 9 | 0 | 1 | MB | LD | 1.570796327 | 4 |
| 14 | 0 | 1 | MB | LD | 1.570796327 | 4 |
| 8 | 0 | 1 | MB | LD | 1.570796327 | 4 |
| 5 | 0 | 1 | MB | LD | 1.570796327 | 4 |
| 3 | 0 | 1 | MB | LD | 1.570796327 | 4 |
| 7 | 0 | 1 | MB | LD | 1.570796327 | 4 |
| 6 | 0 | 1 | MB | LD | 1.570796327 | 4 |
| 4 | 0 | 1 | MB | LD | 1.570796327 | 4 |
| 17 | 0 | 1 | MB | LD | 1.570796327 | 4 |
| 9 | 0 | 1 | MB | LD | 1.570796327 | 4 |
| 30 | 0 | 1 | MB | LD | 1.570796327 | 4 |
| 33 | 0 | 1 | MB | LD | 1.570796327 | 4 |
| 7 | 0 | 1 | MB | LD | 1.570796327 | 4 |
| 10 | 0 | 1 | MB | LD | 1.570796327 | 4 |
| 15 | 0 | 1 | MB | LD | 1.570796327 | 4 |
| 11 | 0 | 1 | MB | LD | 1.570796327 | 4 |
| 4 | 0 | 1 | MB | LD | 1.570796327 | 4 |
| 10 | 0 | 1 | MB | LD | 1.570796327 | 4 |
| 4 | 0 | 1 | MB | LD | 1.570796327 | 4 |
| 10 | 0 | 1 | MB | LD | 1.570796327 | 4 |
| 4 | 0 | 1 | MB | LD | 1.570796327 | 4 |
| 9 | 0 | 1 | MB | LD | 1.570796327 | 4 |
| 6 | 0 | 1 | MB | LD | 1.570796327 | 4 |
| 9 | 0 | 1 | MB | LD | 1.570796327 | 4 |
| 14 | 0 | 1 | MB | LD | 1.570796327 | 4 |
| 7 | 0 | 1 | MB | LD | 1.570796327 | 4 |
| 9 | 0 | 1 | MB | LD | 1.570796327 | 4 |
| 6 | 0 | 1 | MB | LD | 1.570796327 | 4 |
| 2 | 0 | 1 | MB | LD | 1.570796327 | 4 |
| 1 | 0 | 1 | MB | HD | 1.570796327 | 4 |
| 10 | 0 | 1 | MB | HD | 1.570796327 | 4 |
| 7 | 0 | 1 | MB | HD | 1.570796327 | 4 |
| 14 | 0 | 1 | MB | HD | 1.570796327 | 4 |
| 9 | 0 | 1 | MB | HD | 1.570796327 | 4 |
| 8 | 0 | 1 | MB | HD | 1.570796327 | 4 |
| 11 | 0 | 1 | MB | HD | 1.570796327 | 4 |
| 4 | 0 | 1 | MB | HD | 1.570796327 | 4 |
| 7 | 0 | 1 | MB | HD | 1.570796327 | 4 |
| 13 | 0 | 1 | MB | HD | 1.570796327 | 4 |
| 6 | 0 | 1 | MB | HD | 1.570796327 | 4 |
| 11 | 0 | 1 | MB | HD | 1.570796327 | 4 |
| 9 | 0 | 1 | MB | HD | 1.570796327 | 4 |
| 10 | 0 | 1 | MB | HD | 1.570796327 | 4 |
| 3 | 0 | 1 | MB | HD | 1.570796327 | 4 |
| 8 | 0 | 1 | MB | HD | 1.570796327 | 4 |
| 12 | 0 | 1 | MB | HD | 1.570796327 | 4 |
| 9 | 0 | 1 | MB | HD | 1.570796327 | 4 |
| 9 | 0 | 1 | MB | HD | 1.570796327 | 4 |
| 6 | 0 | 1 | MB | HD | 1.570796327 | 4 |
| 3 | 0 | 1 | MB | HD | 1.570796327 | 4 |
| 1 | 0 | 1 | MB | HD | 1.570796327 | 4 |
| 10 | 0 | 1 | MB | HD | 1.570796327 | 4 |
| 6 | 0 | 1 | MB | HD | 1.570796327 | 4 |
| 5 | 0 | 1 | MB | HD | 1.570796327 | 4 |
| 8 | 0 | 1 | MB | HD | 1.570796327 | 4 |
| 8 | 0 | 1 | MB | HD | 1.570796327 | 4 |
| 3 | 0 | 1 | MB | HD | 1.570796327 | 4 |
| 15 | 0 | 1 | MB | HD | 1.570796327 | 4 |
| 7 | 0 | 1 | MB | HD | 1.570796327 | 4 |
| 27 | 2 | 0.931034483 | MB | LD | 1.305067208 | 4 |
| 13 | 1 | 0.928571429 | MB | LD | 1.300246564 | 4 |
| 13 | 1 | 0.928571429 | MB | LD | 1.300246564 | 4 |
| 12 | 1 | 0.923076923 | MB | HD | 1.289761425 | 4 |
| 11 | 1 | 0.916666667 | MB | LD | 1.277953555 | 4 |
| 10 | 1 | 0.909090909 | MB | LD | 1.264518958 | 4 |
| 10 | 1 | 0.909090909 | MB | HD | 1.264518958 | 4 |
| 9 | 1 | 0.9 | MB | LD | 1.249045772 | 4 |
| 9 | 1 | 0.9 | MB | LD | 1.249045772 | 4 |
| 9 | 1 | 0.9 | MB | HD | 1.249045772 | 4 |
| 9 | 1 | 0.9 | MB | HD | 1.249045772 | 4 |
| 9 | 1 | 0.9 | MB | HD | 1.249045772 | 4 |
| 8 | 1 | 0.888888889 | MB | LD | 1.230959417 | 4 |
| 8 | 1 | 0.888888889 | MB | HD | 1.230959417 | 4 |
| 8 | 1 | 0.888888889 | MB | HD | 1.230959417 | 4 |
| 8 | 1 | 0.888888889 | MB | HD | 1.230959417 | 4 |
| 8 | 1 | 0.888888889 | MB | HD | 1.230959417 | 4 |
| 15 | 2 | 0.882352941 | MB | HD | 1.220690549 | 4 |
| 7 | 1 | 0.875 | MB | LD | 1.209429203 | 4 |
| 7 | 1 | 0.875 | MB | LD | 1.209429203 | 4 |
| 7 | 1 | 0.875 | MB | HD | 1.209429203 | 4 |
| 14 | 2 | 0.875 | MB | HD | 1.209429203 | 4 |
| 7 | 1 | 0.875 | MB | HD | 1.209429203 | 4 |
| 25 | 4 | 0.862068966 | MB | LD | 1.19028995 | 4 |
| 6 | 1 | 0.857142857 | MB | LD | 1.18319964 | 4 |
| 12 | 2 | 0.857142857 | MB | HD | 1.18319964 | 4 |
| 6 | 1 | 0.857142857 | MB | HD | 1.18319964 | 4 |
| 6 | 1 | 0.857142857 | MB | HD | 1.18319964 | 4 |
| 6 | 1 | 0.857142857 | MB | HD | 1.18319964 | 4 |
| 6 | 1 | 0.857142857 | MB | HD | 1.18319964 | 4 |
| 6 | 1 | 0.857142857 | MB | HD | 1.18319964 | 4 |
| 6 | 1 | 0.857142857 | MB | HD | 1.18319964 | 4 |
| 40 | 7 | 0.85106383 | MB | LD | 1.174588755 | 4 |
| 11 | 2 | 0.846153846 | MB | LD | 1.167739252 | 4 |
| 11 | 2 | 0.846153846 | MB | HD | 1.167739252 | 4 |
| 5 | 1 | 0.833333333 | MB | LD | 1.150261992 | 4 |
| 5 | 1 | 0.833333333 | MB | LD | 1.150261992 | 4 |
| 5 | 1 | 0.833333333 | MB | LD | 1.150261992 | 4 |
| 5 | 1 | 0.833333333 | MB | HD | 1.150261992 | 4 |
| 10 | 2 | 0.833333333 | MB | HD | 1.150261992 | 4 |
| 5 | 1 | 0.833333333 | MB | HD | 1.150261992 | 4 |
| 5 | 1 | 0.833333333 | MB | HD | 1.150261992 | 4 |
| 5 | 1 | 0.833333333 | MB | HD | 1.150261992 | 4 |
| 5 | 1 | 0.833333333 | MB | HD | 1.150261992 | 4 |
| 5 | 1 | 0.833333333 | MB | HD | 1.150261992 | 4 |
| 5 | 1 | 0.833333333 | MB | HD | 1.150261992 | 4 |
| 27 | 6 | 0.818181818 | MB | LD | 1.130285664 | 4 |
| 9 | 2 | 0.818181818 | MB | LD | 1.130285664 | 4 |
| 9 | 2 | 0.818181818 | MB | HD | 1.130285664 | 4 |
| 4 | 1 | 0.8 | MB | LD | 1.107148718 | 4 |
| 8 | 2 | 0.8 | MB | HD | 1.107148718 | 4 |
| 8 | 2 | 0.8 | MB | HD | 1.107148718 | 4 |
| 8 | 2 | 0.8 | MB | HD | 1.107148718 | 4 |
| 4 | 1 | 0.8 | MB | HD | 1.107148718 | 4 |
| 10 | 3 | 0.769230769 | MB | HD | 1.069703314 | 4 |
| 3 | 1 | 0.75 | MB | LD | 1.047197551 | 4 |
| 6 | 2 | 0.75 | MB | LD | 1.047197551 | 4 |
| 6 | 2 | 0.75 | MB | HD | 1.047197551 | 4 |
| 3 | 1 | 0.75 | MB | HD | 1.047197551 | 4 |
| 6 | 2 | 0.75 | MB | HD | 1.047197551 | 4 |
| 6 | 2 | 0.75 | MB | HD | 1.047197551 | 4 |
| 6 | 2 | 0.75 | MB | HD | 1.047197551 | 4 |
| 6 | 2 | 0.75 | MB | HD | 1.047197551 | 4 |
| 23 | 9 | 0.71875 | MB | HD | 1.011806461 | 4 |
| 5 | 2 | 0.714285714 | MB | HD | 1.006853685 | 4 |
| 28 | 13 | 0.682926829 | MB | LD | 0.972673107 | 4 |
| 2 | 1 | 0.666666667 | MB | HD | 0.955316618 | 4 |
| 2 | 1 | 0.666666667 | MB | HD | 0.955316618 | 4 |
| 6 | 3 | 0.666666667 | MB | HD | 0.955316618 | 4 |
| 6 | 3 | 0.666666667 | MB | HD | 0.955316618 | 4 |
| 7 | 4 | 0.636363636 | MB | HD | 0.923511479 | 4 |
| 5 | 3 | 0.625 | MB | HD | 0.911738291 | 4 |
| 5 | 3 | 0.625 | MB | HD | 0.911738291 | 4 |
| 3 | 2 | 0.6 | MB | HD | 0.886077124 | 4 |
| 3 | 2 | 0.6 | MB | HD | 0.886077124 | 4 |
| 4 | 3 | 0.571428571 | MB | HD | 0.857071948 | 4 |
| 4 | 3 | 0.571428571 | MB | HD | 0.857071948 | 4 |
| 4 | 3 | 0.571428571 | MB | HD | 0.857071948 | 4 |
| 3 | 3 | 0.5 | MB | HD | 0.785398163 | 4 |
| 5 | 6 | 0.454545455 | MB | LD | 0.739880774 | 4 |
| 3 | 4 | 0.428571429 | MB | LD | 0.713724379 | 4 |
| 3 | 4 | 0.428571429 | MB | HD | 0.713724379 | 4 |
| 2 | 3 | 0.4 | MB | HD | 0.684719203 | 4 |
| 3 | 5 | 0.375 | MB | LD | 0.659058036 | 4 |
| 3 | 5 | 0.375 | MB | LD | 0.659058036 | 4 |
| 1 | 2 | 0.333333333 | MB | HD | 0.615479709 | 4 |
| 3 | 7 | 0.3 | MB | LD | 0.57963974 | 4 |
| 4 | 10 | 0.285714286 | MB | HD | 0.563942641 | 4 |
| 2 | 7 | 0.222222222 | MB | HD | 0.490882678 | 4 |
| 2 | 8 | 0.2 | MB | HD | 0.463647609 | 4 |
| 2 | 12 | 0.142857143 | MB | HD | 0.387596687 | 4 |
| 1 | 11 | 0.083333333 | MB | HD | 0.292842772 | 4 |

Supplementary Table 6: Sperm offence assay, 9 day old males, non-transformed data with red and white eye values

| **Red** | **White** | **Selection** | **Treatment** | **P2** | **P2 transformed** | **Block** |
| --- | --- | --- | --- | --- | --- | --- |
| 26 | 0 | MB | LD | 1 | 1.570796327 | 1 |
| 25 | 0 | MB | HD | 1 | 1.570796327 | 1 |
| 23 | 0 | MCU | LD | 1 | 1.570796327 | 4 |
| 23 | 1 | MCU | LD | 0.958333333 | 1.365227396 | 2 |
| 23 | 3 | MCU | HD | 0.884615385 | 1.224216373 | 1 |
| 23 | 1 | MB | HD | 0.958333333 | 1.365227396 | 1 |
| 23 | 0 | MB | LD | 1 | 1.570796327 | 1 |
| 22 | 1 | MB | LD | 0.956521739 | 1.360740588 | 2 |
| 22 | 2 | MCU | LD | 0.916666667 | 1.277953555 | 1 |
| 21 | 1 | MCU | LD | 0.954545455 | 1.355946494 | 3 |
| 21 | 3 | MB | HD | 0.875 | 1.209429203 | 3 |
| 21 | 2 | MB | HD | 0.913043478 | 1.2714623 | 3 |
| 21 | 1 | MCU | HD | 0.954545455 | 1.355946494 | 2 |
| 21 | 1 | MB | HD | 0.954545455 | 1.355946494 | 2 |
| 21 | 3 | MB | HD | 0.875 | 1.209429203 | 1 |
| 20 | 3 | MB | HD | 0.869565217 | 1.201287387 | 4 |
| 20 | 0 | MCU | LD | 1 | 1.570796327 | 1 |
| 20 | 0 | MCU | LD | 1 | 1.570796327 | 1 |
| 19 | 5 | MCU | HD | 0.791666667 | 1.096811456 | 3 |
| 19 | 1 | MB | LD | 0.95 | 1.345282921 | 2 |
| 18 | 0 | MB | LD | 1 | 1.570796327 | 4 |
| 18 | 3 | MCU | HD | 0.857142857 | 1.18319964 | 3 |
| 18 | 2 | MB | HD | 0.9 | 1.249045772 | 3 |
| 18 | 0 | MB | LD | 1 | 1.570796327 | 3 |
| 18 | 0 | MB | HD | 1 | 1.570796327 | 3 |
| 18 | 0 | MCU | HD | 1 | 1.570796327 | 2 |
| 18 | 2 | MB | HD | 0.9 | 1.249045772 | 2 |
| 17 | 4 | MCU | LD | 0.80952381 | 1.119162886 | 4 |
| 17 | 1 | MCU | HD | 0.944444444 | 1.332855202 | 3 |
| 17 | 0 | MCU | LD | 1 | 1.570796327 | 3 |
| 17 | 0 | MCU | LD | 1 | 1.570796327 | 3 |
| 17 | 3 | MB | LD | 0.85 | 1.173096912 | 3 |
| 17 | 0 | MB | HD | 1 | 1.570796327 | 3 |
| 17 | 2 | MCU | LD | 0.894736842 | 1.240373679 | 2 |
| 17 | 1 | MCU | HD | 0.944444444 | 1.332855202 | 2 |
| 17 | 0 | MCU | LD | 1 | 1.570796327 | 2 |
| 17 | 1 | MB | LD | 0.944444444 | 1.332855202 | 2 |
| 17 | 7 | MCU | HD | 0.708333333 | 1.000285879 | 1 |
| 17 | 3 | MCU | LD | 0.85 | 1.173096912 | 1 |
| 17 | 0 | MCU | LD | 1 | 1.570796327 | 1 |
| 17 | 0 | MCU | HD | 1 | 1.570796327 | 1 |
| 17 | 4 | MB | HD | 0.80952381 | 1.119162886 | 1 |
| 17 | 2 | MB | HD | 0.894736842 | 1.240373679 | 1 |
| 17 | 1 | MB | LD | 0.944444444 | 1.332855202 | 1 |
| 17 | 0 | MB | HD | 1 | 1.570796327 | 1 |
| 16 | 3 | MCU | LD | 0.842105263 | 1.162158472 | 4 |
| 16 | 0 | MCU | LD | 1 | 1.570796327 | 4 |
| 16 | 3 | MB | LD | 0.842105263 | 1.162158472 | 4 |
| 16 | 3 | MCU | HD | 0.842105263 | 1.162158472 | 3 |
| 16 | 2 | MCU | LD | 0.888888889 | 1.230959417 | 3 |
| 16 | 1 | MCU | LD | 0.941176471 | 1.325817664 | 3 |
| 16 | 4 | MB | HD | 0.8 | 1.107148718 | 3 |
| 16 | 1 | MCU | HD | 0.941176471 | 1.325817664 | 2 |
| 16 | 4 | MB | LD | 0.8 | 1.107148718 | 2 |
| 16 | 6 | MCU | HD | 0.727272727 | 1.021329082 | 1 |
| 16 | 5 | MCU | LD | 0.761904762 | 1.061056648 | 1 |
| 16 | 0 | MCU | LD | 1 | 1.570796327 | 1 |
| 15 | 5 | MB | LD | 0.75 | 1.047197551 | 4 |
| 15 | 1 | MB | HD | 0.9375 | 1.318116072 | 4 |
| 15 | 1 | MB | HD | 0.9375 | 1.318116072 | 4 |
| 15 | 0 | MCU | LD | 1 | 1.570796327 | 3 |
| 15 | 5 | MB | HD | 0.75 | 1.047197551 | 3 |
| 15 | 2 | MB | LD | 0.882352941 | 1.220690549 | 3 |
| 15 | 2 | MB | LD | 0.882352941 | 1.220690549 | 3 |
| 15 | 1 | MCU | HD | 0.9375 | 1.318116072 | 1 |
| 15 | 0 | MCU | LD | 1 | 1.570796327 | 1 |
| 15 | 0 | MCU | HD | 1 | 1.570796327 | 1 |
| 14 | 0 | MCU | LD | 1 | 1.570796327 | 4 |
| 14 | 1 | MCU | HD | 0.933333333 | 1.309638916 | 3 |
| 14 | 0 | MCU | HD | 1 | 1.570796327 | 3 |
| 14 | 3 | MB | LD | 0.823529412 | 1.137258417 | 3 |
| 14 | 0 | MB | LD | 1 | 1.570796327 | 3 |
| 14 | 2 | MCU | HD | 0.875 | 1.209429203 | 2 |
| 14 | 4 | MB | LD | 0.777777778 | 1.079913649 | 2 |
| 14 | 2 | MB | HD | 0.875 | 1.209429203 | 2 |
| 14 | 1 | MB | LD | 0.933333333 | 1.309638916 | 2 |
| 14 | 1 | MB | LD | 0.933333333 | 1.309638916 | 2 |
| 14 | 0 | MB | LD | 1 | 1.570796327 | 2 |
| 14 | 0 | MB | HD | 1 | 1.570796327 | 2 |
| 14 | 5 | MCU | HD | 0.736842105 | 1.032132861 | 1 |
| 14 | 2 | MCU | LD | 0.875 | 1.209429203 | 1 |
| 14 | 0 | MCU | HD | 1 | 1.570796327 | 1 |
| 14 | 7 | MB | HD | 0.666666667 | 0.955316618 | 1 |
| 14 | 1 | MB | LD | 0.933333333 | 1.309638916 | 1 |
| 14 | 1 | MB | HD | 0.933333333 | 1.309638916 | 1 |
| 14 | 0 | MB | LD | 1 | 1.570796327 | 1 |
| 14 | 0 | MB | HD | 1 | 1.570796327 | 1 |
| 13 | 1 | MCU | LD | 0.928571429 | 1.300246564 | 4 |
| 13 | 0 | MCU | LD | 1 | 1.570796327 | 4 |
| 13 | 0 | MCU | HD | 1 | 1.570796327 | 4 |
| 13 | 12 | MB | LD | 0.52 | 0.805403501 | 4 |
| 13 | 0 | MB | HD | 1 | 1.570796327 | 4 |
| 13 | 7 | MCU | HD | 0.65 | 0.93774449 | 3 |
| 13 | 6 | MCU | LD | 0.684210526 | 0.97405318 | 3 |
| 13 | 4 | MCU | LD | 0.764705882 | 1.064351683 | 3 |
| 13 | 3 | MCU | LD | 0.8125 | 1.12296393 | 3 |
| 13 | 2 | MCU | LD | 0.866666667 | 1.197004152 | 3 |
| 13 | 0 | MCU | LD | 1 | 1.570796327 | 3 |
| 13 | 0 | MCU | HD | 1 | 1.570796327 | 3 |
| 13 | 4 | MB | HD | 0.764705882 | 1.064351683 | 3 |
| 13 | 3 | MB | HD | 0.8125 | 1.12296393 | 3 |
| 13 | 2 | MB | LD | 0.866666667 | 1.197004152 | 3 |
| 13 | 0 | MB | HD | 1 | 1.570796327 | 3 |
| 13 | 0 | MB | HD | 1 | 1.570796327 | 3 |
| 13 | 4 | MCU | LD | 0.764705882 | 1.064351683 | 2 |
| 13 | 4 | MCU | HD | 0.764705882 | 1.064351683 | 2 |
| 13 | 2 | MCU | HD | 0.866666667 | 1.197004152 | 2 |
| 13 | 0 | MCU | LD | 1 | 1.570796327 | 2 |
| 13 | 0 | MCU | LD | 1 | 1.570796327 | 2 |
| 13 | 0 | MCU | HD | 1 | 1.570796327 | 2 |
| 13 | 0 | MB | LD | 1 | 1.570796327 | 2 |
| 13 | 0 | MB | LD | 1 | 1.570796327 | 2 |
| 13 | 0 | MB | HD | 1 | 1.570796327 | 2 |
| 13 | 0 | MB | HD | 1 | 1.570796327 | 2 |
| 13 | 2 | MCU | HD | 0.866666667 | 1.197004152 | 1 |
| 13 | 1 | MCU | LD | 0.928571429 | 1.300246564 | 1 |
| 13 | 0 | MCU | LD | 1 | 1.570796327 | 1 |
| 13 | 0 | MCU | HD | 1 | 1.570796327 | 1 |
| 13 | 10 | MB | HD | 0.565217391 | 0.850801911 | 1 |
| 13 | 4 | MB | LD | 0.764705882 | 1.064351683 | 1 |
| 13 | 1 | MB | HD | 0.928571429 | 1.300246564 | 1 |
| 13 | 1 | MB | HD | 0.928571429 | 1.300246564 | 1 |
| 13 | 1 | MB | HD | 0.928571429 | 1.300246564 | 1 |
| 12 | 3 | MCU | HD | 0.8 | 1.107148718 | 4 |
| 12 | 2 | MCU | LD | 0.857142857 | 1.18319964 | 4 |
| 12 | 2 | MCU | LD | 0.857142857 | 1.18319964 | 4 |
| 12 | 1 | MCU | HD | 0.923076923 | 1.289761425 | 4 |
| 12 | 0 | MCU | HD | 1 | 1.570796327 | 4 |
| 12 | 1 | MB | LD | 0.923076923 | 1.289761425 | 4 |
| 12 | 9 | MCU | HD | 0.571428571 | 0.857071948 | 3 |
| 12 | 2 | MCU | LD | 0.857142857 | 1.18319964 | 3 |
| 12 | 2 | MCU | HD | 0.857142857 | 1.18319964 | 3 |
| 12 | 2 | MCU | HD | 0.857142857 | 1.18319964 | 3 |
| 12 | 1 | MCU | HD | 0.923076923 | 1.289761425 | 3 |
| 12 | 0 | MCU | LD | 1 | 1.570796327 | 3 |
| 12 | 0 | MCU | HD | 1 | 1.570796327 | 3 |
| 12 | 3 | MB | HD | 0.8 | 1.107148718 | 3 |
| 12 | 0 | MB | LD | 1 | 1.570796327 | 3 |
| 12 | 0 | MB | LD | 1 | 1.570796327 | 3 |
| 12 | 0 | MB | HD | 1 | 1.570796327 | 3 |
| 12 | 0 | MB | HD | 1 | 1.570796327 | 3 |
| 12 | 2 | MCU | LD | 0.857142857 | 1.18319964 | 2 |
| 12 | 0 | MCU | LD | 1 | 1.570796327 | 2 |
| 12 | 1 | MB | LD | 0.923076923 | 1.289761425 | 2 |
| 12 | 0 | MB | LD | 1 | 1.570796327 | 2 |
| 12 | 0 | MB | LD | 1 | 1.570796327 | 2 |
| 12 | 0 | MB | HD | 1 | 1.570796327 | 2 |
| 12 | 4 | MCU | HD | 0.75 | 1.047197551 | 1 |
| 12 | 3 | MCU | HD | 0.8 | 1.107148718 | 1 |
| 12 | 6 | MB | HD | 0.666666667 | 0.955316618 | 1 |
| 12 | 2 | MB | HD | 0.857142857 | 1.18319964 | 1 |
| 12 | 1 | MB | HD | 0.923076923 | 1.289761425 | 1 |
| 12 | 1 | MB | HD | 0.923076923 | 1.289761425 | 1 |
| 11 | 1 | MCU | HD | 0.916666667 | 1.277953555 | 4 |
| 11 | 0 | MCU | LD | 1 | 1.570796327 | 4 |
| 11 | 3 | MB | HD | 0.785714286 | 1.089520953 | 4 |
| 11 | 3 | MB | HD | 0.785714286 | 1.089520953 | 4 |
| 11 | 1 | MB | LD | 0.916666667 | 1.277953555 | 4 |
| 11 | 1 | MB | LD | 0.916666667 | 1.277953555 | 4 |
| 11 | 0 | MB | LD | 1 | 1.570796327 | 4 |
| 11 | 0 | MB | HD | 1 | 1.570796327 | 4 |
| 11 | 4 | MCU | LD | 0.733333333 | 1.028157225 | 3 |
| 11 | 3 | MCU | LD | 0.785714286 | 1.089520953 | 3 |
| 11 | 3 | MCU | HD | 0.785714286 | 1.089520953 | 3 |
| 11 | 1 | MCU | HD | 0.916666667 | 1.277953555 | 3 |
| 11 | 0 | MCU | HD | 1 | 1.570796327 | 3 |
| 11 | 0 | MCU | HD | 1 | 1.570796327 | 3 |
| 11 | 0 | MCU | HD | 1 | 1.570796327 | 3 |
| 11 | 0 | MCU | HD | 1 | 1.570796327 | 3 |
| 11 | 0 | MCU | HD | 1 | 1.570796327 | 3 |
| 11 | 6 | MB | HD | 0.647058824 | 0.934664264 | 3 |
| 11 | 5 | MB | HD | 0.6875 | 0.977596551 | 3 |
| 11 | 4 | MB | HD | 0.733333333 | 1.028157225 | 3 |
| 11 | 1 | MB | HD | 0.916666667 | 1.277953555 | 3 |
| 11 | 0 | MB | LD | 1 | 1.570796327 | 3 |
| 11 | 0 | MB | LD | 1 | 1.570796327 | 3 |
| 11 | 0 | MCU | LD | 1 | 1.570796327 | 2 |
| 11 | 6 | MB | HD | 0.647058824 | 0.934664264 | 2 |
| 11 | 5 | MB | HD | 0.6875 | 0.977596551 | 2 |
| 11 | 2 | MB | HD | 0.846153846 | 1.167739252 | 2 |
| 11 | 2 | MB | HD | 0.846153846 | 1.167739252 | 2 |
| 11 | 1 | MB | HD | 0.916666667 | 1.277953555 | 2 |
| 11 | 0 | MB | LD | 1 | 1.570796327 | 2 |
| 11 | 0 | MB | HD | 1 | 1.570796327 | 2 |
| 11 | 4 | MCU | LD | 0.733333333 | 1.028157225 | 1 |
| 11 | 3 | MCU | LD | 0.785714286 | 1.089520953 | 1 |
| 11 | 3 | MCU | HD | 0.785714286 | 1.089520953 | 1 |
| 11 | 3 | MCU | HD | 0.785714286 | 1.089520953 | 1 |
| 11 | 2 | MCU | LD | 0.846153846 | 1.167739252 | 1 |
| 11 | 2 | MCU | LD | 0.846153846 | 1.167739252 | 1 |
| 11 | 2 | MCU | HD | 0.846153846 | 1.167739252 | 1 |
| 11 | 2 | MCU | HD | 0.846153846 | 1.167739252 | 1 |
| 11 | 1 | MCU | LD | 0.916666667 | 1.277953555 | 1 |
| 11 | 1 | MCU | LD | 0.916666667 | 1.277953555 | 1 |
| 11 | 1 | MCU | HD | 0.916666667 | 1.277953555 | 1 |
| 11 | 1 | MCU | HD | 0.916666667 | 1.277953555 | 1 |
| 11 | 0 | MCU | HD | 1 | 1.570796327 | 1 |
| 11 | 5 | MB | LD | 0.6875 | 0.977596551 | 1 |
| 11 | 3 | MB | LD | 0.785714286 | 1.089520953 | 1 |
| 11 | 3 | MB | HD | 0.785714286 | 1.089520953 | 1 |
| 11 | 0 | MB | HD | 1 | 1.570796327 | 1 |
| 11 | 0 | MB | HD | 1 | 1.570796327 | 1 |
| 11 | 0 | MB | HD | 1 | 1.570796327 | 1 |
| 11 | 0 | MB | HD | 1 | 1.570796327 | 1 |
| 10 | 7 | MCU | HD | 0.588235294 | 0.874097966 | 4 |
| 10 | 3 | MCU | LD | 0.769230769 | 1.069703314 | 4 |
| 10 | 1 | MCU | LD | 0.909090909 | 1.264518958 | 4 |
| 10 | 1 | MCU | HD | 0.909090909 | 1.264518958 | 4 |
| 10 | 0 | MCU | HD | 1 | 1.570796327 | 4 |
| 10 | 8 | MB | HD | 0.555555556 | 0.841068671 | 4 |
| 10 | 4 | MB | LD | 0.714285714 | 1.006853685 | 4 |
| 10 | 2 | MB | HD | 0.833333333 | 1.150261992 | 4 |
| 10 | 1 | MB | LD | 0.909090909 | 1.264518958 | 4 |
| 10 | 1 | MB | HD | 0.909090909 | 1.264518958 | 4 |
| 10 | 1 | MB | HD | 0.909090909 | 1.264518958 | 4 |
| 10 | 0 | MB | LD | 1 | 1.570796327 | 4 |
| 10 | 11 | MCU | HD | 0.476190476 | 0.761579632 | 3 |
| 10 | 2 | MCU | LD | 0.833333333 | 1.150261992 | 3 |
| 10 | 1 | MCU | LD | 0.909090909 | 1.264518958 | 3 |
| 10 | 1 | MCU | HD | 0.909090909 | 1.264518958 | 3 |
| 10 | 0 | MCU | HD | 1 | 1.570796327 | 3 |
| 10 | 6 | MB | HD | 0.625 | 0.911738291 | 3 |
| 10 | 2 | MB | HD | 0.833333333 | 1.150261992 | 3 |
| 10 | 1 | MB | HD | 0.909090909 | 1.264518958 | 3 |
| 10 | 1 | MB | HD | 0.909090909 | 1.264518958 | 3 |
| 10 | 0 | MB | HD | 1 | 1.570796327 | 3 |
| 10 | 0 | MB | HD | 1 | 1.570796327 | 3 |
| 10 | 0 | MB | HD | 1 | 1.570796327 | 3 |
| 10 | 3 | MCU | LD | 0.769230769 | 1.069703314 | 2 |
| 10 | 1 | MCU | LD | 0.909090909 | 1.264518958 | 2 |
| 10 | 0 | MCU | HD | 1 | 1.570796327 | 2 |
| 10 | 0 | MCU | HD | 1 | 1.570796327 | 2 |
| 10 | 0 | MCU | HD | 1 | 1.570796327 | 2 |
| 10 | 5 | MB | HD | 0.666666667 | 0.955316618 | 2 |
| 10 | 1 | MB | LD | 0.909090909 | 1.264518958 | 2 |
| 10 | 1 | MB | HD | 0.909090909 | 1.264518958 | 2 |
| 10 | 1 | MB | HD | 0.909090909 | 1.264518958 | 2 |
| 10 | 0 | MB | LD | 1 | 1.570796327 | 2 |
| 10 | 5 | MCU | LD | 0.666666667 | 0.955316618 | 1 |
| 10 | 5 | MCU | LD | 0.666666667 | 0.955316618 | 1 |
| 10 | 2 | MCU | LD | 0.833333333 | 1.150261992 | 1 |
| 10 | 1 | MCU | HD | 0.909090909 | 1.264518958 | 1 |
| 10 | 10 | MB | HD | 0.5 | 0.785398163 | 1 |
| 10 | 5 | MB | HD | 0.666666667 | 0.955316618 | 1 |
| 10 | 5 | MB | HD | 0.666666667 | 0.955316618 | 1 |
| 10 | 3 | MB | HD | 0.769230769 | 1.069703314 | 1 |
| 10 | 3 | MB | HD | 0.769230769 | 1.069703314 | 1 |
| 10 | 0 | MB | LD | 1 | 1.570796327 | 1 |
| 10 | 0 | MB | HD | 1 | 1.570796327 | 1 |
| 9 | 1 | MCU | LD | 0.9 | 1.249045772 | 4 |
| 9 | 0 | MCU | LD | 1 | 1.570796327 | 4 |
| 9 | 0 | MCU | LD | 1 | 1.570796327 | 4 |
| 9 | 5 | MB | HD | 0.642857143 | 0.930274014 | 4 |
| 9 | 1 | MB | HD | 0.9 | 1.249045772 | 4 |
| 9 | 0 | MB | HD | 1 | 1.570796327 | 4 |
| 9 | 0 | MB | HD | 1 | 1.570796327 | 4 |
| 9 | 5 | MCU | HD | 0.642857143 | 0.930274014 | 3 |
| 9 | 3 | MCU | HD | 0.75 | 1.047197551 | 3 |
| 9 | 2 | MCU | HD | 0.818181818 | 1.130285664 | 3 |
| 9 | 2 | MCU | HD | 0.818181818 | 1.130285664 | 3 |
| 9 | 1 | MCU | HD | 0.9 | 1.249045772 | 3 |
| 9 | 1 | MCU | HD | 0.9 | 1.249045772 | 3 |
| 9 | 0 | MCU | LD | 1 | 1.570796327 | 3 |
| 9 | 0 | MCU | LD | 1 | 1.570796327 | 3 |
| 9 | 9 | MB | HD | 0.5 | 0.785398163 | 3 |
| 9 | 5 | MB | HD | 0.642857143 | 0.930274014 | 3 |
| 9 | 4 | MB | LD | 0.692307692 | 0.982793723 | 3 |
| 9 | 2 | MB | LD | 0.818181818 | 1.130285664 | 3 |
| 9 | 2 | MB | LD | 0.818181818 | 1.130285664 | 3 |
| 9 | 2 | MB | HD | 0.818181818 | 1.130285664 | 3 |
| 9 | 1 | MB | HD | 0.9 | 1.249045772 | 3 |
| 9 | 1 | MB | HD | 0.9 | 1.249045772 | 3 |
| 9 | 1 | MB | HD | 0.9 | 1.249045772 | 3 |
| 9 | 1 | MB | HD | 0.9 | 1.249045772 | 3 |
| 9 | 6 | MCU | HD | 0.6 | 0.886077124 | 2 |
| 9 | 3 | MCU | LD | 0.75 | 1.047197551 | 2 |
| 9 | 3 | MCU | LD | 0.75 | 1.047197551 | 2 |
| 9 | 1 | MCU | LD | 0.9 | 1.249045772 | 2 |
| 9 | 1 | MCU | LD | 0.9 | 1.249045772 | 2 |
| 9 | 1 | MCU | LD | 0.9 | 1.249045772 | 2 |
| 9 | 1 | MCU | HD | 0.9 | 1.249045772 | 2 |
| 9 | 0 | MCU | LD | 1 | 1.570796327 | 2 |
| 9 | 0 | MCU | HD | 1 | 1.570796327 | 2 |
| 9 | 5 | MB | LD | 0.642857143 | 0.930274014 | 2 |
| 9 | 3 | MB | HD | 0.75 | 1.047197551 | 2 |
| 9 | 2 | MB | HD | 0.818181818 | 1.130285664 | 2 |
| 9 | 0 | MB | LD | 1 | 1.570796327 | 2 |
| 9 | 0 | MB | LD | 1 | 1.570796327 | 2 |
| 9 | 0 | MB | LD | 1 | 1.570796327 | 2 |
| 9 | 0 | MB | LD | 1 | 1.570796327 | 2 |
| 9 | 0 | MB | HD | 1 | 1.570796327 | 2 |
| 9 | 0 | MB | HD | 1 | 1.570796327 | 2 |
| 9 | 10 | MCU | LD | 0.473684211 | 0.759070209 | 1 |
| 9 | 5 | MCU | HD | 0.642857143 | 0.930274014 | 1 |
| 9 | 2 | MCU | HD | 0.818181818 | 1.130285664 | 1 |
| 9 | 2 | MCU | HD | 0.818181818 | 1.130285664 | 1 |
| 9 | 1 | MCU | LD | 0.9 | 1.249045772 | 1 |
| 9 | 1 | MCU | LD | 0.9 | 1.249045772 | 1 |
| 9 | 1 | MCU | HD | 0.9 | 1.249045772 | 1 |
| 9 | 1 | MCU | HD | 0.9 | 1.249045772 | 1 |
| 9 | 1 | MCU | HD | 0.9 | 1.249045772 | 1 |
| 9 | 1 | MCU | HD | 0.9 | 1.249045772 | 1 |
| 9 | 0 | MCU | HD | 1 | 1.570796327 | 1 |
| 9 | 0 | MCU | HD | 1 | 1.570796327 | 1 |
| 9 | 9 | MB | HD | 0.5 | 0.785398163 | 1 |
| 9 | 4 | MB | LD | 0.692307692 | 0.982793723 | 1 |
| 9 | 2 | MB | HD | 0.818181818 | 1.130285664 | 1 |
| 9 | 2 | MB | HD | 0.818181818 | 1.130285664 | 1 |
| 9 | 0 | MB | LD | 1 | 1.570796327 | 1 |
| 9 | 0 | MB | HD | 1 | 1.570796327 | 1 |
| 9 | 0 | MB | HD | 1 | 1.570796327 | 1 |
| 9 | 0 | MB | HD | 1 | 1.570796327 | 1 |
| 8 | 3 | MCU | LD | 0.727272727 | 1.021329082 | 4 |
| 8 | 1 | MCU | HD | 0.888888889 | 1.230959417 | 4 |
| 8 | 1 | MCU | HD | 0.888888889 | 1.230959417 | 4 |
| 8 | 4 | MB | HD | 0.666666667 | 0.955316618 | 4 |
| 8 | 4 | MB | HD | 0.666666667 | 0.955316618 | 4 |
| 8 | 2 | MB | HD | 0.8 | 1.107148718 | 4 |
| 8 | 1 | MB | LD | 0.888888889 | 1.230959417 | 4 |
| 8 | 1 | MB | HD | 0.888888889 | 1.230959417 | 4 |
| 8 | 1 | MB | HD | 0.888888889 | 1.230959417 | 4 |
| 8 | 0 | MB | LD | 1 | 1.570796327 | 4 |
| 8 | 0 | MB | HD | 1 | 1.570796327 | 4 |
| 8 | 7 | MCU | HD | 0.533333333 | 0.818756238 | 3 |
| 8 | 4 | MCU | HD | 0.666666667 | 0.955316618 | 3 |
| 8 | 3 | MCU | HD | 0.727272727 | 1.021329082 | 3 |
| 8 | 2 | MCU | HD | 0.8 | 1.107148718 | 3 |
| 8 | 0 | MCU | LD | 1 | 1.570796327 | 3 |
| 8 | 0 | MCU | LD | 1 | 1.570796327 | 3 |
| 8 | 0 | MCU | LD | 1 | 1.570796327 | 3 |
| 8 | 0 | MCU | HD | 1 | 1.570796327 | 3 |
| 8 | 0 | MCU | HD | 1 | 1.570796327 | 3 |
| 8 | 0 | MCU | HD | 1 | 1.570796327 | 3 |
| 8 | 0 | MCU | HD | 1 | 1.570796327 | 3 |
| 8 | 0 | MCU | HD | 1 | 1.570796327 | 3 |
| 8 | 7 | MB | LD | 0.533333333 | 0.818756238 | 3 |
| 8 | 6 | MB | HD | 0.571428571 | 0.857071948 | 3 |
| 8 | 2 | MB | HD | 0.8 | 1.107148718 | 3 |
| 8 | 2 | MB | HD | 0.8 | 1.107148718 | 3 |
| 8 | 1 | MB | HD | 0.888888889 | 1.230959417 | 3 |
| 8 | 0 | MB | HD | 1 | 1.570796327 | 3 |
| 8 | 4 | MCU | HD | 0.666666667 | 0.955316618 | 2 |
| 8 | 6 | MB | HD | 0.571428571 | 0.857071948 | 2 |
| 8 | 5 | MB | HD | 0.615384615 | 0.901832253 | 2 |
| 8 | 2 | MB | HD | 0.8 | 1.107148718 | 2 |
| 8 | 2 | MB | HD | 0.8 | 1.107148718 | 2 |
| 8 | 2 | MB | HD | 0.8 | 1.107148718 | 2 |
| 8 | 1 | MB | LD | 0.888888889 | 1.230959417 | 2 |
| 8 | 1 | MB | LD | 0.888888889 | 1.230959417 | 2 |
| 8 | 1 | MB | HD | 0.888888889 | 1.230959417 | 2 |
| 8 | 0 | MB | LD | 1 | 1.570796327 | 2 |
| 8 | 0 | MB | LD | 1 | 1.570796327 | 2 |
| 8 | 0 | MB | HD | 1 | 1.570796327 | 2 |
| 8 | 0 | MB | HD | 1 | 1.570796327 | 2 |
| 8 | 0 | MB | HD | 1 | 1.570796327 | 2 |
| 8 | 0 | MB | HD | 1 | 1.570796327 | 2 |
| 8 | 0 | MB | HD | 1 | 1.570796327 | 2 |
| 8 | 4 | MCU | HD | 0.666666667 | 0.955316618 | 1 |
| 8 | 3 | MCU | LD | 0.727272727 | 1.021329082 | 1 |
| 8 | 3 | MCU | LD | 0.727272727 | 1.021329082 | 1 |
| 8 | 2 | MCU | LD | 0.8 | 1.107148718 | 1 |
| 8 | 0 | MCU | LD | 1 | 1.570796327 | 1 |
| 8 | 0 | MCU | LD | 1 | 1.570796327 | 1 |
| 8 | 0 | MCU | LD | 1 | 1.570796327 | 1 |
| 8 | 0 | MCU | HD | 1 | 1.570796327 | 1 |
| 8 | 0 | MCU | HD | 1 | 1.570796327 | 1 |
| 8 | 5 | MB | HD | 0.615384615 | 0.901832253 | 1 |
| 8 | 3 | MB | HD | 0.727272727 | 1.021329082 | 1 |
| 8 | 2 | MB | HD | 0.8 | 1.107148718 | 1 |
| 8 | 1 | MB | HD | 0.888888889 | 1.230959417 | 1 |
| 8 | 1 | MB | HD | 0.888888889 | 1.230959417 | 1 |
| 8 | 0 | MB | HD | 1 | 1.570796327 | 1 |
| 7 | 8 | MCU | HD | 0.466666667 | 0.752040089 | 4 |
| 7 | 3 | MCU | LD | 0.7 | 0.991156586 | 4 |
| 7 | 3 | MCU | HD | 0.7 | 0.991156586 | 4 |
| 7 | 3 | MB | LD | 0.7 | 0.991156586 | 4 |
| 7 | 2 | MB | HD | 0.777777778 | 1.079913649 | 4 |
| 7 | 1 | MB | LD | 0.875 | 1.209429203 | 4 |
| 7 | 1 | MB | HD | 0.875 | 1.209429203 | 4 |
| 7 | 1 | MB | HD | 0.875 | 1.209429203 | 4 |
| 7 | 0 | MB | HD | 1 | 1.570796327 | 4 |
| 7 | 0 | MB | HD | 1 | 1.570796327 | 4 |
| 7 | 0 | MB | HD | 1 | 1.570796327 | 4 |
| 7 | 5 | MCU | LD | 0.583333333 | 0.869122203 | 3 |
| 7 | 5 | MCU | HD | 0.583333333 | 0.869122203 | 3 |
| 7 | 4 | MCU | HD | 0.636363636 | 0.923511479 | 3 |
| 7 | 3 | MCU | HD | 0.7 | 0.991156586 | 3 |
| 7 | 2 | MCU | HD | 0.777777778 | 1.079913649 | 3 |
| 7 | 2 | MCU | HD | 0.777777778 | 1.079913649 | 3 |
| 7 | 1 | MCU | LD | 0.875 | 1.209429203 | 3 |
| 7 | 1 | MCU | LD | 0.875 | 1.209429203 | 3 |
| 7 | 1 | MCU | HD | 0.875 | 1.209429203 | 3 |
| 7 | 1 | MCU | HD | 0.875 | 1.209429203 | 3 |
| 7 | 1 | MCU | HD | 0.875 | 1.209429203 | 3 |
| 7 | 0 | MCU | LD | 1 | 1.570796327 | 3 |
| 7 | 0 | MCU | LD | 1 | 1.570796327 | 3 |
| 7 | 0 | MCU | HD | 1 | 1.570796327 | 3 |
| 7 | 0 | MCU | HD | 1 | 1.570796327 | 3 |
| 7 | 0 | MCU | HD | 1 | 1.570796327 | 3 |
| 7 | 0 | MCU | HD | 1 | 1.570796327 | 3 |
| 7 | 6 | MB | HD | 0.538461538 | 0.823897734 | 3 |
| 7 | 3 | MB | HD | 0.7 | 0.991156586 | 3 |
| 7 | 2 | MB | LD | 0.777777778 | 1.079913649 | 3 |
| 7 | 1 | MB | LD | 0.875 | 1.209429203 | 3 |
| 7 | 1 | MB | LD | 0.875 | 1.209429203 | 3 |
| 7 | 1 | MB | HD | 0.875 | 1.209429203 | 3 |
| 7 | 0 | MB | HD | 1 | 1.570796327 | 3 |
| 7 | 0 | MB | HD | 1 | 1.570796327 | 3 |
| 7 | 0 | MB | HD | 1 | 1.570796327 | 3 |
| 7 | 0 | MB | HD | 1 | 1.570796327 | 3 |
| 7 | 3 | MCU | HD | 0.7 | 0.991156586 | 2 |
| 7 | 2 | MCU | HD | 0.777777778 | 1.079913649 | 2 |
| 7 | 2 | MCU | HD | 0.777777778 | 1.079913649 | 2 |
| 7 | 1 | MCU | LD | 0.875 | 1.209429203 | 2 |
| 7 | 1 | MCU | HD | 0.875 | 1.209429203 | 2 |
| 7 | 0 | MCU | HD | 1 | 1.570796327 | 2 |
| 7 | 0 | MCU | HD | 1 | 1.570796327 | 2 |
| 7 | 0 | MCU | HD | 1 | 1.570796327 | 2 |
| 7 | 2 | MB | HD | 0.777777778 | 1.079913649 | 2 |
| 7 | 1 | MB | HD | 0.875 | 1.209429203 | 2 |
| 7 | 1 | MB | HD | 0.875 | 1.209429203 | 2 |
| 7 | 0 | MB | LD | 1 | 1.570796327 | 2 |
| 7 | 0 | MB | LD | 1 | 1.570796327 | 2 |
| 7 | 0 | MB | HD | 1 | 1.570796327 | 2 |
| 7 | 5 | MCU | HD | 0.583333333 | 0.869122203 | 1 |
| 7 | 4 | MCU | HD | 0.636363636 | 0.923511479 | 1 |
| 7 | 3 | MCU | LD | 0.7 | 0.991156586 | 1 |
| 7 | 3 | MCU | HD | 0.7 | 0.991156586 | 1 |
| 7 | 3 | MCU | HD | 0.7 | 0.991156586 | 1 |
| 7 | 1 | MCU | LD | 0.875 | 1.209429203 | 1 |
| 7 | 1 | MCU | LD | 0.875 | 1.209429203 | 1 |
| 7 | 1 | MCU | HD | 0.875 | 1.209429203 | 1 |
| 7 | 1 | MCU | HD | 0.875 | 1.209429203 | 1 |
| 7 | 1 | MCU | HD | 0.875 | 1.209429203 | 1 |
| 7 | 0 | MCU | HD | 1 | 1.570796327 | 1 |
| 7 | 0 | MCU | HD | 1 | 1.570796327 | 1 |
| 7 | 5 | MB | HD | 0.583333333 | 0.869122203 | 1 |
| 7 | 5 | MB | HD | 0.583333333 | 0.869122203 | 1 |
| 7 | 3 | MB | HD | 0.7 | 0.991156586 | 1 |
| 7 | 2 | MB | HD | 0.777777778 | 1.079913649 | 1 |
| 7 | 1 | MB | HD | 0.875 | 1.209429203 | 1 |
| 7 | 1 | MB | HD | 0.875 | 1.209429203 | 1 |
| 7 | 1 | MB | HD | 0.875 | 1.209429203 | 1 |
| 7 | 0 | MB | HD | 1 | 1.570796327 | 1 |
| 6 | 2 | MCU | LD | 0.75 | 1.047197551 | 4 |
| 6 | 0 | MCU | LD | 1 | 1.570796327 | 4 |
| 6 | 4 | MB | HD | 0.6 | 0.886077124 | 4 |
| 6 | 3 | MB | HD | 0.666666667 | 0.955316618 | 4 |
| 6 | 2 | MB | LD | 0.75 | 1.047197551 | 4 |
| 6 | 1 | MB | HD | 0.857142857 | 1.18319964 | 4 |
| 6 | 1 | MB | HD | 0.857142857 | 1.18319964 | 4 |
| 6 | 0 | MB | LD | 1 | 1.570796327 | 4 |
| 6 | 0 | MB | HD | 1 | 1.570796327 | 4 |
| 6 | 4 | MCU | LD | 0.6 | 0.886077124 | 3 |
| 6 | 4 | MCU | HD | 0.6 | 0.886077124 | 3 |
| 6 | 3 | MCU | HD | 0.666666667 | 0.955316618 | 3 |
| 6 | 2 | MCU | HD | 0.75 | 1.047197551 | 3 |
| 6 | 1 | MCU | LD | 0.857142857 | 1.18319964 | 3 |
| 6 | 1 | MCU | HD | 0.857142857 | 1.18319964 | 3 |
| 6 | 1 | MCU | HD | 0.857142857 | 1.18319964 | 3 |
| 6 | 0 | MCU | LD | 1 | 1.570796327 | 3 |
| 6 | 11 | MB | LD | 0.352941176 | 0.636132063 | 3 |
| 6 | 3 | MB | HD | 0.666666667 | 0.955316618 | 3 |
| 6 | 2 | MB | HD | 0.75 | 1.047197551 | 3 |
| 6 | 2 | MB | HD | 0.75 | 1.047197551 | 3 |
| 6 | 1 | MB | LD | 0.857142857 | 1.18319964 | 3 |
| 6 | 0 | MB | LD | 1 | 1.570796327 | 3 |
| 6 | 0 | MB | LD | 1 | 1.570796327 | 3 |
| 6 | 0 | MB | LD | 1 | 1.570796327 | 3 |
| 6 | 0 | MB | LD | 1 | 1.570796327 | 3 |
| 6 | 5 | MCU | HD | 0.545454545 | 0.830915552 | 2 |
| 6 | 3 | MCU | LD | 0.666666667 | 0.955316618 | 2 |
| 6 | 3 | MCU | HD | 0.666666667 | 0.955316618 | 2 |
| 6 | 2 | MCU | LD | 0.75 | 1.047197551 | 2 |
| 6 | 2 | MCU | LD | 0.75 | 1.047197551 | 2 |
| 6 | 2 | MCU | HD | 0.75 | 1.047197551 | 2 |
| 6 | 2 | MCU | HD | 0.75 | 1.047197551 | 2 |
| 6 | 1 | MCU | LD | 0.857142857 | 1.18319964 | 2 |
| 6 | 1 | MCU | LD | 0.857142857 | 1.18319964 | 2 |
| 6 | 1 | MCU | LD | 0.857142857 | 1.18319964 | 2 |
| 6 | 1 | MCU | HD | 0.857142857 | 1.18319964 | 2 |
| 6 | 0 | MCU | LD | 1 | 1.570796327 | 2 |
| 6 | 0 | MCU | LD | 1 | 1.570796327 | 2 |
| 6 | 0 | MCU | LD | 1 | 1.570796327 | 2 |
| 6 | 0 | MCU | LD | 1 | 1.570796327 | 2 |
| 6 | 0 | MCU | LD | 1 | 1.570796327 | 2 |
| 6 | 0 | MCU | HD | 1 | 1.570796327 | 2 |
| 6 | 4 | MB | HD | 0.6 | 0.886077124 | 2 |
| 6 | 3 | MB | HD | 0.666666667 | 0.955316618 | 2 |
| 6 | 2 | MB | HD | 0.75 | 1.047197551 | 2 |
| 6 | 2 | MB | HD | 0.75 | 1.047197551 | 2 |
| 6 | 2 | MB | HD | 0.75 | 1.047197551 | 2 |
| 6 | 1 | MB | HD | 0.857142857 | 1.18319964 | 2 |
| 6 | 1 | MB | HD | 0.857142857 | 1.18319964 | 2 |
| 6 | 0 | MB | LD | 1 | 1.570796327 | 2 |
| 6 | 0 | MB | LD | 1 | 1.570796327 | 2 |
| 6 | 0 | MB | HD | 1 | 1.570796327 | 2 |
| 6 | 0 | MB | HD | 1 | 1.570796327 | 2 |
| 6 | 0 | MB | HD | 1 | 1.570796327 | 2 |
| 6 | 0 | MB | HD | 1 | 1.570796327 | 2 |
| 6 | 14 | MCU | HD | 0.3 | 0.57963974 | 1 |
| 6 | 7 | MCU | HD | 0.461538462 | 0.746898593 | 1 |
| 6 | 3 | MCU | LD | 0.666666667 | 0.955316618 | 1 |
| 6 | 3 | MCU | HD | 0.666666667 | 0.955316618 | 1 |
| 6 | 1 | MCU | LD | 0.857142857 | 1.18319964 | 1 |
| 6 | 2 | MB | HD | 0.75 | 1.047197551 | 1 |
| 6 | 2 | MB | HD | 0.75 | 1.047197551 | 1 |
| 6 | 1 | MB | HD | 0.857142857 | 1.18319964 | 1 |
| 6 | 1 | MB | HD | 0.857142857 | 1.18319964 | 1 |
| 6 | 0 | MB | LD | 1 | 1.570796327 | 1 |
| 6 | 0 | MB | LD | 1 | 1.570796327 | 1 |
| 6 | 0 | MB | LD | 1 | 1.570796327 | 1 |
| 5 | 3 | MCU | LD | 0.625 | 0.911738291 | 4 |
| 5 | 1 | MCU | HD | 0.833333333 | 1.150261992 | 4 |
| 5 | 0 | MCU | LD | 1 | 1.570796327 | 4 |
| 5 | 6 | MB | HD | 0.454545455 | 0.739880774 | 4 |
| 5 | 3 | MB | LD | 0.625 | 0.911738291 | 4 |
| 5 | 3 | MB | HD | 0.625 | 0.911738291 | 4 |
| 5 | 3 | MB | HD | 0.625 | 0.911738291 | 4 |
| 5 | 3 | MB | HD | 0.625 | 0.911738291 | 4 |
| 5 | 2 | MB | LD | 0.714285714 | 1.006853685 | 4 |
| 5 | 0 | MB | LD | 1 | 1.570796327 | 4 |
| 5 | 0 | MB | HD | 1 | 1.570796327 | 4 |
| 5 | 13 | MCU | LD | 0.277777778 | 0.555121168 | 3 |
| 5 | 2 | MCU | LD | 0.714285714 | 1.006853685 | 3 |
| 5 | 1 | MCU | LD | 0.833333333 | 1.150261992 | 3 |
| 5 | 1 | MCU | LD | 0.833333333 | 1.150261992 | 3 |
| 5 | 1 | MCU | HD | 0.833333333 | 1.150261992 | 3 |
| 5 | 0 | MCU | HD | 1 | 1.570796327 | 3 |
| 5 | 0 | MCU | HD | 1 | 1.570796327 | 3 |
| 5 | 0 | MCU | HD | 1 | 1.570796327 | 3 |
| 5 | 0 | MCU | HD | 1 | 1.570796327 | 3 |
| 5 | 3 | MB | LD | 0.625 | 0.911738291 | 3 |
| 5 | 3 | MB | HD | 0.625 | 0.911738291 | 3 |
| 5 | 2 | MB | LD | 0.714285714 | 1.006853685 | 3 |
| 5 | 2 | MB | HD | 0.714285714 | 1.006853685 | 3 |
| 5 | 2 | MB | HD | 0.714285714 | 1.006853685 | 3 |
| 5 | 0 | MB | LD | 1 | 1.570796327 | 3 |
| 5 | 0 | MB | LD | 1 | 1.570796327 | 3 |
| 5 | 0 | MB | HD | 1 | 1.570796327 | 3 |
| 5 | 8 | MCU | LD | 0.384615385 | 0.668964074 | 2 |
| 5 | 8 | MCU | HD | 0.384615385 | 0.668964074 | 2 |
| 5 | 2 | MCU | LD | 0.714285714 | 1.006853685 | 2 |
| 5 | 2 | MCU | LD | 0.714285714 | 1.006853685 | 2 |
| 5 | 1 | MCU | HD | 0.833333333 | 1.150261992 | 2 |
| 5 | 0 | MCU | LD | 1 | 1.570796327 | 2 |
| 5 | 0 | MCU | LD | 1 | 1.570796327 | 2 |
| 5 | 0 | MCU | HD | 1 | 1.570796327 | 2 |
| 5 | 0 | MCU | HD | 1 | 1.570796327 | 2 |
| 5 | 0 | MCU | HD | 1 | 1.570796327 | 2 |
| 5 | 0 | MB | HD | 1 | 1.570796327 | 2 |
| 5 | 0 | MB | HD | 1 | 1.570796327 | 2 |
| 5 | 0 | MB | HD | 1 | 1.570796327 | 2 |
| 5 | 5 | MCU | HD | 0.5 | 0.785398163 | 1 |
| 5 | 4 | MCU | LD | 0.555555556 | 0.841068671 | 1 |
| 5 | 4 | MCU | HD | 0.555555556 | 0.841068671 | 1 |
| 5 | 4 | MCU | HD | 0.555555556 | 0.841068671 | 1 |
| 5 | 3 | MCU | HD | 0.625 | 0.911738291 | 1 |
| 5 | 3 | MCU | HD | 0.625 | 0.911738291 | 1 |
| 5 | 2 | MCU | LD | 0.714285714 | 1.006853685 | 1 |
| 5 | 1 | MCU | LD | 0.833333333 | 1.150261992 | 1 |
| 5 | 1 | MCU | HD | 0.833333333 | 1.150261992 | 1 |
| 5 | 9 | MB | HD | 0.357142857 | 0.640522313 | 1 |
| 5 | 6 | MB | LD | 0.454545455 | 0.739880774 | 1 |
| 5 | 6 | MB | HD | 0.454545455 | 0.739880774 | 1 |
| 5 | 6 | MB | HD | 0.454545455 | 0.739880774 | 1 |
| 5 | 2 | MB | HD | 0.714285714 | 1.006853685 | 1 |
| 5 | 0 | MB | LD | 1 | 1.570796327 | 1 |
| 4 | 3 | MCU | LD | 0.571428571 | 0.857071948 | 4 |
| 4 | 2 | MCU | LD | 0.666666667 | 0.955316618 | 4 |
| 4 | 2 | MCU | HD | 0.666666667 | 0.955316618 | 4 |
| 4 | 1 | MCU | LD | 0.8 | 1.107148718 | 4 |
| 4 | 1 | MCU | HD | 0.8 | 1.107148718 | 4 |
| 4 | 0 | MCU | LD | 1 | 1.570796327 | 4 |
| 4 | 0 | MCU | HD | 1 | 1.570796327 | 4 |
| 4 | 0 | MCU | HD | 1 | 1.570796327 | 4 |
| 4 | 5 | MB | LD | 0.444444444 | 0.729727656 | 4 |
| 4 | 3 | MB | LD | 0.571428571 | 0.857071948 | 4 |
| 4 | 3 | MB | HD | 0.571428571 | 0.857071948 | 4 |
| 4 | 3 | MB | HD | 0.571428571 | 0.857071948 | 4 |
| 4 | 1 | MB | LD | 0.8 | 1.107148718 | 4 |
| 4 | 1 | MB | HD | 0.8 | 1.107148718 | 4 |
| 4 | 0 | MB | LD | 1 | 1.570796327 | 4 |
| 4 | 0 | MB | HD | 1 | 1.570796327 | 4 |
| 4 | 4 | MCU | LD | 0.5 | 0.785398163 | 3 |
| 4 | 4 | MCU | HD | 0.5 | 0.785398163 | 3 |
| 4 | 2 | MCU | HD | 0.666666667 | 0.955316618 | 3 |
| 4 | 2 | MCU | HD | 0.666666667 | 0.955316618 | 3 |
| 4 | 1 | MCU | HD | 0.8 | 1.107148718 | 3 |
| 4 | 1 | MCU | HD | 0.8 | 1.107148718 | 3 |
| 4 | 0 | MCU | HD | 1 | 1.570796327 | 3 |
| 4 | 0 | MCU | HD | 1 | 1.570796327 | 3 |
| 4 | 11 | MB | HD | 0.266666667 | 0.542639102 | 3 |
| 4 | 4 | MB | LD | 0.5 | 0.785398163 | 3 |
| 4 | 4 | MB | HD | 0.5 | 0.785398163 | 3 |
| 4 | 3 | MB | LD | 0.571428571 | 0.857071948 | 3 |
| 4 | 3 | MB | HD | 0.571428571 | 0.857071948 | 3 |
| 4 | 3 | MB | HD | 0.571428571 | 0.857071948 | 3 |
| 4 | 3 | MB | HD | 0.571428571 | 0.857071948 | 3 |
| 4 | 2 | MB | LD | 0.666666667 | 0.955316618 | 3 |
| 4 | 2 | MB | LD | 0.666666667 | 0.955316618 | 3 |
| 4 | 1 | MB | HD | 0.8 | 1.107148718 | 3 |
| 4 | 1 | MB | HD | 0.8 | 1.107148718 | 3 |
| 4 | 0 | MB | LD | 1 | 1.570796327 | 3 |
| 4 | 0 | MB | HD | 1 | 1.570796327 | 3 |
| 4 | 0 | MB | HD | 1 | 1.570796327 | 3 |
| 4 | 0 | MB | HD | 1 | 1.570796327 | 3 |
| 4 | 4 | MCU | LD | 0.5 | 0.785398163 | 2 |
| 4 | 3 | MCU | LD | 0.571428571 | 0.857071948 | 2 |
| 4 | 3 | MCU | HD | 0.571428571 | 0.857071948 | 2 |
| 4 | 2 | MCU | LD | 0.666666667 | 0.955316618 | 2 |
| 4 | 0 | MCU | HD | 1 | 1.570796327 | 2 |
| 4 | 3 | MB | HD | 0.571428571 | 0.857071948 | 2 |
| 4 | 2 | MB | LD | 0.666666667 | 0.955316618 | 2 |
| 4 | 2 | MB | LD | 0.666666667 | 0.955316618 | 2 |
| 4 | 2 | MB | HD | 0.666666667 | 0.955316618 | 2 |
| 4 | 0 | MB | HD | 1 | 1.570796327 | 2 |
| 4 | 0 | MB | HD | 1 | 1.570796327 | 2 |
| 4 | 5 | MCU | HD | 0.444444444 | 0.729727656 | 1 |
| 4 | 2 | MCU | HD | 0.666666667 | 0.955316618 | 1 |
| 4 | 2 | MCU | HD | 0.666666667 | 0.955316618 | 1 |
| 4 | 0 | MCU | LD | 1 | 1.570796327 | 1 |
| 4 | 0 | MCU | LD | 1 | 1.570796327 | 1 |
| 4 | 0 | MCU | LD | 1 | 1.570796327 | 1 |
| 4 | 0 | MCU | LD | 1 | 1.570796327 | 1 |
| 4 | 0 | MCU | LD | 1 | 1.570796327 | 1 |
| 4 | 0 | MCU | HD | 1 | 1.570796327 | 1 |
| 4 | 17 | MB | HD | 0.19047619 | 0.451633441 | 1 |
| 4 | 3 | MB | HD | 0.571428571 | 0.857071948 | 1 |
| 4 | 0 | MB | LD | 1 | 1.570796327 | 1 |
| 4 | 0 | MB | HD | 1 | 1.570796327 | 1 |
| 4 | 0 | MB | HD | 1 | 1.570796327 | 1 |
| 3 | 10 | MCU | HD | 0.230769231 | 0.501093013 | 4 |
| 3 | 1 | MCU | HD | 0.75 | 1.047197551 | 4 |
| 3 | 0 | MCU | LD | 1 | 1.570796327 | 4 |
| 3 | 0 | MCU | HD | 1 | 1.570796327 | 4 |
| 3 | 10 | MB | HD | 0.230769231 | 0.501093013 | 4 |
| 3 | 3 | MB | HD | 0.5 | 0.785398163 | 4 |
| 3 | 3 | MB | HD | 0.5 | 0.785398163 | 4 |
| 3 | 2 | MB | LD | 0.6 | 0.886077124 | 4 |
| 3 | 0 | MB | HD | 1 | 1.570796327 | 4 |
| 3 | 9 | MCU | HD | 0.25 | 0.523598776 | 3 |
| 3 | 2 | MCU | HD | 0.6 | 0.886077124 | 3 |
| 3 | 1 | MCU | LD | 0.75 | 1.047197551 | 3 |
| 3 | 0 | MCU | LD | 1 | 1.570796327 | 3 |
| 3 | 0 | MCU | HD | 1 | 1.570796327 | 3 |
| 3 | 4 | MB | LD | 0.428571429 | 0.713724379 | 3 |
| 3 | 1 | MB | HD | 0.75 | 1.047197551 | 3 |
| 3 | 1 | MB | HD | 0.75 | 1.047197551 | 3 |
| 3 | 1 | MB | HD | 0.75 | 1.047197551 | 3 |
| 3 | 0 | MB | LD | 1 | 1.570796327 | 3 |
| 3 | 7 | MCU | LD | 0.3 | 0.57963974 | 2 |
| 3 | 4 | MCU | HD | 0.428571429 | 0.713724379 | 2 |
| 3 | 3 | MCU | HD | 0.5 | 0.785398163 | 2 |
| 3 | 1 | MCU | HD | 0.75 | 1.047197551 | 2 |
| 3 | 0 | MCU | HD | 1 | 1.570796327 | 2 |
| 3 | 3 | MB | LD | 0.5 | 0.785398163 | 2 |
| 3 | 3 | MB | HD | 0.5 | 0.785398163 | 2 |
| 3 | 2 | MB | LD | 0.6 | 0.886077124 | 2 |
| 3 | 2 | MB | HD | 0.6 | 0.886077124 | 2 |
| 3 | 1 | MB | LD | 0.75 | 1.047197551 | 2 |
| 3 | 1 | MB | HD | 0.75 | 1.047197551 | 2 |
| 3 | 0 | MB | LD | 1 | 1.570796327 | 2 |
| 3 | 1 | MCU | HD | 0.75 | 1.047197551 | 1 |
| 3 | 1 | MB | HD | 0.75 | 1.047197551 | 1 |
| 3 | 0 | MB | HD | 1 | 1.570796327 | 1 |
| 2 | 8 | MCU | HD | 0.2 | 0.463647609 | 4 |
| 2 | 0 | MCU | HD | 1 | 1.570796327 | 4 |
| 2 | 3 | MB | HD | 0.4 | 0.684719203 | 4 |
| 2 | 12 | MCU | HD | 0.142857143 | 0.387596687 | 3 |
| 2 | 2 | MCU | HD | 0.5 | 0.785398163 | 3 |
| 2 | 2 | MCU | HD | 0.5 | 0.785398163 | 3 |
| 2 | 2 | MCU | HD | 0.5 | 0.785398163 | 3 |
| 2 | 0 | MCU | LD | 1 | 1.570796327 | 3 |
| 2 | 4 | MB | HD | 0.333333333 | 0.615479709 | 3 |
| 2 | 1 | MB | HD | 0.666666667 | 0.955316618 | 3 |
| 2 | 1 | MB | HD | 0.666666667 | 0.955316618 | 3 |
| 2 | 0 | MB | LD | 1 | 1.570796327 | 3 |
| 2 | 5 | MCU | HD | 0.285714286 | 0.563942641 | 2 |
| 2 | 4 | MCU | HD | 0.333333333 | 0.615479709 | 2 |
| 2 | 2 | MCU | LD | 0.5 | 0.785398163 | 2 |
| 2 | 0 | MCU | HD | 1 | 1.570796327 | 2 |
| 2 | 0 | MCU | HD | 1 | 1.570796327 | 2 |
| 2 | 9 | MB | LD | 0.181818182 | 0.440510663 | 2 |
| 2 | 5 | MB | HD | 0.285714286 | 0.563942641 | 2 |
| 2 | 3 | MB | HD | 0.4 | 0.684719203 | 2 |
| 2 | 2 | MB | HD | 0.5 | 0.785398163 | 2 |
| 2 | 0 | MB | LD | 1 | 1.570796327 | 2 |
| 2 | 0 | MB | HD | 1 | 1.570796327 | 2 |
| 2 | 0 | MB | HD | 1 | 1.570796327 | 2 |
| 2 | 0 | MB | HD | 1 | 1.570796327 | 2 |
| 2 | 0 | MB | HD | 1 | 1.570796327 | 2 |
| 2 | 10 | MCU | HD | 0.166666667 | 0.420534335 | 1 |
| 2 | 4 | MCU | LD | 0.333333333 | 0.615479709 | 1 |
| 2 | 4 | MCU | HD | 0.333333333 | 0.615479709 | 1 |
| 2 | 4 | MCU | HD | 0.333333333 | 0.615479709 | 1 |
| 2 | 1 | MCU | LD | 0.666666667 | 0.955316618 | 1 |
| 2 | 1 | MCU | HD | 0.666666667 | 0.955316618 | 1 |
| 2 | 0 | MCU | HD | 1 | 1.570796327 | 1 |
| 2 | 0 | MCU | HD | 1 | 1.570796327 | 1 |
| 2 | 0 | MCU | HD | 1 | 1.570796327 | 1 |
| 2 | 11 | MB | HD | 0.153846154 | 0.403057074 | 1 |
| 2 | 5 | MB | HD | 0.285714286 | 0.563942641 | 1 |
| 1 | 22 | MCU | HD | 0.043478261 | 0.210055739 | 4 |
| 1 | 10 | MCU | HD | 0.090909091 | 0.306277369 | 4 |
| 1 | 8 | MCU | HD | 0.111111111 | 0.339836909 | 4 |
| 1 | 4 | MCU | HD | 0.2 | 0.463647609 | 4 |
| 1 | 2 | MCU | LD | 0.333333333 | 0.615479709 | 4 |
| 1 | 3 | MB | HD | 0.25 | 0.523598776 | 4 |
| 1 | 0 | MB | LD | 1 | 1.570796327 | 4 |
| 1 | 6 | MCU | HD | 0.142857143 | 0.387596687 | 3 |
| 1 | 4 | MCU | HD | 0.2 | 0.463647609 | 3 |
| 1 | 2 | MCU | LD | 0.333333333 | 0.615479709 | 3 |
| 1 | 0 | MCU | LD | 1 | 1.570796327 | 3 |
| 1 | 9 | MB | LD | 0.1 | 0.321750554 | 3 |
| 1 | 3 | MB | HD | 0.25 | 0.523598776 | 3 |
| 1 | 1 | MB | HD | 0.5 | 0.785398163 | 3 |
| 1 | 2 | MCU | LD | 0.333333333 | 0.615479709 | 2 |
| 1 | 1 | MCU | HD | 0.5 | 0.785398163 | 2 |
| 1 | 1 | MCU | HD | 0.5 | 0.785398163 | 2 |
| 1 | 0 | MCU | LD | 1 | 1.570796327 | 2 |
| 1 | 0 | MCU | LD | 1 | 1.570796327 | 2 |
| 1 | 0 | MCU | HD | 1 | 1.570796327 | 2 |
| 1 | 0 | MCU | HD | 1 | 1.570796327 | 2 |
| 1 | 0 | MB | HD | 1 | 1.570796327 | 2 |
| 1 | 9 | MCU | HD | 0.1 | 0.321750554 | 1 |
| 1 | 2 | MCU | HD | 0.333333333 | 0.615479709 | 1 |
| 1 | 2 | MCU | HD | 0.333333333 | 0.615479709 | 1 |
| 1 | 1 | MCU | HD | 0.5 | 0.785398163 | 1 |
| 1 | 1 | MB | HD | 0.5 | 0.785398163 | 1 |
| 1 | 0 | MB | HD | 1 | 1.570796327 | 1 |

Supplementary Table 7: Results of logistic regression in sperm Defense assay

|  | **Estimate** | **Std. Error** | **z value** | **Pr(>\|z\|)** |
| --- | --- | --- | --- | --- |
| **(Intercept)** | 1.798 | 0.211 | 8.492 | <0.001 |
| **SelectionMCU** | -0.948 | 0.278 | -3.409 | 0.001 |
| **TreatmentLD** | -0.065 | 0.286 | -0.228 | 0.819 |
| **SelectionMCU:TreatmentLD** | 1.243 | 0.399 | 3.114 | 0.002 |

Supplementary Table 8: Results of GLMM model for sperm Defense ability

|  | **Estimate** | **Std. Error** | **z value** | **Pr(>\|z\|)** |
| --- | --- | --- | --- | --- |
| **(Intercept)** | -0.440 | 0.115 | -3.844 | 0.001 |
| **SelectionMCU** | -0.659 | 0.069 | -9.527 | <0.001 |
| **TreatmentLD** | -20.193 | 0.062 | 3.128 | 0.0017 |
| **SelectionMCU:TreatmentLD** | 0.509 | 0.092 | 5.5018 | <0.001 |

Supplementary Table 9: Effects of selection regime and larval crowding treatment on red eye female progenies by males 4 days post-eclosion during a sperm Defense assay. The table presents F-statistic (F), degrees of freedom (Df), residual degrees of freedom (Df.res), and the probability value (Pr(>F)) for each term in the model. Significant effects are marked with an asterisk (*), indicating a p-value < 0.05

|  | **Chisq** | **Df** | **Pr(>Chisq)** |
| --- | --- | --- | --- |
| **(Intercept)** | 62.674 | 1 | <0.001 |
| **Selection** | 5.583 | 1 | 0.018 |
| **Treatment** | 2.079 | 1 | 0.1493 |
| **Selection:Treatment** | 2.031 | 1 | 0.154 |

Supplementary Table 10: Pairwise comparisons of sperm offense ability among 4-day-old males under different treatments

| contrast | estimate | SE | df | t.ratio | p.value |
| --- | --- | --- | --- | --- | --- |
| MBHD - MCU HD | 0.0313 | 0.0241 | 1208 | 1.299 | 0.5639 |
| MBHD -MBLD | -0.1359 | 0.0284 | 1208 | -4.79 | <.0001 |
| MBHD -MCULD | -0.1855 | 0.028 | 1208 | -6.627 | <.0001 |
| MCUHD-MBLD | -0.1672 | 0.0279 | 1208 | -5.993 | <.0001 |
| MCUHD-MCULD | -0.2168 | 0.0275 | 1208 | -7.879 | <.0001 |
| MBLD-MCULD | -0.0496 | 0.0313 | 1208 | -1.584 | 0.3882 |

Supplementary Table 11:Pairwise comparisons of sperm offense ability among 9-day-old males under different treatments

| contrast | estimate | SE | df | t.ratio | p.value |
| --- | --- | --- | --- | --- | --- |
| MB HD - MCU HD | 0.0309 | 0.0251 | 775 | 1.227 | 0.6098 |
| MB HD - MB LD | -0.0873 | 0.0308 | 775 | -2.83 | 0.0246 |
| MB HD - MCU LD | -0.0257 | 0.0275 | 773 | -0.934 | 0.7868 |
| MCU HD - MB LD | -0.1182 | 0.0317 | 768 | -3.732 | 0.0012 |
| MCU HD - MCU LD | -0.0566 | 0.0284 | 775 | -1.994 | 0.1911 |
| MB LD - MCU LD | 0.0616 | 0.0335 | 775 | 1.837 | 0.2566 |

Supplementary Table 12: Results of GLMM for sperm offense assay for 4 days old males

|  | **Estimate** | **Std. Error** | **z value** | **Pr(>\|z\|)** |
| --- | --- | --- | --- | --- |
| **(Intercept)** | 0.922 | 0.157 | 5.855 | <0.001 |
| **SelectionMCU** | -0.065 | 0.055 | -1.175 | 0.239 |
| **TreatmentLD** | 0.419 | 0.0673 | 6.233 | <0.001 |
| **SelectionMCU:TreatmentLD** | 0.504 | 0.0983 | 5.1260 | <0.001 |

Supplementary Table 13: Results of GLMM for sperm offense assay for 9 days old males

|  | **Estimate** | **Std. Error** | **z value** | **Pr(>\|z\|)** |
| --- | --- | --- | --- | --- |
| **(Intercept)** | 1.358 | 0.088 | 15.390 | <0.001 |
| **SelectionMCU** | -0.180 | 0.072 | 2.483 | 0.013 |
| **TreatmentLD** | 0.342 | 0.094 | 3.641 | 0.0003 |
| **SelectionMCU:TreatmentLD** | 0.182 | 0.128 | 1.432 | 0.152 |

Supplementary Table 14: Effects of selection regime and larval crowding treatment on red eye female progenies by males 4 days post-eclosion during a sperm offense assay. The table presents F-statistic (F), degrees of freedom (Df), residual degrees of freedom (Df.res), and the probability value (Pr(>F)) for each term in the model. Significant effects are marked with an asterisk (*), indicating a p-value < 0.05

|  | **Chisq** | **Df** | **Pr(>Chisq)** |
| --- | --- | --- | --- |
| **(Intercept)** | 32.354 | 1 | <0.001 |
| **Selection** | 0.541 | 1 | 0.462 |
| **Treatment** | 40.389 | 1 | <0.001 |
| **Selection:Treatment** | 0.173 | 1 | 0.678 |

Supplementary Table 15: Effects of selection regime and larval crowding treatment on red eye female progenies by males 9 days post-eclosion during a sperm offense assay. The table presents F-statistic (F), degrees of freedom (Df), residual degrees of freedom (Df.res), and the probability value (Pr(>F)) for each term in the model. Significant effects are marked with an asterisk (*), indicating a p-value < 0.05

|  | **Chisq** | **Df** | **Pr(>Chisq)** |
| --- | --- | --- | --- |
| **(Intercept)** | 375.002 | 1 | <0.001 |
| **Selection** | 2.848 | 1 | 0.092 |
| **Treatment** | 3.179 | 1 | 0.0746 |
| **Selection:Treatment** | 0.611 | 1 | 0.434 |
